# The NRF2-Dependent Transcriptional Regulation of Antioxidant Defense Pathways: Relevance for Cell Type-Specific Vulnerability to Neurodegeneration and Therapeutic Intervention

**DOI:** 10.3390/antiox11010008

**Published:** 2021-12-21

**Authors:** Stephanie M. Boas, Kathlene L. Joyce, Rita M. Cowell

**Affiliations:** 1 Department of Neuroscience, Southern Research, 2000 9th Avenue South, Birmingham, AL 35205, USA; boas2@uab.edu (S.M.B.); kjoyce@uab.edu (K.L.J.); 2 Department of Cell, Developmental, and Integrative Biology, University of Alabama at Birmingham, 1720 2nd Avenue South, Birmingham, AL 35294, USA

**Keywords:** transcription, antioxidant defense, central nervous system, astrocytes, KEAP1

## Abstract

Oxidative stress has been implicated in the etiology and pathobiology of various neurodegenerative diseases. At baseline, the cells of the nervous system have the capability to regulate the genes for antioxidant defenses by engaging nuclear factor erythroid 2 (NFE2/NRF)-dependent transcriptional mechanisms, and a number of strategies have been proposed to activate these pathways to promote neuroprotection. Here, we briefly review the biology of the transcription factors of the NFE2/NRF family in the brain and provide evidence for the differential cellular localization of NFE2/NRF family members in the cells of the nervous system. We then discuss these findings in the context of the oxidative stress observed in two neurodegenerative diseases, Parkinson’s disease (PD) and amyotrophic lateral sclerosis (ALS), and present current strategies for activating NFE2/NRF-dependent transcription. Based on the expression of the NFE2/NRF family members in restricted populations of neurons and glia, we propose that, when designing strategies to engage these pathways for neuroprotection, the relative contributions of neuronal and non-neuronal cell types to the overall oxidative state of tissue should be considered, as well as the cell types which have the greatest intrinsic capacity for producing antioxidant enzymes.

## 1. Introduction

In multiple neurodegenerative diseases, substantial evidence has implicated oxidative stress as a major pathological contributor (reviewed in [1,2,3]). An underutilized strategy has been taking advantage of intrinsic transcriptional pathways for the regulation of genes involved in antioxidant defense. However, the transcriptional regulators and their cell type-specific expressions must be further explored before strategies can be developed to target the appropriate cell types in vivo. Here, we summarize the state of knowledge on members of the NFE2/NRF transcription factor family. Then, we explore their cell type-specific distributions in the cells of the nervous system using a publicly available single-cell transcriptomics database generated in the mouse brain [4]. Interestingly, mRNA expression patterns for the NRF transcription factors 1–3, encoded by *Nfe2l1* (NRF1), *Nfe2l2* (NRF2), *Nfe2l3* (NRF3), and their interacting partners show distinct cell type-specific distributions in neurons and glia. We go on to discuss the evidence for oxidative stress in neurodegenerative diseases, focusing specifically on those with confirmed risk-associated mutations in oxidative stress response genes: Parkinson’s disease (PD) and amyotrophic lateral sclerosis (ALS). In the context of these disorders, we discuss how cell type-specific information can be used to understand the vulnerability of neuronal and non-neuronal cell types in these disorders.

## 2. Oxidative Stress in Health and Disease

### 2.1. Enzymes Involved in Neutralizing Reactive Oxygen Species

Reactive oxygen species (ROS) are a byproduct of many cellular functions and enzymatic reactions, and serve an important role in signaling cascades for all cell types [5,6]. ROS are produced continuously during normal mitochondrial respiration [7,8,9], in the form of superoxides and hydrogen peroxides, by the electron transport chain (ETC) [10]. Mitochondria have the ability to inactivate ROS internally using mitochondrially-localized superoxide dismutase 2 (SOD2); in the cytosol, copper ion scavenging is utilized to convert superoxides to hydrogen peroxide via superoxide dismutase 1 (SOD1) and the copper chaperone for superoxide dismutase (CCS) [11,12]. If copper and zinc are not available, often the cell can use iron as a cation in the scavenging process [13]. However, with aging, mitochondrial membranes show increased depolarization and permeability to toxic species, allowing ROS to more readily translocate from within mitochondria to the cytosol [14]. Glutathione peroxidases (GPx1-8) are responsible for the reduction of hydrogen peroxide into water, using glutathione as a substrate (reviewed in [15]), while catalase (CAT) breaks down hydrogen peroxide into oxygen and water via a two-step process involving its heme-containing moieties (reviewed in [16]). Peroxiredoxins are also involved in hydrogen peroxide signaling due to the highly sensitive nature of the cysteine in its active site (PRDX1-6) (reviewed in [17]). Other enzymes involved in general detoxification in oxidative states include the glutathione S-transferases (GST family) (reviewed in [18]), which conjugate the reduced form of glutathione to xenobiotic substrates for detoxification [19,20]. Additionally, NAD(P)H:quinone oxidoreductases (NQO1 and 2) are cytosolic proteins which remove quinones from toxins, stabilize p53, and play a role in vitamin E metabolism [21,22].

### 2.2. Cellular Effects of Reactive Oxygen Species Accumulation

Ultimately, the overload of the antioxidant defense system and/or an overabundance of DNA damage and protein oxidation leads to the induction of regulated cell death [23,24]. ROS target the cysteine residues on proteins with many possible outcomes, including targeting the autophagosome for degradation, the localization to different subcellular compartments, functional inactivation, or the disruption of protein-protein interactions [7]. Mitochondrial ribosomes are particularly sensitive to ROS-mediated damage and have been shown to decrease translation in response to increased ROS content [25]. Alterations in the proteome lead to an eventual responses from the autophagy system; these include increased autophagic/mitophagic flux, as well as increasedrelated mechanisms necessary to clear damaged endoplasmic reticular membranes and misfolded protein aggregates (reviewed in [26,27,28]). Increasing ROS can also cause double-stranded DNA breaks and other forms of DNA damage [29,30]. Accordingly, increased ROS accumulation has been reported in aging studies, particularly in the central nervous system (CNS) [31,32]. As aging is the primary risk factor in neurodegenerative diseases, substantial evidence points to oxidative stress as a significant contributor to the progression of cell death in age-related diseases such as PD and ALS (see below; reviewed in [33]).

### 2.3. The Transcriptional Regulation of Antioxidant Defense Enzymes

**Figure 1:**
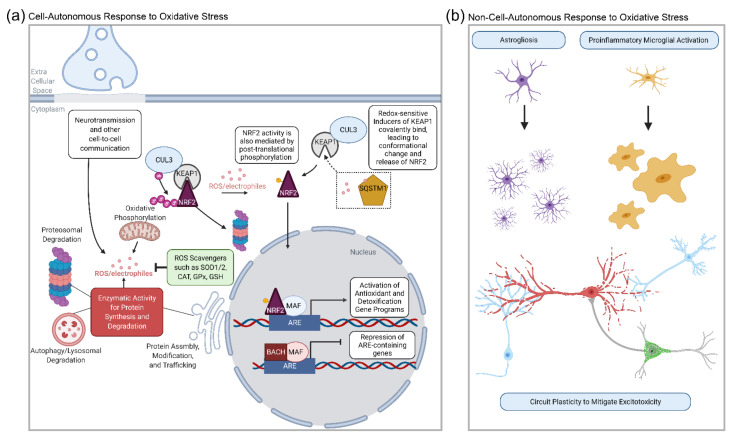
Schematic of canonical factors in cell-autonomous sources of oxidative stress and response are shown in (**a**). At basal state, transcription factor NRF2 is sequestered in the nucleus by the KEAP1 ubiquitination complex; here, CUL3 ligase activity leads to poly-ubiquitination of NRF2 for its targeted proteasomal degradation. Oxidative stress leads to a conformational change in KEAP1, and NRF2 is released to be phosphorylated and translocated into the nucleus to activate antioxidant response gene programs. Examples of non-cell-autonomous mediators of oxidative stress are shown in (**b**). Created with BioRender.com (accessed on 21 October 2021).

A number of proteostatic and transcriptional mechanisms are in place to handle ROS in basal states. Key cell-intrinsic factors of the oxidative stress response are shown in Figure 1a, with CNS cell types involved in the circuit-level mitigation of oxidative stress shown in Figure 1b. In this review, we will focus on transcription factors and their demonstrated roles in regulating transcriptional programs for antioxidant defense in both neuronal and non-neuronal cell types.

#### 2.3.1. The Identification of the Antioxidant Response Element and Its Binding Factors

Early studies identified a sequence of DNA required for transcriptional responses to xenobiotics, and other electrophiles, in hepatoma cell lines [34] and coined this sequence the “antioxidant response element (ARE)” [35]. The ARE was identified in transcriptionally active regions of the glutathione S-transferase and NAD (P) H:quinone reductase genes, and was later found to contain binding sites for the transcription factors AP-1 [36], jun-B, c-Fos [37], and NRF1/NRF2 [38], with NRF1/NRF2 being the primary drivers of transcription [38]. Since then, many studies have used a combination of luciferase reporter assays and deletion/overexpression strategies to determine the roles of the members of the NRF family in the ROS-mediated induction of gene expression.

NRF family members are encoded by the *NFE2*, *NFE2L1* (NRF1), *NFE2L2* (NRF2), and *NFE2L3* (NRF3) genes [38]. Of note, in this article, we will use given gene names when referring to genetic sequences or transcripts, to avoid confusing *Nfe2l1* with nuclear respiratory factor 1 *(Nrf1*). When referring to the transcription factors at the protein level, we will use the corresponding NRF designation. Of the three transcription factor family members, NRF2 is by far the most well-studied, particularly in terms of validated gene targets and regulation. Canonically, NRF2 is basally localized to the cytoplasm, sequestered by the protein Kelch-like ECH-associated protein 1 (KEAP1) in basal states. In the presence of ROS, NRF2 is released from KEAP1 and translocates to the nucleus, where it activates transcription in genes containing AREs [39,40,41]. NRF1 and NRF3 also bind to AREs but are relatively understudied [42,43]. Other transcription factors can activate genes with AREs, including EP300 and CREB [44,45], and the sMAF family of proteins (MAFa MAFf and MAFk MAFg), which can homodimerize or heterodimerize with other sMAF members, or the NRF family, to both activate and repress gene expression [46,47]. Repressors of NRFs include the BTB domain and CNC homologs 1 and 2 (BACH1 and BACH2), which can form heterodimers with members of the MAF and NRF families, thereby preventing the overactivation of antioxidant pathways [46].

#### 2.3.2. NRF Transcription Factors in The Brain

In terms of the NRF family member function in the CNS, NRF2 is the most well-characterized. NRF2 knockout mice [48] exhibited a number of abnormalities, including: age-related retinopathy [49]; higher LDL cholesterol levels and age-induced obesity [50]; reduced synaptic density and exacerbated age-related cognitive deficits [51,52,53]; reduced generation of neural stem/progenitor cells in the hippocampus [54] and subventricular zone [55]; an age-related decline in the expression of genes involved in autophagy [56]; and an increased activity in the forced-swim test with increased dopamine and serotonin levels [57]. Regarding the role of NRF2 in pathological states, knockout mice showed lower survival rates, more severe injury, and increased inflammation after intracerebral hemorrhage [58,59], neonatal hypoxia–ischemia [60], traumatic brain injury [61], and middle cerebral artery occlusion [62], highlighting the critical role of NRF2 in maintaining neuronal survival after oxidative insults.

Less is known about the roles of NRF1 and NRF3 in the brain. NRF1 is expressed early in development in the liver and brain, in addition to bone and other tissues ([63]; reviewed in [64]). Due to defects in lung development, as well as anemia, NRF1 knockout mice are embryonic-lethal [65]. Conditional deletion studies have shown that NRF1 is involved in lipid homeostasis in the adult liver, and that NRF1 is required for neuronal survival [66,67,68]. Conditional NRF1 deletion with the *Camk2aCre* line causes forebrain atrophy and neuron loss, accompanied by ubiquitin positivity and high-molecular weight ubiquitin–protein conjugates, as well as proteasome dysfunction. Interestingly, glutathione is not depleted in this line, but the gene expression for multiple components of the 19S complex and 20S core is reduced [69]. In another model, NRF1 deletion throughout the CNS, earlier in development and investigated using the *NestinCre* line, caused early mortality (within 3 weeks of birth), associated with increased oxidative stress in the spinal cord [70]. Interestingly, mice with a developmental deletion in the CNS showed similarities to Mafg:Mafk compound mutant mice [71,72], such as selective neurodegeneraton and signs of motor dysfunction supporting the interaction of NRF1 with MAF family members in the CNS [72]. Based on these findings, NRF1 has recently been touted as a key regulator of proteasome genes, with evidence for its activation and translocation by proteasome-related stress [73,74,75]. As mentioned above, very little is known about the roles of NRF3 in the brain; NRF3 knockout mice are viable and fertile, with few obvious abnormalities [76].

The most widely studied NRF targets include genes encoding NQO1, HO-1, GSTs, and PRDxs (reviewed in [77]), but NRF2 knockdown has also been associated with the reduced expression of SOD1, SOD2, CAT, and PRDx1 [78]. More recently, studies using chromatin immunoprecipitation (ChIP) assays have identified overlapping targets for the NRF family members for genes involved in RNA metabolism, development, and the unfolded protein response [79,80]. ChIP-seq in A539 non-small cell lung cancer cells identified a number of genes involved in focal adhesion pathways [81], while ChIP-seq for NRF2 in lymphoblastoid cells identified the retinoid receptor RXRA as a putative target [82], with emerging roles in the regulation of genes involved in autophagy and the unfolded protein response [83]. Interestingly, some NRF2 targets (*Gclc* and *Nqo1*) are shared by the histone variant H2AX, the phosphorylation of which mediates DNA repair, suggesting an overlap between NRF2 activity and DNA damage-related transcriptional responses in fibroblasts [84]. Altogether, these findings indicate that while NRF1, NRF2, and NRF3 may share consensus binding sequences in gene promoters, downstream consequences of their modulation may vary greatly by tissue and/or cell type.

#### 2.3.3. The Localization of the Transcriptional Regulators of NRF-Dependent Processes Using Mouse Brain Single-Cell Transcriptomics Data

Despite the profound effects of NRF2 knockdown on neuronal survival in different pathological contexts (above), multiple reports indicate that astrocytes and microglia express the highest levels of NRF2 in the brain [85,86,87,88,89,90]. One of the first studies to explore the roles of NRF2 in the cells of the nervous system was performed using cortical astrocytes from mice lacking NRF2 [91]. A number of NRF2-dependent genes were identified, including *Nqo1*. Since then, several studies have suggested the enrichment of NRF2 in astrocytes and other non-neuronal cells. In fact, NRF2 knockout mice exhibit astrogliosis and leukoencephalopathy at baseline [92]; increased astrogliosis in a closed head injury model [93]; increased neuroinflammation in multiple sclerosis [94], tauopathy, and amyloidopathy models [95]; and a hypersensitivity to pro-inflammatory stimuli [96]. The relative deficiency of NRF2 in adult neurons was demonstrated by Bell et al. [90]; interestingly, the authors found that the deficiency observed in adulthood was associated with the repression of promoter activity and that the NRF2 expression associated with an immature phenotype served to maintain the REDOX status while inhibiting the JNK and WNT pathway responses to oxidative stress. The same group also demonstrated that astrocyte-derived NRF2 enabled the production of glutathione for its release and subsequent uptake by neighboring neurons [97], highlighting the non-cell-autonomous role of NRF2 in neuronal survival. Accordingly, the activation of NRF2 pathways in astrocytes has been suggested as a therapeutic approach for neuroprotection [98]. The overexpression of NRF2 in astrocytes is beneficial in a mouse model of Alexander disease, which is associated with mutations in glial fibrillary acidic proteins (GFAP) and the overactivation of astrocytes [99]. The activation of NRF2 pathways in models of PD and ALS will be discussed in more detail below. Less is known about the cellular location of NRF1 and NRF3 in the CNS.

**Figure 2:**
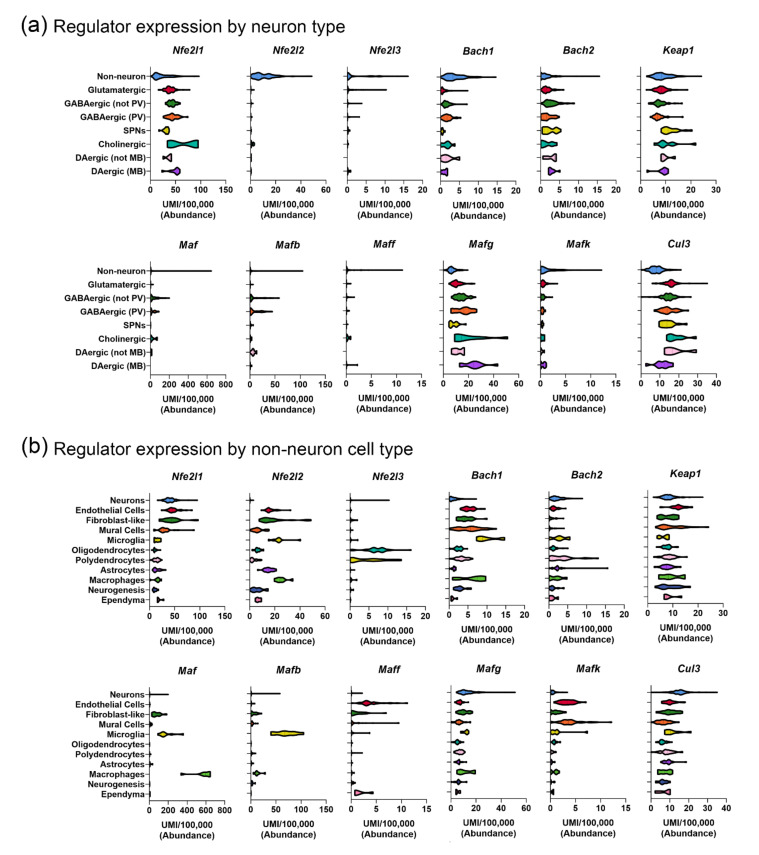
Transcript abundance for transcriptional regulators was assessed in neuronal (**a**) and non-neuronal (**b**) populations using the Dropviz.org dataset [4]. All values are expressed as UMI (unique molecular identifier)/100,000 in each cell subcluster. Statistically significant differences among groups for (**a**,**b**) are included in Supplementary Table S1.

To explore the cell type-specific expression profiles of NRF family members and their related transcriptional regulators, we used a publicly available database of single-cell transcriptomic data ([4]; Figure 2), sorted by the neuron type (Figure 2a) and the non-neuronal cell type (Figure 2b). It is important to note that this database reflects mRNA expression patterns at baseline in a wildtype adult mouse; it is possible that conditions associated with oxidative stress could stimulate different patterns of gene expressions across these cell types. As suggested from the *Nfe2l2* distribution studies cited above, *Nfe2l2* mRNA expression is most abundant in macrophages, microglia, astrocytes, and fibroblast-like cells. *Nfe2l1* (NRF1), however, is more highly enriched in neurons, and *Nfe2l3* (NRF3) is particularly enriched in oligodendrocytes and polydendrocytes (myelinating cells). These distribution patterns explain the different physical and behavioral phenotypes in the respective knockout mouse lines, with NRF1 knockout mice exhibiting neuronal loss and NRF3 knockout mice showing very few abnormalities. Based on *Nfe2l3* distribution data, it would be predicted that an NRF3 knockout may cause myelination deficits, although this has yet to be tested.

Information regarding the cell type-specific distribution of other transcriptional regulators can be used to predict which combinations of factors may be jointly involved in transcriptional regulation. For example, it is interesting to note that while *Keap1* expression is relatively ubiquitous, *Mafg* is enriched in neurons and *Mafb* is enriched in microglia. Based on these observations, the interactions between *Nfe2l1* and *Mafg* or between *Nfe2l2* and *Mafb* could be explored further in these cell types. The observation of ubiquitous *Keap1* expression with glial-enriched *Nfe2l2* expression suggests that KEAP1 may interact with other proteins in neuronal cells; in fact, KEAP1 has been found to interact with sequestosome 1 (SQSTM1/P62) [100] and RNA binding proteins (RBM45) [101]. Mutations of SQSTM1 have been associated with the risk for frontotemporal dementia (FTD), with mutations leading to disrupted interactions between KEAP1 and SQSTM1 [100,102]. These studies go on to hypothesize that the disrupted KEAP1 interactions lead to the repression of NRF2 signaling; however, considering the low expression of NRF2 in neurons, the potential relevance of this interaction should be explored in glial cell types.

**Figure 3:**
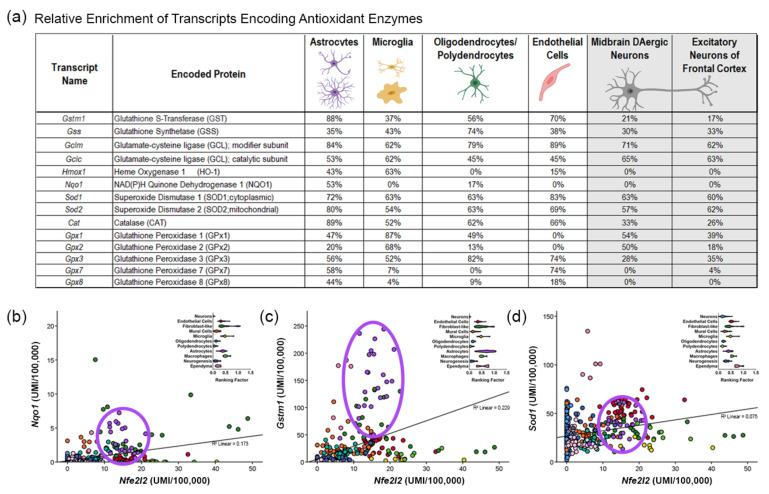
Dropviz.org database [4] was used to demonstrate the cell type-specific differences in downstream transcriptional targets involved in antioxidant defense (**a**); percentages shown in table represent transcript abundance with respect to the highest expressing cell subcluster and relationships between transcript abundance of transcriptional regulators (shown in Figure 2). The level of antioxidant response gene expression was explored in (**b**–**d**). Values are expressed as UMI/100,000 in graphs and as “ranking factor” for cell type-specific patterns of expression in figure insets. Ranking factors were generated by calculating the distance from the origin for each cell type using the equation [x2 + y2]^0.5^, where (x,y) represents UMI/100,000 values normalized to the highest expressing subcluster.

These data can also be mined to investigate the cell type-specific distributions of antioxidant enzyme gene expression (Figure 3a), the relationships between transcriptional regulators and their putative downstream targets, or to identify cell-types of interest to explore relevant protein-protein interactions (Figure 3b,d). In Figure 3a, we show the distribution of mRNA expression for different antioxidant enzymes in non-neuronal cells and neuron types, with respect to the highest expressing cell subcluster from Dropviz.org [4]. Considering *Nfe2l2* enrichment in non-neuronal populations, we then explored the relationship between *Nfe2l2* and two of its putative targets, *Gstm1* and *Nqo1*. As indicated by [91], *Nfe2l2*, *Gstm1*, and *Nqo1* are all expressed by astrocytes (purple circles, Figure 3c,d), but are also co-expressed in other non-neuronal populations. However, there was not a strong relationship between *Nfe2l2* and *Sod1* (Figure 3d) or *Sod2* expression (not shown), suggesting that other factors could be involved in *Sod1* and *Sod2* regulation.

Regarding the transcriptional regulation of SOD1 and SOD2, there is some evidence for the regulation of SOD2 by CREB and the transcriptional coactivator, peroxisome proliferator-activated receptor coactivator 1-α (PGC-1α) [103]. Our laboratory has shown that mice lacking PGC-1α exhibit decreased *Sod2* expression in various regions of the brain [104]. Interestingly, SOD2 knockout mice show vacuolization throughout the brain [105,106,107], similar to PGC-1α knockout mice [104,108,109]. However, the molecular contributors to PGC-1α knockout-related encephalopathy likely also involve the deficiency of a number of other PGC-1α putative targets, such as the neurofilament heavy chain (NEFH) [110,111,112]. Overall, the lack of cell type-specific co-localization and/or strong correlations between many of these transcriptional regulators and their presumed targets suggest that a number of other transcriptional targets may be revealed if cell type-specific strategies (translating ribosome affinity purification or nuc-seq) are used to profile NRF knockout systems.

## 3. Oxidative Stress and the Transcriptional Regulation of Antioxidant Defense Enzymes in Disease States

There is abundant evidence for oxidative stress in neurodegenerative disorders (reviewed in [1,2,3]). Based on our observations of the cell type-specific distribution of transcription factors involved in the regulation of antioxidant responses (Figure 2 and Figure 3), here, we discuss the cell-autonomous and non-cell-autonomous contributors to neuronal oxidative stress in two disorders, Parkinson’s disease (PD) and amyotrophic lateral sclerosis (ALS). We also present strategies which have been attempted in models of both disorders for promoting normal and/or therapeutically relevant transcriptional activity of NRF family members, including overexpression strategies and the manipulation of NRF-interacting proteins.

### 3.1. Parkinson’s Disease and Oxidative Stress

PD is a severe movement disorder characterized by the loss of dopaminergic (DAergic) neurons of the substantia nigra pars compacta (SNc) as well as the accumulation of proteinaceous aggregates containing misfolded α-synuclein (α-syn) proteins. Patients are most often diagnosed at the onset of motor symptoms, at which time they have already sustained a loss of approximately 80% of DAergic nigrostriatal terminals, with the predicted loss of over half of the SNc DAergic neurons [113,114]; reviewed in [115].

Substantial clinical evidence, from both ante- and post-mortem studies, implicates cellular stress caused by reactive species in PD pathology (reviewed in [116]). Post-mortem studies in the PD brain have reported increased lipid peroxidation [117], hyperoxide accumulation [118], and protein carbonyls [119], which are all indicators of oxidative stress. It is thought that a dysregulated maintenance of reactive species is an early feature of the disease. Dexter et al. [118] reported that a decreased ratio of reduced glutathione to its oxidized counterpart (GSH:GSSG) preceded cell death, and further posited that GSH levels are predictive of DAergic vulnerability. The surviving/resilient DAergic neurons also showed an increased expression of GPx1 [120], as well as an increase in the translocation of NRF2 to the nucleus [121]. There is also evidence for oxidative damage to both nuclear and mitochondrial DNA and RNA, especially in SNc neurons [122].

Further exacerbation of oxidative stress in PD could be attributed to an increased cellular accumulation of iron (Fe) in the SNc [123,124]. Fe and other transition metals act as pivotal electron shuttles that are necessary to catalyze multiple cellular processes. However, excessive cellular Fe disrupts REDOX homeostasis, stabilizing reactive species that normally exist only transiently with normal ROS signalling (reviewed in [125]). Fe dyshomeostasis is thought to be more deleterious in DAergic neurons, as multiple reactions in the synthesis and metabolism of dopamine are dependent on Fe, and abnormalities in these pathways lead to the generation and accumulation of toxic species via Fenton reactions (reviewed in [126]). While increased Fe accumulation has been shown in the caudate putamen, SNc, and other basal ganglia structures with age [127,128], in PD, Fe accumulation in these regions is significantly higher than what is observed in healthy, age-matched controls. In one of the first studies reporting this pathological observation, PD post-mortem brains showed over a 2-fold increase in the total Fe amount, and over a 4-fold increase of its ferric, oxidized form [123]. Follow-up studies replicated this finding, showing a more modest, but still significant, increase in Fe when compared to age-matched controls [129]. Increased Fe accumulation, specific to the SNc, has been consistently substantiated with a variety of brain imaging techniques in both sporadic and familial forms of PD, and it correlates with disease severity [130,131,132,133].

Interestingly, in *PARKIN*-type early-onset juvenile PD, there is more intense SNc Fe staining [134]. However, while Fe is present in multiple brain cell types, Fe accumulation is not found in the cortex, hippocampus, putamen, or globus pallidus in PD brains [124] and is significantly reduced in the temporal cortex [135]. This could be due to the enrichment of neuromelanin in the dopaminergic neurons of the SNc; neuromelanin assists with the storage of Fe in these neurons, while other regions of the brain utilize the ferritin protein complexes, H-ferritin and L-ferritin [136].

α-syn-containing Lewy bodies (LBs), a pathological hallmark of PD and LB-associated disorders such as PD with dementia (PDD) and dementia with Lewy bodies (DLB) (reviewed in [137]), could be a consequence of REDOX dysregulation. LBs form in cell bodies and the projections of the DAergic neurons of the SNc, but also in other neuronal populations throughout the brain [138]. However, LB pathology is not associated with cell death in most other regions and cell types outside of the SNc [139]. While it is not well-understood as to what makes specific neuronal populations susceptible to developing LB pathology, there is a compelling link between α-syn, oxidative stress, and Fe dyshomeostasis. First, high levels of Fe are found within LB aggregates [140], and Fe is able to mediate alterations in α-syn structure [141,142,143,144,145]. An intriguing explanation for this link is that α-syn plays a major role in regulating Fe homeostasis. It has been proposed that α-syn can act as a ferrireductase, reducing oxidized Fe (Fe^3+^) to its bioavailable ferrous (Fe^2+^) form, which could lead to oxidative stress through Fenton chemistry [146,147]. Alternatively, it is possible that the aggregation of α-syn would inhibit its reductive ability, leading to an increased accumulation of oxidized Fe [147]. Interestingly, computational analyses of *SNCA* transcript secondary structure suggest that human *SNCA*, but not *SNCB* or *SNCG*, may have an RNA stem loop within its 5′ UTF that is structurally similar to the iron response element (IRE) seen in ferritins [148]. While the functional relevance of this computational modeling is not clear, Olivares et al. [149] provided evidence for α-syn’s iron-dependent posttranscriptional regulatory function in SH-SY5Y cell culture, suggesting the possibility of the role of α-syn in regulating Fe levels at both the RNA and protein levels [149]. Additionally, it is important to recognize that other pathological processes, such as the accumulation of beta-amyloids, could contribute to PD risk [150] (reviewed in [151,152]), although amyloid deposition is more common in PDD and DLB than in PD without dementia [150].

While the progressive motor dysfunction observed in PD is attributed to the selective degeneration of DAergic neurons in the SNc, the causal mechanisms of DAergic vulnerability and eventual cell death in PD are still largely speculative. However, a collation of evidence generated in post-mortem tissue analyses, neurotoxin studies (rotenone, paraquat, and MPTP), and the identification of loss-of-function mutations in genes linked to mitochondrial clearance (e.g., *PARKIN* and *PINK1*) and function (e.g., *DJ-1*) implicate a mitochondrial-driven imbalance in REDOX systems. Mechanistic links between *LRRK2* mutations, the most common cause of familial, autosomal-dominant forms of PD (reviewed in [153]), as well as mitochondrial dysfunction are not as clear as for other PD risk genes. The majority of *LRRK2* mutations are associated with increased kinase activity [154,155,156], and the cellular consequences of increased *LRRK2* activity include impairments in a number of neuronal processes, such as vesicular trafficking, actin dynamics, calcium buffering, and proteostasis (reviewed in [157,158]. The most compelling link between *LRRK2* mutations and mitochondrial function is *LRRK2*′s potential roles in mitophagy (reviewed in [159]). In fact, *LRRK2* inhibitors have been reported to rescue impairments in mitophagy [160] and reverse mtDNA damage observed in the immune cells of PD patients [161,162], indicating a role for mutation-induced *LRRK2* activation in mitochondrial dysfunction and suggesting that mitochondrial deficits could be ameliorated by treatments using *LRRK2* inhibitors, some of which are currently in clinical trials [163].

DA metabolism [164], maintenance of DAergic tone through cell-autonomous pacemaking firing [165], and continuous neurotransmissions along extensively arborized axonal projections to the caudate putamen [166,167,168,169] all generate a huge bioenergetic demand in this population. However, DAergic neurons must meet this demand with a relatively low mitochondrial density [170], creating a delicate balance between constant energetic needs and the oxidative stress load. In fact, there is strong evidence for higher basal oxidative stress in non-disease SNc compared to other regions of the brain [1,171,172,173]. This may explain the selective vulnerability of the SNc in PD, as negative consequences of accumulating oxidative stress increase in normal aging. To date, the factor associated with the greatest risk for developing PD is age [172,174].

Further supporting oxidative stress as a contributive factor in PD pathogenesis are the mutations observed in *PARK7*, the gene that encodes DJ-1, which are reported to cause an autosomal-recessive, early-onset form of PD through the loss of DJ-1 expression or activity [175]. Over 25 PD-linked *PARK7* mutations have been identified (NIH; https://medlineplus.gov/genetics/gene/park7/#conditions (accessed on 25 February 2021)). Notably, decreased levels of DJ-1 have also been observed in sporadic PD, post-mortem SNc, and the cortex [176]. While diverse cellular functions for DJ-1 have been proposed, data from a wide span of model systems have shown increased oxidative stress and diminished cellular viability when DJ-1 expression or activity is impeded, supporting its role as a critical sensor for cellular oxidative stress (reviewed in [177]). Under oxidizing conditions, DJ-1 converts to its more acidic isoform, with sulfinic acid modifying cysteine residue [178], shifting its isoelectric point (pI) from around 6.2 to 5.8. Levels of the pI 5.8 isoform are basally low, but increase during exposure to oxidative stress, with a reduction back to basal levels when the stress is removed. In the human post-mortem frontal cortex, Bandopadhyay et al. [179] found six different pI isoforms of DJ-1 [179]. The most basic, isoform 6.6, appeared diminished/missing in the R98Q PD model. There was also a congruent increase in more acidic isoform expressions following oxidative stress. The C106A mutant form of DJ-1 showed lower cell viability and increased cellular sensitivity to the oxidative stressor MPP+, indicating that this function is protective, at least in vitro [179]. It has been suggested that, following the modification of C106, DJ-1 localizes to the outer membrane of mitochondria [175,178,180,181]. This localization is further supported in studies that show DJ-1-dependent mitochondrial dysfunction. For example, Irrcher et al. [182] investigated DJ-1 deficiency in multiple models of PD (cells, mice, and human-derived lymphoblasts from PD patients), all of which showed increased susceptibility to oxidative stress and subsequent neuronal death [182].

#### 3.1.1. PD and NRF2

NRF2 was first implicated in PD when changes in its expression were observed in the PD post-mortem brain, with increased nuclear localization of the NRF2 protein in DAergic neurons when compared to controls [121], which was replicated in [183,184]. It is thought that DAergic neurons of the SNc upregulate the NRF2 regulon in response to mounting oxidative stress; however, this response is ultimately not sufficient to protect DAergic neurons from degeneration in PD [184]. Interestingly, a mutation in *NFE2L2,* that is thought to increase its transcriptional activity, was shown to decrease the risk of PD and delay its onset, suggesting that levels of NRF2 activity may be inversely correlated with PD susceptibility [185].

A study with fibroblasts derived from patients with *LRRK2* mutations with or without PD found an increase in NRF2 expression in patients with PD, accompanied by an increase in mitochondrial mass [186]. Urate, an activator of NRF2, was elevated in *LRRK2* mutation carriers resistant to PD [187]; the authors of that study suggested that peripheral urate levels could prevent the emergence of PD by activating NRF2-dependent pathways. Protective effects of lovastatin in G2019S *LRRK2*-expressing neurons could be mediated by an increase in NRF2 phosphorylation [188]; the phosphorylation of NRF2′s Neh2 domain by enzymes such as PKC can activate it by facilitating the dissociation from KEAP1, while the phosphorylation of the Neh6 domain by glycogen synthase kinase 3α or 3β can promote its degradation (reviewed in [189]). In cortical neurons that overexpress G2019S *LRRK2*, NRF2 overexpression was shown to induce the expression of ARE-containing genes, increase synuclein degradation, cause the sequestration of mutant *LRRK2* in neurons, and can attenuate cell death [190].

The potential neuroprotective role of NRF2 overexpression in the face of PD-related oxidative stress has been supported by a large body of research using both cell and in vivo rodent model systems (reviewed in [77,191]). Chen et al. [192] reported decreased NRF2 activity and ARE-containing gene expression in the MPTP mouse model, with the loss of *Nef2l2* expression exacerbating the pathology of this model [192]. Additionally, *Nfe2l2* knockout mice are shown to be more vulnerable to 6-OHDA treatment [193]. In a more recent study, the pharmacologic activation of NRF2 was neuroprotective in mice with a viral-mediated expression of human α-syn; this effect was absent in NRF2 knockout mice, which showed worsened phenotypes of α-syn toxicity [194]. However, there is still some ambiguity concerning the neuroprotective mechanisms of increased NRF2 activity and which cell types are most dependent on NRF2-mediated pathways [90,195,196,197].

#### 3.1.2. The Role of Non-Neuronal Cells in Responses to Oxidative Stress

While PD research has been largely focused on cell-intrinsic contributors to DAergic vulnerability, surrounding glia are important potential mediators of neuronal oxidative stress. Accumulating evidence suggests that glial-mediated microenvironments and cross-talk with neurons is disrupted in numerous neurological disorders, and emerging literature supports the link between glial dysfunction and PD etiology (reviewed in [198,199,200,201,202]). Previous studies and transcriptional analyses from [4] (Figure 2) demonstrated that *Nfe2l2* expression was enriched in astrocytes and microglia [4,89], and several studies have provided evidence supporting a role for the glial-expressed NRF2 in neuroprotection. It has been demonstrated that NRF2 regulates different genes in different cell types, specifically when comparing astrocytes and neurons [203], supporting the notion that different cell populations could contribute to the NRF2-mediated mitigation of oxidative stress in the brain.

While upon their discovery, astrocytes were initially thought of as structural supports, as well as having essential roles in development, memory consolidation, neuroimmune function, and the buffering of extracellular glutamate have been heavily substantiated (reviewed in [204]). In a recent review, it was proposed that boosting NRF2/WNT transcriptional pathways in astrocytes, and enhancing astrocyte-neuron crosstalk pathways, may lead to DAergic neuroprotection [205]; the overexpression of *Nfe2l2* was found to rescue DAergic cell loss in both 6-OHDA [193] and MPTP models of PD [192]. A recent study involving the activation of NRF2 in a *LRRK2* mutant drosophila model further supports the potential neuroprotective utility of NRF2 activation as well as a critical role for astrocytes in the NRF2 response. The forced expression of wild-type or G2019S *LRRK2* in DAergic neurons caused motor impairment that could be rescued by treatment with the herbal extract *Gastrodia elata* (GE); this rescue was associated with the activation of the ARE regulon in non-neuronal cells [206].

Importantly, this paper demonstrated that the deletion of the drosophila homolog of *Nfe2l2*, *cnc*, in DAergic neurons had no effect on the GE neuroprotective efficacy, while the knockdown of *cnc* in astrocyte-like glia prevented neuroprotection. These findings indicate that activation of the NRF2 pathway in glia can be neuroprotective in the context of neuronally-expressed *LRRK2* mutations. In contrast to these observations, Ramsey et al. [184] did not observe increased nuclear-NRF2 translocation in the surrounding glial populations of SNc, and posited that the glial activation of NRF2 may not be necessary for DAergic neuroprotection [184]. Additionally, the NRF2 activation in cell culture models of PD (MPP+, paraquat, and 6-OHDA) was neuroprotective in the absence of astrocytes and other glial populations (reviewed in [77]). It is possible that the involvement of astrocytes in DAergic vulnerability differs with the type of pathological stimulus. Future studies should assess NRF2 activity in models of cell death mediated by chronic stressors, like α-synucleinopathy, compared to the more acute stressors used in toxin models.

Consistent with an enrichment of *Nfe2l2* mRNA in astrocytes, the expression of NRF2-ARE regulon members such as *Nqo1* are basally expressed more highly in astrocytes than in any other type of cell in the brain ([4]; Figure 3a,b). In humans, NQO1 expression in steady-state DAergic neurons and in the SNc of the healthy brain is minimal; however, it is markedly increased in both astrocytes and neurons in PD post-mortem brains [207]. HO-1 and metallothinein proteins MT1 and MT2 (encoded by ARE-containing genes) are vital for Fe metabolism; transcripts encoding these proteins are all highly enriched in astrocytes [4]. Notably, HO-1 levels are reduced in the PD post-mortem brain [183]. The basal enrichment of these stress-responsive NRF2 targets suggests that astrocytes likely play a principal role in maintaining homeostatic levels of oxidative species, as well as cellular Fe and other transition metals across the brain.

Beyond the enrichment of stress-responsive gene programs, evidence of astrocytic dysfunction has been observed both in the clinic and in model systems of PD. Interestingly, while there is an increase in astrocytes in post-mortem tissue from PD patients when compared to tissue from controls [208], there is an inverse relationship between GFAP-positive astrocytes and DAergic cell loss in PD [120]. Astrocytes derived from induced pluripotent stem cells from *LRRK2* mutation carriers are impaired in their ability to provide neurotrophic support for DAergic neurons in culture [209], as well as showing reduced complexity, mitochondrial density, and ATP production (33785762), and an abnormal accumulation of synuclein and dysfunctional autophagy, causing the degeneration of normal DAergic neurons [210]. *LRRK2* G2019S astrocytes show an impaired internalization and clearance of α-synuclein, potentially through the loss-of-function of annexin A2 [211], the depletion of calcium in the endoplasmic reticulum, and mitochondrial dysfunction [212]. In vivo, *LRRK2* mutations cause a loss of primary cilia in cholinergic neurons and astrocytes [213] and a decrease in astrocyte numbers in the striatum [214]. Interestingly, *LRRK2* inhibition is capable of rescuing inflammatory and lysosomal defects in astrocytes derived from mice expressing the D409V mutant of GBA [215].

In addition to scavenging glutamate, certain populations of astrocytes are involved in DA clearance and metabolism. When astrocytes are treated with DA in culture, they are seen to have a biphasic, metabolically complex response [216]. In fact, midbrain astrocytes, and to a lesser extent, striatal astrocytes, are particularly enriched with the aldehyde dehydrogenases *Aldh1l1* and *Aldh1a1* [4]. These are thought to be involved in DA catabolism, and have been implicated in PD [217,218,219]. Midbrain astrocytes are also relatively enriched with *Maob*, which encodes monamine oxidase b (MAOB) [4]. The MAOB breakdown of DA leads to the generation of superoxides, which, given their high basal expression of NRF2 regulon factors, enables astrocytes to be more equipped to handle them than their neuronal counterparts. However, the role of astrocytes in this context is understudied.

Recent studies further supported that astrocytes from different regions of the brain have different properties [220] and that these properties change with age [221]; however, differences between astrocytes in differentially vulnerable regions of the midbrain (SNc vs. VTA) are not known. It is now widely accepted that DAergic neurons of the VTA co-release glutamate and DA to the nucleus accumbens (Nac) [222]. Interestingly, astrocytes upregulate *Nfe2l2* expression following glutamate exposure/treatment [223]. Perhaps Daergic neurons of the VTA are more resilient to damage from oxidative stress due to the surrounding astrocytes being primed to mitigate glutamatergic toxicity, and subsequently, damage from reactive species. While this is merely speculative, clarity may be gained from an in-depth characterization of regional differences between astrocyte populations.

Microglia have also been proposed to play a major role in REDOX homeostasis in the midbrain. In the mouse brain, the microglial density in the SNc is significantly higher than other regions [224,225], and these populations are more reactive to PD-specific stress than microglia in other brain regions [226]. Interestingly, *Nef2l2* is highly enriched in microglia ([4,89]; Figure 2b), and there is substantial evidence that NRF2 activity in microglia promotes their inactivation. In a mouse model of traumatic brain injury, the siRNA-knockdown of *Nef2l2* in microglia caused an increase in the expression of the pro-inflammatory cytokines TNFα and IL-6, which was reversed in microglia overexpressing *Nef2l2* [5]. This parallels the increased microglial activation observed in NRF2 knockout mice, which also showed DAergic neuron loss [227]. Additionally, midbrain microglia have the highest enrichment in *Flt1* (L-Ferritin) [4]. This confers with iron storage capabilities, pointing to a reliance of DAergic neurons in the SNc on the surrounding microglia for Fe homeostasis and REDOX balance. These data suggest that microglia in the SNc microenvironment play a major role in mitigating oxidative stress in this region at a steady state.

Despite the enrichment of *Nef2l2*, midbrain microglia also have the highest transcript expression of proinflammatory factors, such as *Tnf* [4], which could contribute to the high basal oxidative stress in the region and the subsequent vulnerability to neuroinflammation. In PD patients, microgliosis has been observed both in the post-mortem SNc and in PET imaging studies [228,229]. It is thought that microgliosis occurs early in disease progression and is sustained over its course [229]. This observed increased in microglial occupation could also explain a recent report of increased BACH1 expression in the PD post-mortem midbrain [230]. As we show in Figure 2b, microglia are enriched with *Bach1*, a transcriptional repressor of ARE-containing genes [4], so they would be a likely contributor. This study goes on to show that BACH1 KO animals are more resilient to MPTP treatments, that and BACH1 inhibitors in wild-type animals attenuate MPTP-mediated toxicity [230]. These data suggest that the knockdown of BACH1 could be a viable strategy for promoting the neuroprotection of DAergic neurons in the context of PD.

Notably, inflammation is observed in regions affected by α-syn inclusions [231,232,233,234,235]. Microglia are thought to be integral in the clearance of excessive α-syn, and their spatial and temporal tracking with α-syn pathology supports the hypothesis that microglia play a role in the spread of pathological α-syn species via micosomes/exosomes [236,237]. A recent study demonstrated that activation of the glucagon-like peptide-1 receptor conferred neuroprotection in synucleinapthy models by acting on microglia to block the conversion of astrocytes to their toxic state, implicating a role for microglial-astrocyte crosstalk in synuclein models [238].

While research regarding the potential roles for astrocytes and microglia in PD has increased in recent years, few studies have explored the roles for other glial species such as oligodendrocytes and polydendrocytes, possibly due to reports of the relatively low myelination of DAergic neurons, which project to the caudate putamen [239,240]. However, the oligodendroglial enrichment for transcripts of the PD-causative genes, such as *Pink1* (*PARK6*) [4], point to their possible contribution to PD pathology. In fact, in animal models of multiple systems atrophy, the selective overexpression of α-syn in oligodendrocytes, using the myelin basic protein or proteolipid protein promoters, causes DAergic cell loss in mice [241,242]. In terms of REDOX regulators, oligodendrocytes are highly enriched in *Gss* (Glutathione) and *Fth1* (H-ferritin) [4], both of which contain AREs. Additionally, oligodendrocytes have the highest Fe content of any cell type in the brain; they are the major source of CNS iron storage [243]. Considering the enrichment of *Nfe2l3* in oligodendrocytes in mice (Figure 2b), future studies should explore the roles for this factor in response to oxidative stress or synucleinopathy.

**Figure 4:**
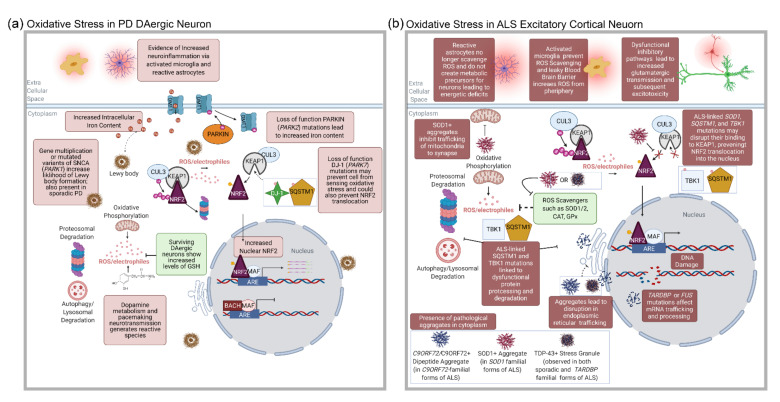
Schematics show how the cellular REDOX factors discussed in this review could be affected by, or contribute to, disease-specific stressors in the context of midbrain DAergic neurons in PD (**a**) and excitatory cortical neurons in ALS (**b**). Considerations include observations from clinical data, post-mortem tissue studies, and putative mechanisms derived from model systems. Created with BioRender.com (accessed on 25 October 2021).

Altogether, the studies referenced above, with the cell type-specific contextualization of disease-mitigating factors we have generated here suggest that, while DAergic neurons are most noticably affected in PD, it is probable that the dysfunction in multiple cell types could instigate PD pathogenesis (summarized in Figure 4a). Additionally, it underscores that cell type-specific expression profiles need to be referenced when generating and interpreting model systems, as well as in the process of therapeutic target engagement.

### 3.2. Amyotrophic Lateral Sclerosis and Oxidative Stress

ALS, also known as Lou Gehrig’s Disease, is a severe, degenerative movement disorder that presents as a loss of control of the extremities [244]. More rare symptoms include a loss of control of the musculature around the throat and vocal cords, rendering patients incapable of communicating (Bulbar ALS) [245]. Classic and bulbar forms of ALS have different prognoses, with the progression of the disease being highly variable based on patient lifestyle, diet, and other genetic risk factors [246]. On average, however, both subtypes have a life expectancy of 1–2 years post-diagnosis, with the eventual loss of diaphragm control and subsequent death [245]. Inheritable forms of ALS account for 10% of cases; the remaining 90% are sporadic in nature, with the emergence of de novo mutations, or no identified genetic component [247]. The most common causal ALS mutations are found in *SOD1* [248,249] and the C9 open reading frame 72 (*C9ORF72*; repeat expansion) [250].

The first indication of an involvement of oxidative stress in the pathobiology of ALS came from the discovery of the familial mutations in the *SOD1* gene [251]. Over 130 mutations have been identified in *SOD1* [252]. The primary role of *SOD1* is the scavenging of superoxides generated by oxidative phosphorylation [253,254]. *SOD1* mutations can be either familial or sporadic, leading to the generation of dominant-negative forms of *SOD1*, changes in its 3-D conformation, and abnormal aggregation and function [255]. Multiple studies have shown that *SOD1*-positive aggregates in the cytosol are associated with Tar DNA binding protein 43 (TDP-43, encoded by *TARDBP*) [29,256,257], reviewed in [257]. *SOD1*-positive aggregates can inhibit the trafficking of mitochondrial and synaptic proteins, leading to a decrease in ATP availability at the synapse [258]. *SOD1*-positive aggregates can also sequester BCL-2, leading to mitochondrial-mediated apoptosis and an increased autophagic flux [10,259]. Additional dysfunctions can involve the abnormal sequestration of copper (Cu), manganese (Mn), and zinc (Zn) ions [260], as well as the impairment of other proteins involved in Cu-mediated ROS scavenging including CCS, COX17, and ATX1 [261] (summarized in Figure 4b).

The most common pathological feature in the post-mortem brain tissue of patients with ALS is the intracellular and extracellular accumulation of protein aggregates [2,262], often containing TDP-43. Interestingly, TDP-43-containing aggregates are observed in the post-mortem tissue of patients with ALS, regardless of the *TARDBP* mutation status [263]. Other proteins that are shown to aggregate in ALS are optenurin (OPTN) and fused in sarcoma (FUS). A comparison of *SOD1*-mutation-carrying patients to those with mutations in *FUS* and *TARDBP* show similar, mutation-dependent progression, with some patients showing a significantly earlier onset [254].

TDP-43 is a DNA/RNA-binding protein responsible for transporting RNA out of the nucleus [264]. In pathological states, TDP-43 aggregates in the cytoplasm in protein- and RNA-containing stress granules [33]. TDP-43 aggregation has been shown to cause protein trafficking problems [2], leading to changes in the mitophagy and autophagic flux, an increase in damaged mitochondria, and the lower production of mitochondrial proteins by the mitochondrial ribosomes [265]. The gene and protein expressions of components of the electron transport chain is reduced, negatively impacting oxidative flux and mitochondrial capacity [266]. Recent studies also indicate that oxidative stress, such as an increase in hydrogen peroxide, can induce the mislocalization of TDP-43, and that this exclusion from the nucleus is dependent on poly(ADP-ribose) polymerase 1 (PARP-1) activity, a known ARE binding factor [267].

Compared to *TARDBP*-associated ALS mutations, mutations in *FUS* are more often associated with juvenile cases, with the predominant involvement of lower motor neuron degeneration [268]. Interestingly, FUS mutations do not involve TDP-43 aggregation (reviewed in [256]). However, similar to TDP-43, FUS associates with RNA/protein-containing stress granules [269] and nuclear paraspeckles, which mediate RNA splicing and RNA trafficking [270], which is especially prone to aggregation [271]. Similar to what is observed in TDP-43-related ALS, FUS aggregation is associated with an impairment in mitochondrial and endoplasmic reticulum function that compounds the effects of oxidative stress [272]. FUS-associated pathology also involves an enhanced sensitivity of AMPA receptors with increased excitotoxicity [273].

Due to the vulnerability of the *C9ORF72* locus to slippage during DNA replication, ALS-causative *C9ORF72* mutations arise from the repeat expansion of the hexanucleotide repeat CCCGGG, with toxicity observed at 30+ repeats [274]. Toxic repeat expansions lead to RNA aggregation in the nucleus and RAN-translation in the cytoplasm, generating dipeptide strands that aggregate and lead to a sequestration of proteasomal and chaperone proteins [275,276]. Much like the previously-discussed mutations, these aggregates inhibit protein-trafficking, disrupt lysosomal and mitochondrial functions, and increase the likelihood of oxidative stress-induced cell damage and death [277].

#### 3.2.1. The Neuronal Cell Types Affected in ALS

ALS is the most common motor neuron disease, with robust cell loss occurring in the lower and upper motor neuron populations of the spinal cord and motor cortex, respectively [278,279,280]. While lower motor neuron dysfunction and loss gives rise to muscle atrophy and motor impairment, cortical cell loss can contribute not only to a reduction in the excitatory drive to lower motor neurons, but also to the development of frontotemporal dementia (FTD) in approximately 40% of ALS patients [262]. FTD symptoms also include personality changes such as aggression, irritability, and impaired decision-making skills that are attributed to atrophy and cell death in the frontal cortex [277]. Recent studies have shown that the von Economo neuron subtype is the most affected in the frontal and temporal lobes of FTD and ALS/FTD patients. These are pyramidal neurons, with long axons and a high firing rate, are involved in communications across the anterior cingulate cortex and are found only in higher order social animals [281]. This characteristic complexity of the axonal structures and their rapid firing rate, which require high metabolic output, are thought to make these neurons particularly vulnerable to to ALS-related aggregates [262,277]; however, the mechanisms which contribute to their dysfunction and death are not as well-characterized as the processes that impact cholinergic motor neuron populations [39].

Dysfunctional cortical and spinal cord interneurons have also been implicated in ALS [231,232,233,282]. Multiple TDP-43-overexpressing mouse models have a loss of parvalbumin (PV)-expressing interneurons (PV-INs) and a reduction in the function of somatostatin (SST)-expressing interneurons [33,272,283,284,285,286], with interneuron impairment preceding the loss of motor neurons [283,284]. With a high intrinsic firing rate powered by high mitochondrial density, PV-INs serve as the main source of feed-forward inhibition in the prefrontal cortex [286,287,288]. In ALS, it is thought that the diminished inhibition leads to excitotoxicity via the disinhibition of glutamatergic circuits [285]. Studies from our laboratory indicate that these neurons express high levels of PGC-1α [104,289,290], and recently, we reviewed the known roles for PGC-1α in neurons [291]. In addition to being required for the expression of the genes involved in oxidative phosphorylation, PGC-1α is necessary for the expression of the PV-IN-enriched transcripts for neurotransmitter release and axonal integrity, including *Nefh* [110,111,112,292], whose protein product is used as a biomarker of axonal damage in ALS [293,294]. Interestingly, PGC-1α expression is reduced in TDP-43 modes, both in vitro and in mouse models [253,283,295]. While no evidence currently exists for a direct interaction between PGC-1α and the proteins implicated in ALS pathophysiology, it is possible that the deficiency of PGC-1α and/or PGC-1α-dependent transcripts could contribute to the interneuron dysfunction in ALS, which is notably preceded by an accumulation of ROS and mitochondrial dysfunction [285]. 

#### 3.2.2. Astrocyte Involvement in ALS

There is substantial evidence to indicate the involvement of astrocyte dysfunction in ALS (recently reviewed in [296]). *SOD1* ALS mutations are shown to affect both neuronal and non-neuronal populations, and along with TDP-43 overexpression, *SOD1* mutations lead to gliosis and the loss of glial support in the vicinity of motor neurons and interneurons [13,246,297,298,299]. Glia are seen to be especially affected in *SOD1* G38A model systems [300], and late-stage *SOD1*-mutation-carrying patients [301]. It is thought that these mutations ultimately lead to the loss of the ability to provide GSH and other reactive species scavengers to local neurons [257,302]. In fact, neuron loss is reported in a mouse model with the selective expression of mutant *SOD1* in astrocytes [303]. Considering the enrichment of *Nfe2l2* expressions in astrocytes, compared to neurons [4], it is interesting to speculate how disrupted NRF2 activation could influence astrocyte function. Experiments in [304] showed that the treatment of cultured astrocytes with tBHQ or with transfection by *Nrf2* was sufficient to increase glutathione activity and protect motor neurons from toxicity mediated by G93A-expressing astrocytes.

Additionally, glutamate excitotoxicity has been observed in both familial and sporadic ALS cases [44,273,305]; considering the pivotal role astrocytes play in the uptake of extracellular glutamate [306], their dysfunction could be a source of excitotoxicity-mediated cell death in ALS [276,307]. Alternatively, pathologically-affected astrocytes could also contribute to ALS-linked neuron loss through the interuption of astrocyte-neuron cross-talk or an interference in the provision of metabolic substrates to neurons [25,196,308].

#### 3.2.3. ALS and NRF2

While several studies have investigated the putative neuroprotective benefits of the NRF2-dependent pathway activation using transgenics and NRF2 modulators in cell cultures and in in vivo models of ALS, results have been mixed. The *Nef2l2*/NRF2 transcript and protein levels were reduced in NSC-34 (neuron-like) cells transfected with G93A or G37R, mutated *SOD1*, with the coordinated downregulation of putative NRF2 targets including *G6pdx*, *Prdx3*, and *Prdx4* [309], suggesting that ALS-associated mutations lead to a downregulation of NRF2-ARE regulon expression. In contrast, a study using an ARE reporter mouse reported that *SOD1* mutations led to an activation of ARE-containing genes [203]. To observe NRF2 activity in vivo, Dr. Jeffrey Johnson’s group developed a reporter mouse line with human placental alkaline phosphatase (hPAP) driven by the ARE from the rat *Nqo1* promoter [310]. Kraft et al. [203] found that when these ARE reporter mice are crossed with G93A or H46R/Q SOD1 animals, hPAP immunoreactivity, a proxy for NRF2 activity in this model, was observed in muscles at early timepoints, as well as in motor neurons at later timepoints. The differences between the cell culture and the in vivo studies highlight the importance of using in vivo models with cell type-specific resolution to determine the impact of ALS-associated mutations on NRF2 biology. Similarly, Neymotin et al. [311] reported that NRF2 activators attenuated the oxidative stress response in fibroblasts, with cytoprotective effects absent in NRF2-null fibroblasts. While these effects have not been recapitulated in neuronal cell lines or in vivo, a recent study by Wen et al. [312] confirmed NRF2 pathway activation with the triterpenoids CDDO-EA and CDDO-TFEA. Following triterpenoid treatment, they showed an increased expression of *Nqo1*, *Gsta3*, and *Hmox1*, a reduction in inflammatory markers, and an increased expression of *Coxii*, *Esrra*, *Nrf1*, and *Ppargc1a* in the spinal cord of G93A mutant mice [312]. However, in the context of our data showing the enrichment of *Nqo1* and *Nfe2l2* in astrocytes and *Ppargc1a* and *Esrra* in neurons [291,313], it is likely that these factors activate distinct transcriptional programs in different cell types. In neurodegenerative disease, an important consideration is the impact of cell damage/loss when evaluating changes in gene expression in tissue homogenates. To determine what cell population is contributing to changes in expression, as well as that changes are not simply an artefact of cell loss, single-cell labeling/sequencing is necessary.

ARE-containing gene expression is likely context- and cell type-dependent, with cell type-specific regulators determining the differential expressions of targets. As multiple transcriptional regulators with ARE-binding capabilities have been identified (including other NRF family members, mentioned above), NRF2 is probably not the sole mediator of ARE-containing gene-expression. In fact, major ARE-binding proteins that are seen to be activated by the antioxidant tertiary butylhydroquinone (tBHQ) also include the transcription factors c-Jun and members of the AP-1 complex [314]. Multiple studies also provided evidence that the nuclear localization of NRF2 alone is not sufficient to activate ARE-containing gene expression. In [315], when neuroblastoma cells were treated with hydrogen peroxide, SOD1 expression increased with concurrent NRF2 nuclear translocation; however, there were no observed changes in NRF2 binding at the *SOD1* promoter. Further studies showed that mutant forms of TDP-43 also induced NRF2 translocation to the nucleus without the activation of downstream gene targets (*Hmox1*) [316,317]. Altogether, these studies raise the possibility that the activation of transcriptional programs for antioxidant defense could require the activation and recruitment of other transcriptional regulators for the cell to mount a robust transcriptional response.

#### 3.2.4. KEAP1 Interactors in the Context of ALS/FTD

KEAP1-centric protein complexes in the cytoplasm provide an additional regulation of NRF2 translocation/activation. At steady states, KEAP1 sequesters NRF2, but when oxidative stress increases within the cell, the electrophilic binding partners of KEAP1 allow for the displacement of NRF2, which can then translocate into the nucleus. One of these KEAP1 binding partners, SQSTM1, an autophagy-related protein, has been shown to interact with both KEAP1 and NRF2 [102]. Mutations in SQSTM1, a component of the LC3-mediated autophagy pathway, are associated with familial ALS [318,319,320]. Studies also show that the loss of SQSTM1 is linked to a shorter lifespan in mSOD1 mouse models [321], potentially due to an increase in mSOD1-positive inclusions and elevated oxidative stress. However, contrary to initial predictions, the overexpression of SQSTM1 using a ubiquitous promoter (CAG) led to accelerated mortality in the H46R SOD1 mouse model, with no observed impact on SOD1 aggregation [322]. These mice form aggregations containing both SOD1 and SQSTM1 in the anterior horn of the lumbar spinal cord, gliosis, and a reduction in *Nqo1* expression. Later studies found that mutations in SQSTM1 are causal for FTD, potentially by promoting the stabilization of KEAP1/NRF2 even with oxidative modification of NRF2 [323]. Recent studies also indicated that the G427R mutation in SQSTM1 impaired the interaction of KEAP1-SQSTM1, leading to the downregulation of NRF2-dependent genes and an increase in TDP-43-associated stress granule formation [100]. Interestingly, this study found a reduction in *Gstm1* expression in SQSTM1 knockout brains; as shown in Figure 3c, *Gstm1* is highly expressed in astrocytes. Altogether, these studies suggest that the level of SQSTM1 must be tightly regulated to promote the normal function of KEAP1 and that SQSTM1 could have a role in the regulation of astrocyte gene expression, potentially via modulating NRF2 pathways. Additional studies should explore the astrocyte-specific functions of SQSTM1.

Mutations in TANK-binding kinase 1 (*TBK1*), which encodes another component of the selective autophagy pathway, leads to the decreased ubiquitination of KEAP1, preventing the release of NRF2 [100]. Interestingly, wild-type TBK1 can phosphorylate SQSTM1 and act as an inhibitor of NRF2 activity, while some mutations (p.G175S) lose this inhibitory activity, indicating a complicated relationship between TBK1, SQSTM1 modifications, and the NRF2 pathway [324]. Future studies should explore the relationship among these proteins in both astrocytes and neurons to determine the cellular mechanisms contributing to ALS and FTD with SQSTM1 or TBK1 mutations.

## 4. The Cell type-specificity of KEAP1-Centric Therapeutic Strategies

Recent reviews have covered the main tools used to amplify NRF2-signaling pathways in the context of neurodegeneration, including electrophiles, protein-protein-interaction inhibitors, and multi-target drugs [325,326,327,328]. Interestingly, most of these approaches stimulate NRF2 translocation to the nucleus by interfering with NRF2 and KEAP1 interactions. With this in mind, here we focus on KEAP1-centric strategies, with the consideration and discussion of the cell type-specific distributions of *Keap1* and some of its well-characterized interacting partners relevant for PD and ALS.

### 4.1. Cellular Enrichment of KEAP1 and Its Interactors

While some studies have documented neuronal NRF2 nuclear translocation (e.g., in DAergic neurons in PD post-mortem tissue [184]), the majority of studies suggest that NRF2 expression is enriched in non-neuronal cells such as astrocytes, microglia, macrophages, and endothelial cells (Figure 2b and Figure 3). Therefore, KEAP1-targeted strategies may be more likely to cause NRF2 translocation in non-neuronal cells, unless *Nfe2l2*/NRF2 is induced or stabilized in neurons in a pathological state. However, it is important to note that the translocation of NRF2 to the nucleus could be desired in non-neuronal cells; in fact, promoting the translocation of NRF2 in microglia, macrophages, and astrocytes can reduce the production of inflammatory cytokines [329].

**Figure 5:**
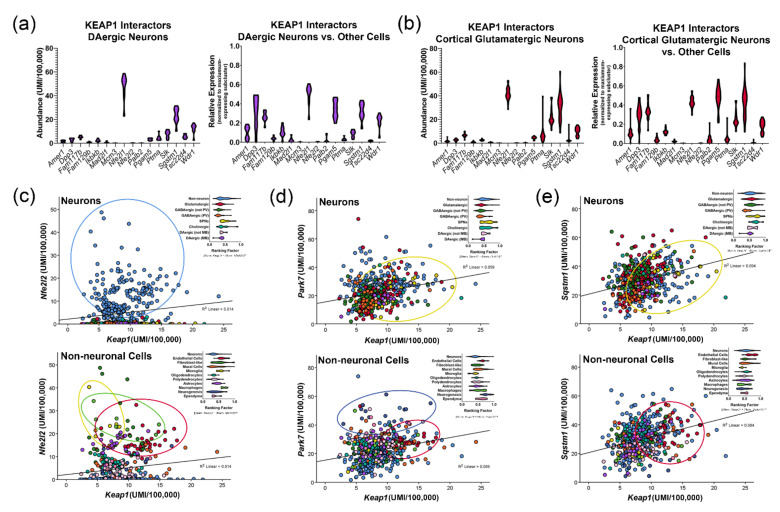
Data from Dropviz.org (accessed on 9 December 2021) [4] were mined to explore cell type-specific expression of KEAP1 interactors in DAergic neurons (relevant to therapeutic targeting in PD) (**a**), and in cortical glutamatergic neurons (relevant to therapeutic targeting of excitotoxicity in ALS) (**b**) in the neuroanatomical relationship between transcript abundance for *Keap1* and *Nfe2l2* (**c**), *Park7* (**d**), and *Sqstm1* (**e**). Values are expressed as UMI/100,000 in graphs and as “Ranking factor” for cell type-specific patterns of expression in figure insets. Ranking factors were generated by calculating the distance from the origin for each cell type using the equation [x2 + y2]^0.5^, where (x,y) represent UMI/100,000 values normalized to the highest expressing subcluster. Statistics are provided in Supplementary Table S1.

Considering that many of the current approaches target KEAP1 interaction sites and KEAP1 can interact with a number of factors in the cytoplasm [102,330,331,332,333], it is important to take into account NRF2-independent KEAP1 interactions that could be affected by KEAP1 inhibition [334]. With this in mind, we explored the abundance and enrichment of KEAP1-interactors in neurons [334], with a focus on DAergic neurons of the midbrain (PD) and cortical excitatory neurons (ALS), which are well-annotated in the Dropviz.org database [4]. We found that, of all the KEAP1 interactors included in this analysis, *Nfe2l1* and *Sqstm1* mRNAs were the most abundant in DAergic neurons (Figure 5a). When the relationship between *Keap1* and *Sqstm1* expression was explored across all cell types in the database, *Sqstm1* was expressed in the majority of cell types in which *Keap1* was expressed, with a strong direct relationship between *Sqstm1* and *Keap1* expression (Figure 5b,c). Within the neuronal populations, the spiny projection neurons of the striatum expressed the highest level of *Keap1* and *Sqstm1* transcripts (Figure 5b); within non-neuronal cells, endothelial cells expressed the highest levels of *Keap1* and *Sqstm1*. If protein abundance mirrors mRNA abundance in these cellular populations, we would predict that KEAP1-targeted strategies may impact those cellular populations to the greatest extent. Similar types of strategies could be used to prioritize the combinations of factors most likely affected by KEAP1 inhibition in cell types of interest, with confirmation in relevant cell types using methods such as proximity ligation [335,336,337,338], BioID [336,339,340], or Apex2 approaches [341].

### 4.2. The Need for Target Engagement Studies in Models of Neurodegeneration

One of the most important considerations in the models of therapeutic intervention is the assessment of target engagement. Without information regarding the ability of the intervention (e.g., test compound) to effectively engage the intended target, it is impossible to determine if the effects of treatment are due to the manipulation of the intended molecular interactions or the pathway. Measures of target engagement are rarely included in therapeutic studies in mouse models of neurodegeneration. For example, while Yang et al. [342] reported that the triterpenoid CDDO-MA can induce nuclear translocation of NRF2 in neuroblastoma cells and its actions are blocked in NRF2-null fibroblasts, when the efficacy of the triterpenoid CDDO-MA was tested in a mouse model for MPTP-induced DAergic degeneration, the authors did not test whether treatment with the compound increased NRF2 nuclear localization or ARE-containing gene expression in the brain. Target engagement measures are particularly important, especially in studies involving peripherally administered toxins such as MPTP, because seemingly neuroprotective events could be mediated by the effects on peripheral tissues involved in the metabolism of MPTP (or other neurotoxins). Triterpenoids can have robust effects on the liver [343,344,345,346,347]; it is possible that CDDO treatment influences the metabolism of MPTP in the liver. However, it is important to note that neuroprotective effects of CDDO-ethyl amide were observed in a transgenic model of Huntington’s disease [348] with an upregulation of ARE-containing genes in the brain (*Hmox1*, *Gts3a*, and *Nqo1*); however, this study did not evaluate nuclear localization of NRF2, so it is not clear whether the effects were mediated by NRF2 activation. Considering the concentration of NRF2 and these genes in astrocytes, it would be interesting to determine whether the neuroprotection was mediated by effects in astrocytes; in fact, a recent study found that NRF2 is enriched in astrocytes in the rat 6-OHDA and drosophila rotenone models and that the overexpression of NRF2 in astrocytes provided more neuroprotection than the overexpression in neurons [349]. However, it is interesting to note that the NRF2 knockdown alone caused neurodegeneration in drosophila and that CDDO-Me could attenuate this toxicity, suggesting NRF2-independent roles for CDDO-Me in regulating ARE-responsive transcripts [349]. Future studies are necessary to determine which ARE responses are KEAP1 and NRF2-dependent so that the appropriate strategies can be employed depending on the desired cell type-specific effects.

There is substantial evidence indicating that translocation of NRF2 to the nucleus and subsequent, subsequently, the activation of genes with AREs are involved in the cellular response to oxidative stress. While strategies have been developed to modulate NRF2 activity via its direct activation, or through the inhibition of KEAP1, there are concerns of off-target, or ‘mutli-target’ engagement [350]. For diseases with widespread peripheral inflammation, multi-target strategies are logical, but nonspecific effects in the nervous system may have more deleterious consequences. There are several NRF2-modulating strategies currently being investigated at the preclinical stage, most notably, the series of KEAP1 inhibitors developed by Keapstone Therapeutics; however, for the treatment of neurodegenerative diseases involving oxidative stress in the brain (e.g. PD and ALS), there are no current treatments being utilized in patients (reviewed in [351,352]). Additionally, while several preclinical studies report NRF2-dependent benefits in various models of non-CNS diseases (reviewed in [351,352]), direct evidence of target engagement in these studies is lacking.

## 5. Conclusions

Here, we utilized a single-cell transcriptomic database to explore the cell type-specific distributions of members of the *Nfe2*/NRF transcription factor family, and their putative targets, to identify the cell types most likely affected by NRF2 pathway activation. Concordant with most previous work regarding the cell type-specific expression of *Nfe2l2*, we found the enrichment of this factor and several of its downstream target genes in non-neuronal cells of the mouse brain, with low basal expressions in neurons (Figure 3a). The relatively ubiquitous expression of some of the downstream targets we investigated in neurons and non-neurons (e.g., *Gclm*, *Gclc*, *Sod1*, *Sod2*, *Gpxl1*, and *Gpxl2*) suggests that the expression of these targets can be regulated in an NRF2-independent manner. In contrast, the NRF2-interacting protein KEAP1 is expressed ubiquitously, with a robust expression in neurons [4].

These findings suggest that neurons could be incapable of mounting a complete response for antioxidant gene transcription in times of stress, potentially contributing to their vulnerability in disease. Alternatively, other factors could be involved in REDOX sensing in neurons. In fact, recent studies have indicated that neuron-enriched members of the estrogen-related receptor (ERR) family [353,354,355] and PGC-1α [103] can serve as REDOX sensors, but these factors may also have roles in regulating non-mitochondrial genes as well, which should be considered when targeting these factors (reviewed in [291]). Our findings also suggest that therapies aimed at disrupting KEAP1 interactions could influence NRF2-independent pathways in neurons. Additional studies should use similar approaches to investigate cell type-specific patterns of gene and protein expressions to direct biological and target engagement studies in vivo.

## Data Availability

The database used for this article was published previously and is publicly accessible [4].

## Supplementary Materials

The following are available online at https://www.mdpi.com/article/10.3390/antiox11010008/s1, Supplementary Table S1: Statistical results for Figure 2 and Figure 5.
