# Supplementary material for: The NRF2-Dependent Transcriptional Regulation of Antioxidant Defense Pathways: Relevance for Cell Type-Specific Vulnerability to Neurodegeneration and Therapeutic Intervention
Source: Antioxidants (Basel). 2021 Dec 21;11(1):8. doi: 10.3390/antiox11010008 (PMC8772787; doi:10.3390/antiox11010008)
Supplement: Supplementary file 1 [file antioxidants-11-00008-s001.zip › antioxidants-1481550-supplementary.pdf]

Supplementary Materials: The NRF2-Dependent Transcriptional Regulation of Antioxidant Defense Pathways: Relevance for Cell Type-Specific Vulnerability to Neurodegeneration and Therapeutic Intervention

Stephanie M. Boas [1,2,†], Kathlene L. Joyce [1,2,†] and Rita M. Cowell [1,2,*]

1 Department of Neuroscience, Southern Research, 2000 9th Avenue South, Birmingham, AL 35205, USA; boas2@uab.edu (S.M.B.); kjoyce@uab.edu (K.L.J.)
2 Department of Cell, Developmental, and Integrative Biology, University of Alabama at Birmingham, 1720 2nd Avenue South, Birmingham, AL 35294, USA
* Correspondence: rcowell@southernresearch.org
† These authors contributed equally to this work.

Nfe2l2-neuron

Number of families	1
Number of comparisons per family	28
Alpha	0.05

Dunn's multiple comparisons test	Mean rank diff.	Significant?	Summary	Adjusted P Value	
Non-neuron vs. Glutamatergic	-141.9	Yes	****	<0.0001	A-B
Non-neuron vs. GABAergic (not PV)	-206.2	Yes	****	<0.0001	A-C
Non-neuron vs. GABAergic (PV)	-211.5	Yes	****	<0.0001	A-D
Non-neuron vs. SPNs	-38.42	No	ns	>0.9999	A-E
Non-neuron vs. Cholinergic	-269.1	Yes	****	<0.0001	A-F
Non-neuron vs. DAergic (not MB)	-123.1	No	ns	>0.9999	A-G
Non-neuron vs. DAergic (MB)	-265.8	Yes	****	<0.0001	A-H
Glutamatergic vs. GABAergic (not PV)	-64.27	No	ns	0.0628	B-C
Glutamatergic vs. GABAergic (PV)	-69.59	No	ns	0.5598	B-D
Glutamatergic vs. SPNs	103.5	No	ns	0.2873	B-E
Glutamatergic vs. Cholinergic	-127.1	No	ns	0.7358	B-F
Glutamatergic vs. DAergic (not MB)	18.84	No	ns	>0.9999	B-G
Glutamatergic vs. DAergic (MB)	-123.9	No	ns	0.6178	B-H
GABAergic (not PV) vs. GABAergic (PV)	-5.318	No	ns	>0.9999	C-D
GABAergic (not PV) vs. SPNs	167.8	Yes	**	0.0016	C-E
GABAergic (not PV) vs. Cholinergic	-62.87	No	ns	>0.9999	C-F
GABAergic (not PV) vs. DAergic (not MB)	83.11	No	ns	>0.9999	C-G
GABAergic (not PV) vs. DAergic (MB)	-59.61	No	ns	>0.9999	C-H
GABAergic (PV) vs. SPNs	173.1	Yes	**	0.0062	D-E
GABAergic (PV) vs. Cholinergic	-57.55	No	ns	>0.9999	D-F
GABAergic (PV) vs. DAergic (not MB)	88.42	No	ns	>0.9999	D-G
GABAergic (PV) vs. DAergic (MB)	-54.29	No	ns	>0.9999	D-H
SPNs vs. Cholinergic	-230.7	Yes	*	0.0182	E-F
SPNs vs. DAergic (not MB)	-84.69	No	ns	>0.9999	E-G
SPNs vs. DAergic (MB)	-227.4	Yes	*	0.0132	E-H
Cholinergic vs. DAergic (not MB)	146	No	ns	>0.9999	F-G

Cholinergic vs. DAergic (MB)	3.264	No	ns	>0.9999	F-H
DAergic (not MB) vs. DAergic (MB)	-142.7	No	ns	>0.9999	G-H

Test details	Mean rank 1	Mean rank 2	Mean rank diff.	n1	n2	Z
Non-neuron vs. Glutamatergic	176.3	318.2	-141.9	231	153	8.632
Non-neuron vs. GABAergic (not PV)	176.3	382.5	-206.2	231	89	10.48
Non-neuron vs. GABAergic (PV)	176.3	387.8	-211.5	231	34	7.3
Non-neuron vs. SPNs	176.3	214.7	-38.42	231	17	0.969
Non-neuron vs. Cholinergic	176.3	445.4	-269.1	231	8	4.743
Non-neuron vs. DAergic (not MB)	176.3	299.4	-123.1	231	5	1.726
Non-neuron vs. DAergic (MB)	176.3	442.1	-265.8	231	9	4.959
Glutamatergic vs. GABAergic (not PV)	318.2	382.5	-64.27	153	89	3.056
Glutamatergic vs. GABAergic (PV)	318.2	387.8	-69.59	153	34	2.326
Glutamatergic vs. SPNs	318.2	214.7	103.5	153	17	2.567
Glutamatergic vs. Cholinergic	318.2	445.4	-127.1	153	8	2.222
Glutamatergic vs. DAergic (not MB)	318.2	299.4	18.84	153	5	0.2627
Glutamatergic vs. DAergic (MB)	318.2	442.1	-123.9	153	9	2.289
GABAergic (not PV) vs. GABAergic (PV)	382.5	387.8	-5.318	89	34	0.1672
GABAergic (not PV) vs. SPNs	382.5	214.7	167.8	89	17	4.018
GABAergic (not PV) vs. Cholinergic	382.5	445.4	-62.87	89	8	1.08
GABAergic (not PV) vs. DAergic (not MB)	382.5	299.4	83.11	89	5	1.146
GABAergic (not PV) vs. DAergic (MB)	382.5	442.1	-59.61	89	9	1.08
GABAergic (PV) vs. SPNs	387.8	214.7	173.1	34	17	3.694
GABAergic (PV) vs. Cholinergic	387.8	445.4	-57.55	34	8	0.9284
GABAergic (PV) vs. DAergic (not MB)	387.8	299.4	88.42	34	5	1.17
GABAergic (PV) vs. DAergic (MB)	387.8	442.1	-54.29	34	9	0.918
SPNs vs. Cholinergic	214.7	445.4	-230.7	17	8	3.41
SPNs vs. DAergic (not MB)	214.7	299.4	-84.69	17	5	1.055
SPNs vs. DAergic (MB)	214.7	442.1	-227.4	17	9	3.497
Cholinergic vs. DAergic (not MB)	445.4	299.4	146	8	5	1.623
Cholinergic vs. DAergic (MB)	445.4	442.1	3.264	8	9	0.04258
DAergic (not MB) vs. DAergic (MB)	299.4	442.1	-142.7	5	9	1.622

Nfe2l2-neuron

Number of families	1
Number of comparisons per family	28
Alpha	0.05

Dunn's multiple comparisons test	Mean rank diff.	Significant?	Summary	Adjusted P Value	
Non-neuron vs. Glutamatergic	266.2	Yes	****	<0.0001	A-B
Non-neuron vs. GABAergic (not PV)	264.2	Yes	****	<0.0001	A-C
Non-neuron vs. GABAergic (PV)	275.2	Yes	****	<0.0001	A-D
Non-neuron vs. SPNs	249.3	Yes	****	<0.0001	A-E
Non-neuron vs. Cholinergic	201.3	Yes	**	0.0099	A-F
Non-neuron vs. DAergic (not MB)	237.3	Yes	*	0.0226	A-G

Non-neuron vs. DAergic (MB)	272.6	Yes	****	<0.0001	A-H
Glutamatergic vs. GABAergic (not PV)	-2.016	No	ns	>0.9999	B-C
Glutamatergic vs. GABAergic (PV)	8.944	No	ns	>0.9999	B-D
Glutamatergic vs. SPNs	-16.97	No	ns	>0.9999	B-E
Glutamatergic vs. Cholinergic	-64.93	No	ns	>0.9999	B-F
Glutamatergic vs. DAergic (not MB)	-28.96	No	ns	>0.9999	B-G
Glutamatergic vs. DAergic (MB)	6.333	No	ns	>0.9999	B-H
GABAergic (not PV) vs. GABAergic (PV)	10.96	No	ns	>0.9999	C-D
GABAergic (not PV) vs. SPNs	-14.95	No	ns	>0.9999	C-E
GABAergic (not PV) vs. Cholinergic	-62.91	No	ns	>0.9999	C-F
GABAergic (not PV) vs. DAergic (not MB)	-26.94	No	ns	>0.9999	C-G
GABAergic (not PV) vs. DAergic (MB)	8.35	No	ns	>0.9999	C-H
GABAergic (PV) vs. SPNs	-25.91	No	ns	>0.9999	D-E
GABAergic (PV) vs. Cholinergic	-73.88	No	ns	>0.9999	D-F
GABAergic (PV) vs. DAergic (not MB)	-37.9	No	ns	>0.9999	D-G
GABAergic (PV) vs. DAergic (MB)	-2.611	No	ns	>0.9999	D-H
SPNs vs. Cholinergic	-47.96	No	ns	>0.9999	E-F
SPNs vs. DAergic (not MB)	-11.99	No	ns	>0.9999	E-G
SPNs vs. DAergic (MB)	23.3	No	ns	>0.9999	E-H
Cholinergic vs. DAergic (not MB)	35.98	No	ns	>0.9999	F-G
Cholinergic vs. DAergic (MB)	71.26	No	ns	>0.9999	F-H
DAergic (not MB) vs. DAergic (MB)	35.29	No	ns	>0.9999	G-H

Test details	Mean rank 1	Mean rank 2	Mean rank diff.	n1	n2	Z
Non-neuron vs. Glutamatergic	425.7	159.4	266.2	231	153	16.3
Non-neuron vs. GABAergic (not PV)	425.7	161.5	264.2	231	89	13.52
Non-neuron vs. GABAergic (PV)	425.7	150.5	275.2	231	34	9.563
Non-neuron vs. SPNs	425.7	176.4	249.3	231	17	6.332
Non-neuron vs. Cholinergic	425.7	224.4	201.3	231	8	3.573
Non-neuron vs. DAergic (not MB)	425.7	188.4	237.3	231	5	3.351
Non-neuron vs. DAergic (MB)	425.7	153.1	272.6	231	9	5.121
Glutamatergic vs. GABAergic (not PV)	159.4	161.5	-2.016	153	89	0.09654
Glutamatergic vs. GABAergic (PV)	159.4	150.5	8.944	153	34	0.3011
Glutamatergic vs. SPNs	159.4	176.4	-16.97	153	17	0.4236
Glutamatergic vs. Cholinergic	159.4	224.4	-64.93	153	8	1.143
Glutamatergic vs. DAergic (not MB)	159.4	188.4	-28.96	153	5	0.4067
Glutamatergic vs. DAergic (MB)	159.4	153.1	6.333	153	9	0.1179
GABAergic (not PV) vs. GABAergic (PV)	161.5	150.5	10.96	89	34	0.347
GABAergic (not PV) vs. SPNs	161.5	176.4	-14.95	89	17	0.3605
GABAergic (not PV) vs. Cholinergic	161.5	224.4	-62.91	89	8	1.088
GABAergic (not PV) vs. DAergic (not MB)	161.5	188.4	-26.94	89	5	0.3741
GABAergic (not PV) vs. DAergic (MB)	161.5	153.1	8.35	89	9	0.1524
GABAergic (PV) vs. SPNs	150.5	176.4	-25.91	34	17	0.5568
GABAergic (PV) vs. Cholinergic	150.5	224.4	-73.88	34	8	1.2
GABAergic (PV) vs. DAergic (not MB)	150.5	188.4	-37.9	34	5	0.5051
GABAergic (PV) vs. DAergic (MB)	150.5	153.1	-2.611	34	9	0.04446

SPNs vs. Cholinergic	176.4	224.4	-47.96	17	8	0.714
SPNs vs. DAergic (not MB)	176.4	188.4	-11.99	17	5	0.1504
SPNs vs. DAergic (MB)	176.4	153.1	23.3	17	9	0.3608
Cholinergic vs. DAergic (not MB)	224.4	188.4	35.98	8	5	0.4028
Cholinergic vs. DAergic (MB)	224.4	153.1	71.26	8	9	0.9361
DAergic (not MB) vs. DAergic (MB)	188.4	153.1	35.29	5	9	0.4038

Nfe2l3-neuron

Number of families	1
Number of comparisons per family	28
Alpha	0.05

Dunn's multiple comparisons test	Mean rank diff.	Significant?	Summary	Adjusted P Value	
Non-neuron vs. Glutamatergic	-18.09	No	ns	>0.9999	A-B
Non-neuron vs. GABAergic (not PV)	66.32	Yes	**	0.0097	A-C
Non-neuron vs. GABAergic (PV)	70.19	No	ns	0.2833	A-D
Non-neuron vs. SPNs	24.19	No	ns	>0.9999	A-E
Non-neuron vs. Cholinergic	127.1	No	ns	0.4865	A-F
Non-neuron vs. DAergic (not MB)	156.2	No	ns	0.5618	A-G
Non-neuron vs. DAergic (MB)	60.66	No	ns	>0.9999	A-H
Glutamatergic vs. GABAergic (not PV)	84.41	Yes	***	0.0006	B-C
Glutamatergic vs. GABAergic (PV)	88.27	Yes	*	0.0483	B-D
Glutamatergic vs. SPNs	42.27	No	ns	>0.9999	B-E
Glutamatergic vs. Cholinergic	145.2	No	ns	0.1975	B-F
Glutamatergic vs. DAergic (not MB)	174.2	No	ns	0.2762	B-G
Glutamatergic vs. DAergic (MB)	78.75	No	ns	>0.9999	B-H
GABAergic (not PV) vs. GABAergic (PV)	3.866	No	ns	>0.9999	C-D
GABAergic (not PV) vs. SPNs	-42.13	No	ns	>0.9999	C-E
GABAergic (not PV) vs. Cholinergic	60.77	No	ns	>0.9999	C-F
GABAergic (not PV) vs. DAergic (not MB)	89.84	No	ns	>0.9999	C-G
GABAergic (not PV) vs. DAergic (MB)	-5.663	No	ns	>0.9999	C-H
GABAergic (PV) vs. SPNs	-46	No	ns	>0.9999	D-E
GABAergic (PV) vs. Cholinergic	56.91	No	ns	>0.9999	D-F
GABAergic (PV) vs. DAergic (not MB)	85.97	No	ns	>0.9999	D-G
GABAergic (PV) vs. DAergic (MB)	-9.529	No	ns	>0.9999	D-H
SPNs vs. Cholinergic	102.9	No	ns	>0.9999	E-F
SPNs vs. DAergic (not MB)	132	No	ns	>0.9999	E-G
SPNs vs. DAergic (MB)	36.47	No	ns	>0.9999	E-H
Cholinergic vs. DAergic (not MB)	29.06	No	ns	>0.9999	F-G
Cholinergic vs. DAergic (MB)	-66.44	No	ns	>0.9999	F-H
DAergic (not MB) vs. DAergic (MB)	-95.5	No	ns	>0.9999	G-H

Test details	Mean rank 1	Mean rank 2	Mean rank diff.	n1	n2	Z
Non-neuron vs. Glutamatergic	288.7	306.7	-18.09	231	153	1.168
Non-neuron vs. GABAergic (not PV)	288.7	222.3	66.32	231	89	3.578

Non-neuron vs. GABAergic (PV)	288.7	218.5	70.19	231	34	2.572
Non-neuron vs. SPNs	288.7	264.5	24.19	231	17	0.6478
Non-neuron vs. Cholinergic	288.7	161.6	127.1	231	8	2.379
Non-neuron vs. DAergic (not MB)	288.7	132.5	156.2	231	5	2.325
Non-neuron vs. DAergic (MB)	288.7	228	60.66	231	9	1.202
Glutamatergic vs. GABAergic (not PV)	306.7	222.3	84.41	153	89	4.262
Glutamatergic vs. GABAergic (PV)	306.7	218.5	88.27	153	34	3.134
Glutamatergic vs. SPNs	306.7	264.5	42.27	153	17	1.113
Glutamatergic vs. Cholinergic	306.7	161.6	145.2	153	8	2.694
Glutamatergic vs. DAergic (not MB)	306.7	132.5	174.2	153	5	2.581
Glutamatergic vs. DAergic (MB)	306.7	228	78.75	153	9	1.545
GABAergic (not PV) vs. GABAergic (PV)	222.3	218.5	3.866	89	34	0.1291
GABAergic (not PV) vs. SPNs	222.3	264.5	-42.13	89	17	1.071
GABAergic (not PV) vs. Cholinergic	222.3	161.6	60.77	89	8	1.108
GABAergic (not PV) vs. DAergic (not MB)	222.3	132.5	89.84	89	5	1.316
GABAergic (not PV) vs. DAergic (MB)	222.3	228	-5.663	89	9	0.109
GABAergic (PV) vs. SPNs	218.5	264.5	-46	34	17	1.042
GABAergic (PV) vs. Cholinergic	218.5	161.6	56.91	34	8	0.9747
GABAergic (PV) vs. DAergic (not MB)	218.5	132.5	85.97	34	5	1.208
GABAergic (PV) vs. DAergic (MB)	218.5	228	-9.529	34	9	0.1711
SPNs vs. Cholinergic	264.5	161.6	102.9	17	8	1.615
SPNs vs. DAergic (not MB)	264.5	132.5	132	17	5	1.746
SPNs vs. DAergic (MB)	264.5	228	36.47	17	9	0.5955
Cholinergic vs. DAergic (not MB)	161.6	132.5	29.06	8	5	0.3431
Cholinergic vs. DAergic (MB)	161.6	228	-66.44	8	9	0.9202
DAergic (not MB) vs. DAergic (MB)	132.5	228	-95.5	5	9	1.152

Bach1-neuron

Number of families	1
Number of comparisons per family	28
Alpha	0.05

Dunn's multiple comparisons test	Mean rank diff.	Significant?	Summary	Adjusted P Value	
Non-neuron vs. Glutamatergic	242.8	Yes	****	<0.0001	A-B
Non-neuron vs. GABAergic (not PV)	132.1	Yes	****	<0.0001	A-C
Non-neuron vs. GABAergic (PV)	118.8	Yes	**	0.0012	A-D
Non-neuron vs. SPNs	298.9	Yes	****	<0.0001	A-E
Non-neuron vs. Cholinergic	133.6	No	ns	0.5174	A-F
Non-neuron vs. DAergic (not MB)	135.8	No	ns	>0.9999	A-G
Non-neuron vs. DAergic (MB)	207.6	Yes	**	0.003	A-H
Glutamatergic vs. GABAergic (not PV)	-110.7	Yes	****	<0.0001	B-C
Glutamatergic vs. GABAergic (PV)	-124	Yes	***	0.0009	B-D
Glutamatergic vs. SPNs	56.12	No	ns	>0.9999	B-E
Glutamatergic vs. Cholinergic	-109.2	No	ns	>0.9999	B-F
Glutamatergic vs. DAergic (not MB)	-107	No	ns	>0.9999	B-G

Glutamatergic vs. DAergic (MB)	-35.19	No	ns	>0.9999	B-H
GABAergic (not PV) vs. GABAergic (PV)	-13.3	No	ns	>0.9999	C-D
GABAergic (not PV) vs. SPNs	166.8	Yes	**	0.0018	C-E
GABAergic (not PV) vs. Cholinergic	1.565	No	ns	>0.9999	C-F
GABAergic (not PV) vs. DAergic (not MB)	3.715	No	ns	>0.9999	C-G
GABAergic (not PV) vs. DAergic (MB)	75.54	No	ns	>0.9999	C-H
GABAergic (PV) vs. SPNs	180.1	Yes	**	0.0034	D-E
GABAergic (PV) vs. Cholinergic	14.87	No	ns	>0.9999	D-F
GABAergic (PV) vs. DAergic (not MB)	17.02	No	ns	>0.9999	D-G
GABAergic (PV) vs. DAergic (MB)	88.84	No	ns	>0.9999	D-H
SPNs vs. Cholinergic	-165.3	No	ns	0.407	E-F
SPNs vs. DAergic (not MB)	-163.1	No	ns	>0.9999	E-G
SPNs vs. DAergic (MB)	-91.31	No	ns	>0.9999	E-H
Cholinergic vs. DAergic (not MB)	2.15	No	ns	>0.9999	F-G
Cholinergic vs. DAergic (MB)	73.97	No	ns	>0.9999	F-H
DAergic (not MB) vs. DAergic (MB)	71.82	No	ns	>0.9999	G-H

Test details	Mean rank 1	Mean rank 2	Mean rank diff.	n1	n2	Z
Non-neuron vs. Glutamatergic	386.4	143.6	242.8	231	153	14.77
Non-neuron vs. GABAergic (not PV)	386.4	254.3	132.1	231	89	6.711
Non-neuron vs. GABAergic (PV)	386.4	267.6	118.8	231	34	4.099
Non-neuron vs. SPNs	386.4	87.47	298.9	231	17	7.541
Non-neuron vs. Cholinergic	386.4	252.8	133.6	231	8	2.356
Non-neuron vs. DAergic (not MB)	386.4	250.6	135.8	231	5	1.904
Non-neuron vs. DAergic (MB)	386.4	178.8	207.6	231	9	3.874
Glutamatergic vs. GABAergic (not PV)	143.6	254.3	-110.7	153	89	5.265
Glutamatergic vs. GABAergic (PV)	143.6	267.6	-124	153	34	4.147
Glutamatergic vs. SPNs	143.6	87.47	56.12	153	17	1.392
Glutamatergic vs. Cholinergic	143.6	252.8	-109.2	153	8	1.908
Glutamatergic vs. DAergic (not MB)	143.6	250.6	-107	153	5	1.493
Glutamatergic vs. DAergic (MB)	143.6	178.8	-35.19	153	9	0.6504
GABAergic (not PV) vs. GABAergic (PV)	254.3	267.6	-13.3	89	34	0.4183
GABAergic (not PV) vs. SPNs	254.3	87.47	166.8	89	17	3.996
GABAergic (not PV) vs. Cholinergic	254.3	252.8	1.565	89	8	0.02687
GABAergic (not PV) vs. DAergic (not MB)	254.3	250.6	3.715	89	5	0.05124
GABAergic (not PV) vs. DAergic (MB)	254.3	178.8	75.54	89	9	1.369
GABAergic (PV) vs. SPNs	267.6	87.47	180.1	34	17	3.845
GABAergic (PV) vs. Cholinergic	267.6	252.8	14.87	34	8	0.2399
GABAergic (PV) vs. DAergic (not MB)	267.6	250.6	17.02	34	5	0.2252
GABAergic (PV) vs. DAergic (MB)	267.6	178.8	88.84	34	9	1.502
SPNs vs. Cholinergic	87.47	252.8	-165.3	17	8	2.444
SPNs vs. DAergic (not MB)	87.47	250.6	-163.1	17	5	2.033
SPNs vs. DAergic (MB)	87.47	178.8	-91.31	17	9	1.404
Cholinergic vs. DAergic (not MB)	252.8	250.6	2.15	8	5	0.02391
Cholinergic vs. DAergic (MB)	252.8	178.8	73.97	8	9	0.9651
DAergic (not MB) vs. DAergic (MB)	250.6	178.8	71.82	5	9	0.8163

Bach2-neuron

Number of families	1
Number of comparisons per family	28
Alpha	0.05

Dunn's multiple comparisons test	Mean rank diff.	Significant?	Summary	Adjusted P Value	
Non-neuron vs. Glutamatergic	-45.19	No	ns	0.1665	A-B
Non-neuron vs. GABAergic (not PV)	-150.9	Yes	****	<0.0001	A-C
Non-neuron vs. GABAergic (PV)	-39.54	No	ns	>0.9999	A-D
Non-neuron vs. SPNs	-104.6	No	ns	0.2324	A-E
Non-neuron vs. Cholinergic	-20.2	No	ns	>0.9999	A-F
Non-neuron vs. DAergic (not MB)	-149.7	No	ns	0.9992	A-G
Non-neuron vs. DAergic (MB)	-189.4	Yes	*	0.0114	A-H
Glutamatergic vs. GABAergic (not PV)	-105.7	Yes	****	<0.0001	B-C
Glutamatergic vs. GABAergic (PV)	5.655	No	ns	>0.9999	B-D
Glutamatergic vs. SPNs	-59.36	No	ns	>0.9999	B-E
Glutamatergic vs. Cholinergic	25	No	ns	>0.9999	B-F
Glutamatergic vs. DAergic (not MB)	-104.5	No	ns	>0.9999	B-G
Glutamatergic vs. DAergic (MB)	-144.2	No	ns	0.2144	B-H
GABAergic (not PV) vs. GABAergic (PV)	111.3	Yes	*	0.0129	C-D
GABAergic (not PV) vs. SPNs	46.3	No	ns	>0.9999	C-E
GABAergic (not PV) vs. Cholinergic	130.7	No	ns	0.692	C-F
GABAergic (not PV) vs. DAergic (not MB)	1.196	No	ns	>0.9999	C-G
GABAergic (not PV) vs. DAergic (MB)	-38.52	No	ns	>0.9999	C-H
GABAergic (PV) vs. SPNs	-65.01	No	ns	>0.9999	D-E
GABAergic (PV) vs. Cholinergic	19.34	No	ns	>0.9999	D-F
GABAergic (PV) vs. DAergic (not MB)	-110.1	No	ns	>0.9999	D-G
GABAergic (PV) vs. DAergic (MB)	-149.8	No	ns	0.3141	D-H
SPNs vs. Cholinergic	84.36	No	ns	>0.9999	E-F
SPNs vs. DAergic (not MB)	-45.11	No	ns	>0.9999	E-G
SPNs vs. DAergic (MB)	-84.82	No	ns	>0.9999	E-H
Cholinergic vs. DAergic (not MB)	-129.5	No	ns	>0.9999	F-G
Cholinergic vs. DAergic (MB)	-169.2	No	ns	0.7613	F-H
DAergic (not MB) vs. DAergic (MB)	-39.71	No	ns	>0.9999	G-H

Test details	Mean rank 1	Mean rank 2	Mean rank diff.	n1	n2	Z
Non-neuron vs. Glutamatergic	225.7	270.9	-45.19	231	153	2.751
Non-neuron vs. GABAergic (not PV)	225.7	376.6	-150.9	231	89	7.671
Non-neuron vs. GABAergic (PV)	225.7	265.3	-39.54	231	34	1.366
Non-neuron vs. SPNs	225.7	330.3	-104.6	231	17	2.64
Non-neuron vs. Cholinergic	225.7	245.9	-20.2	231	8	0.3563
Non-neuron vs. DAergic (not MB)	225.7	375.4	-149.7	231	5	2.101
Non-neuron vs. DAergic (MB)	225.7	415.1	-189.4	231	9	3.536
Glutamatergic vs. GABAergic (not PV)	270.9	376.6	-105.7	153	89	5.028
Glutamatergic vs. GABAergic (PV)	270.9	265.3	5.655	153	34	0.1892

Glutamatergic vs. SPNs	270.9	330.3	-59.36	153	17	1.473
Glutamatergic vs. Cholinergic	270.9	245.9	25	153	8	0.4373
Glutamatergic vs. DAergic (not MB)	270.9	375.4	-104.5	153	5	1.458
Glutamatergic vs. DAergic (MB)	270.9	415.1	-144.2	153	9	2.667
GABAergic (not PV) vs. GABAergic (PV)	376.6	265.3	111.3	89	34	3.503
GABAergic (not PV) vs. SPNs	376.6	330.3	46.3	89	17	1.11
GABAergic (not PV) vs. Cholinergic	376.6	245.9	130.7	89	8	2.246
GABAergic (not PV) vs. DAergic (not MB)	376.6	375.4	1.196	89	5	0.0165
GABAergic (not PV) vs. DAergic (MB)	376.6	415.1	-38.52	89	9	0.6986
GABAergic (PV) vs. SPNs	265.3	330.3	-65.01	34	17	1.389
GABAergic (PV) vs. Cholinergic	265.3	245.9	19.34	34	8	0.3123
GABAergic (PV) vs. DAergic (not MB)	265.3	375.4	-110.1	34	5	1.459
GABAergic (PV) vs. DAergic (MB)	265.3	415.1	-149.8	34	9	2.536
SPNs vs. Cholinergic	330.3	245.9	84.36	17	8	1.248
SPNs vs. DAergic (not MB)	330.3	375.4	-45.11	17	5	0.5625
SPNs vs. DAergic (MB)	330.3	415.1	-84.82	17	9	1.305
Cholinergic vs. DAergic (not MB)	245.9	375.4	-129.5	8	5	1.441
Cholinergic vs. DAergic (MB)	245.9	415.1	-169.2	8	9	2.209
DAergic (not MB) vs. DAergic (MB)	375.4	415.1	-39.71	5	9	0.4517

Keap1-neuron

Number of families	1
Number of comparisons per family	28
Alpha	0.05

Dunn's multiple comparisons test	Mean rank diff.	Significant?	Summary	Adjusted P Value	
Non-neuron vs. Glutamatergic	13.62	No	ns	>0.9999	A-B
Non-neuron vs. GABAergic (not PV)	56.19	No	ns	0.1205	A-C
Non-neuron vs. GABAergic (PV)	98.53	Yes	*	0.0189	A-D
Non-neuron vs. SPNs	-162	Yes	**	0.0012	A-E
Non-neuron vs. Cholinergic	-78.36	No	ns	>0.9999	A-F
Non-neuron vs. DAergic (not MB)	-79.11	No	ns	>0.9999	A-G
Non-neuron vs. DAergic (MB)	-49	No	ns	>0.9999	A-H
Glutamatergic vs. GABAergic (not PV)	42.57	No	ns	>0.9999	B-C
Glutamatergic vs. GABAergic (PV)	84.92	No	ns	0.1268	B-D
Glutamatergic vs. SPNs	-175.6	Yes	***	0.0004	B-E
Glutamatergic vs. Cholinergic	-91.98	No	ns	>0.9999	B-F
Glutamatergic vs. DAergic (not MB)	-92.73	No	ns	>0.9999	B-G
Glutamatergic vs. DAergic (MB)	-62.62	No	ns	>0.9999	B-H
GABAergic (not PV) vs. GABAergic (PV)	42.34	No	ns	>0.9999	C-D
GABAergic (not PV) vs. SPNs	-218.2	Yes	****	<0.0001	C-E
GABAergic (not PV) vs. Cholinergic	-134.6	No	ns	0.5837	C-F
GABAergic (not PV) vs. DAergic (not MB)	-135.3	No	ns	>0.9999	C-G
GABAergic (not PV) vs. DAergic (MB)	-105.2	No	ns	>0.9999	C-H
GABAergic (PV) vs. SPNs	-260.5	Yes	****	<0.0001	D-E
GABAergic (PV) vs. Cholinergic	-176.9	No	ns	0.1211	D-F

GABAergic (PV) vs. DAergic (not MB)	-177.6	No	ns	0.5243	D-G
GABAergic (PV) vs. DAergic (MB)	-147.5	No	ns	0.3529	D-H
SPNs vs. Cholinergic	83.63	No	ns	>0.9999	E-F
SPNs vs. DAergic (not MB)	82.88	No	ns	>0.9999	E-G
SPNs vs. DAergic (MB)	113	No	ns	>0.9999	E-H
Cholinergic vs. DAergic (not MB)	-0.75	No	ns	>0.9999	F-G
Cholinergic vs. DAergic (MB)	29.36	No	ns	>0.9999	F-H
DAergic (not MB) vs. DAergic (MB)	30.11	No	ns	>0.9999	G-H

Test details	Mean rank 1	Mean rank 2	Mean rank diff.	n1	n2	Z
Non-neuron vs. Glutamatergic	284.9	271.3	13.62	231	153	0.8282
Non-neuron vs. GABAergic (not PV)	284.9	228.7	56.19	231	89	2.855
Non-neuron vs. GABAergic (PV)	284.9	186.4	98.53	231	34	3.4
Non-neuron vs. SPNs	284.9	446.9	-162	231	17	4.086
Non-neuron vs. Cholinergic	284.9	363.3	-78.36	231	8	1.381
Non-neuron vs. DAergic (not MB)	284.9	364	-79.11	231	5	1.109
Non-neuron vs. DAergic (MB)	284.9	333.9	-49	231	9	0.9142
Glutamatergic vs. GABAergic (not PV)	271.3	228.7	42.57	153	89	2.024
Glutamatergic vs. GABAergic (PV)	271.3	186.4	84.92	153	34	2.839
Glutamatergic vs. SPNs	271.3	446.9	-175.6	153	17	4.354
Glutamatergic vs. Cholinergic	271.3	363.3	-91.98	153	8	1.608
Glutamatergic vs. DAergic (not MB)	271.3	364	-92.73	153	5	1.293
Glutamatergic vs. DAergic (MB)	271.3	333.9	-62.62	153	9	1.157
GABAergic (not PV) vs. GABAergic (PV)	228.7	186.4	42.34	89	34	1.331
GABAergic (not PV) vs. SPNs	228.7	446.9	-218.2	89	17	5.225
GABAergic (not PV) vs. Cholinergic	228.7	363.3	-134.6	89	8	2.311
GABAergic (not PV) vs. DAergic (not MB)	228.7	364	-135.3	89	5	1.866
GABAergic (not PV) vs. DAergic (MB)	228.7	333.9	-105.2	89	9	1.906
GABAergic (PV) vs. SPNs	186.4	446.9	-260.5	34	17	5.56
GABAergic (PV) vs. Cholinergic	186.4	363.3	-176.9	34	8	2.854
GABAergic (PV) vs. DAergic (not MB)	186.4	364	-177.6	34	5	2.351
GABAergic (PV) vs. DAergic (MB)	186.4	333.9	-147.5	34	9	2.495
SPNs vs. Cholinergic	446.9	363.3	83.63	17	8	1.236
SPNs vs. DAergic (not MB)	446.9	364	82.88	17	5	1.033
SPNs vs. DAergic (MB)	446.9	333.9	113	17	9	1.737
Cholinergic vs. DAergic (not MB)	363.3	364	-0.75	8	5	0.008339
Cholinergic vs. DAergic (MB)	363.3	333.9	29.36	8	9	0.383
DAergic (not MB) vs. DAergic (MB)	364	333.9	30.11	5	9	0.3422

Maf-neuron

Number of families	1
Number of comparisons per family	28
Alpha	0.05

Dunn's multiple comparisons test	Mean rank diff.	Significant?	Summary	Adjusted P Value

Non-neuron vs. Glutamatergic	127.9	Yes	****	<0.0001		A-B
Non-neuron vs. GABAergic (not PV)	-70.79	Yes	**		0.0089	A-C
Non-neuron vs. GABAergic (PV)	-58.33	No	ns	>0.9999		A-D
Non-neuron vs. SPNs	117.4	No	ns		0.085	A-E
Non-neuron vs. Cholinergic	25.84	No	ns	>0.9999		A-F
Non-neuron vs. DAergic (not MB)	7.644	No	ns	>0.9999		A-G
Non-neuron vs. DAergic (MB)	186.2	Yes	*		0.0141	A-H
Glutamatergic vs. GABAergic (not PV)	-198.7	Yes	****	<0.0001		B-C
Glutamatergic vs. GABAergic (PV)	-186.2	Yes	****	<0.0001		B-D
Glutamatergic vs. SPNs	-10.53	No	ns	>0.9999		B-E
Glutamatergic vs. Cholinergic	-102.1	No	ns	>0.9999		B-F
Glutamatergic vs. DAergic (not MB)	-120.3	No	ns	>0.9999		B-G
Glutamatergic vs. DAergic (MB)	58.33	No	ns	>0.9999		B-H
GABAergic (not PV) vs. GABAergic (PV)	12.45	No	ns	>0.9999		C-D
GABAergic (not PV) vs. SPNs	188.2	Yes	***		0.0002	C-E
GABAergic (not PV) vs. Cholinergic	96.63	No	ns	>0.9999		C-F
GABAergic (not PV) vs. DAergic (not MB)	78.43	No	ns	>0.9999		C-G
GABAergic (not PV) vs. DAergic (MB)	257	Yes	****	<0.0001		C-H
GABAergic (PV) vs. SPNs	175.7	Yes	**		0.0049	D-E
GABAergic (PV) vs. Cholinergic	84.18	No	ns	>0.9999		D-F
GABAergic (PV) vs. DAergic (not MB)	65.98	No	ns	>0.9999		D-G
GABAergic (PV) vs. DAergic (MB)	244.6	Yes	***		0.001	D-H
SPNs vs. Cholinergic	-91.53	No	ns	>0.9999		E-F
SPNs vs. DAergic (not MB)	-109.7	No	ns	>0.9999		E-G
SPNs vs. DAergic (MB)	68.86	No	ns	>0.9999		E-H
Cholinergic vs. DAergic (not MB)	-18.2	No	ns	>0.9999		F-G
Cholinergic vs. DAergic (MB)	160.4	No	ns	>0.9999		F-H
DAergic (not MB) vs. DAergic (MB)	178.6	No	ns	>0.9999		G-H

Test details	Mean rank 1	Mean rank 2	Mean rank diff.	n1	n2	Z
Non-neuron vs. Glutamatergic	301.3	173.4	127.9	231	153	7.788
Non-neuron vs. GABAergic (not PV)	301.3	372.1	-70.79	231	89	3.601
Non-neuron vs. GABAergic (PV)	301.3	359.7	-58.33	231	34	2.015
Non-neuron vs. SPNs	301.3	184	117.4	231	17	2.964
Non-neuron vs. Cholinergic	301.3	275.5	25.84	231	8	0.4561
Non-neuron vs. DAergic (not MB)	301.3	293.7	7.644	231	5	0.1073
Non-neuron vs. DAergic (MB)	301.3	115.1	186.2	231	9	3.479
Glutamatergic vs. GABAergic (not PV)	173.4	372.1	-198.7	153	89	9.459
Glutamatergic vs. GABAergic (PV)	173.4	359.7	-186.2	153	34	6.234
Glutamatergic vs. SPNs	173.4	184	-10.53	153	17	0.2615
Glutamatergic vs. Cholinergic	173.4	275.5	-102.1	153	8	1.786
Glutamatergic vs. DAergic (not MB)	173.4	293.7	-120.3	153	5	1.679
Glutamatergic vs. DAergic (MB)	173.4	115.1	58.33	153	9	1.079
GABAergic (not PV) vs. GABAergic (PV)	372.1	359.7	12.45	89	34	0.392
GABAergic (not PV) vs. SPNs	372.1	184	188.2	89	17	4.512
GABAergic (not PV) vs. Cholinergic	372.1	275.5	96.63	89	8	1.662

GABAergic (not PV) vs. DAergic (not MB)	372.1	293.7	78.43	89	5	1.083
GABAergic (not PV) vs. DAergic (MB)	372.1	115.1	257	89	9	4.663
GABAergic (PV) vs. SPNs	359.7	184	175.7	34	17	3.754
GABAergic (PV) vs. Cholinergic	359.7	275.5	84.18	34	8	1.36
GABAergic (PV) vs. DAergic (not MB)	359.7	293.7	65.98	34	5	0.8742
GABAergic (PV) vs. DAergic (MB)	359.7	115.1	244.6	34	9	4.141
SPNs vs. Cholinergic	184	275.5	-91.53	17	8	1.355
SPNs vs. DAergic (not MB)	184	293.7	-109.7	17	5	1.369
SPNs vs. DAergic (MB)	184	115.1	68.86	17	9	1.06
Cholinergic vs. DAergic (not MB)	275.5	293.7	-18.2	8	5	0.2026
Cholinergic vs. DAergic (MB)	275.5	115.1	160.4	8	9	2.095
DAergic (not MB) vs. DAergic (MB)	293.7	115.1	178.6	5	9	2.032

Mafb-neuron

Number of families	1
Number of comparisons per family	28
Alpha	0.05

Dunn's multiple comparisons test	Mean rank diff.	Significant?	Summary	Adjusted P Value	
Non-neuron vs. Glutamatergic	96.89	Yes	****	<0.0001	A-B
Non-neuron vs. GABAergic (not PV)	-79	Yes	**	0.0015	A-C
Non-neuron vs. GABAergic (PV)	-50.2	No	ns	>0.9999	A-D
Non-neuron vs. SPNs	32.12	No	ns	>0.9999	A-E
Non-neuron vs. Cholinergic	28.2	No	ns	>0.9999	A-F
Non-neuron vs. DAergic (not MB)	-107	No	ns	>0.9999	A-G
Non-neuron vs. DAergic (MB)	106	No	ns	>0.9999	A-H
Glutamatergic vs. GABAergic (not PV)	-175.9	Yes	****	<0.0001	B-C
Glutamatergic vs. GABAergic (PV)	-147.1	Yes	****	<0.0001	B-D
Glutamatergic vs. SPNs	-64.76	No	ns	>0.9999	B-E
Glutamatergic vs. Cholinergic	-68.68	No	ns	>0.9999	B-F
Glutamatergic vs. DAergic (not MB)	-203.9	No	ns	0.1181	B-G
Glutamatergic vs. DAergic (MB)	9.163	No	ns	>0.9999	B-H
GABAergic (not PV) vs. GABAergic (PV)	28.8	No	ns	>0.9999	C-D
GABAergic (not PV) vs. SPNs	111.1	No	ns	0.2073	C-E
GABAergic (not PV) vs. Cholinergic	107.2	No	ns	>0.9999	C-F
GABAergic (not PV) vs. DAergic (not MB)	-27.97	No	ns	>0.9999	C-G
GABAergic (not PV) vs. DAergic (MB)	185.1	Yes	*	0.0207	C-H
GABAergic (PV) vs. SPNs	82.32	No	ns	>0.9999	D-E
GABAergic (PV) vs. Cholinergic	78.4	No	ns	>0.9999	D-F
GABAergic (PV) vs. DAergic (not MB)	-56.77	No	ns	>0.9999	D-G
GABAergic (PV) vs. DAergic (MB)	156.3	No	ns	0.2195	D-H
SPNs vs. Cholinergic	-3.919	No	ns	>0.9999	E-F
SPNs vs. DAergic (not MB)	-139.1	No	ns	>0.9999	E-G
SPNs vs. DAergic (MB)	73.93	No	ns	>0.9999	E-H
Cholinergic vs. DAergic (not MB)	-135.2	No	ns	>0.9999	F-G

Cholinergic vs. DAergic (MB)	77.85	No	ns	>0.9999	F-H
DAergic (not MB) vs. DAergic (MB)	213	No	ns	0.4156	G-H

Test details	Mean rank 1	Mean rank 2	Mean rank diff.	n1	n2	Z
Non-neuron vs. Glutamatergic	286.8	189.9	96.89	231	153	5.929
Non-neuron vs. GABAergic (not PV)	286.8	365.8	-79	231	89	4.04
Non-neuron vs. GABAergic (PV)	286.8	337	-50.2	231	34	1.743
Non-neuron vs. SPNs	286.8	254.7	32.12	231	17	0.8153
Non-neuron vs. Cholinergic	286.8	258.6	28.2	231	8	0.5002
Non-neuron vs. DAergic (not MB)	286.8	393.8	-107	231	5	1.51
Non-neuron vs. DAergic (MB)	286.8	180.8	106	231	9	1.991
Glutamatergic vs. GABAergic (not PV)	189.9	365.8	-175.9	153	89	8.416
Glutamatergic vs. GABAergic (PV)	189.9	337	-147.1	153	34	4.949
Glutamatergic vs. SPNs	189.9	254.7	-64.76	153	17	1.616
Glutamatergic vs. Cholinergic	189.9	258.6	-68.68	153	8	1.208
Glutamatergic vs. DAergic (not MB)	189.9	393.8	-203.9	153	5	2.861
Glutamatergic vs. DAergic (MB)	189.9	180.8	9.163	153	9	0.1704
GABAergic (not PV) vs. GABAergic (PV)	365.8	337	28.8	89	34	0.9113
GABAergic (not PV) vs. SPNs	365.8	254.7	111.1	89	17	2.678
GABAergic (not PV) vs. Cholinergic	365.8	258.6	107.2	89	8	1.853
GABAergic (not PV) vs. DAergic (not MB)	365.8	393.8	-27.97	89	5	0.3882
GABAergic (not PV) vs. DAergic (MB)	365.8	180.8	185.1	89	9	3.375
GABAergic (PV) vs. SPNs	337	254.7	82.32	34	17	1.768
GABAergic (PV) vs. Cholinergic	337	258.6	78.4	34	8	1.273
GABAergic (PV) vs. DAergic (not MB)	337	393.8	-56.77	34	5	0.7561
GABAergic (PV) vs. DAergic (MB)	337	180.8	156.3	34	9	2.659
SPNs vs. Cholinergic	254.7	258.6	-3.919	17	8	0.05831
SPNs vs. DAergic (not MB)	254.7	393.8	-139.1	17	5	1.744
SPNs vs. DAergic (MB)	254.7	180.8	73.93	17	9	1.144
Cholinergic vs. DAergic (not MB)	258.6	393.8	-135.2	8	5	1.513
Cholinergic vs. DAergic (MB)	258.6	180.8	77.85	8	9	1.022
DAergic (not MB) vs. DAergic (MB)	393.8	180.8	213	5	9	2.436

Maff-neuron

Number of families	1
Number of comparisons per family	28
Alpha	0.05

Dunn's multiple comparisons test	Mean rank diff.	Significant?	Summary	Adjusted P Value	
Non-neuron vs. Glutamatergic	48.26	Yes	*	0.0303	A-B
Non-neuron vs. GABAergic (not PV)	31.2	No	ns	>0.9999	A-C
Non-neuron vs. GABAergic (PV)	53.13	No	ns	>0.9999	A-D
Non-neuron vs. SPNs	92.43	No	ns	0.2637	A-E
Non-neuron vs. Cholinergic	-30.92	No	ns	>0.9999	A-F
Non-neuron vs. DAergic (not MB)	141.9	No	ns	0.7471	A-G

Non-neuron vs. DAergic (MB)	76.12	No	ns	>0.9999	A-H
Glutamatergic vs. GABAergic (not PV)	-17.06	No	ns	>0.9999	B-C
Glutamatergic vs. GABAergic (PV)	4.873	No	ns	>0.9999	B-D
Glutamatergic vs. SPNs	44.17	No	ns	>0.9999	B-E
Glutamatergic vs. Cholinergic	-79.18	No	ns	>0.9999	B-F
Glutamatergic vs. DAergic (not MB)	93.64	No	ns	>0.9999	B-G
Glutamatergic vs. DAergic (MB)	27.86	No	ns	>0.9999	B-H
GABAergic (not PV) vs. GABAergic (PV)	21.93	No	ns	>0.9999	C-D
GABAergic (not PV) vs. SPNs	61.23	No	ns	>0.9999	C-E
GABAergic (not PV) vs. Cholinergic	-62.12	No	ns	>0.9999	C-F
GABAergic (not PV) vs. DAergic (not MB)	110.7	No	ns	>0.9999	C-G
GABAergic (not PV) vs. DAergic (MB)	44.92	No	ns	>0.9999	C-H
GABAergic (PV) vs. SPNs	39.29	No	ns	>0.9999	D-E
GABAergic (PV) vs. Cholinergic	-84.05	No	ns	>0.9999	D-F
GABAergic (PV) vs. DAergic (not MB)	88.76	No	ns	>0.9999	D-G
GABAergic (PV) vs. DAergic (MB)	22.99	No	ns	>0.9999	D-H
SPNs vs. Cholinergic	-123.3	No	ns	>0.9999	E-F
SPNs vs. DAergic (not MB)	49.47	No	ns	>0.9999	E-G
SPNs vs. DAergic (MB)	-16.31	No	ns	>0.9999	E-H
Cholinergic vs. DAergic (not MB)	172.8	No	ns	0.9058	F-G
Cholinergic vs. DAergic (MB)	107	No	ns	>0.9999	F-H
DAergic (not MB) vs. DAergic (MB)	-65.78	No	ns	>0.9999	G-H

Test details	Mean rank 1	Mean rank 2	Mean rank diff.	n1	n2	Z
Non-neuron vs. Glutamatergic	300.4	252.1	48.26	231	153	3.269
Non-neuron vs. GABAergic (not PV)	300.4	269.2	31.2	231	89	1.766
Non-neuron vs. GABAergic (PV)	300.4	247.3	53.13	231	34	2.042
Non-neuron vs. SPNs	300.4	208	92.43	231	17	2.597
Non-neuron vs. Cholinergic	300.4	331.3	-30.92	231	8	0.6069
Non-neuron vs. DAergic (not MB)	300.4	158.5	141.9	231	5	2.216
Non-neuron vs. DAergic (MB)	300.4	224.3	76.12	231	9	1.582
Glutamatergic vs. GABAergic (not PV)	252.1	269.2	-17.06	153	89	0.9034
Glutamatergic vs. GABAergic (PV)	252.1	247.3	4.873	153	34	0.1814
Glutamatergic vs. SPNs	252.1	208	44.17	153	17	1.22
Glutamatergic vs. Cholinergic	252.1	331.3	-79.18	153	8	1.541
Glutamatergic vs. DAergic (not MB)	252.1	158.5	93.64	153	5	1.455
Glutamatergic vs. DAergic (MB)	252.1	224.3	27.86	153	9	0.5734
GABAergic (not PV) vs. GABAergic (PV)	269.2	247.3	21.93	89	34	0.768
GABAergic (not PV) vs. SPNs	269.2	208	61.23	89	17	1.633
GABAergic (not PV) vs. Cholinergic	269.2	331.3	-62.12	89	8	1.188
GABAergic (not PV) vs. DAergic (not MB)	269.2	158.5	110.7	89	5	1.7
GABAergic (not PV) vs. DAergic (MB)	269.2	224.3	44.92	89	9	0.9066
GABAergic (PV) vs. SPNs	247.3	208	39.29	34	17	0.9339
GABAergic (PV) vs. Cholinergic	247.3	331.3	-84.05	34	8	1.51
GABAergic (PV) vs. DAergic (not MB)	247.3	158.5	88.76	34	5	1.308
GABAergic (PV) vs. DAergic (MB)	247.3	224.3	22.99	34	9	0.4329

SPNs vs. Cholinergic	208	331.3	-123.3	17	8	2.031
SPNs vs. DAergic (not MB)	208	158.5	49.47	17	5	0.6865
SPNs vs. DAergic (MB)	208	224.3	-16.31	17	9	0.2793
Cholinergic vs. DAergic (not MB)	331.3	158.5	172.8	8	5	2.14
Cholinergic vs. DAergic (MB)	331.3	224.3	107	8	9	1.555
DAergic (not MB) vs. DAergic (MB)	158.5	224.3	-65.78	5	9	0.8326

Maff-neuron

Number of families	1
Number of comparisons per family	28
Alpha	0.05

Dunn's multiple comparisons test	Mean rank diff.	Significant?	Summary	Adjusted P Value	
Non-neuron vs. Glutamatergic	48.26	Yes	*	0.0303	A-B
Non-neuron vs. GABAergic (not PV)	31.2	No	ns	>0.9999	A-C
Non-neuron vs. GABAergic (PV)	53.13	No	ns	>0.9999	A-D
Non-neuron vs. SPNs	92.43	No	ns	0.2637	A-E
Non-neuron vs. Cholinergic	-30.92	No	ns	>0.9999	A-F
Non-neuron vs. DAergic (not MB)	141.9	No	ns	0.7471	A-G
Non-neuron vs. DAergic (MB)	76.12	No	ns	>0.9999	A-H
Glutamatergic vs. GABAergic (not PV)	-17.06	No	ns	>0.9999	B-C
Glutamatergic vs. GABAergic (PV)	4.873	No	ns	>0.9999	B-D
Glutamatergic vs. SPNs	44.17	No	ns	>0.9999	B-E
Glutamatergic vs. Cholinergic	-79.18	No	ns	>0.9999	B-F
Glutamatergic vs. DAergic (not MB)	93.64	No	ns	>0.9999	B-G
Glutamatergic vs. DAergic (MB)	27.86	No	ns	>0.9999	B-H
GABAergic (not PV) vs. GABAergic (PV)	21.93	No	ns	>0.9999	C-D
GABAergic (not PV) vs. SPNs	61.23	No	ns	>0.9999	C-E
GABAergic (not PV) vs. Cholinergic	-62.12	No	ns	>0.9999	C-F
GABAergic (not PV) vs. DAergic (not MB)	110.7	No	ns	>0.9999	C-G
GABAergic (not PV) vs. DAergic (MB)	44.92	No	ns	>0.9999	C-H
GABAergic (PV) vs. SPNs	39.29	No	ns	>0.9999	D-E
GABAergic (PV) vs. Cholinergic	-84.05	No	ns	>0.9999	D-F
GABAergic (PV) vs. DAergic (not MB)	88.76	No	ns	>0.9999	D-G
GABAergic (PV) vs. DAergic (MB)	22.99	No	ns	>0.9999	D-H
SPNs vs. Cholinergic	-123.3	No	ns	>0.9999	E-F
SPNs vs. DAergic (not MB)	49.47	No	ns	>0.9999	E-G
SPNs vs. DAergic (MB)	-16.31	No	ns	>0.9999	E-H
Cholinergic vs. DAergic (not MB)	172.8	No	ns	0.9058	F-G
Cholinergic vs. DAergic (MB)	107	No	ns	>0.9999	F-H
DAergic (not MB) vs. DAergic (MB)	-65.78	No	ns	>0.9999	G-H

Test details	Mean rank 1	Mean rank 2	Mean rank diff.	n1	n2	Z
Non-neuron vs. Glutamatergic	300.4	252.1	48.26	231	153	3.269
Non-neuron vs. GABAergic (not PV)	300.4	269.2	31.2	231	89	1.766

Non-neuron vs. GABAergic (PV)	300.4	247.3	53.13	231	34	2.042
Non-neuron vs. SPNs	300.4	208	92.43	231	17	2.597
Non-neuron vs. Cholinergic	300.4	331.3	-30.92	231	8	0.6069
Non-neuron vs. DAergic (not MB)	300.4	158.5	141.9	231	5	2.216
Non-neuron vs. DAergic (MB)	300.4	224.3	76.12	231	9	1.582
Glutamatergic vs. GABAergic (not PV)	252.1	269.2	-17.06	153	89	0.9034
Glutamatergic vs. GABAergic (PV)	252.1	247.3	4.873	153	34	0.1814
Glutamatergic vs. SPNs	252.1	208	44.17	153	17	1.22
Glutamatergic vs. Cholinergic	252.1	331.3	-79.18	153	8	1.541
Glutamatergic vs. DAergic (not MB)	252.1	158.5	93.64	153	5	1.455
Glutamatergic vs. DAergic (MB)	252.1	224.3	27.86	153	9	0.5734
GABAergic (not PV) vs. GABAergic (PV)	269.2	247.3	21.93	89	34	0.768
GABAergic (not PV) vs. SPNs	269.2	208	61.23	89	17	1.633
GABAergic (not PV) vs. Cholinergic	269.2	331.3	-62.12	89	8	1.188
GABAergic (not PV) vs. DAergic (not MB)	269.2	158.5	110.7	89	5	1.7
GABAergic (not PV) vs. DAergic (MB)	269.2	224.3	44.92	89	9	0.9066
GABAergic (PV) vs. SPNs	247.3	208	39.29	34	17	0.9339
GABAergic (PV) vs. Cholinergic	247.3	331.3	-84.05	34	8	1.51
GABAergic (PV) vs. DAergic (not MB)	247.3	158.5	88.76	34	5	1.308
GABAergic (PV) vs. DAergic (MB)	247.3	224.3	22.99	34	9	0.4329
SPNs vs. Cholinergic	208	331.3	-123.3	17	8	2.031
SPNs vs. DAergic (not MB)	208	158.5	49.47	17	5	0.6865
SPNs vs. DAergic (MB)	208	224.3	-16.31	17	9	0.2793
Cholinergic vs. DAergic (not MB)	331.3	158.5	172.8	8	5	2.14
Cholinergic vs. DAergic (MB)	331.3	224.3	107	8	9	1.555
DAergic (not MB) vs. DAergic (MB)	158.5	224.3	-65.78	5	9	0.8326

Mafg-neuron

Number of families	1
Number of comparisons per family	28
Alpha	0.05

Dunn's multiple comparisons test	Mean rank diff.	Significant?	Summary	Adjusted P Value	
Non-neuron vs. Glutamatergic	-148.3	Yes	****	<0.0001	A-B
Non-neuron vs. GABAergic (not PV)	-241.5	Yes	****	<0.0001	A-C
Non-neuron vs. GABAergic (PV)	-250.6	Yes	****	<0.0001	A-D
Non-neuron vs. SPNs	-49.16	No	ns	>0.9999	A-E
Non-neuron vs. Cholinergic	-252	Yes	***	0.0002	A-F
Non-neuron vs. DAergic (not MB)	-181.2	No	ns	0.3092	A-G
Non-neuron vs. DAergic (MB)	-346.1	Yes	****	<0.0001	A-H
Glutamatergic vs. GABAergic (not PV)	-93.19	Yes	***	0.0003	B-C
Glutamatergic vs. GABAergic (PV)	-102.3	Yes	*	0.0176	B-D
Glutamatergic vs. SPNs	99.18	No	ns	0.3901	B-E
Glutamatergic vs. Cholinergic	-103.7	No	ns	>0.9999	B-F
Glutamatergic vs. DAergic (not MB)	-32.89	No	ns	>0.9999	B-G
Glutamatergic vs. DAergic (MB)	-197.7	Yes	**	0.0072	B-H
GABAergic (not PV) vs. GABAergic (PV)	-9.072	No	ns	>0.9999	C-D

GABAergic (not PV) vs. SPNs	192.4	Yes	***		0.0001	C-E
GABAergic (not PV) vs. Cholinergic	-10.48	No	ns	>0.9999		C-F
GABAergic (not PV) vs. DAergic (not MB)	60.3	No	ns	>0.9999		C-G
GABAergic (not PV) vs. DAergic (MB)	-104.5	No	ns	>0.9999		C-H
GABAergic (PV) vs. SPNs	201.4	Yes	***		0.0005	D-E
GABAergic (PV) vs. Cholinergic	-1.404	No	ns	>0.9999		D-F
GABAergic (PV) vs. DAergic (not MB)	69.37	No	ns	>0.9999		D-G
GABAergic (PV) vs. DAergic (MB)	-95.47	No	ns	>0.9999		D-H
SPNs vs. Cholinergic	-202.8	No	ns		0.0759	E-F
SPNs vs. DAergic (not MB)	-132.1	No	ns	>0.9999		E-G
SPNs vs. DAergic (MB)	-296.9	Yes	***		0.0001	E-H
Cholinergic vs. DAergic (not MB)	70.78	No	ns	>0.9999		F-G
Cholinergic vs. DAergic (MB)	-94.07	No	ns	>0.9999		F-H
DAergic (not MB) vs. DAergic (MB)	-164.8	No	ns	>0.9999		G-H

Test details	Mean rank 1	Mean rank 2	Mean rank diff.	n1	n2	Z
Non-neuron vs. Glutamatergic	164.4	312.7	-148.3	231	153	9.02
Non-neuron vs. GABAergic (not PV)	164.4	405.9	-241.5	231	89	12.27
Non-neuron vs. GABAergic (PV)	164.4	415	-250.6	231	34	8.648
Non-neuron vs. SPNs	164.4	213.5	-49.16	231	17	1.24
Non-neuron vs. Cholinergic	164.4	416.4	-252	231	8	4.442
Non-neuron vs. DAergic (not MB)	164.4	345.6	-181.2	231	5	2.541
Non-neuron vs. DAergic (MB)	164.4	510.4	-346.1	231	9	6.456
Glutamatergic vs. GABAergic (not PV)	312.7	405.9	-93.19	153	89	4.431
Glutamatergic vs. GABAergic (PV)	312.7	415	-102.3	153	34	3.419
Glutamatergic vs. SPNs	312.7	213.5	99.18	153	17	2.459
Glutamatergic vs. Cholinergic	312.7	416.4	-103.7	153	8	1.812
Glutamatergic vs. DAergic (not MB)	312.7	345.6	-32.89	153	5	0.4588
Glutamatergic vs. DAergic (MB)	312.7	510.4	-197.7	153	9	3.654
GABAergic (not PV) vs. GABAergic (PV)	405.9	415	-9.072	89	34	0.2852
GABAergic (not PV) vs. SPNs	405.9	213.5	192.4	89	17	4.607
GABAergic (not PV) vs. Cholinergic	405.9	416.4	-10.48	89	8	0.1799
GABAergic (not PV) vs. DAergic (not MB)	405.9	345.6	60.3	89	5	0.8316
GABAergic (not PV) vs. DAergic (MB)	405.9	510.4	-104.5	89	9	1.895
GABAergic (PV) vs. SPNs	415	213.5	201.4	34	17	4.299
GABAergic (PV) vs. Cholinergic	415	416.4	-1.404	34	8	0.02265
GABAergic (PV) vs. DAergic (not MB)	415	345.6	69.37	34	5	0.9181
GABAergic (PV) vs. DAergic (MB)	415	510.4	-95.47	34	9	1.614
SPNs vs. Cholinergic	213.5	416.4	-202.8	17	8	2.999
SPNs vs. DAergic (not MB)	213.5	345.6	-132.1	17	5	1.646
SPNs vs. DAergic (MB)	213.5	510.4	-296.9	17	9	4.566
Cholinergic vs. DAergic (not MB)	416.4	345.6	70.78	8	5	0.7869
Cholinergic vs. DAergic (MB)	416.4	510.4	-94.07	8	9	1.227
DAergic (not MB) vs. DAergic (MB)	345.6	510.4	-164.8	5	9	1.873

Mafk-neuron

Number of families	1
Number of comparisons per family	28
Alpha	0.05

Dunn's multiple comparisons test	Mean rank diff.	Significant?	Summary	Adjusted P Value	
Non-neuron vs. Glutamatergic	54.64	Yes	*	0.0237	A-B
Non-neuron vs. GABAergic (not PV)	105.3	Yes	****	<0.0001	A-C
Non-neuron vs. GABAergic (PV)	123	Yes	***	0.0006	A-D
Non-neuron vs. SPNs	137.3	Yes	*	0.0142	A-E
Non-neuron vs. Cholinergic	110.1	No	ns	>0.9999	A-F
Non-neuron vs. DAergic (not MB)	177.7	No	ns	0.3448	A-G
Non-neuron vs. DAergic (MB)	73.12	No	ns	>0.9999	A-H
Glutamatergic vs. GABAergic (not PV)	50.62	No	ns	0.4383	B-C
Glutamatergic vs. GABAergic (PV)	68.32	No	ns	0.6104	B-D
Glutamatergic vs. SPNs	82.65	No	ns	>0.9999	B-E
Glutamatergic vs. Cholinergic	55.46	No	ns	>0.9999	B-F
Glutamatergic vs. DAergic (not MB)	123.1	No	ns	>0.9999	B-G
Glutamatergic vs. DAergic (MB)	18.48	No	ns	>0.9999	B-H
GABAergic (not PV) vs. GABAergic (PV)	17.71	No	ns	>0.9999	C-D
GABAergic (not PV) vs. SPNs	32.03	No	ns	>0.9999	C-E
GABAergic (not PV) vs. Cholinergic	4.84	No	ns	>0.9999	C-F
GABAergic (not PV) vs. DAergic (not MB)	72.49	No	ns	>0.9999	C-G
GABAergic (not PV) vs. DAergic (MB)	-32.13	No	ns	>0.9999	C-H
GABAergic (PV) vs. SPNs	14.32	No	ns	>0.9999	D-E
GABAergic (PV) vs. Cholinergic	-12.87	No	ns	>0.9999	D-F
GABAergic (PV) vs. DAergic (not MB)	54.78	No	ns	>0.9999	D-G
GABAergic (PV) vs. DAergic (MB)	-49.84	No	ns	>0.9999	D-H
SPNs vs. Cholinergic	-27.19	No	ns	>0.9999	E-F
SPNs vs. DAergic (not MB)	40.46	No	ns	>0.9999	E-G
SPNs vs. DAergic (MB)	-64.16	No	ns	>0.9999	E-H
Cholinergic vs. DAergic (not MB)	67.65	No	ns	>0.9999	F-G
Cholinergic vs. DAergic (MB)	-36.97	No	ns	>0.9999	F-H
DAergic (not MB) vs. DAergic (MB)	-104.6	No	ns	>0.9999	G-H

Test details	Mean rank 1	Mean rank 2	Mean rank diff.	n1	n2	Z
Non-neuron vs. Glutamatergic	322.3	267.7	54.64	231	153	3.337
Non-neuron vs. GABAergic (not PV)	322.3	217.1	105.3	231	89	5.37
Non-neuron vs. GABAergic (PV)	322.3	199.4	123	231	34	4.261
Non-neuron vs. SPNs	322.3	185.1	137.3	231	17	3.478
Non-neuron vs. Cholinergic	322.3	212.3	110.1	231	8	1.949
Non-neuron vs. DAergic (not MB)	322.3	144.6	177.7	231	5	2.503
Non-neuron vs. DAergic (MB)	322.3	249.2	73.12	231	9	1.37
Glutamatergic vs. GABAergic (not PV)	267.7	217.1	50.62	153	89	2.417
Glutamatergic vs. GABAergic (PV)	267.7	199.4	68.32	153	34	2.294

Glutamatergic vs. SPNs	267.7	185.1	82.65	153	17	2.058
Glutamatergic vs. Cholinergic	267.7	212.3	55.46	153	8	0.9733
Glutamatergic vs. DAergic (not MB)	267.7	144.6	123.1	153	5	1.724
Glutamatergic vs. DAergic (MB)	267.7	249.2	18.48	153	9	0.343
GABAergic (not PV) vs. GABAergic (PV)	217.1	199.4	17.71	89	34	0.5591
GABAergic (not PV) vs. SPNs	217.1	185.1	32.03	89	17	0.7703
GABAergic (not PV) vs. Cholinergic	217.1	212.3	4.84	89	8	0.08347
GABAergic (not PV) vs. DAergic (not MB)	217.1	144.6	72.49	89	5	1.004
GABAergic (not PV) vs. DAergic (MB)	217.1	249.2	-32.13	89	9	0.5848
GABAergic (PV) vs. SPNs	199.4	185.1	14.32	34	17	0.3069
GABAergic (PV) vs. Cholinergic	199.4	212.3	-12.87	34	8	0.2084
GABAergic (PV) vs. DAergic (not MB)	199.4	144.6	54.78	34	5	0.7281
GABAergic (PV) vs. DAergic (MB)	199.4	249.2	-49.84	34	9	0.8463
SPNs vs. Cholinergic	185.1	212.3	-27.19	17	8	0.4037
SPNs vs. DAergic (not MB)	185.1	144.6	40.46	17	5	0.5062
SPNs vs. DAergic (MB)	185.1	249.2	-64.16	17	9	0.9908
Cholinergic vs. DAergic (not MB)	212.3	144.6	67.65	8	5	0.7554
Cholinergic vs. DAergic (MB)	212.3	249.2	-36.97	8	9	0.4843
DAergic (not MB) vs. DAergic (MB)	144.6	249.2	-104.6	5	9	1.194

Cul3-neuron

Number of families	1
Number of comparisons per family	28
Alpha	0.05

Dunn's multiple comparisons test	Mean rank diff.	Significant?	Summary	Adjusted P Value	
Non-neuron vs. Glutamatergic	-247.1	Yes	****	<0.0001	A-B
Non-neuron vs. GABAergic (not PV)	-209.8	Yes	****	<0.0001	A-C
Non-neuron vs. GABAergic (PV)	-202.5	Yes	****	<0.0001	A-D
Non-neuron vs. SPNs	-193.9	Yes	****	<0.0001	A-E
Non-neuron vs. Cholinergic	-307.7	Yes	****	<0.0001	A-F
Non-neuron vs. DAergic (not MB)	-267	Yes	**	0.0051	A-G
Non-neuron vs. DAergic (MB)	-102.7	No	ns	>0.9999	A-H
Glutamatergic vs. GABAergic (not PV)	37.3	No	ns	>0.9999	B-C
Glutamatergic vs. GABAergic (PV)	44.6	No	ns	>0.9999	B-D
Glutamatergic vs. SPNs	53.16	No	ns	>0.9999	B-E
Glutamatergic vs. Cholinergic	-60.63	No	ns	>0.9999	B-F
Glutamatergic vs. DAergic (not MB)	-19.93	No	ns	>0.9999	B-G
Glutamatergic vs. DAergic (MB)	144.4	No	ns	0.213	B-H
GABAergic (not PV) vs. GABAergic (PV)	7.308	No	ns	>0.9999	C-D
GABAergic (not PV) vs. SPNs	15.87	No	ns	>0.9999	C-E
GABAergic (not PV) vs. Cholinergic	-97.93	No	ns	>0.9999	C-F
GABAergic (not PV) vs. DAergic (not MB)	-57.23	No	ns	>0.9999	C-G
GABAergic (not PV) vs. DAergic (MB)	107.1	No	ns	>0.9999	C-H
GABAergic (PV) vs. SPNs	8.559	No	ns	>0.9999	D-E

GABAergic (PV) vs. Cholinergic	-105.2	No	ns	>0.9999	D-F
GABAergic (PV) vs. DAergic (not MB)	-64.54	No	ns	>0.9999	D-G
GABAergic (PV) vs. DAergic (MB)	99.82	No	ns	>0.9999	D-H
SPNs vs. Cholinergic	-113.8	No	ns	>0.9999	E-F
SPNs vs. DAergic (not MB)	-73.09	No	ns	>0.9999	E-G
SPNs vs. DAergic (MB)	91.26	No	ns	>0.9999	E-H
Cholinergic vs. DAergic (not MB)	40.7	No	ns	>0.9999	F-G
Cholinergic vs. DAergic (MB)	205.1	No	ns	0.2093	F-H
DAergic (not MB) vs. DAergic (MB)	164.4	No	ns	>0.9999	G-H

Test details	Mean rank 1	Mean rank 2	Mean rank diff.	n1	n2	Z
Non-neuron vs. Glutamatergic	142.8	389.9	-247.1	231	153	15.03
Non-neuron vs. GABAergic (not PV)	142.8	352.6	-209.8	231	89	10.66
Non-neuron vs. GABAergic (PV)	142.8	345.3	-202.5	231	34	6.988
Non-neuron vs. SPNs	142.8	336.7	-193.9	231	17	4.892
Non-neuron vs. Cholinergic	142.8	450.5	-307.7	231	8	5.424
Non-neuron vs. DAergic (not MB)	142.8	409.8	-267	231	5	3.745
Non-neuron vs. DAergic (MB)	142.8	245.4	-102.7	231	9	1.916
Glutamatergic vs. GABAergic (not PV)	389.9	352.6	37.3	153	89	1.773
Glutamatergic vs. GABAergic (PV)	389.9	345.3	44.6	153	34	1.491
Glutamatergic vs. SPNs	389.9	336.7	53.16	153	17	1.318
Glutamatergic vs. Cholinergic	389.9	450.5	-60.63	153	8	1.06
Glutamatergic vs. DAergic (not MB)	389.9	409.8	-19.93	153	5	0.278
Glutamatergic vs. DAergic (MB)	389.9	245.4	144.4	153	9	2.669
GABAergic (not PV) vs. GABAergic (PV)	352.6	345.3	7.308	89	34	0.2298
GABAergic (not PV) vs. SPNs	352.6	336.7	15.87	89	17	0.38
GABAergic (not PV) vs. Cholinergic	352.6	450.5	-97.93	89	8	1.682
GABAergic (not PV) vs. DAergic (not MB)	352.6	409.8	-57.23	89	5	0.7893
GABAergic (not PV) vs. DAergic (MB)	352.6	245.4	107.1	89	9	1.941
GABAergic (PV) vs. SPNs	345.3	336.7	8.559	34	17	0.1826
GABAergic (PV) vs. Cholinergic	345.3	450.5	-105.2	34	8	1.698
GABAergic (PV) vs. DAergic (not MB)	345.3	409.8	-64.54	34	5	0.8541
GABAergic (PV) vs. DAergic (MB)	345.3	245.4	99.82	34	9	1.688
SPNs vs. Cholinergic	336.7	450.5	-113.8	17	8	1.682
SPNs vs. DAergic (not MB)	336.7	409.8	-73.09	17	5	0.9107
SPNs vs. DAergic (MB)	336.7	245.4	91.26	17	9	1.403
Cholinergic vs. DAergic (not MB)	450.5	409.8	40.7	8	5	0.4525
Cholinergic vs. DAergic (MB)	450.5	245.4	205.1	8	9	2.675
DAergic (not MB) vs. DAergic (MB)	409.8	245.4	164.4	5	9	1.868

Nfe2l1-cell

Number of families	1
Number of comparisons per family	55
Alpha	0.05

Dunn's multiple comparisons test	Mean rank diff.	Significant?	Summary	Adjusted P Value	
Neurons vs. Endothelial Cells	-69.98	No	ns	0.7701	A-B
Neurons vs. Fibroblast-like	-20.09	No	ns	>0.9999	A-C
Neurons vs. Mural Cells	137.9	Yes	****	<0.0001	A-D
Neurons vs. Microglia	255	Yes	***	0.0003	A-E
Neurons vs. Oligodendrocytes	290.8	Yes	****	<0.0001	A-F
Neurons vs. Polydendrocytes	265	Yes	****	<0.0001	A-G
Neurons vs. Astrocytes	257.3	Yes	****	<0.0001	A-H
Neurons vs. Macrophages	250.6	Yes	**	0.0018	A-I
Neurons vs. Neurogenesis	296.2	Yes	****	<0.0001	A-J
Neurons vs. Ependyma	225.2	No	ns	0.0848	A-K
Endothelial Cells vs. Fibroblast-like	49.88	No	ns	>0.9999	B-C
Endothelial Cells vs. Mural Cells	207.9	Yes	****	<0.0001	B-D
Endothelial Cells vs. Microglia	325	Yes	****	<0.0001	B-E
Endothelial Cells vs. Oligodendrocytes	360.8	Yes	****	<0.0001	B-F
Endothelial Cells vs. Polydendrocytes	335	Yes	****	<0.0001	B-G
Endothelial Cells vs. Astrocytes	327.2	Yes	****	<0.0001	B-H
Endothelial Cells vs. Macrophages	320.6	Yes	****	<0.0001	B-I
Endothelial Cells vs. Neurogenesis	366.2	Yes	****	<0.0001	B-J
Endothelial Cells vs. Ependyma	295.2	Yes	**	0.0052	B-K
Fibroblast-like vs. Mural Cells	158	Yes	**	0.006	C-D
Fibroblast-like vs. Microglia	275.1	Yes	***	0.001	C-E
Fibroblast-like vs. Oligodendrocytes	310.9	Yes	****	<0.0001	C-F
Fibroblast-like vs. Polydendrocytes	285.1	Yes	****	<0.0001	C-G
Fibroblast-like vs. Astrocytes	277.4	Yes	****	<0.0001	C-H
Fibroblast-like vs. Macrophages	270.7	Yes	**	0.0033	C-I
Fibroblast-like vs. Neurogenesis	316.3	Yes	****	<0.0001	C-J
Fibroblast-like vs. Ependyma	245.3	No	ns	0.0828	C-K
Mural Cells vs. Microglia	117.1	No	ns	>0.9999	D-E
Mural Cells vs. Oligodendrocytes	152.9	Yes	***	0.0008	D-F
Mural Cells vs. Polydendrocytes	127.1	Yes	*	0.0292	D-G
Mural Cells vs. Astrocytes	119.4	No	ns	0.2408	D-H
Mural Cells vs. Macrophages	112.7	No	ns	>0.9999	D-I
Mural Cells vs. Neurogenesis	158.3	No	ns	0.1387	D-J
Mural Cells vs. Ependyma	87.29	No	ns	>0.9999	D-K
Microglia vs. Oligodendrocytes	35.8	No	ns	>0.9999	E-F
Microglia vs. Polydendrocytes	9.939	No	ns	>0.9999	E-G
Microglia vs. Astrocytes	2.228	No	ns	>0.9999	E-H
Microglia vs. Macrophages	-4.393	No	ns	>0.9999	E-I
Microglia vs. Neurogenesis	41.17	No	ns	>0.9999	E-J
Microglia vs. Ependyma	-29.85	No	ns	>0.9999	E-K
Oligodendrocytes vs. Polydendrocytes	-25.86	No	ns	>0.9999	F-G
Oligodendrocytes vs. Astrocytes	-33.57	No	ns	>0.9999	F-H
Oligodendrocytes vs. Macrophages	-40.19	No	ns	>0.9999	F-I
Oligodendrocytes vs. Neurogenesis	5.37	No	ns	>0.9999	F-J

Oligodendrocytes vs. Ependyma	-65.65	No	ns	>0.9999	F-K
Polydendrocytes vs. Astrocytes	-7.711	No	ns	>0.9999	G-H
Polydendrocytes vs. Macrophages	-14.33	No	ns	>0.9999	G-I
Polydendrocytes vs. Neurogenesis	31.23	No	ns	>0.9999	G-J
Polydendrocytes vs. Ependyma	-39.79	No	ns	>0.9999	G-K
Astrocytes vs. Macrophages	-6.621	No	ns	>0.9999	H-I
Astrocytes vs. Neurogenesis	38.94	No	ns	>0.9999	H-J
Astrocytes vs. Ependyma	-32.08	No	ns	>0.9999	H-K
Macrophages vs. Neurogenesis	45.56	No	ns	>0.9999	I-J
Macrophages vs. Ependyma	-25.46	No	ns	>0.9999	I-K
Neurogenesis vs. Ependyma	-71.02	No	ns	>0.9999	J-K

Test details	Mean rank 1	Mean rank 2	Mean rank diff.	n1	n2	Z
Neurons vs. Endothelial Cells	344.8	414.8	-69.98	315	34	2.457
Neurons vs. Fibroblast-like	344.8	364.9	-20.09	315	25	0.6129
Neurons vs. Mural Cells	344.8	206.9	137.9	315	37	5.03
Neurons vs. Microglia	344.8	89.75	255	315	8	4.515
Neurons vs. Oligodendrocytes	344.8	53.95	290.8	315	43	11.34
Neurons vs. Polydendrocytes	344.8	79.81	265	315	37	9.665
Neurons vs. Astrocytes	344.8	87.52	257.3	315	23	7.55
Neurons vs. Macrophages	344.8	94.14	250.6	315	7	4.158
Neurons vs. Neurogenesis	344.8	48.58	296.2	315	12	6.384
Neurons vs. Ependyma	344.8	119.6	225.2	315	5	3.167
Endothelial Cells vs. Fibroblast-like	414.8	364.9	49.88	34	25	1.2
Endothelial Cells vs. Mural Cells	414.8	206.9	207.9	34	37	5.546
Endothelial Cells vs. Microglia	414.8	89.75	325	34	8	5.243
Endothelial Cells vs. Oligodendrocytes	414.8	53.95	360.8	34	43	9.966
Endothelial Cells vs. Polydendrocytes	414.8	79.81	335	34	37	8.937
Endothelial Cells vs. Astrocytes	414.8	87.52	327.2	34	23	7.683
Endothelial Cells vs. Macrophages	414.8	94.14	320.6	34	7	4.897
Endothelial Cells vs. Neurogenesis	414.8	48.58	366.2	34	12	6.913
Endothelial Cells vs. Ependyma	414.8	119.6	295.2	34	5	3.906
Fibroblast-like vs. Mural Cells	364.9	206.9	158	25	37	3.868
Fibroblast-like vs. Microglia	364.9	89.75	275.1	25	8	4.293
Fibroblast-like vs. Oligodendrocytes	364.9	53.95	310.9	25	43	7.836
Fibroblast-like vs. Polydendrocytes	364.9	79.81	285.1	25	37	6.98
Fibroblast-like vs. Astrocytes	364.9	87.52	277.4	25	23	6.085
Fibroblast-like vs. Macrophages	364.9	94.14	270.7	25	7	4.013
Fibroblast-like vs. Neurogenesis	364.9	48.58	316.3	25	12	5.709
Fibroblast-like vs. Ependyma	364.9	119.6	245.3	25	5	3.174
Mural Cells vs. Microglia	206.9	89.75	117.1	37	8	1.904
Mural Cells vs. Oligodendrocytes	206.9	53.95	152.9	37	43	4.323
Mural Cells vs. Polydendrocytes	206.9	79.81	127.1	37	37	3.465
Mural Cells vs. Astrocytes	206.9	87.52	119.4	37	23	2.85
Mural Cells vs. Macrophages	206.9	94.14	112.7	37	7	1.734
Mural Cells vs. Neurogenesis	206.9	48.58	158.3	37	12	3.021

Mural Cells vs. Ependyma	206.9	119.6	87.29	37	5	1.161
Microglia vs. Oligodendrocytes	89.75	53.95	35.8	8	43	0.5893
Microglia vs. Polydendrocytes	89.75	79.81	9.939	8	37	0.1616
Microglia vs. Astrocytes	89.75	87.52	2.228	8	23	0.03441
Microglia vs. Macrophages	89.75	94.14	-4.393	8	7	0.0538
Microglia vs. Neurogenesis	89.75	48.58	41.17	8	12	0.5717
Microglia vs. Ependyma	89.75	119.6	-29.85	8	5	0.3319
Oligodendrocytes vs. Polydendrocytes	53.95	79.81	-25.86	43	37	0.7309
Oligodendrocytes vs. Astrocytes	53.95	87.52	-33.57	43	23	0.8237
Oligodendrocytes vs. Macrophages	53.95	94.14	-40.19	43	7	0.625
Oligodendrocytes vs. Neurogenesis	53.95	48.58	5.37	43	12	0.1043
Oligodendrocytes vs. Ependyma	53.95	119.6	-65.65	43	5	0.8807
Polydendrocytes vs. Astrocytes	79.81	87.52	-7.711	37	23	0.1841
Polydendrocytes vs. Macrophages	79.81	94.14	-14.33	37	7	0.2204
Polydendrocytes vs. Neurogenesis	79.81	48.58	31.23	37	12	0.5958
Polydendrocytes vs. Ependyma	79.81	119.6	-39.79	37	5	0.5293
Astrocytes vs. Macrophages	87.52	94.14	-6.621	23	7	0.09723
Astrocytes vs. Neurogenesis	87.52	48.58	38.94	23	12	0.6931
Astrocytes vs. Ependyma	87.52	119.6	-32.08	23	5	0.4121
Macrophages vs. Neurogenesis	94.14	48.58	45.56	7	12	0.6072
Macrophages vs. Ependyma	94.14	119.6	-25.46	7	5	0.2756
Neurogenesis vs. Ependyma	48.58	119.6	-71.02	12	5	0.8457

Nfe2l2-cell

Number of families	1
Number of comparisons per family	55
Alpha	0.05

Dunn's multiple comparisons test	Mean rank diff.	Significant?	Summary	Adjusted P Value	
Neurons vs. Endothelial Cells	-330.8	Yes	****	<0.0001	A-B
Neurons vs. Fibroblast-like	-327.2	Yes	****	<0.0001	A-C
Neurons vs. Mural Cells	-219.2	Yes	****	<0.0001	A-D
Neurons vs. Microglia	-362.4	Yes	****	<0.0001	A-E
Neurons vs. Oligodendrocytes	-235.4	Yes	****	<0.0001	A-F
Neurons vs. Polydendrocytes	-181.9	Yes	****	<0.0001	A-G
Neurons vs. Astrocytes	-319.8	Yes	****	<0.0001	A-H
Neurons vs. Macrophages	-371.1	Yes	****	<0.0001	A-I
Neurons vs. Neurogenesis	-207.2	Yes	***	0.0004	A-J
Neurons vs. Ependyma	-241.7	Yes	*	0.0341	A-K
Endothelial Cells vs. Fibroblast-like	3.567	No	ns	>0.9999	B-C
Endothelial Cells vs. Mural Cells	111.5	No	ns	0.1501	B-D
Endothelial Cells vs. Microglia	-31.6	No	ns	>0.9999	B-E
Endothelial Cells vs. Oligodendrocytes	95.37	No	ns	0.4395	B-F
Endothelial Cells vs. Polydendrocytes	148.8	Yes	**	0.0035	B-G
Endothelial Cells vs. Astrocytes	10.91	No	ns	>0.9999	B-H

Endothelial Cells vs. Macrophages	-40.35	No	ns	>0.9999	B-I
Endothelial Cells vs. Neurogenesis	123.6	No	ns	>0.9999	B-J
Endothelial Cells vs. Ependyma	89.05	No	ns	>0.9999	B-K
Fibroblast-like vs. Mural Cells	108	No	ns	0.4272	C-D
Fibroblast-like vs. Microglia	-35.17	No	ns	>0.9999	C-E
Fibroblast-like vs. Oligodendrocytes	91.8	No	ns	>0.9999	C-F
Fibroblast-like vs. Polydendrocytes	145.2	Yes	*	0.0188	C-G
Fibroblast-like vs. Astrocytes	7.341	No	ns	>0.9999	C-H
Fibroblast-like vs. Macrophages	-43.92	No	ns	>0.9999	C-I
Fibroblast-like vs. Neurogenesis	120	No	ns	>0.9999	C-J
Fibroblast-like vs. Ependyma	85.48	No	ns	>0.9999	C-K
Mural Cells vs. Microglia	-143.1	No	ns	>0.9999	D-E
Mural Cells vs. Oligodendrocytes	-16.17	No	ns	>0.9999	D-F
Mural Cells vs. Polydendrocytes	37.27	No	ns	>0.9999	D-G
Mural Cells vs. Astrocytes	-100.6	No	ns	0.8559	D-H
Mural Cells vs. Macrophages	-151.9	No	ns	>0.9999	D-I
Mural Cells vs. Neurogenesis	12.02	No	ns	>0.9999	D-J
Mural Cells vs. Ependyma	-22.49	No	ns	>0.9999	D-K
Microglia vs. Oligodendrocytes	127	No	ns	>0.9999	E-F
Microglia vs. Polydendrocytes	180.4	No	ns	0.1728	E-G
Microglia vs. Astrocytes	42.51	No	ns	>0.9999	E-H
Microglia vs. Macrophages	-8.75	No	ns	>0.9999	E-I
Microglia vs. Neurogenesis	155.2	No	ns	>0.9999	E-J
Microglia vs. Ependyma	120.7	No	ns	>0.9999	E-K
Oligodendrocytes vs. Polydendrocytes	53.44	No	ns	>0.9999	F-G
Oligodendrocytes vs. Astrocytes	-84.46	No	ns	>0.9999	F-H
Oligodendrocytes vs. Macrophages	-135.7	No	ns	>0.9999	F-I
Oligodendrocytes vs. Neurogenesis	28.2	No	ns	>0.9999	F-J
Oligodendrocytes vs. Ependyma	-6.321	No	ns	>0.9999	F-K
Polydendrocytes vs. Astrocytes	-137.9	No	ns	0.0504	G-H
Polydendrocytes vs. Macrophages	-189.2	No	ns	0.1868	G-I
Polydendrocytes vs. Neurogenesis	-25.25	No	ns	>0.9999	G-J
Polydendrocytes vs. Ependyma	-59.76	No	ns	>0.9999	G-K
Astrocytes vs. Macrophages	-51.26	No	ns	>0.9999	H-I
Astrocytes vs. Neurogenesis	112.7	No	ns	>0.9999	H-J
Astrocytes vs. Ependyma	78.14	No	ns	>0.9999	H-K
Macrophages vs. Neurogenesis	163.9	No	ns	>0.9999	I-J
Macrophages vs. Ependyma	129.4	No	ns	>0.9999	I-K
Neurogenesis vs. Ependyma	-34.52	No	ns	>0.9999	J-K

Test details	Mean rank 1	Mean rank 2	Mean rank diff.	n1	n2	Z
Neurons vs. Endothelial Cells	161.9	492.6	-330.8	315	34	11.7
Neurons vs. Fibroblast-like	161.9	489.1	-327.2	315	25	10.05
Neurons vs. Mural Cells	161.9	381.1	-219.2	315	37	8.052
Neurons vs. Microglia	161.9	524.3	-362.4	315	8	6.46
Neurons vs. Oligodendrocytes	161.9	397.3	-235.4	315	43	9.242

Neurons vs. Polydendrocytes	161.9	343.8	-181.9	315	37	6.683
Neurons vs. Astrocytes	161.9	481.7	-319.8	315	23	9.452
Neurons vs. Macrophages	161.9	533	-371.1	315	7	6.199
Neurons vs. Neurogenesis	161.9	369.1	-207.2	315	12	4.496
Neurons vs. Ependyma	161.9	403.6	-241.7	315	5	3.423
Endothelial Cells vs. Fibroblast-like	492.6	489.1	3.567	34	25	0.08642
Endothelial Cells vs. Mural Cells	492.6	381.1	111.5	34	37	2.997
Endothelial Cells vs. Microglia	492.6	524.3	-31.6	34	8	0.5133
Endothelial Cells vs. Oligodendrocytes	492.6	397.3	95.37	34	43	2.652
Endothelial Cells vs. Polydendrocytes	492.6	343.8	148.8	34	37	3.998
Endothelial Cells vs. Astrocytes	492.6	481.7	10.91	34	23	0.2579
Endothelial Cells vs. Macrophages	492.6	533	-40.35	34	7	0.6206
Endothelial Cells vs. Neurogenesis	492.6	369.1	123.6	34	12	2.349
Endothelial Cells vs. Ependyma	492.6	403.6	89.05	34	5	1.187
Fibroblast-like vs. Mural Cells	489.1	381.1	108	25	37	2.662
Fibroblast-like vs. Microglia	489.1	524.3	-35.17	25	8	0.5527
Fibroblast-like vs. Oligodendrocytes	489.1	397.3	91.8	25	43	2.33
Fibroblast-like vs. Polydendrocytes	489.1	343.8	145.2	25	37	3.581
Fibroblast-like vs. Astrocytes	489.1	481.7	7.341	25	23	0.1622
Fibroblast-like vs. Macrophages	489.1	533	-43.92	25	7	0.6556
Fibroblast-like vs. Neurogenesis	489.1	369.1	120	25	12	2.181
Fibroblast-like vs. Ependyma	489.1	403.6	85.48	25	5	1.114
Mural Cells vs. Microglia	381.1	524.3	-143.1	37	8	2.343
Mural Cells vs. Oligodendrocytes	381.1	397.3	-16.17	37	43	0.4603
Mural Cells vs. Polydendrocytes	381.1	343.8	37.27	37	37	1.023
Mural Cells vs. Astrocytes	381.1	481.7	-100.6	37	23	2.419
Mural Cells vs. Macrophages	381.1	533	-151.9	37	7	2.352
Mural Cells vs. Neurogenesis	381.1	369.1	12.02	37	12	0.231
Mural Cells vs. Ependyma	381.1	403.6	-22.49	37	5	0.3013
Microglia vs. Oligodendrocytes	524.3	397.3	127	8	43	2.105
Microglia vs. Polydendrocytes	524.3	343.8	180.4	8	37	2.953
Microglia vs. Astrocytes	524.3	481.7	42.51	8	23	0.6611
Microglia vs. Macrophages	524.3	533	-8.75	8	7	0.1079
Microglia vs. Neurogenesis	524.3	369.1	155.2	8	12	2.17
Microglia vs. Ependyma	524.3	403.6	120.7	8	5	1.351
Oligodendrocytes vs. Polydendrocytes	397.3	343.8	53.44	43	37	1.521
Oligodendrocytes vs. Astrocytes	397.3	481.7	-84.46	43	23	2.087
Oligodendrocytes vs. Macrophages	397.3	533	-135.7	43	7	2.126
Oligodendrocytes vs. Neurogenesis	397.3	369.1	28.2	43	12	0.5512
Oligodendrocytes vs. Ependyma	397.3	403.6	-6.321	43	5	0.08539
Polydendrocytes vs. Astrocytes	343.8	481.7	-137.9	37	23	3.315
Polydendrocytes vs. Macrophages	343.8	533	-189.2	37	7	2.929
Polydendrocytes vs. Neurogenesis	343.8	369.1	-25.25	37	12	0.4851
Polydendrocytes vs. Ependyma	343.8	403.6	-59.76	37	5	0.8006
Astrocytes vs. Macrophages	481.7	533	-51.26	23	7	0.758
Astrocytes vs. Neurogenesis	481.7	369.1	112.7	23	12	2.019

Astrocytes vs. Ependyma	481.7	403.6	78.14	23	5	1.011
Macrophages vs. Neurogenesis	533	369.1	163.9	7	12	2.2
Macrophages vs. Ependyma	533	403.6	129.4	7	5	1.411
Neurogenesis vs. Ependyma	369.1	403.6	-34.52	12	5	0.4139

Nfe2l3-cell

Number of families	1
Number of comparisons per family	55
Alpha	0.05

Dunn's multiple comparisons test	Mean rank diff.	Significant?	Summary	Adjusted P Value	
Neurons vs. Endothelial Cells	66.66	No	ns	0.7115	A-B
Neurons vs. Fibroblast-like	20.46	No	ns	>0.9999	A-C
Neurons vs. Mural Cells	103.2	Yes	**	0.0035	A-D
Neurons vs. Microglia	31.95	No	ns	>0.9999	A-E
Neurons vs. Oligodendrocytes	-237.3	Yes	****	<0.0001	A-F
Neurons vs. Polydendrocytes	-155.6	Yes	****	<0.0001	A-G
Neurons vs. Astrocytes	57.95	No	ns	>0.9999	A-H
Neurons vs. Macrophages	81.81	No	ns	>0.9999	A-I
Neurons vs. Neurogenesis	56.59	No	ns	>0.9999	A-J
Neurons vs. Ependyma	91.78	No	ns	>0.9999	A-K
Endothelial Cells vs. Fibroblast-like	-46.2	No	ns	>0.9999	B-C
Endothelial Cells vs. Mural Cells	36.52	No	ns	>0.9999	B-D
Endothelial Cells vs. Microglia	-34.72	No	ns	>0.9999	B-E
Endothelial Cells vs. Oligodendrocytes	-304	Yes	****	<0.0001	B-F
Endothelial Cells vs. Polydendrocytes	-222.3	Yes	****	<0.0001	B-G
Endothelial Cells vs. Astrocytes	-8.714	No	ns	>0.9999	B-H
Endothelial Cells vs. Macrophages	15.15	No	ns	>0.9999	B-I
Endothelial Cells vs. Neurogenesis	-10.07	No	ns	>0.9999	B-J
Endothelial Cells vs. Ependyma	25.12	No	ns	>0.9999	B-K
Fibroblast-like vs. Mural Cells	82.72	No	ns	>0.9999	C-D
Fibroblast-like vs. Microglia	11.48	No	ns	>0.9999	C-E
Fibroblast-like vs. Oligodendrocytes	-257.8	Yes	****	<0.0001	C-F
Fibroblast-like vs. Polydendrocytes	-176.1	Yes	***	0.0003	C-G
Fibroblast-like vs. Astrocytes	37.49	No	ns	>0.9999	C-H
Fibroblast-like vs. Macrophages	61.35	No	ns	>0.9999	C-I
Fibroblast-like vs. Neurogenesis	36.13	No	ns	>0.9999	C-J
Fibroblast-like vs. Ependyma	71.32	No	ns	>0.9999	C-K
Mural Cells vs. Microglia	-71.23	No	ns	>0.9999	D-E
Mural Cells vs. Oligodendrocytes	-340.5	Yes	****	<0.0001	D-F
Mural Cells vs. Polydendrocytes	-258.8	Yes	****	<0.0001	D-G
Mural Cells vs. Astrocytes	-45.23	No	ns	>0.9999	D-H
Mural Cells vs. Macrophages	-21.37	No	ns	>0.9999	D-I
Mural Cells vs. Neurogenesis	-46.59	No	ns	>0.9999	D-J
Mural Cells vs. Ependyma	-11.4	No	ns	>0.9999	D-K

Microglia vs. Oligodendrocytes	-269.3	Yes	***	0.0001	E-F
Microglia vs. Polydendrocytes	-187.6	No	ns	0.0662	E-G
Microglia vs. Astrocytes	26	No	ns	>0.9999	E-H
Microglia vs. Macrophages	49.87	No	ns	>0.9999	E-I
Microglia vs. Neurogenesis	24.65	No	ns	>0.9999	E-J
Microglia vs. Ependyma	59.84	No	ns	>0.9999	E-K
Oligodendrocytes vs. Polydendrocytes	81.72	No	ns	0.7796	F-G
Oligodendrocytes vs. Astrocytes	295.3	Yes	****	<0.0001	F-H
Oligodendrocytes vs. Macrophages	319.2	Yes	****	<0.0001	F-I
Oligodendrocytes vs. Neurogenesis	293.9	Yes	****	<0.0001	F-J
Oligodendrocytes vs. Ependyma	329.1	Yes	***	0.0002	F-K
Polydendrocytes vs. Astrocytes	213.6	Yes	****	<0.0001	G-H
Polydendrocytes vs. Macrophages	237.4	Yes	**	0.0058	G-I
Polydendrocytes vs. Neurogenesis	212.2	Yes	***	0.0009	G-J
Polydendrocytes vs. Ependyma	247.4	Yes	*	0.0261	G-K
Astrocytes vs. Macrophages	23.86	No	ns	>0.9999	H-I
Astrocytes vs. Neurogenesis	-1.357	No	ns	>0.9999	H-J
Astrocytes vs. Ependyma	33.83	No	ns	>0.9999	H-K
Macrophages vs. Neurogenesis	-25.22	No	ns	>0.9999	I-J
Macrophages vs. Ependyma	9.971	No	ns	>0.9999	I-K
Neurogenesis vs. Ependyma	35.19	No	ns	>0.9999	J-K

Test details	Mean rank 1	Mean rank 2	Mean rank diff.	n1	n2	Z
Neurons vs. Endothelial Cells	262.4	195.7	66.66	315	34	2.486
Neurons vs. Fibroblast-like	262.4	241.9	20.46	315	25	0.6629
Neurons vs. Mural Cells	262.4	159.2	103.2	315	37	3.996
Neurons vs. Microglia	262.4	230.4	31.95	315	8	0.6006
Neurons vs. Oligodendrocytes	262.4	499.7	-237.3	315	43	9.826
Neurons vs. Polydendrocytes	262.4	418	-155.6	315	37	6.027
Neurons vs. Astrocytes	262.4	204.4	57.95	315	23	1.806
Neurons vs. Macrophages	262.4	180.6	81.81	315	7	1.441
Neurons vs. Neurogenesis	262.4	205.8	56.59	315	12	1.295
Neurons vs. Ependyma	262.4	170.6	91.78	315	5	1.371
Endothelial Cells vs. Fibroblast-like	195.7	241.9	-46.2	34	25	1.18
Endothelial Cells vs. Mural Cells	195.7	159.2	36.52	34	37	1.035
Endothelial Cells vs. Microglia	195.7	230.4	-34.72	34	8	0.5946
Endothelial Cells vs. Oligodendrocytes	195.7	499.7	-304	34	43	8.916
Endothelial Cells vs. Polydendrocytes	195.7	418	-222.3	34	37	6.298
Endothelial Cells vs. Astrocytes	195.7	204.4	-8.714	34	23	0.2172
Endothelial Cells vs. Macrophages	195.7	180.6	15.15	34	7	0.2457
Endothelial Cells vs. Neurogenesis	195.7	205.8	-10.07	34	12	0.2019
Endothelial Cells vs. Ependyma	195.7	170.6	25.12	34	5	0.353
Fibroblast-like vs. Mural Cells	241.9	159.2	82.72	25	37	2.15
Fibroblast-like vs. Microglia	241.9	230.4	11.48	25	8	0.1903
Fibroblast-like vs. Oligodendrocytes	241.9	499.7	-257.8	25	43	6.899
Fibroblast-like vs. Polydendrocytes	241.9	418	-176.1	25	37	4.578

Fibroblast-like vs. Astrocytes	241.9	204.4	37.49	25	23	0.8732
Fibroblast-like vs. Macrophages	241.9	180.6	61.35	25	7	0.9656
Fibroblast-like vs. Neurogenesis	241.9	205.8	36.13	25	12	0.6924
Fibroblast-like vs. Ependyma	241.9	170.6	71.32	25	5	0.9798
Mural Cells vs. Microglia	159.2	230.4	-71.23	37	8	1.23
Mural Cells vs. Oligodendrocytes	159.2	499.7	-340.5	37	43	10.22
Mural Cells vs. Polydendrocytes	159.2	418	-258.8	37	37	7.492
Mural Cells vs. Astrocytes	159.2	204.4	-45.23	37	23	1.147
Mural Cells vs. Macrophages	159.2	180.6	-21.37	37	7	0.3489
Mural Cells vs. Neurogenesis	159.2	205.8	-46.59	37	12	0.9439
Mural Cells vs. Ependyma	159.2	170.6	-11.4	37	5	0.161
Microglia vs. Oligodendrocytes	230.4	499.7	-269.3	8	43	4.707
Microglia vs. Polydendrocytes	230.4	418	-187.6	8	37	3.238
Microglia vs. Astrocytes	230.4	204.4	26	8	23	0.4264
Microglia vs. Macrophages	230.4	180.6	49.87	8	7	0.6485
Microglia vs. Neurogenesis	230.4	205.8	24.65	8	12	0.3634
Microglia vs. Ependyma	230.4	170.6	59.84	8	5	0.7064
Oligodendrocytes vs. Polydendrocytes	499.7	418	81.72	43	37	2.453
Oligodendrocytes vs. Astrocytes	499.7	204.4	295.3	43	23	7.694
Oligodendrocytes vs. Macrophages	499.7	180.6	319.2	43	7	5.271
Oligodendrocytes vs. Neurogenesis	499.7	205.8	293.9	43	12	6.06
Oligodendrocytes vs. Ependyma	499.7	170.6	329.1	43	5	4.688
Polydendrocytes vs. Astrocytes	418	204.4	213.6	37	23	5.414
Polydendrocytes vs. Macrophages	418	180.6	237.4	37	7	3.877
Polydendrocytes vs. Neurogenesis	418	205.8	212.2	37	12	4.3
Polydendrocytes vs. Ependyma	418	170.6	247.4	37	5	3.495
Astrocytes vs. Macrophages	204.4	180.6	23.86	23	7	0.3721
Astrocytes vs. Neurogenesis	204.4	205.8	-1.357	23	12	0.02565
Astrocytes vs. Ependyma	204.4	170.6	33.83	23	5	0.4615
Macrophages vs. Neurogenesis	180.6	205.8	-25.22	7	12	0.3569
Macrophages vs. Ependyma	180.6	170.6	9.971	7	5	0.1146
Neurogenesis vs. Ependyma	205.8	170.6	35.19	12	5	0.445

Bach1-cell

Number of families	1
Number of comparisons per family	55
Alpha	0.05

Dunn's multiple comparisons test	Mean rank diff.	Significant?	Summary	Adjusted P Value	
Neurons vs. Endothelial Cells	-287.6	Yes	****	<0.0001	A-B
Neurons vs. Fibroblast-like	-247.1	Yes	****	<0.0001	A-C
Neurons vs. Mural Cells	-242.8	Yes	****	<0.0001	A-D
Neurons vs. Microglia	-344.3	Yes	****	<0.0001	A-E
Neurons vs. Oligodendrocytes	-137.5	Yes	****	<0.0001	A-F
Neurons vs. Polydendrocytes	-198.6	Yes	****	<0.0001	A-G
Neurons vs. Astrocytes	-15.86	No	ns	>0.9999	A-H

Neurons vs. Macrophages	-263	Yes	***	0.0007	A-I
Neurons vs. Neurogenesis	-178.6	Yes	**	0.0065	A-J
Neurons vs. Ependyma	19.91	No	ns	>0.9999	A-K
Endothelial Cells vs. Fibroblast-like	40.5	No	ns	>0.9999	B-C
Endothelial Cells vs. Mural Cells	44.78	No	ns	>0.9999	B-D
Endothelial Cells vs. Microglia	-56.74	No	ns	>0.9999	B-E
Endothelial Cells vs. Oligodendrocytes	150	Yes	**	0.0019	B-F
Endothelial Cells vs. Polydendrocytes	88.91	No	ns	0.9716	B-G
Endothelial Cells vs. Astrocytes	271.7	Yes	****	<0.0001	B-H
Endothelial Cells vs. Macrophages	24.55	No	ns	>0.9999	B-I
Endothelial Cells vs. Neurogenesis	108.9	No	ns	>0.9999	B-J
Endothelial Cells vs. Ependyma	307.5	Yes	**	0.0026	B-K
Fibroblast-like vs. Mural Cells	4.274	No	ns	>0.9999	C-D
Fibroblast-like vs. Microglia	-97.24	No	ns	>0.9999	C-E
Fibroblast-like vs. Oligodendrocytes	109.5	No	ns	0.3178	C-F
Fibroblast-like vs. Polydendrocytes	48.41	No	ns	>0.9999	C-G
Fibroblast-like vs. Astrocytes	231.2	Yes	****	<0.0001	C-H
Fibroblast-like vs. Macrophages	-15.95	No	ns	>0.9999	C-I
Fibroblast-like vs. Neurogenesis	68.43	No	ns	>0.9999	C-J
Fibroblast-like vs. Ependyma	267	Yes	*	0.0303	C-K
Mural Cells vs. Microglia	-101.5	No	ns	>0.9999	D-E
Mural Cells vs. Oligodendrocytes	105.2	No	ns	0.1612	D-F
Mural Cells vs. Polydendrocytes	44.14	No	ns	>0.9999	D-G
Mural Cells vs. Astrocytes	226.9	Yes	****	<0.0001	D-H
Mural Cells vs. Macrophages	-20.23	No	ns	>0.9999	D-I
Mural Cells vs. Neurogenesis	64.15	No	ns	>0.9999	D-J
Mural Cells vs. Ependyma	262.7	Yes	*	0.0261	D-K
Microglia vs. Oligodendrocytes	206.7	Yes	*	0.0365	E-F
Microglia vs. Polydendrocytes	145.6	No	ns	0.9836	E-G
Microglia vs. Astrocytes	328.4	Yes	****	<0.0001	E-H
Microglia vs. Macrophages	81.29	No	ns	>0.9999	E-I
Microglia vs. Neurogenesis	165.7	No	ns	>0.9999	E-J
Microglia vs. Ependyma	364.2	Yes	**	0.0028	E-K
Oligodendrocytes vs. Polydendrocytes	-61.1	No	ns	>0.9999	F-G
Oligodendrocytes vs. Astrocytes	121.7	No	ns	0.1553	F-H
Oligodendrocytes vs. Macrophages	-125.5	No	ns	>0.9999	F-I
Oligodendrocytes vs. Neurogenesis	-41.08	No	ns	>0.9999	F-J
Oligodendrocytes vs. Ependyma	157.5	No	ns	>0.9999	F-K
Polydendrocytes vs. Astrocytes	182.8	Yes	***	0.0007	G-H
Polydendrocytes vs. Macrophages	-64.36	No	ns	>0.9999	G-I
Polydendrocytes vs. Neurogenesis	20.02	No	ns	>0.9999	G-J
Polydendrocytes vs. Ependyma	218.6	No	ns	0.2002	G-K
Astrocytes vs. Macrophages	-247.1	Yes	*	0.0156	H-I
Astrocytes vs. Neurogenesis	-162.8	No	ns	0.2069	H-J
Astrocytes vs. Ependyma	35.77	No	ns	>0.9999	H-K

Macrophages vs. Neurogenesis	84.38	No	ns	>0.9999	I-J	
Macrophages vs. Ependyma	282.9	No	ns	0.1205	I-K	
Neurogenesis vs. Ependyma	198.5	No	ns	0.9932	J-K	

Test details	Mean rank 1	Mean rank 2	Mean rank diff.	n1	n2	Z
Neurons vs. Endothelial Cells	190.7	478.3	-287.6	315	34	10.1
Neurons vs. Fibroblast-like	190.7	437.8	-247.1	315	25	7.537
Neurons vs. Mural Cells	190.7	433.5	-242.8	315	37	8.856
Neurons vs. Microglia	190.7	535	-344.3	315	8	6.096
Neurons vs. Oligodendrocytes	190.7	328.3	-137.5	315	43	5.363
Neurons vs. Polydendrocytes	190.7	389.4	-198.6	315	37	7.246
Neurons vs. Astrocytes	190.7	206.6	-15.86	315	23	0.4654
Neurons vs. Macrophages	190.7	453.7	-263	315	7	4.363
Neurons vs. Neurogenesis	190.7	369.3	-178.6	315	12	3.85
Neurons vs. Ependyma	190.7	170.8	19.91	315	5	0.28
Endothelial Cells vs. Fibroblast-like	478.3	437.8	40.5	34	25	0.9746
Endothelial Cells vs. Mural Cells	478.3	433.5	44.78	34	37	1.195
Endothelial Cells vs. Microglia	478.3	535	-56.74	34	8	0.9153
Endothelial Cells vs. Oligodendrocytes	478.3	328.3	150	34	43	4.144
Endothelial Cells vs. Polydendrocytes	478.3	389.4	88.91	34	37	2.373
Endothelial Cells vs. Astrocytes	478.3	206.6	271.7	34	23	6.38
Endothelial Cells vs. Macrophages	478.3	453.7	24.55	34	7	0.375
Endothelial Cells vs. Neurogenesis	478.3	369.3	108.9	34	12	2.057
Endothelial Cells vs. Ependyma	478.3	170.8	307.5	34	5	4.069
Fibroblast-like vs. Mural Cells	437.8	433.5	4.274	25	37	0.1046
Fibroblast-like vs. Microglia	437.8	535	-97.24	25	8	1.518
Fibroblast-like vs. Oligodendrocytes	437.8	328.3	109.5	25	43	2.76
Fibroblast-like vs. Polydendrocytes	437.8	389.4	48.41	25	37	1.185
Fibroblast-like vs. Astrocytes	437.8	206.6	231.2	25	23	5.073
Fibroblast-like vs. Macrophages	437.8	453.7	-15.95	25	7	0.2365
Fibroblast-like vs. Neurogenesis	437.8	369.3	68.43	25	12	1.235
Fibroblast-like vs. Ependyma	437.8	170.8	267	25	5	3.454
Mural Cells vs. Microglia	433.5	535	-101.5	37	8	1.65
Mural Cells vs. Oligodendrocytes	433.5	328.3	105.2	37	43	2.975
Mural Cells vs. Polydendrocytes	433.5	389.4	44.14	37	37	1.203
Mural Cells vs. Astrocytes	433.5	206.6	226.9	37	23	5.418
Mural Cells vs. Macrophages	433.5	453.7	-20.23	37	7	0.3111
Mural Cells vs. Neurogenesis	433.5	369.3	64.15	37	12	1.224
Mural Cells vs. Ependyma	433.5	170.8	262.7	37	5	3.495
Microglia vs. Oligodendrocytes	535	328.3	206.7	8	43	3.404
Microglia vs. Polydendrocytes	535	389.4	145.6	8	37	2.368
Microglia vs. Astrocytes	535	206.6	328.4	8	23	5.072
Microglia vs. Macrophages	535	453.7	81.29	8	7	0.9956
Microglia vs. Neurogenesis	535	369.3	165.7	8	12	2.301
Microglia vs. Ependyma	535	170.8	364.2	8	5	4.05
Oligodendrocytes vs. Polydendrocytes	328.3	389.4	-61.1	43	37	1.727

Oligodendrocytes vs. Astrocytes	328.3	206.6	121.7	43	23	2.986
Oligodendrocytes vs. Macrophages	328.3	453.7	-125.5	43	7	1.951
Oligodendrocytes vs. Neurogenesis	328.3	369.3	-41.08	43	12	0.7976
Oligodendrocytes vs. Ependyma	328.3	170.8	157.5	43	5	2.113
Polydendrocytes vs. Astrocytes	389.4	206.6	182.8	37	23	4.364
Polydendrocytes vs. Macrophages	389.4	453.7	-64.36	37	7	0.9899
Polydendrocytes vs. Neurogenesis	389.4	369.3	20.02	37	12	0.382
Polydendrocytes vs. Ependyma	389.4	170.8	218.6	37	5	2.908
Astrocytes vs. Macrophages	206.6	453.7	-247.1	23	7	3.63
Astrocytes vs. Neurogenesis	206.6	369.3	-162.8	23	12	2.898
Astrocytes vs. Ependyma	206.6	170.8	35.77	23	5	0.4595
Macrophages vs. Neurogenesis	453.7	369.3	84.38	7	12	1.125
Macrophages vs. Ependyma	453.7	170.8	282.9	7	5	3.063
Neurogenesis vs. Ependyma	369.3	170.8	198.5	12	5	2.364

Bach2-cell

Number of families	1
Number of comparisons per family	55
Alpha	0.05

Dunn's multiple comparisons test	Mean rank diff.	Significant?	Summary	Adjusted P Value	
Neurons vs. Endothelial Cells	66.85	No	ns	>0.9999	A-B
Neurons vs. Fibroblast-like	190.7	Yes	****	<0.0001	A-C
Neurons vs. Mural Cells	212.8	Yes	****	<0.0001	A-D
Neurons vs. Microglia	-42.73	No	ns	>0.9999	A-E
Neurons vs. Oligodendrocytes	86.37	Yes	*	0.0413	A-F
Neurons vs. Polydendrocytes	-41.54	No	ns	>0.9999	A-G
Neurons vs. Astrocytes	-19.43	No	ns	>0.9999	A-H
Neurons vs. Macrophages	65.74	No	ns	>0.9999	A-I
Neurons vs. Neurogenesis	133.6	No	ns	0.218	A-J
Neurons vs. Ependyma	151.1	No	ns	>0.9999	A-K
Endothelial Cells vs. Fibroblast-like	123.9	No	ns	0.1567	B-C
Endothelial Cells vs. Mural Cells	146	Yes	**	0.0053	B-D
Endothelial Cells vs. Microglia	-109.6	No	ns	>0.9999	B-E
Endothelial Cells vs. Oligodendrocytes	19.53	No	ns	>0.9999	B-F
Endothelial Cells vs. Polydendrocytes	-108.4	No	ns	0.2088	B-G
Endothelial Cells vs. Astrocytes	-86.28	No	ns	>0.9999	B-H
Endothelial Cells vs. Macrophages	-1.109	No	ns	>0.9999	B-I
Endothelial Cells vs. Neurogenesis	66.72	No	ns	>0.9999	B-J
Endothelial Cells vs. Ependyma	84.28	No	ns	>0.9999	B-K
Fibroblast-like vs. Mural Cells	22.09	No	ns	>0.9999	C-D
Fibroblast-like vs. Microglia	-233.5	Yes	*	0.0146	C-E
Fibroblast-like vs. Oligodendrocytes	-104.4	No	ns	0.4658	C-F
Fibroblast-like vs. Polydendrocytes	-232.3	Yes	****	<0.0001	C-G
Fibroblast-like vs. Astrocytes	-210.2	Yes	***	0.0002	C-H

Fibroblast-like vs. Macrophages	-125	No	ns	>0.9999	C-I
Fibroblast-like vs. Neurogenesis	-57.18	No	ns	>0.9999	C-J
Fibroblast-like vs. Ependyma	-39.62	No	ns	>0.9999	C-K
Mural Cells vs. Microglia	-255.6	Yes	**	0.0018	D-E
Mural Cells vs. Oligodendrocytes	-126.5	Yes	*	0.019	D-F
Mural Cells vs. Polydendrocytes	-254.4	Yes	****	<0.0001	D-G
Mural Cells vs. Astrocytes	-232.3	Yes	****	<0.0001	D-H
Mural Cells vs. Macrophages	-147.1	No	ns	>0.9999	D-I
Mural Cells vs. Neurogenesis	-79.27	No	ns	>0.9999	D-J
Mural Cells vs. Ependyma	-61.71	No	ns	>0.9999	D-K
Microglia vs. Oligodendrocytes	129.1	No	ns	>0.9999	E-F
Microglia vs. Polydendrocytes	1.182	No	ns	>0.9999	E-G
Microglia vs. Astrocytes	23.29	No	ns	>0.9999	E-H
Microglia vs. Macrophages	108.5	No	ns	>0.9999	E-I
Microglia vs. Neurogenesis	176.3	No	ns	0.7848	E-J
Microglia vs. Ependyma	193.9	No	ns	>0.9999	E-K
Oligodendrocytes vs. Polydendrocytes	-127.9	Yes	*	0.0163	F-G
Oligodendrocytes vs. Astrocytes	-105.8	No	ns	0.515	F-H
Oligodendrocytes vs. Macrophages	-20.63	No	ns	>0.9999	F-I
Oligodendrocytes vs. Neurogenesis	47.19	No	ns	>0.9999	F-J
Oligodendrocytes vs. Ependyma	64.75	No	ns	>0.9999	F-K
Polydendrocytes vs. Astrocytes	22.11	No	ns	>0.9999	G-H
Polydendrocytes vs. Macrophages	107.3	No	ns	>0.9999	G-I
Polydendrocytes vs. Neurogenesis	175.1	Yes	*	0.0454	G-J
Polydendrocytes vs. Ependyma	192.7	No	ns	0.5668	G-K
Astrocytes vs. Macrophages	85.17	No	ns	>0.9999	H-I
Astrocytes vs. Neurogenesis	153	No	ns	0.3528	H-J
Astrocytes vs. Ependyma	170.6	No	ns	>0.9999	H-K
Macrophages vs. Neurogenesis	67.83	No	ns	>0.9999	I-J
Macrophages vs. Ependyma	85.39	No	ns	>0.9999	I-K
Neurogenesis vs. Ependyma	17.56	No	ns	>0.9999	J-K

Test details	Mean rank 1	Mean rank 2	Mean rank diff.	n1	n2	Z
Neurons vs. Endothelial Cells	308.5	241.7	66.85	315	34	2.349
Neurons vs. Fibroblast-like	308.5	117.8	190.7	315	25	5.824
Neurons vs. Mural Cells	308.5	95.69	212.8	315	37	7.77
Neurons vs. Microglia	308.5	351.3	-42.73	315	8	0.7571
Neurons vs. Oligodendrocytes	308.5	222.2	86.37	315	43	3.371
Neurons vs. Polydendrocytes	308.5	350.1	-41.54	315	37	1.517
Neurons vs. Astrocytes	308.5	328	-19.43	315	23	0.5708
Neurons vs. Macrophages	308.5	242.8	65.74	315	7	1.091
Neurons vs. Neurogenesis	308.5	175	133.6	315	12	2.881
Neurons vs. Ependyma	308.5	157.4	151.1	315	5	2.127
Endothelial Cells vs. Fibroblast-like	241.7	117.8	123.9	34	25	2.984
Endothelial Cells vs. Mural Cells	241.7	95.69	146	34	37	3.899
Endothelial Cells vs. Microglia	241.7	351.3	-109.6	34	8	1.769

Endothelial Cells vs. Oligodendrocytes	241.7	222.2	19.53	34	43	0.5398
Endothelial Cells vs. Polydendrocytes	241.7	350.1	-108.4	34	37	2.895
Endothelial Cells vs. Astrocytes	241.7	328	-86.28	34	23	2.027
Endothelial Cells vs. Macrophages	241.7	242.8	-1.109	34	7	0.01696
Endothelial Cells vs. Neurogenesis	241.7	175	66.72	34	12	1.261
Endothelial Cells vs. Ependyma	241.7	157.4	84.28	34	5	1.116
Fibroblast-like vs. Mural Cells	117.8	95.69	22.09	25	37	0.5413
Fibroblast-like vs. Microglia	117.8	351.3	-233.5	25	8	3.646
Fibroblast-like vs. Oligodendrocytes	117.8	222.2	-104.4	25	43	2.633
Fibroblast-like vs. Polydendrocytes	117.8	350.1	-232.3	25	37	5.692
Fibroblast-like vs. Astrocytes	117.8	328	-210.2	25	23	4.615
Fibroblast-like vs. Macrophages	117.8	242.8	-125	25	7	1.855
Fibroblast-like vs. Neurogenesis	117.8	175	-57.18	25	12	1.033
Fibroblast-like vs. Ependyma	117.8	157.4	-39.62	25	5	0.5131
Mural Cells vs. Microglia	95.69	351.3	-255.6	37	8	4.158
Mural Cells vs. Oligodendrocytes	95.69	222.2	-126.5	37	43	3.578
Mural Cells vs. Polydendrocytes	95.69	350.1	-254.4	37	37	6.941
Mural Cells vs. Astrocytes	95.69	328	-232.3	37	23	5.55
Mural Cells vs. Macrophages	95.69	242.8	-147.1	37	7	2.264
Mural Cells vs. Neurogenesis	95.69	175	-79.27	37	12	1.514
Mural Cells vs. Ependyma	95.69	157.4	-61.71	37	5	0.8217
Microglia vs. Oligodendrocytes	351.3	222.2	129.1	8	43	2.127
Microglia vs. Polydendrocytes	351.3	350.1	1.182	8	37	0.01924
Microglia vs. Astrocytes	351.3	328	23.29	8	23	0.36
Microglia vs. Macrophages	351.3	242.8	108.5	8	7	1.33
Microglia vs. Neurogenesis	351.3	175	176.3	8	12	2.45
Microglia vs. Ependyma	351.3	157.4	193.9	8	5	2.157
Oligodendrocytes vs. Polydendrocytes	222.2	350.1	-127.9	43	37	3.619
Oligodendrocytes vs. Astrocytes	222.2	328	-105.8	43	23	2.598
Oligodendrocytes vs. Macrophages	222.2	242.8	-20.63	43	7	0.3212
Oligodendrocytes vs. Neurogenesis	222.2	175	47.19	43	12	0.9171
Oligodendrocytes vs. Ependyma	222.2	157.4	64.75	43	5	0.8694
Polydendrocytes vs. Astrocytes	350.1	328	22.11	37	23	0.5283
Polydendrocytes vs. Macrophages	350.1	242.8	107.3	37	7	1.651
Polydendrocytes vs. Neurogenesis	350.1	175	175.1	37	12	3.344
Polydendrocytes vs. Ependyma	350.1	157.4	192.7	37	5	2.565
Astrocytes vs. Macrophages	328	242.8	85.17	23	7	1.252
Astrocytes vs. Neurogenesis	328	175	153	23	12	2.726
Astrocytes vs. Ependyma	328	157.4	170.6	23	5	2.193
Macrophages vs. Neurogenesis	242.8	175	67.83	7	12	0.9048
Macrophages vs. Ependyma	242.8	157.4	85.39	7	5	0.9252
Neurogenesis vs. Ependyma	175	157.4	17.56	12	5	0.2093

Keap1-cell

Number of families	1
Number of comparisons per family	55

Alpha	0.05				
Dunn's multiple comparisons test	Mean rank diff.	Significant?	Summary	Adjusted P Value	
Neurons vs. Endothelial Cells	-189.8	Yes	****	<0.0001	A-B
Neurons vs. Fibroblast-like	1.989	No	ns	>0.9999	A-C
Neurons vs. Mural Cells	-17.18	No	ns	>0.9999	A-D
Neurons vs. Microglia	104.8	No	ns	>0.9999	A-E
Neurons vs. Oligodendrocytes	37.17	No	ns	>0.9999	A-F
Neurons vs. Polydendrocytes	-14.26	No	ns	>0.9999	A-G
Neurons vs. Astrocytes	35.67	No	ns	>0.9999	A-H
Neurons vs. Macrophages	-26.42	No	ns	>0.9999	A-I
Neurons vs. Neurogenesis	-14.1	No	ns	>0.9999	A-J
Neurons vs. Ependyma	20.95	No	ns	>0.9999	A-K
Endothelial Cells vs. Fibroblast-like	191.8	Yes	***	0.0002	B-C
Endothelial Cells vs. Mural Cells	172.6	Yes	***	0.0002	B-D
Endothelial Cells vs. Microglia	294.6	Yes	***	0.0001	B-E
Endothelial Cells vs. Oligodendrocytes	227	Yes	****	<0.0001	B-F
Endothelial Cells vs. Polydendrocytes	175.6	Yes	***	0.0002	B-G
Endothelial Cells vs. Astrocytes	225.5	Yes	****	<0.0001	B-H
Endothelial Cells vs. Macrophages	163.4	No	ns	0.6919	B-I
Endothelial Cells vs. Neurogenesis	175.7	No	ns	0.05	B-J
Endothelial Cells vs. Ependyma	210.8	No	ns	0.2905	B-K
Fibroblast-like vs. Mural Cells	-19.16	No	ns	>0.9999	C-D
Fibroblast-like vs. Microglia	102.8	No	ns	>0.9999	C-E
Fibroblast-like vs. Oligodendrocytes	35.18	No	ns	>0.9999	C-F
Fibroblast-like vs. Polydendrocytes	-16.25	No	ns	>0.9999	C-G
Fibroblast-like vs. Astrocytes	33.68	No	ns	>0.9999	C-H
Fibroblast-like vs. Macrophages	-28.41	No	ns	>0.9999	C-I
Fibroblast-like vs. Neurogenesis	-16.09	No	ns	>0.9999	C-J
Fibroblast-like vs. Ependyma	18.96	No	ns	>0.9999	C-K
Mural Cells vs. Microglia	121.9	No	ns	>0.9999	D-E
Mural Cells vs. Oligodendrocytes	54.35	No	ns	>0.9999	D-F
Mural Cells vs. Polydendrocytes	2.919	No	ns	>0.9999	D-G
Mural Cells vs. Astrocytes	52.85	No	ns	>0.9999	D-H
Mural Cells vs. Macrophages	-9.247	No	ns	>0.9999	D-I
Mural Cells vs. Neurogenesis	3.074	No	ns	>0.9999	D-J
Mural Cells vs. Ependyma	38.12	No	ns	>0.9999	D-K
Microglia vs. Oligodendrocytes	-67.6	No	ns	>0.9999	E-F
Microglia vs. Polydendrocytes	-119	No	ns	>0.9999	E-G
Microglia vs. Astrocytes	-69.1	No	ns	>0.9999	E-H
Microglia vs. Macrophages	-131.2	No	ns	>0.9999	E-I
Microglia vs. Neurogenesis	-118.9	No	ns	>0.9999	E-J
Microglia vs. Ependyma	-83.83	No	ns	>0.9999	E-K
Oligodendrocytes vs. Polydendrocytes	-51.43	No	ns	>0.9999	F-G
Oligodendrocytes vs. Astrocytes	-1.502	No	ns	>0.9999	F-H
Oligodendrocytes vs. Macrophages	-63.59	No	ns	>0.9999	F-I

Oligodendrocytes vs. Neurogenesis	-51.27	No	ns	>0.9999	F-J
Oligodendrocytes vs. Ependyma	-16.22	No	ns	>0.9999	F-K
Polydendrocytes vs. Astrocytes	49.93	No	ns	>0.9999	G-H
Polydendrocytes vs. Macrophages	-12.17	No	ns	>0.9999	G-I
Polydendrocytes vs. Neurogenesis	0.1554	No	ns	>0.9999	G-J
Polydendrocytes vs. Ependyma	35.21	No	ns	>0.9999	G-K
Astrocytes vs. Macrophages	-62.09	No	ns	>0.9999	H-I
Astrocytes vs. Neurogenesis	-49.77	No	ns	>0.9999	H-J
Astrocytes vs. Ependyma	-14.72	No	ns	>0.9999	H-K
Macrophages vs. Neurogenesis	12.32	No	ns	>0.9999	I-J
Macrophages vs. Ependyma	47.37	No	ns	>0.9999	I-K
Neurogenesis vs. Ependyma	35.05	No	ns	>0.9999	J-K

Test details	Mean rank 1	Mean rank 2	Mean rank diff.	n1	n2	Z
Neurons vs. Endothelial Cells	265.1	455	-189.8	315	34	6.665
Neurons vs. Fibroblast-like	265.1	263.2	1.989	315	25	0.06068
Neurons vs. Mural Cells	265.1	282.3	-17.18	315	37	0.6264
Neurons vs. Microglia	265.1	160.4	104.8	315	8	1.855
Neurons vs. Oligodendrocytes	265.1	228	37.17	315	43	1.449
Neurons vs. Polydendrocytes	265.1	279.4	-14.26	315	37	0.52
Neurons vs. Astrocytes	265.1	229.5	35.67	315	23	1.047
Neurons vs. Macrophages	265.1	291.6	-26.42	315	7	0.4383
Neurons vs. Neurogenesis	265.1	279.3	-14.1	315	12	0.3039
Neurons vs. Ependyma	265.1	244.2	20.95	315	5	0.2946
Endothelial Cells vs. Fibroblast-like	455	263.2	191.8	34	25	4.615
Endothelial Cells vs. Mural Cells	455	282.3	172.6	34	37	4.606
Endothelial Cells vs. Microglia	455	160.4	294.6	34	8	4.752
Endothelial Cells vs. Oligodendrocytes	455	228	227	34	43	6.27
Endothelial Cells vs. Polydendrocytes	455	279.4	175.6	34	37	4.684
Endothelial Cells vs. Astrocytes	455	229.5	225.5	34	23	5.294
Endothelial Cells vs. Macrophages	455	291.6	163.4	34	7	2.495
Endothelial Cells vs. Neurogenesis	455	279.3	175.7	34	12	3.317
Endothelial Cells vs. Ependyma	455	244.2	210.8	34	5	2.789
Fibroblast-like vs. Mural Cells	263.2	282.3	-19.16	25	37	0.4692
Fibroblast-like vs. Microglia	263.2	160.4	102.8	25	8	1.604
Fibroblast-like vs. Oligodendrocytes	263.2	228	35.18	25	43	0.8867
Fibroblast-like vs. Polydendrocytes	263.2	279.4	-16.25	25	37	0.3977
Fibroblast-like vs. Astrocytes	263.2	229.5	33.68	25	23	0.7389
Fibroblast-like vs. Macrophages	263.2	291.6	-28.41	25	7	0.4212
Fibroblast-like vs. Neurogenesis	263.2	279.3	-16.09	25	12	0.2904
Fibroblast-like vs. Ependyma	263.2	244.2	18.96	25	5	0.2453
Mural Cells vs. Microglia	282.3	160.4	121.9	37	8	1.983
Mural Cells vs. Oligodendrocytes	282.3	228	54.35	37	43	1.536
Mural Cells vs. Polydendrocytes	282.3	279.4	2.919	37	37	0.07958
Mural Cells vs. Astrocytes	282.3	229.5	52.85	37	23	1.262
Mural Cells vs. Macrophages	282.3	291.6	-9.247	37	7	0.1422

Mural Cells vs. Neurogenesis	282.3	279.3	3.074	37	12	0.05866
Mural Cells vs. Ependyma	282.3	244.2	38.12	37	5	0.5072
Microglia vs. Oligodendrocytes	160.4	228	-67.6	8	43	1.113
Microglia vs. Polydendrocytes	160.4	279.4	-119	8	37	1.935
Microglia vs. Astrocytes	160.4	229.5	-69.1	8	23	1.067
Microglia vs. Macrophages	160.4	291.6	-131.2	8	7	1.607
Microglia vs. Neurogenesis	160.4	279.3	-118.9	8	12	1.651
Microglia vs. Ependyma	160.4	244.2	-83.83	8	5	0.932
Oligodendrocytes vs. Polydendrocytes	228	279.4	-51.43	43	37	1.454
Oligodendrocytes vs. Astrocytes	228	229.5	-1.502	43	23	0.03684
Oligodendrocytes vs. Macrophages	228	291.6	-63.59	43	7	0.9891
Oligodendrocytes vs. Neurogenesis	228	279.3	-51.27	43	12	0.9955
Oligodendrocytes vs. Ependyma	228	244.2	-16.22	43	5	0.2176
Polydendrocytes vs. Astrocytes	279.4	229.5	49.93	37	23	1.192
Polydendrocytes vs. Macrophages	279.4	291.6	-12.17	37	7	0.1871
Polydendrocytes vs. Neurogenesis	279.4	279.3	0.1554	37	12	0.002965
Polydendrocytes vs. Ependyma	279.4	244.2	35.21	37	5	0.4684
Astrocytes vs. Macrophages	229.5	291.6	-62.09	23	7	0.9118
Astrocytes vs. Neurogenesis	229.5	279.3	-49.77	23	12	0.8859
Astrocytes vs. Ependyma	229.5	244.2	-14.72	23	5	0.1891
Macrophages vs. Neurogenesis	291.6	279.3	12.32	7	12	0.1642
Macrophages vs. Ependyma	291.6	244.2	47.37	7	5	0.5128
Neurogenesis vs. Ependyma	279.3	244.2	35.05	12	5	0.4174

Maf-cell

Number of families	1
Number of comparisons per family	55
Alpha	0.05

Dunn's multiple comparisons test	Mean rank diff.	Significant?	Summary	Adjusted P Value	
Neurons vs. Endothelial Cells	-26.35	No	ns	>0.9999	A-B
Neurons vs. Fibroblast-like	-243.5	Yes	****	<0.0001	A-C
Neurons vs. Mural Cells	-82.26	No	ns	0.1466	A-D
Neurons vs. Microglia	-277.7	Yes	****	<0.0001	A-E
Neurons vs. Oligodendrocytes	95.28	Yes	*	0.011	A-F
Neurons vs. Polydendrocytes	42.39	No	ns	>0.9999	A-G
Neurons vs. Astrocytes	-103.4	No	ns	0.1304	A-H
Neurons vs. Macrophages	-289.8	Yes	****	<0.0001	A-I
Neurons vs. Neurogenesis	32.37	No	ns	>0.9999	A-J
Neurons vs. Ependyma	-109.5	No	ns	>0.9999	A-K
Endothelial Cells vs. Fibroblast-like	-217.1	Yes	****	<0.0001	B-C
Endothelial Cells vs. Mural Cells	-55.91	No	ns	>0.9999	B-D
Endothelial Cells vs. Microglia	-251.3	Yes	**	0.0027	B-E
Endothelial Cells vs. Oligodendrocytes	121.6	Yes	*	0.0423	B-F
Endothelial Cells vs. Polydendrocytes	68.74	No	ns	>0.9999	B-G

Endothelial Cells vs. Astrocytes	-77.1	No	ns	>0.9999	B-H
Endothelial Cells vs. Macrophages	-263.4	Yes	**	0.0031	B-I
Endothelial Cells vs. Neurogenesis	58.72	No	ns	>0.9999	B-J
Endothelial Cells vs. Ependyma	-83.17	No	ns	>0.9999	B-K
Fibroblast-like vs. Mural Cells	161.2	Yes	**	0.0043	C-D
Fibroblast-like vs. Microglia	-34.19	No	ns	>0.9999	C-E
Fibroblast-like vs. Oligodendrocytes	338.8	Yes	****	<0.0001	C-F
Fibroblast-like vs. Polydendrocytes	285.9	Yes	****	<0.0001	C-G
Fibroblast-like vs. Astrocytes	140	No	ns	0.1153	C-H
Fibroblast-like vs. Macrophages	-46.3	No	ns	>0.9999	C-I
Fibroblast-like vs. Neurogenesis	275.9	Yes	****	<0.0001	C-J
Fibroblast-like vs. Ependyma	134	No	ns	>0.9999	C-K
Mural Cells vs. Microglia	-195.4	No	ns	0.0808	D-E
Mural Cells vs. Oligodendrocytes	177.5	Yes	****	<0.0001	D-F
Mural Cells vs. Polydendrocytes	124.6	Yes	*	0.0367	D-G
Mural Cells vs. Astrocytes	-21.18	No	ns	>0.9999	D-H
Mural Cells vs. Macrophages	-207.5	No	ns	0.0768	D-I
Mural Cells vs. Neurogenesis	114.6	No	ns	>0.9999	D-J
Mural Cells vs. Ependyma	-27.26	No	ns	>0.9999	D-K
Microglia vs. Oligodendrocytes	372.9	Yes	****	<0.0001	E-F
Microglia vs. Polydendrocytes	320.1	Yes	****	<0.0001	E-G
Microglia vs. Astrocytes	174.2	No	ns	0.3884	E-H
Microglia vs. Macrophages	-12.11	No	ns	>0.9999	E-I
Microglia vs. Neurogenesis	310	Yes	***	0.0009	E-J
Microglia vs. Ependyma	168.2	No	ns	>0.9999	E-K
Oligodendrocytes vs. Polydendrocytes	-52.89	No	ns	>0.9999	F-G
Oligodendrocytes vs. Astrocytes	-198.7	Yes	****	<0.0001	F-H
Oligodendrocytes vs. Macrophages	-385.1	Yes	****	<0.0001	F-I
Oligodendrocytes vs. Neurogenesis	-62.91	No	ns	>0.9999	F-J
Oligodendrocytes vs. Ependyma	-204.8	No	ns	0.3269	F-K
Polydendrocytes vs. Astrocytes	-145.8	Yes	*	0.027	G-H
Polydendrocytes vs. Macrophages	-332.2	Yes	****	<0.0001	G-I
Polydendrocytes vs. Neurogenesis	-10.02	No	ns	>0.9999	G-J
Polydendrocytes vs. Ependyma	-151.9	No	ns	>0.9999	G-K
Astrocytes vs. Macrophages	-186.3	No	ns	0.3383	H-I
Astrocytes vs. Neurogenesis	135.8	No	ns	0.8525	H-J
Astrocytes vs. Ependyma	-6.078	No	ns	>0.9999	H-K
Macrophages vs. Neurogenesis	322.1	Yes	***	0.0009	I-J
Macrophages vs. Ependyma	180.3	No	ns	>0.9999	I-K
Neurogenesis vs. Ependyma	-141.9	No	ns	>0.9999	J-K

Test details	Mean rank 1	Mean rank 2	Mean rank diff.	n1	n2	Z
Neurons vs. Endothelial Cells	253.1	279.4	-26.35	315	34	0.9263
Neurons vs. Fibroblast-like	253.1	496.6	-243.5	315	25	7.437
Neurons vs. Mural Cells	253.1	335.3	-82.26	315	37	3.004
Neurons vs. Microglia	253.1	530.8	-277.7	315	8	4.922

Neurons vs. Oligodendrocytes	253.1	157.8	95.28	315	43	3.72
Neurons vs. Polydendrocytes	253.1	210.7	42.39	315	37	1.548
Neurons vs. Astrocytes	253.1	356.5	-103.4	315	23	3.039
Neurons vs. Macrophages	253.1	542.9	-289.8	315	7	4.813
Neurons vs. Neurogenesis	253.1	220.7	32.37	315	12	0.6985
Neurons vs. Ependyma	253.1	362.6	-109.5	315	5	1.542
Endothelial Cells vs. Fibroblast-like	279.4	496.6	-217.1	34	25	5.231
Endothelial Cells vs. Mural Cells	279.4	335.3	-55.91	34	37	1.494
Endothelial Cells vs. Microglia	279.4	530.8	-251.3	34	8	4.059
Endothelial Cells vs. Oligodendrocytes	279.4	157.8	121.6	34	43	3.363
Endothelial Cells vs. Polydendrocytes	279.4	210.7	68.74	34	37	1.836
Endothelial Cells vs. Astrocytes	279.4	356.5	-77.1	34	23	1.812
Endothelial Cells vs. Macrophages	279.4	542.9	-263.4	34	7	4.028
Endothelial Cells vs. Neurogenesis	279.4	220.7	58.72	34	12	1.11
Endothelial Cells vs. Ependyma	279.4	362.6	-83.17	34	5	1.102
Fibroblast-like vs. Mural Cells	496.6	335.3	161.2	25	37	3.952
Fibroblast-like vs. Microglia	496.6	530.8	-34.19	25	8	0.5342
Fibroblast-like vs. Oligodendrocytes	496.6	157.8	338.8	25	43	8.548
Fibroblast-like vs. Polydendrocytes	496.6	210.7	285.9	25	37	7.008
Fibroblast-like vs. Astrocytes	496.6	356.5	140	25	23	3.076
Fibroblast-like vs. Macrophages	496.6	542.9	-46.3	25	7	0.6871
Fibroblast-like vs. Neurogenesis	496.6	220.7	275.9	25	12	4.985
Fibroblast-like vs. Ependyma	496.6	362.6	134	25	5	1.735
Mural Cells vs. Microglia	335.3	530.8	-195.4	37	8	3.181
Mural Cells vs. Oligodendrocytes	335.3	157.8	177.5	37	43	5.025
Mural Cells vs. Polydendrocytes	335.3	210.7	124.6	37	37	3.403
Mural Cells vs. Astrocytes	335.3	356.5	-21.18	37	23	0.5063
Mural Cells vs. Macrophages	335.3	542.9	-207.5	37	7	3.195
Mural Cells vs. Neurogenesis	335.3	220.7	114.6	37	12	2.19
Mural Cells vs. Ependyma	335.3	362.6	-27.26	37	5	0.3631
Microglia vs. Oligodendrocytes	530.8	157.8	372.9	8	43	6.147
Microglia vs. Polydendrocytes	530.8	210.7	320.1	8	37	5.21
Microglia vs. Astrocytes	530.8	356.5	174.2	8	23	2.694
Microglia vs. Macrophages	530.8	542.9	-12.11	8	7	0.1485
Microglia vs. Neurogenesis	530.8	220.7	310	8	12	4.311
Microglia vs. Ependyma	530.8	362.6	168.2	8	5	1.872
Oligodendrocytes vs. Polydendrocytes	157.8	210.7	-52.89	43	37	1.497
Oligodendrocytes vs. Astrocytes	157.8	356.5	-198.7	43	23	4.882
Oligodendrocytes vs. Macrophages	157.8	542.9	-385.1	43	7	5.996
Oligodendrocytes vs. Neurogenesis	157.8	220.7	-62.91	43	12	1.223
Oligodendrocytes vs. Ependyma	157.8	362.6	-204.8	43	5	2.751
Polydendrocytes vs. Astrocytes	210.7	356.5	-145.8	37	23	3.486
Polydendrocytes vs. Macrophages	210.7	542.9	-332.2	37	7	5.115
Polydendrocytes vs. Neurogenesis	210.7	220.7	-10.02	37	12	0.1914
Polydendrocytes vs. Ependyma	210.7	362.6	-151.9	37	5	2.023
Astrocytes vs. Macrophages	356.5	542.9	-186.3	23	7	2.74
Astrocytes vs. Neurogenesis	356.5	220.7	135.8	23	12	2.42

Astrocytes vs. Ependyma	356.5	362.6	-6.078	23	5	0.07818
Macrophages vs. Neurogenesis	542.9	220.7	322.1	7	12	4.299
Macrophages vs. Ependyma	542.9	362.6	180.3	7	5	1.954
Neurogenesis vs. Ependyma	220.7	362.6	-141.9	12	5	1.692

Mafb-cell

Number of families	1
Number of comparisons per family	55
Alpha	0.05

Dunn's multiple comparisons test	Mean rank diff.	Significant?	Summary	Adjusted P Value	
Neurons vs. Endothelial Cells	-21.6	No	ns	>0.9999	A-B
Neurons vs. Fibroblast-like	-146.2	Yes	***	0.0004	A-C
Neurons vs. Mural Cells	-68.27	No	ns	0.6715	A-D
Neurons vs. Microglia	-277.8	Yes	****	<0.0001	A-E
Neurons vs. Oligodendrocytes	123.2	Yes	****	<0.0001	A-F
Neurons vs. Polydendrocytes	15.65	No	ns	>0.9999	A-G
Neurons vs. Astrocytes	-1.273	No	ns	>0.9999	A-H
Neurons vs. Macrophages	-230.4	Yes	**	0.0066	A-I
Neurons vs. Neurogenesis	-85.19	No	ns	>0.9999	A-J
Neurons vs. Ependyma	117.7	No	ns	>0.9999	A-K
Endothelial Cells vs. Fibroblast-like	-124.6	No	ns	0.1401	B-C
Endothelial Cells vs. Mural Cells	-46.68	No	ns	>0.9999	B-D
Endothelial Cells vs. Microglia	-256.2	Yes	**	0.0018	B-E
Endothelial Cells vs. Oligodendrocytes	144.8	Yes	**	0.0031	B-F
Endothelial Cells vs. Polydendrocytes	37.24	No	ns	>0.9999	B-G
Endothelial Cells vs. Astrocytes	20.32	No	ns	>0.9999	B-H
Endothelial Cells vs. Macrophages	-208.8	No	ns	0.0732	B-I
Endothelial Cells vs. Neurogenesis	-63.59	No	ns	>0.9999	B-J
Endothelial Cells vs. Ependyma	139.3	No	ns	>0.9999	B-K
Fibroblast-like vs. Mural Cells	77.96	No	ns	>0.9999	C-D
Fibroblast-like vs. Microglia	-131.5	No	ns	>0.9999	C-E
Fibroblast-like vs. Oligodendrocytes	269.5	Yes	****	<0.0001	C-F
Fibroblast-like vs. Polydendrocytes	161.9	Yes	**	0.0037	C-G
Fibroblast-like vs. Astrocytes	145	No	ns	0.0755	C-H
Fibroblast-like vs. Macrophages	-84.18	No	ns	>0.9999	C-I
Fibroblast-like vs. Neurogenesis	61.04	No	ns	>0.9999	C-J
Fibroblast-like vs. Ependyma	264	Yes	*	0.0323	C-K
Mural Cells vs. Microglia	-209.5	Yes	*	0.0335	D-E
Mural Cells vs. Oligodendrocytes	191.5	Yes	****	<0.0001	D-F
Mural Cells vs. Polydendrocytes	83.92	No	ns	>0.9999	D-G
Mural Cells vs. Astrocytes	67	No	ns	>0.9999	D-H
Mural Cells vs. Macrophages	-162.1	No	ns	0.6651	D-I
Mural Cells vs. Neurogenesis	-16.92	No	ns	>0.9999	D-J
Mural Cells vs. Ependyma	186	No	ns	0.7023	D-K
Microglia vs. Oligodendrocytes	401	Yes	****	<0.0001	E-F

Microglia vs. Polydendrocytes	293.4	Yes	****	<0.0001	E-G
Microglia vs. Astrocytes	276.5	Yes	***	0.001	E-H
Microglia vs. Macrophages	47.36	No	ns	>0.9999	E-I
Microglia vs. Neurogenesis	192.6	No	ns	0.3913	E-J
Microglia vs. Ependyma	395.5	Yes	***	0.0005	E-K
Oligodendrocytes vs. Polydendrocytes	-107.6	No	ns	0.1214	F-G
Oligodendrocytes vs. Astrocytes	-124.5	No	ns	0.1159	F-H
Oligodendrocytes vs. Macrophages	-353.7	Yes	****	<0.0001	F-I
Oligodendrocytes vs. Neurogenesis	-208.4	Yes	**	0.0026	F-J
Oligodendrocytes vs. Ependyma	-5.512	No	ns	>0.9999	F-K
Polydendrocytes vs. Astrocytes	-16.92	No	ns	>0.9999	G-H
Polydendrocytes vs. Macrophages	-246.1	Yes	**	0.0077	G-I
Polydendrocytes vs. Neurogenesis	-100.8	No	ns	>0.9999	G-J
Polydendrocytes vs. Ependyma	102.1	No	ns	>0.9999	G-K
Astrocytes vs. Macrophages	-229.1	Yes	*	0.039	H-I
Astrocytes vs. Neurogenesis	-83.92	No	ns	>0.9999	H-J
Astrocytes vs. Ependyma	119	No	ns	>0.9999	H-K
Macrophages vs. Neurogenesis	145.2	No	ns	>0.9999	I-J
Macrophages vs. Ependyma	348.1	Yes	**	0.0082	I-K
Neurogenesis vs. Ependyma	202.9	No	ns	0.8264	J-K

Test details	Mean rank 1	Mean rank 2	Mean rank diff.	n1	n2	Z
Neurons vs. Endothelial Cells	263.7	285.3	-21.6	315	34	0.7632
Neurons vs. Fibroblast-like	263.7	410	-146.2	315	25	4.489
Neurons vs. Mural Cells	263.7	332	-68.27	315	37	2.506
Neurons vs. Microglia	263.7	541.5	-277.8	315	8	4.949
Neurons vs. Oligodendrocytes	263.7	140.5	123.2	315	43	4.836
Neurons vs. Polydendrocytes	263.7	248.1	15.65	315	37	0.5743
Neurons vs. Astrocytes	263.7	265	-1.273	315	23	0.0376
Neurons vs. Macrophages	263.7	494.1	-230.4	315	7	3.846
Neurons vs. Neurogenesis	263.7	348.9	-85.19	315	12	1.848
Neurons vs. Ependyma	263.7	146	117.7	315	5	1.666
Endothelial Cells vs. Fibroblast-like	285.3	410	-124.6	34	25	3.018
Endothelial Cells vs. Mural Cells	285.3	332	-46.68	34	37	1.253
Endothelial Cells vs. Microglia	285.3	541.5	-256.2	34	8	4.159
Endothelial Cells vs. Oligodendrocytes	285.3	140.5	144.8	34	43	4.026
Endothelial Cells vs. Polydendrocytes	285.3	248.1	37.24	34	37	1
Endothelial Cells vs. Astrocytes	285.3	265	20.32	34	23	0.4802
Endothelial Cells vs. Macrophages	285.3	494.1	-208.8	34	7	3.209
Endothelial Cells vs. Neurogenesis	285.3	348.9	-63.59	34	12	1.208
Endothelial Cells vs. Ependyma	285.3	146	139.3	34	5	1.856
Fibroblast-like vs. Mural Cells	410	332	77.96	25	37	1.921
Fibroblast-like vs. Microglia	410	541.5	-131.5	25	8	2.066
Fibroblast-like vs. Oligodendrocytes	410	140.5	269.5	25	43	6.835
Fibroblast-like vs. Polydendrocytes	410	248.1	161.9	25	37	3.989
Fibroblast-like vs. Astrocytes	410	265	145	25	23	3.2
Fibroblast-like vs. Macrophages	410	494.1	-84.18	25	7	1.256

Fibroblast-like vs. Neurogenesis	410	348.9	61.04	25	12	1.109
Fibroblast-like vs. Ependyma	410	146	264	25	5	3.437
Mural Cells vs. Microglia	332	541.5	-209.5	37	8	3.427
Mural Cells vs. Oligodendrocytes	332	140.5	191.5	37	43	5.448
Mural Cells vs. Polydendrocytes	332	248.1	83.92	37	37	2.302
Mural Cells vs. Astrocytes	332	265	67	37	23	1.61
Mural Cells vs. Macrophages	332	494.1	-162.1	37	7	2.509
Mural Cells vs. Neurogenesis	332	348.9	-16.92	37	12	0.3248
Mural Cells vs. Ependyma	332	146	186	37	5	2.49
Microglia vs. Oligodendrocytes	541.5	140.5	401	8	43	6.644
Microglia vs. Polydendrocytes	541.5	248.1	293.4	8	37	4.8
Microglia vs. Astrocytes	541.5	265	276.5	8	23	4.297
Microglia vs. Macrophages	541.5	494.1	47.36	8	7	0.5837
Microglia vs. Neurogenesis	541.5	348.9	192.6	8	12	2.691
Microglia vs. Ependyma	541.5	146	395.5	8	5	4.425
Oligodendrocytes vs. Polydendrocytes	140.5	248.1	-107.6	43	37	3.061
Oligodendrocytes vs. Astrocytes	140.5	265	-124.5	43	23	3.075
Oligodendrocytes vs. Macrophages	140.5	494.1	-353.7	43	7	5.535
Oligodendrocytes vs. Neurogenesis	140.5	348.9	-208.4	43	12	4.072
Oligodendrocytes vs. Ependyma	140.5	146	-5.512	43	5	0.07441
Polydendrocytes vs. Astrocytes	248.1	265	-16.92	37	23	0.4065
Polydendrocytes vs. Macrophages	248.1	494.1	-246.1	37	7	3.808
Polydendrocytes vs. Neurogenesis	248.1	348.9	-100.8	37	12	1.936
Polydendrocytes vs. Ependyma	248.1	146	102.1	37	5	1.367
Astrocytes vs. Macrophages	265	494.1	-229.1	23	7	3.386
Astrocytes vs. Neurogenesis	265	348.9	-83.92	23	12	1.503
Astrocytes vs. Ependyma	265	146	119	23	5	1.538
Macrophages vs. Neurogenesis	494.1	348.9	145.2	7	12	1.948
Macrophages vs. Ependyma	494.1	146	348.1	7	5	3.793
Neurogenesis vs. Ependyma	348.9	146	202.9	12	5	2.432

Maff-cell

Number of families	1
Number of comparisons per family	55
Alpha	0.05

Dunn's multiple comparisons test	Mean rank diff.	Significant?	Summary	Adjusted P Value	
Neurons vs. Endothelial Cells	-258.8	Yes	****	<0.0001	A-B
Neurons vs. Fibroblast-like	-106.1	Yes	*	0.0173	A-C
Neurons vs. Mural Cells	-42.62	No	ns	>0.9999	A-D
Neurons vs. Microglia	12.65	No	ns	>0.9999	A-E
Neurons vs. Oligodendrocytes	38.93	No	ns	>0.9999	A-F
Neurons vs. Polydendrocytes	27.83	No	ns	>0.9999	A-G
Neurons vs. Astrocytes	25.06	No	ns	>0.9999	A-H
Neurons vs. Macrophages	52.06	No	ns	>0.9999	A-I
Neurons vs. Neurogenesis	-24.93	No	ns	>0.9999	A-J

Neurons vs. Ependyma	-237.8	Yes	*	0.0107	A-K
Endothelial Cells vs. Fibroblast-like	152.7	Yes	**	0.0024	B-C
Endothelial Cells vs. Mural Cells	216.2	Yes	****	<0.0001	B-D
Endothelial Cells vs. Microglia	271.4	Yes	****	<0.0001	B-E
Endothelial Cells vs. Oligodendrocytes	297.7	Yes	****	<0.0001	B-F
Endothelial Cells vs. Polydendrocytes	286.6	Yes	****	<0.0001	B-G
Endothelial Cells vs. Astrocytes	283.8	Yes	****	<0.0001	B-H
Endothelial Cells vs. Macrophages	310.8	Yes	****	<0.0001	B-I
Endothelial Cells vs. Neurogenesis	233.8	Yes	****	<0.0001	B-J
Endothelial Cells vs. Ependyma	20.94	No	ns	>0.9999	B-K
Fibroblast-like vs. Mural Cells	63.45	No	ns	>0.9999	C-D
Fibroblast-like vs. Microglia	118.7	No	ns	>0.9999	C-E
Fibroblast-like vs. Oligodendrocytes	145	Yes	**	0.0026	C-F
Fibroblast-like vs. Polydendrocytes	133.9	Yes	*	0.0144	C-G
Fibroblast-like vs. Astrocytes	131.1	No	ns	0.0746	C-H
Fibroblast-like vs. Macrophages	158.1	No	ns	0.4971	C-I
Fibroblast-like vs. Neurogenesis	81.13	No	ns	>0.9999	C-J
Fibroblast-like vs. Ependyma	-131.8	No	ns	>0.9999	C-K
Mural Cells vs. Microglia	55.27	No	ns	>0.9999	D-E
Mural Cells vs. Oligodendrocytes	81.54	No	ns	0.5638	D-F
Mural Cells vs. Polydendrocytes	70.45	No	ns	>0.9999	D-G
Mural Cells vs. Astrocytes	67.67	No	ns	>0.9999	D-H
Mural Cells vs. Macrophages	94.68	No	ns	>0.9999	D-I
Mural Cells vs. Neurogenesis	17.68	No	ns	>0.9999	D-J
Mural Cells vs. Ependyma	-195.2	No	ns	0.2103	D-K
Microglia vs. Oligodendrocytes	26.28	No	ns	>0.9999	E-F
Microglia vs. Polydendrocytes	15.18	No	ns	>0.9999	E-G
Microglia vs. Astrocytes	12.41	No	ns	>0.9999	E-H
Microglia vs. Macrophages	39.41	No	ns	>0.9999	E-I
Microglia vs. Neurogenesis	-37.58	No	ns	>0.9999	E-J
Microglia vs. Ependyma	-250.5	No	ns	0.1058	E-K
Oligodendrocytes vs. Polydendrocytes	-11.1	No	ns	>0.9999	F-G
Oligodendrocytes vs. Astrocytes	-13.87	No	ns	>0.9999	F-H
Oligodendrocytes vs. Macrophages	13.13	No	ns	>0.9999	F-I
Oligodendrocytes vs. Neurogenesis	-63.86	No	ns	>0.9999	F-J
Oligodendrocytes vs. Ependyma	-276.8	Yes	**	0.002	F-K
Polydendrocytes vs. Astrocytes	-2.771	No	ns	>0.9999	G-H
Polydendrocytes vs. Macrophages	24.23	No	ns	>0.9999	G-I
Polydendrocytes vs. Neurogenesis	-52.76	No	ns	>0.9999	G-J
Polydendrocytes vs. Ependyma	-265.7	Yes	**	0.0046	G-K
Astrocytes vs. Macrophages	27	No	ns	>0.9999	H-I
Astrocytes vs. Neurogenesis	-49.99	No	ns	>0.9999	H-J
Astrocytes vs. Ependyma	-262.9	Yes	**	0.0093	H-K
Macrophages vs. Neurogenesis	-76.99	No	ns	>0.9999	I-J
Macrophages vs. Ependyma	-289.9	Yes	*	0.0261	I-K
Neurogenesis vs. Ependyma	-212.9	No	ns	0.2612	J-K

Test details	Mean rank 1	Mean rank 2	Mean rank diff.	n1	n2	Z
Neurons vs. Endothelial Cells	253.8	512.5	-258.8	315	34	10.12
Neurons vs. Fibroblast-like	253.8	359.8	-106.1	315	25	3.604
Neurons vs. Mural Cells	253.8	296.4	-42.62	315	37	1.731
Neurons vs. Microglia	253.8	241.1	12.65	315	8	0.2495
Neurons vs. Oligodendrocytes	253.8	214.8	38.93	315	43	1.69
Neurons vs. Polydendrocytes	253.8	225.9	27.83	315	37	1.131
Neurons vs. Astrocytes	253.8	228.7	25.06	315	23	0.8191
Neurons vs. Macrophages	253.8	201.7	52.06	315	7	0.9618
Neurons vs. Neurogenesis	253.8	278.7	-24.93	315	12	0.5984
Neurons vs. Ependyma	253.8	491.6	-237.8	315	5	3.725
Endothelial Cells vs. Fibroblast-like	512.5	359.8	152.7	34	25	4.092
Endothelial Cells vs. Mural Cells	512.5	296.4	216.2	34	37	6.423
Endothelial Cells vs. Microglia	512.5	241.1	271.4	34	8	4.876
Endothelial Cells vs. Oligodendrocytes	512.5	214.8	297.7	34	43	9.158
Endothelial Cells vs. Polydendrocytes	512.5	225.9	286.6	34	37	8.517
Endothelial Cells vs. Astrocytes	512.5	228.7	283.8	34	23	7.422
Endothelial Cells vs. Macrophages	512.5	201.7	310.8	34	7	5.287
Endothelial Cells vs. Neurogenesis	512.5	278.7	233.8	34	12	4.917
Endothelial Cells vs. Ependyma	512.5	491.6	20.94	34	5	0.3087
Fibroblast-like vs. Mural Cells	359.8	296.4	63.45	25	37	1.73
Fibroblast-like vs. Microglia	359.8	241.1	118.7	25	8	2.063
Fibroblast-like vs. Oligodendrocytes	359.8	214.8	145	25	43	4.07
Fibroblast-like vs. Polydendrocytes	359.8	225.9	133.9	25	37	3.651
Fibroblast-like vs. Astrocytes	359.8	228.7	131.1	25	23	3.204
Fibroblast-like vs. Macrophages	359.8	201.7	158.1	25	7	2.611
Fibroblast-like vs. Neurogenesis	359.8	278.7	81.13	25	12	1.631
Fibroblast-like vs. Ependyma	359.8	491.6	-131.8	25	5	1.899
Mural Cells vs. Microglia	296.4	241.1	55.27	37	8	1.001
Mural Cells vs. Oligodendrocytes	296.4	214.8	81.54	37	43	2.567
Mural Cells vs. Polydendrocytes	296.4	225.9	70.45	37	37	2.139
Mural Cells vs. Astrocytes	296.4	228.7	67.67	37	23	1.799
Mural Cells vs. Macrophages	296.4	201.7	94.68	37	7	1.622
Mural Cells vs. Neurogenesis	296.4	278.7	17.68	37	12	0.3758
Mural Cells vs. Ependyma	296.4	491.6	-195.2	37	5	2.892
Microglia vs. Oligodendrocytes	241.1	214.8	26.28	8	43	0.4818
Microglia vs. Polydendrocytes	241.1	225.9	15.18	8	37	0.2748
Microglia vs. Astrocytes	241.1	228.7	12.41	8	23	0.2134
Microglia vs. Macrophages	241.1	201.7	39.41	8	7	0.5376
Microglia vs. Neurogenesis	241.1	278.7	-37.58	8	12	0.5813
Microglia vs. Ependyma	241.1	491.6	-250.5	8	5	3.102
Oligodendrocytes vs. Polydendrocytes	214.8	225.9	-11.1	43	37	0.3494
Oligodendrocytes vs. Astrocytes	214.8	228.7	-13.87	43	23	0.379
Oligodendrocytes vs. Macrophages	214.8	201.7	13.13	43	7	0.2275
Oligodendrocytes vs. Neurogenesis	214.8	278.7	-63.86	43	12	1.381
Oligodendrocytes vs. Ependyma	214.8	491.6	-276.8	43	5	4.135
Polydendrocytes vs. Astrocytes	225.9	228.7	-2.771	37	23	0.07369

Polydendrocytes vs. Macrophages	225.9	201.7	24.23	37	7	0.4151
Polydendrocytes vs. Neurogenesis	225.9	278.7	-52.76	37	12	1.121
Polydendrocytes vs. Ependyma	225.9	491.6	-265.7	37	5	3.936
Astrocytes vs. Macrophages	228.7	201.7	27	23	7	0.4416
Astrocytes vs. Neurogenesis	228.7	278.7	-49.99	23	12	0.9911
Astrocytes vs. Ependyma	228.7	491.6	-262.9	23	5	3.761
Macrophages vs. Neurogenesis	201.7	278.7	-76.99	7	12	1.143
Macrophages vs. Ependyma	201.7	491.6	-289.9	7	5	3.495
Neurogenesis vs. Ependyma	278.7	491.6	-212.9	12	5	2.824

Mafg-cell

Number of families	1
Number of comparisons per family	55
Alpha	0.05

Dunn's multiple comparisons test	Mean rank diff.	Significant?	Summary	Adjusted P Value	
Neurons vs. Endothelial Cells	191	Yes	****	<0.0001	A-B
Neurons vs. Fibroblast-like	60.05	No	ns	>0.9999	A-C
Neurons vs. Mural Cells	202.2	Yes	****	<0.0001	A-D
Neurons vs. Microglia	-4.598	No	ns	>0.9999	A-E
Neurons vs. Oligodendrocytes	270.4	Yes	****	<0.0001	A-F
Neurons vs. Polydendrocytes	186.9	Yes	****	<0.0001	A-G
Neurons vs. Astrocytes	225.7	Yes	****	<0.0001	A-H
Neurons vs. Macrophages	48.53	No	ns	>0.9999	A-I
Neurons vs. Neurogenesis	238.1	Yes	****	<0.0001	A-J
Neurons vs. Ependyma	265.3	Yes	*	0.0105	A-K
Endothelial Cells vs. Fibroblast-like	-131	No	ns	0.0896	B-C
Endothelial Cells vs. Mural Cells	11.23	No	ns	>0.9999	B-D
Endothelial Cells vs. Microglia	-195.6	No	ns	0.0882	B-E
Endothelial Cells vs. Oligodendrocytes	79.35	No	ns	>0.9999	B-F
Endothelial Cells vs. Polydendrocytes	-4.119	No	ns	>0.9999	B-G
Endothelial Cells vs. Astrocytes	34.7	No	ns	>0.9999	B-H
Endothelial Cells vs. Macrophages	-142.5	No	ns	>0.9999	B-I
Endothelial Cells vs. Neurogenesis	47.07	No	ns	>0.9999	B-J
Endothelial Cells vs. Ependyma	74.33	No	ns	>0.9999	B-K
Fibroblast-like vs. Mural Cells	142.2	Yes	*	0.0275	C-D
Fibroblast-like vs. Microglia	-64.65	No	ns	>0.9999	C-E
Fibroblast-like vs. Oligodendrocytes	210.3	Yes	****	<0.0001	C-F
Fibroblast-like vs. Polydendrocytes	126.8	No	ns	0.1046	C-G
Fibroblast-like vs. Astrocytes	165.7	Yes	*	0.0153	C-H
Fibroblast-like vs. Macrophages	-11.52	No	ns	>0.9999	C-I
Fibroblast-like vs. Neurogenesis	178	No	ns	0.0722	C-J
Fibroblast-like vs. Ependyma	205.3	No	ns	0.4348	C-K
Mural Cells vs. Microglia	-206.8	Yes	*	0.0425	D-E
Mural Cells vs. Oligodendrocytes	68.12	No	ns	>0.9999	D-F
Mural Cells vs. Polydendrocytes	-15.35	No	ns	>0.9999	D-G

Mural Cells vs. Astrocytes	23.47	No	ns	>0.9999	D-H
Mural Cells vs. Macrophages	-153.7	No	ns	0.9949	D-I
Mural Cells vs. Neurogenesis	35.84	No	ns	>0.9999	D-J
Mural Cells vs. Ependyma	63.1	No	ns	>0.9999	D-K
Microglia vs. Oligodendrocytes	275	Yes	***	0.0003	E-F
Microglia vs. Polydendrocytes	191.5	No	ns	0.1019	E-G
Microglia vs. Astrocytes	230.3	Yes	*	0.0207	E-H
Microglia vs. Macrophages	53.13	No	ns	>0.9999	E-I
Microglia vs. Neurogenesis	242.7	Yes	*	0.0413	E-J
Microglia vs. Ependyma	269.9	No	ns	0.1479	E-K
Oligodendrocytes vs. Polydendrocytes	-83.47	No	ns	>0.9999	F-G
Oligodendrocytes vs. Astrocytes	-44.65	No	ns	>0.9999	F-H
Oligodendrocytes vs. Macrophages	-221.8	Yes	*	0.0308	F-I
Oligodendrocytes vs. Neurogenesis	-32.28	No	ns	>0.9999	F-J
Oligodendrocytes vs. Ependyma	-5.026	No	ns	>0.9999	F-K
Polydendrocytes vs. Astrocytes	38.82	No	ns	>0.9999	G-H
Polydendrocytes vs. Macrophages	-138.4	No	ns	>0.9999	G-I
Polydendrocytes vs. Neurogenesis	51.19	No	ns	>0.9999	G-J
Polydendrocytes vs. Ependyma	78.45	No	ns	>0.9999	G-K
Astrocytes vs. Macrophages	-177.2	No	ns	0.5102	H-I
Astrocytes vs. Neurogenesis	12.37	No	ns	>0.9999	H-J
Astrocytes vs. Ependyma	39.63	No	ns	>0.9999	H-K
Macrophages vs. Neurogenesis	189.5	No	ns	0.6342	I-J
Macrophages vs. Ependyma	216.8	No	ns	>0.9999	I-K
Neurogenesis vs. Ependyma	27.26	No	ns	>0.9999	J-K

Test details	Mean rank 1	Mean rank 2	Mean rank diff.	n1	n2	Z
Neurons vs. Endothelial Cells	353.5	162.5	191	315	34	6.707
Neurons vs. Fibroblast-like	353.5	293.5	60.05	315	25	1.832
Neurons vs. Mural Cells	353.5	151.3	202.2	315	37	7.376
Neurons vs. Microglia	353.5	358.1	-4.598	315	8	0.08141
Neurons vs. Oligodendrocytes	353.5	83.17	270.4	315	43	10.54
Neurons vs. Polydendrocytes	353.5	166.6	186.9	315	37	6.816
Neurons vs. Astrocytes	353.5	127.8	225.7	315	23	6.624
Neurons vs. Macrophages	353.5	305	48.53	315	7	0.8049
Neurons vs. Neurogenesis	353.5	115.5	238.1	315	12	5.131
Neurons vs. Ependyma	353.5	88.2	265.3	315	5	3.731
Endothelial Cells vs. Fibroblast-like	162.5	293.5	-131	34	25	3.151
Endothelial Cells vs. Mural Cells	162.5	151.3	11.23	34	37	0.2997
Endothelial Cells vs. Microglia	162.5	358.1	-195.6	34	8	3.155
Endothelial Cells vs. Oligodendrocytes	162.5	83.17	79.35	34	43	2.192
Endothelial Cells vs. Polydendrocytes	162.5	166.6	-4.119	34	37	0.1099
Endothelial Cells vs. Astrocytes	162.5	127.8	34.7	34	23	0.8148
Endothelial Cells vs. Macrophages	162.5	305	-142.5	34	7	2.176
Endothelial Cells vs. Neurogenesis	162.5	115.5	47.07	34	12	0.8886
Endothelial Cells vs. Ependyma	162.5	88.2	74.33	34	5	0.9837
Fibroblast-like vs. Mural Cells	293.5	151.3	142.2	25	37	3.481

Fibroblast-like vs. Microglia	293.5	358.1	-64.65	25	8	1.009
Fibroblast-like vs. Oligodendrocytes	293.5	83.17	210.3	25	43	5.3
Fibroblast-like vs. Polydendrocytes	293.5	166.6	126.8	25	37	3.105
Fibroblast-like vs. Astrocytes	293.5	127.8	165.7	25	23	3.634
Fibroblast-like vs. Macrophages	293.5	305	-11.52	25	7	0.1708
Fibroblast-like vs. Neurogenesis	293.5	115.5	178	25	12	3.213
Fibroblast-like vs. Ependyma	293.5	88.2	205.3	25	5	2.656
Mural Cells vs. Microglia	151.3	358.1	-206.8	37	8	3.362
Mural Cells vs. Oligodendrocytes	151.3	83.17	68.12	37	43	1.926
Mural Cells vs. Polydendrocytes	151.3	166.6	-15.35	37	37	0.4185
Mural Cells vs. Astrocytes	151.3	127.8	23.47	37	23	0.5603
Mural Cells vs. Macrophages	151.3	305	-153.7	37	7	2.364
Mural Cells vs. Neurogenesis	151.3	115.5	35.84	37	12	0.6838
Mural Cells vs. Ependyma	151.3	88.2	63.1	37	5	0.8394
Microglia vs. Oligodendrocytes	358.1	83.17	275	8	43	4.526
Microglia vs. Polydendrocytes	358.1	166.6	191.5	8	37	3.113
Microglia vs. Astrocytes	358.1	127.8	230.3	8	23	3.556
Microglia vs. Macrophages	358.1	305	53.13	8	7	0.6507
Microglia vs. Neurogenesis	358.1	115.5	242.7	8	12	3.37
Microglia vs. Ependyma	358.1	88.2	269.9	8	5	3.001
Oligodendrocytes vs. Polydendrocytes	83.17	166.6	-83.47	43	37	2.36
Oligodendrocytes vs. Astrocytes	83.17	127.8	-44.65	43	23	1.096
Oligodendrocytes vs. Macrophages	83.17	305	-221.8	43	7	3.45
Oligodendrocytes vs. Neurogenesis	83.17	115.5	-32.28	43	12	0.6268
Oligodendrocytes vs. Ependyma	83.17	88.2	-5.026	43	5	0.06742
Polydendrocytes vs. Astrocytes	166.6	127.8	38.82	37	23	0.9268
Polydendrocytes vs. Macrophages	166.6	305	-138.4	37	7	2.128
Polydendrocytes vs. Neurogenesis	166.6	115.5	51.19	37	12	0.9767
Polydendrocytes vs. Ependyma	166.6	88.2	78.45	37	5	1.044
Astrocytes vs. Macrophages	127.8	305	-177.2	23	7	2.602
Astrocytes vs. Neurogenesis	127.8	115.5	12.37	23	12	0.2201
Astrocytes vs. Ependyma	127.8	88.2	39.63	23	5	0.509
Macrophages vs. Neurogenesis	305	115.5	189.5	7	12	2.526
Macrophages vs. Ependyma	305	88.2	216.8	7	5	2.347
Neurogenesis vs. Ependyma	115.5	88.2	27.26	12	5	0.3246

Mafk-cell

Number of families	1
Number of comparisons per family	55
Alpha	0.05

Dunn's multiple comparisons test	Mean rank diff.	Significant?	Summary	Adjusted P Value	
Neurons vs. Endothelial Cells	-262	Yes	****	<0.0001	A-B
Neurons vs. Fibroblast-like	-75.96	No	ns	>0.9999	A-C
Neurons vs. Mural Cells	-237.9	Yes	****	<0.0001	A-D
Neurons vs. Microglia	-101.2	No	ns	>0.9999	A-E

Neurons vs. Oligodendrocytes	-103.1	Yes	**		0.003	A-F
Neurons vs. Polydendrocytes	54.76	No	ns	>0.9999		A-G
Neurons vs. Astrocytes	90.81	No	ns		0.4095	A-H
Neurons vs. Macrophages	-36.46	No	ns	>0.9999		A-I
Neurons vs. Neurogenesis	104.5	No	ns	>0.9999		A-J
Neurons vs. Ependyma	36.68	No	ns	>0.9999		A-K
Endothelial Cells vs. Fibroblast-like	186	Yes	***		0.0004	B-C
Endothelial Cells vs. Mural Cells	24.05	No	ns	>0.9999		B-D
Endothelial Cells vs. Microglia	160.8	No	ns		0.5055	B-E
Endothelial Cells vs. Oligodendrocytes	158.9	Yes	***		0.0006	B-F
Endothelial Cells vs. Polydendrocytes	316.8	Yes	****	<0.0001		B-G
Endothelial Cells vs. Astrocytes	352.8	Yes	****	<0.0001		B-H
Endothelial Cells vs. Macrophages	225.5	Yes	*		0.0298	B-I
Endothelial Cells vs. Neurogenesis	366.5	Yes	****	<0.0001		B-J
Endothelial Cells vs. Ependyma	298.7	Yes	**		0.004	B-K
Fibroblast-like vs. Mural Cells	-162	Yes	**		0.0037	C-D
Fibroblast-like vs. Microglia	-25.24	No	ns	>0.9999		C-E
Fibroblast-like vs. Oligodendrocytes	-27.17	No	ns	>0.9999		C-F
Fibroblast-like vs. Polydendrocytes	130.7	No	ns		0.072	C-G
Fibroblast-like vs. Astrocytes	166.8	Yes	*		0.0131	C-H
Fibroblast-like vs. Macrophages	39.5	No	ns	>0.9999		C-I
Fibroblast-like vs. Neurogenesis	180.5	No	ns		0.0589	C-J
Fibroblast-like vs. Ependyma	112.6	No	ns	>0.9999		C-K
Mural Cells vs. Microglia	136.7	No	ns	>0.9999		D-E
Mural Cells vs. Oligodendrocytes	134.8	Yes	**		0.0071	D-F
Mural Cells vs. Polydendrocytes	292.7	Yes	****	<0.0001		D-G
Mural Cells vs. Astrocytes	328.8	Yes	****	<0.0001		D-H
Mural Cells vs. Macrophages	201.5	No	ns		0.1023	D-I
Mural Cells vs. Neurogenesis	342.5	Yes	****	<0.0001		D-J
Mural Cells vs. Ependyma	274.6	Yes	*		0.0134	D-K
Microglia vs. Oligodendrocytes	-1.939	No	ns	>0.9999		E-F
Microglia vs. Polydendrocytes	156	No	ns		0.5991	E-G
Microglia vs. Astrocytes	192	No	ns		0.1598	E-H
Microglia vs. Macrophages	64.73	No	ns	>0.9999		E-I
Microglia vs. Neurogenesis	205.7	No	ns		0.2266	E-J
Microglia vs. Ependyma	137.9	No	ns	>0.9999		E-K
Oligodendrocytes vs. Polydendrocytes	157.9	Yes	***		0.0004	F-G
Oligodendrocytes vs. Astrocytes	193.9	Yes	****	<0.0001		F-H
Oligodendrocytes vs. Macrophages	66.67	No	ns	>0.9999		F-I
Oligodendrocytes vs. Neurogenesis	207.6	Yes	**		0.0028	F-J
Oligodendrocytes vs. Ependyma	139.8	No	ns	>0.9999		F-K
Polydendrocytes vs. Astrocytes	36.05	No	ns	>0.9999		G-H
Polydendrocytes vs. Macrophages	-91.22	No	ns	>0.9999		G-I
Polydendrocytes vs. Neurogenesis	49.75	No	ns	>0.9999		G-J
Polydendrocytes vs. Ependyma	-18.08	No	ns	>0.9999		G-K
Astrocytes vs. Macrophages	-127.3	No	ns	>0.9999		H-I
Astrocytes vs. Neurogenesis	13.7	No	ns	>0.9999		H-J

Astrocytes vs. Ependyma	-54.13	No	ns	>0.9999	H-K	
Macrophages vs. Neurogenesis	141	No	ns	>0.9999	I-J	
Macrophages vs. Ependyma	73.14	No	ns	>0.9999	I-K	
Neurogenesis vs. Ependyma	-67.83	No	ns	>0.9999	J-K	

Test details	Mean rank 1	Mean rank 2	Mean rank diff.	n1	n2	Z
Neurons vs. Endothelial Cells	237.7	499.7	-262	315	34	9.239
Neurons vs. Fibroblast-like	237.7	313.6	-75.96	315	25	2.327
Neurons vs. Mural Cells	237.7	475.6	-237.9	315	37	8.715
Neurons vs. Microglia	237.7	338.9	-101.2	315	8	1.799
Neurons vs. Oligodendrocytes	237.7	340.8	-103.1	315	43	4.038
Neurons vs. Polydendrocytes	237.7	182.9	54.76	315	37	2.006
Neurons vs. Astrocytes	237.7	146.9	90.81	315	23	2.676
Neurons vs. Macrophages	237.7	274.1	-36.46	315	7	0.6074
Neurons vs. Neurogenesis	237.7	133.2	104.5	315	12	2.262
Neurons vs. Ependyma	237.7	201	36.68	315	5	0.518
Endothelial Cells vs. Fibroblast-like	499.7	313.6	186	34	25	4.495
Endothelial Cells vs. Mural Cells	499.7	475.6	24.05	34	37	0.6445
Endothelial Cells vs. Microglia	499.7	338.9	160.8	34	8	2.605
Endothelial Cells vs. Oligodendrocytes	499.7	340.8	158.9	34	43	4.406
Endothelial Cells vs. Polydendrocytes	499.7	182.9	316.8	34	37	8.487
Endothelial Cells vs. Astrocytes	499.7	146.9	352.8	34	23	8.318
Endothelial Cells vs. Macrophages	499.7	274.1	225.5	34	7	3.459
Endothelial Cells vs. Neurogenesis	499.7	133.2	366.5	34	12	6.948
Endothelial Cells vs. Ependyma	499.7	201	298.7	34	5	3.969
Fibroblast-like vs. Mural Cells	313.6	475.6	-162	25	37	3.983
Fibroblast-like vs. Microglia	313.6	338.9	-25.24	25	8	0.3955
Fibroblast-like vs. Oligodendrocytes	313.6	340.8	-27.17	25	43	0.6878
Fibroblast-like vs. Polydendrocytes	313.6	182.9	130.7	25	37	3.214
Fibroblast-like vs. Astrocytes	313.6	146.9	166.8	25	23	3.674
Fibroblast-like vs. Macrophages	313.6	274.1	39.5	25	7	0.588
Fibroblast-like vs. Neurogenesis	313.6	133.2	180.5	25	12	3.271
Fibroblast-like vs. Ependyma	313.6	201	112.6	25	5	1.464
Mural Cells vs. Microglia	475.6	338.9	136.7	37	8	2.232
Mural Cells vs. Oligodendrocytes	475.6	340.8	134.8	37	43	3.827
Mural Cells vs. Polydendrocytes	475.6	182.9	292.7	37	37	8.014
Mural Cells vs. Astrocytes	475.6	146.9	328.8	37	23	7.881
Mural Cells vs. Macrophages	475.6	274.1	201.5	37	7	3.112
Mural Cells vs. Neurogenesis	475.6	133.2	342.5	37	12	6.562
Mural Cells vs. Ependyma	475.6	201	274.6	37	5	3.669
Microglia vs. Oligodendrocytes	338.9	340.8	-1.939	8	43	0.03205
Microglia vs. Polydendrocytes	338.9	182.9	156	8	37	2.546
Microglia vs. Astrocytes	338.9	146.9	192	8	23	2.978
Microglia vs. Macrophages	338.9	274.1	64.73	8	7	0.7962
Microglia vs. Neurogenesis	338.9	133.2	205.7	8	12	2.869
Microglia vs. Ependyma	338.9	201	137.9	8	5	1.539
Oligodendrocytes vs. Polydendrocytes	340.8	182.9	157.9	43	37	4.482

Oligodendrocytes vs. Astrocytes	340.8	146.9	193.9	43	23	4.779
Oligodendrocytes vs. Macrophages	340.8	274.1	66.67	43	7	1.041
Oligodendrocytes vs. Neurogenesis	340.8	133.2	207.6	43	12	4.049
Oligodendrocytes vs. Ependyma	340.8	201	139.8	43	5	1.884
Polydendrocytes vs. Astrocytes	182.9	146.9	36.05	37	23	0.8642
Polydendrocytes vs. Macrophages	182.9	274.1	-91.22	37	7	1.409
Polydendrocytes vs. Neurogenesis	182.9	133.2	49.75	37	12	0.9533
Polydendrocytes vs. Ependyma	182.9	201	-18.08	37	5	0.2416
Astrocytes vs. Macrophages	146.9	274.1	-127.3	23	7	1.877
Astrocytes vs. Neurogenesis	146.9	133.2	13.7	23	12	0.2449
Astrocytes vs. Ependyma	146.9	201	-54.13	23	5	0.6983
Macrophages vs. Neurogenesis	274.1	133.2	141	7	12	1.887
Macrophages vs. Ependyma	274.1	201	73.14	7	5	0.7951
Neurogenesis vs. Ependyma	133.2	201	-67.83	12	5	0.8112

Cul3-cell

Number of families	1
Number of comparisons per family	55
Alpha	0.05

Dunn's multiple comparisons test	Mean rank diff.	Significant?	Summary	Adjusted P Value	
Neurons vs. Endothelial Cells	165.8	Yes	****	<0.0001	A-B
Neurons vs. Fibroblast-like	163.3	Yes	****	<0.0001	A-C
Neurons vs. Mural Cells	269	Yes	****	<0.0001	A-D
Neurons vs. Microglia	135.3	No	ns	0.9149	A-E
Neurons vs. Oligodendrocytes	290.7	Yes	****	<0.0001	A-F
Neurons vs. Polydendrocytes	214	Yes	****	<0.0001	A-G
Neurons vs. Astrocytes	205.6	Yes	****	<0.0001	A-H
Neurons vs. Macrophages	250.7	Yes	**	0.0018	A-I
Neurons vs. Neurogenesis	286.7	Yes	****	<0.0001	A-J
Neurons vs. Ependyma	250.8	Yes	*	0.0232	A-K
Endothelial Cells vs. Fibroblast-like	-2.511	No	ns	>0.9999	B-C
Endothelial Cells vs. Mural Cells	103.1	No	ns	0.3263	B-D
Endothelial Cells vs. Microglia	-30.6	No	ns	>0.9999	B-E
Endothelial Cells vs. Oligodendrocytes	124.8	Yes	*	0.0311	B-F
Endothelial Cells vs. Polydendrocytes	48.15	No	ns	>0.9999	B-G
Endothelial Cells vs. Astrocytes	39.7	No	ns	>0.9999	B-H
Endothelial Cells vs. Macrophages	84.82	No	ns	>0.9999	B-I
Endothelial Cells vs. Neurogenesis	120.9	No	ns	>0.9999	B-J
Endothelial Cells vs. Ependyma	84.93	No	ns	>0.9999	B-K
Fibroblast-like vs. Mural Cells	105.6	No	ns	0.5335	C-D
Fibroblast-like vs. Microglia	-28.09	No	ns	>0.9999	C-E
Fibroblast-like vs. Oligodendrocytes	127.3	No	ns	0.0733	C-F
Fibroblast-like vs. Polydendrocytes	50.66	No	ns	>0.9999	C-G
Fibroblast-like vs. Astrocytes	42.21	No	ns	>0.9999	C-H
Fibroblast-like vs. Macrophages	87.33	No	ns	>0.9999	C-I

Fibroblast-like vs. Neurogenesis	123.4	No	ns	>0.9999	C-J
Fibroblast-like vs. Ependyma	87.44	No	ns	>0.9999	C-K
Mural Cells vs. Microglia	-133.7	No	ns	>0.9999	D-E
Mural Cells vs. Oligodendrocytes	21.68	No	ns	>0.9999	D-F
Mural Cells vs. Polydendrocytes	-54.97	No	ns	>0.9999	D-G
Mural Cells vs. Astrocytes	-63.42	No	ns	>0.9999	D-H
Mural Cells vs. Macrophages	-18.31	No	ns	>0.9999	D-I
Mural Cells vs. Neurogenesis	17.74	No	ns	>0.9999	D-J
Mural Cells vs. Ependyma	-18.19	No	ns	>0.9999	D-K
Microglia vs. Oligodendrocytes	155.4	No	ns	0.5785	E-F
Microglia vs. Polydendrocytes	78.75	No	ns	>0.9999	E-G
Microglia vs. Astrocytes	70.3	No	ns	>0.9999	E-H
Microglia vs. Macrophages	115.4	No	ns	>0.9999	E-I
Microglia vs. Neurogenesis	151.5	No	ns	>0.9999	E-J
Microglia vs. Ependyma	115.5	No	ns	>0.9999	E-K
Oligodendrocytes vs. Polydendrocytes	-76.66	No	ns	>0.9999	F-G
Oligodendrocytes vs. Astrocytes	-85.11	No	ns	>0.9999	F-H
Oligodendrocytes vs. Macrophages	-39.99	No	ns	>0.9999	F-I
Oligodendrocytes vs. Neurogenesis	-3.946	No	ns	>0.9999	F-J
Oligodendrocytes vs. Ependyma	-39.88	No	ns	>0.9999	F-K
Polydendrocytes vs. Astrocytes	-8.448	No	ns	>0.9999	G-H
Polydendrocytes vs. Macrophages	36.66	No	ns	>0.9999	G-I
Polydendrocytes vs. Neurogenesis	72.71	No	ns	>0.9999	G-J
Polydendrocytes vs. Ependyma	36.78	No	ns	>0.9999	G-K
Astrocytes vs. Macrophages	45.11	No	ns	>0.9999	H-I
Astrocytes vs. Neurogenesis	81.16	No	ns	>0.9999	H-J
Astrocytes vs. Ependyma	45.23	No	ns	>0.9999	H-K
Macrophages vs. Neurogenesis	36.05	No	ns	>0.9999	I-J
Macrophages vs. Ependyma	0.1143	No	ns	>0.9999	I-K
Neurogenesis vs. Ependyma	-35.93	No	ns	>0.9999	J-K

Test details	Mean rank 1	Mean rank 2	Mean rank diff.	n1	n2	Z
Neurons vs. Endothelial Cells	369.4	203.5	165.8	315	34	5.824
Neurons vs. Fibroblast-like	369.4	206	163.3	315	25	4.983
Neurons vs. Mural Cells	369.4	100.4	269	315	37	9.811
Neurons vs. Microglia	369.4	234.1	135.3	315	8	2.395
Neurons vs. Oligodendrocytes	369.4	78.72	290.7	315	43	11.33
Neurons vs. Polydendrocytes	369.4	155.4	214	315	37	7.805
Neurons vs. Astrocytes	369.4	163.8	205.6	315	23	6.032
Neurons vs. Macrophages	369.4	118.7	250.7	315	7	4.158
Neurons vs. Neurogenesis	369.4	82.67	286.7	315	12	6.179
Neurons vs. Ependyma	369.4	118.6	250.8	315	5	3.527
Endothelial Cells vs. Fibroblast-like	203.5	206	-2.511	34	25	0.0604
Endothelial Cells vs. Mural Cells	203.5	100.4	103.1	34	37	2.752
Endothelial Cells vs. Microglia	203.5	234.1	-30.6	34	8	0.4935
Endothelial Cells vs. Oligodendrocytes	203.5	78.72	124.8	34	43	3.447
Endothelial Cells vs. Polydendrocytes	203.5	155.4	48.15	34	37	1.285

Endothelial Cells vs. Astrocytes	203.5	163.8	39.7	34	23	0.9322
Endothelial Cells vs. Macrophages	203.5	118.7	84.82	34	7	1.295
Endothelial Cells vs. Neurogenesis	203.5	82.67	120.9	34	12	2.282
Endothelial Cells vs. Ependyma	203.5	118.6	84.93	34	5	1.124
Fibroblast-like vs. Mural Cells	206	100.4	105.6	25	37	2.586
Fibroblast-like vs. Microglia	206	234.1	-28.09	25	8	0.4383
Fibroblast-like vs. Oligodendrocytes	206	78.72	127.3	25	43	3.209
Fibroblast-like vs. Polydendrocytes	206	155.4	50.66	25	37	1.24
Fibroblast-like vs. Astrocytes	206	163.8	42.21	25	23	0.9261
Fibroblast-like vs. Macrophages	206	118.7	87.33	25	7	1.294
Fibroblast-like vs. Neurogenesis	206	82.67	123.4	25	12	2.227
Fibroblast-like vs. Ependyma	206	118.6	87.44	25	5	1.131
Mural Cells vs. Microglia	100.4	234.1	-133.7	37	8	2.174
Mural Cells vs. Oligodendrocytes	100.4	78.72	21.68	37	43	0.613
Mural Cells vs. Polydendrocytes	100.4	155.4	-54.97	37	37	1.499
Mural Cells vs. Astrocytes	100.4	163.8	-63.42	37	23	1.514
Mural Cells vs. Macrophages	100.4	118.7	-18.31	37	7	0.2816
Mural Cells vs. Neurogenesis	100.4	82.67	17.74	37	12	0.3385
Mural Cells vs. Ependyma	100.4	118.6	-18.19	37	5	0.242
Microglia vs. Oligodendrocytes	234.1	78.72	155.4	8	43	2.558
Microglia vs. Polydendrocytes	234.1	155.4	78.75	8	37	1.28
Microglia vs. Astrocytes	234.1	163.8	70.3	8	23	1.086
Microglia vs. Macrophages	234.1	118.7	115.4	8	7	1.413
Microglia vs. Neurogenesis	234.1	82.67	151.5	8	12	2.103
Microglia vs. Ependyma	234.1	118.6	115.5	8	5	1.285
Oligodendrocytes vs. Polydendrocytes	78.72	155.4	-76.66	43	37	2.167
Oligodendrocytes vs. Astrocytes	78.72	163.8	-85.11	43	23	2.088
Oligodendrocytes vs. Macrophages	78.72	118.7	-39.99	43	7	0.622
Oligodendrocytes vs. Neurogenesis	78.72	82.67	-3.946	43	12	0.07661
Oligodendrocytes vs. Ependyma	78.72	118.6	-39.88	43	5	0.535
Polydendrocytes vs. Astrocytes	155.4	163.8	-8.448	37	23	0.2017
Polydendrocytes vs. Macrophages	155.4	118.7	36.66	37	7	0.5639
Polydendrocytes vs. Neurogenesis	155.4	82.67	72.71	37	12	1.387
Polydendrocytes vs. Ependyma	155.4	118.6	36.78	37	5	0.4893
Astrocytes vs. Macrophages	163.8	118.7	45.11	23	7	0.6624
Astrocytes vs. Neurogenesis	163.8	82.67	81.16	23	12	1.445
Astrocytes vs. Ependyma	163.8	118.6	45.23	23	5	0.581
Macrophages vs. Neurogenesis	118.7	82.67	36.05	7	12	0.4804
Macrophages vs. Ependyma	118.7	118.6	0.1143	7	5	0.001237
Neurogenesis vs. Ependyma	82.67	118.6	-35.93	12	5	0.4279

Nqo-Nfe2l2-cell

Number of families	1
Number of comparisons per family	55
Alpha	0.05

Dunn's multiple comparisons test	Mean rank diff.	Significant?	Summary	Adjusted P Value	
Neurons vs. Endothelial Cells	-316.2	Yes	****	<0.0001	A-B
Neurons vs. Fibroblast-like	-339.2	Yes	****	<0.0001	A-C
Neurons vs. Mural Cells	-226.7	Yes	****	<0.0001	A-D
Neurons vs. Microglia	-353	Yes	****	<0.0001	A-E
Neurons vs. Oligodendrocytes	-229.7	Yes	****	<0.0001	A-F
Neurons vs. Polydendrocytes	-168.4	Yes	****	<0.0001	A-G
Neurons vs. Astrocytes	-334.3	Yes	****	<0.0001	A-H
Neurons vs. Macrophages	-363.6	Yes	****	<0.0001	A-I
Neurons vs. Neurogenesis	-198.4	Yes	**	0.001	A-J
Neurons vs. Ependyma	-263.5	Yes	*	0.0113	A-K
Endothelial Cells vs. Fibroblast-like	-23.02	No	ns	>0.9999	B-C
Endothelial Cells vs. Mural Cells	89.5	No	ns	0.9202	B-D
Endothelial Cells vs. Microglia	-36.81	No	ns	>0.9999	B-E
Endothelial Cells vs. Oligodendrocytes	86.55	No	ns	0.9138	B-F
Endothelial Cells vs. Polydendrocytes	147.8	Yes	**	0.0043	B-G
Endothelial Cells vs. Astrocytes	-18.06	No	ns	>0.9999	B-H
Endothelial Cells vs. Macrophages	-47.34	No	ns	>0.9999	B-I
Endothelial Cells vs. Neurogenesis	117.8	No	ns	>0.9999	B-J
Endothelial Cells vs. Ependyma	52.74	No	ns	>0.9999	B-K
Fibroblast-like vs. Mural Cells	112.5	No	ns	0.3178	C-D
Fibroblast-like vs. Microglia	-13.79	No	ns	>0.9999	C-E
Fibroblast-like vs. Oligodendrocytes	109.6	No	ns	0.3114	C-F
Fibroblast-like vs. Polydendrocytes	170.8	Yes	**	0.0015	C-G
Fibroblast-like vs. Astrocytes	4.96	No	ns	>0.9999	C-H
Fibroblast-like vs. Macrophages	-24.33	No	ns	>0.9999	C-I
Fibroblast-like vs. Neurogenesis	140.8	No	ns	0.5977	C-J
Fibroblast-like vs. Ependyma	75.76	No	ns	>0.9999	C-K
Mural Cells vs. Microglia	-126.3	No	ns	>0.9999	D-E
Mural Cells vs. Oligodendrocytes	-2.949	No	ns	>0.9999	D-F
Mural Cells vs. Polydendrocytes	58.28	No	ns	>0.9999	D-G
Mural Cells vs. Astrocytes	-107.6	No	ns	0.5552	D-H
Mural Cells vs. Macrophages	-136.8	No	ns	>0.9999	D-I
Mural Cells vs. Neurogenesis	28.32	No	ns	>0.9999	D-J
Mural Cells vs. Ependyma	-36.75	No	ns	>0.9999	D-K
Microglia vs. Oligodendrocytes	123.4	No	ns	>0.9999	E-F
Microglia vs. Polydendrocytes	184.6	No	ns	0.1452	E-G
Microglia vs. Astrocytes	18.75	No	ns	>0.9999	E-H
Microglia vs. Macrophages	-10.54	No	ns	>0.9999	E-I
Microglia vs. Neurogenesis	154.6	No	ns	>0.9999	E-J
Microglia vs. Ependyma	89.55	No	ns	>0.9999	E-K
Oligodendrocytes vs. Polydendrocytes	61.23	No	ns	>0.9999	F-G
Oligodendrocytes vs. Astrocytes	-104.6	No	ns	0.5565	F-H
Oligodendrocytes vs. Macrophages	-133.9	No	ns	>0.9999	F-I
Oligodendrocytes vs. Neurogenesis	31.27	No	ns	>0.9999	F-J
Oligodendrocytes vs. Ependyma	-33.8	No	ns	>0.9999	F-K
Polydendrocytes vs. Astrocytes	-165.8	Yes	**	0.004	G-H

Polydendrocytes vs. Macrophages	-195.1	No	ns	0.1453	G-I
Polydendrocytes vs. Neurogenesis	-29.96	No	ns	>0.9999	G-J
Polydendrocytes vs. Ependyma	-95.04	No	ns	>0.9999	G-K
Astrocytes vs. Macrophages	-29.29	No	ns	>0.9999	H-I
Astrocytes vs. Neurogenesis	135.9	No	ns	0.8459	H-J
Astrocytes vs. Ependyma	70.8	No	ns	>0.9999	H-K
Macrophages vs. Neurogenesis	165.2	No	ns	>0.9999	I-J
Macrophages vs. Ependyma	100.1	No	ns	>0.9999	I-K
Neurogenesis vs. Ependyma	-65.08	No	ns	>0.9999	J-K

Test details	Mean rank 1	Mean rank 2	Mean rank diff.	n1	n2	Z
Neurons vs. Endothelial Cells	162.7	478.9	-316.2	315	34	11.13
Neurons vs. Fibroblast-like	162.7	502	-339.2	315	25	10.37
Neurons vs. Mural Cells	162.7	389.4	-226.7	315	37	8.286
Neurons vs. Microglia	162.7	515.8	-353	315	8	6.263
Neurons vs. Oligodendrocytes	162.7	392.4	-229.7	315	43	8.972
Neurons vs. Polydendrocytes	162.7	331.2	-168.4	315	37	6.156
Neurons vs. Astrocytes	162.7	497	-334.3	315	23	9.829
Neurons vs. Macrophages	162.7	526.3	-363.6	315	7	6.042
Neurons vs. Neurogenesis	162.7	361.1	-198.4	315	12	4.284
Neurons vs. Ependyma	162.7	426.2	-263.5	315	5	3.712
Endothelial Cells vs. Fibroblast-like	478.9	502	-23.02	34	25	0.5549
Endothelial Cells vs. Mural Cells	478.9	389.4	89.5	34	37	2.393
Endothelial Cells vs. Microglia	478.9	515.8	-36.81	34	8	0.5949
Endothelial Cells vs. Oligodendrocytes	478.9	392.4	86.55	34	43	2.395
Endothelial Cells vs. Polydendrocytes	478.9	331.2	147.8	34	37	3.951
Endothelial Cells vs. Astrocytes	478.9	497	-18.06	34	23	0.4248
Endothelial Cells vs. Macrophages	478.9	526.3	-47.34	34	7	0.7245
Endothelial Cells vs. Neurogenesis	478.9	361.1	117.8	34	12	2.228
Endothelial Cells vs. Ependyma	478.9	426.2	52.74	34	5	0.6993
Fibroblast-like vs. Mural Cells	502	389.4	112.5	25	37	2.76
Fibroblast-like vs. Microglia	502	515.8	-13.79	25	8	0.2156
Fibroblast-like vs. Oligodendrocytes	502	392.4	109.6	25	43	2.767
Fibroblast-like vs. Polydendrocytes	502	331.2	170.8	25	37	4.19
Fibroblast-like vs. Astrocytes	502	497	4.96	25	23	0.109
Fibroblast-like vs. Macrophages	502	526.3	-24.33	25	7	0.3613
Fibroblast-like vs. Neurogenesis	502	361.1	140.8	25	12	2.547
Fibroblast-like vs. Ependyma	502	426.2	75.76	25	5	0.9822
Mural Cells vs. Microglia	389.4	515.8	-126.3	37	8	2.057
Mural Cells vs. Oligodendrocytes	389.4	392.4	-2.949	37	43	0.08354
Mural Cells vs. Polydendrocytes	389.4	331.2	58.28	37	37	1.592
Mural Cells vs. Astrocytes	389.4	497	-107.6	37	23	2.573
Mural Cells vs. Macrophages	389.4	526.3	-136.8	37	7	2.109
Mural Cells vs. Neurogenesis	389.4	361.1	28.32	37	12	0.5414
Mural Cells vs. Ependyma	389.4	426.2	-36.75	37	5	0.4899
Microglia vs. Oligodendrocytes	515.8	392.4	123.4	8	43	2.035
Microglia vs. Polydendrocytes	515.8	331.2	184.6	8	37	3.007

Microglia vs. Astrocytes	515.8	497	18.75	8	23	0.2901
Microglia vs. Macrophages	515.8	526.3	-10.54	8	7	0.1293
Microglia vs. Neurogenesis	515.8	361.1	154.6	8	12	2.152
Microglia vs. Ependyma	515.8	426.2	89.55	8	5	0.9976
Oligodendrocytes vs. Polydendrocytes	392.4	331.2	61.23	43	37	1.734
Oligodendrocytes vs. Astrocytes	392.4	497	-104.6	43	23	2.572
Oligodendrocytes vs. Macrophages	392.4	526.3	-133.9	43	7	2.086
Oligodendrocytes vs. Neurogenesis	392.4	361.1	31.27	43	12	0.6083
Oligodendrocytes vs. Ependyma	392.4	426.2	-33.8	43	5	0.4544
Polydendrocytes vs. Astrocytes	331.2	497	-165.8	37	23	3.967
Polydendrocytes vs. Macrophages	331.2	526.3	-195.1	37	7	3.007
Polydendrocytes vs. Neurogenesis	331.2	361.1	-29.96	37	12	0.5728
Polydendrocytes vs. Ependyma	331.2	426.2	-95.04	37	5	1.267
Astrocytes vs. Macrophages	497	526.3	-29.29	23	7	0.4309
Astrocytes vs. Neurogenesis	497	361.1	135.9	23	12	2.423
Astrocytes vs. Ependyma	497	426.2	70.8	23	5	0.9113
Macrophages vs. Neurogenesis	526.3	361.1	165.2	7	12	2.206
Macrophages vs. Ependyma	526.3	426.2	100.1	7	5	1.086
Neurogenesis vs. Ependyma	361.1	426.2	-65.08	12	5	0.7765

Gstm1-Nfe2l2-cell

Number of families	1
Number of comparisons per family	55
Alpha	0.05

Dunn's multiple comparisons test	Mean rank diff.	Significant?	Summary	Adjusted P Value	
Neurons vs. Endothelial Cells	-312.1	Yes	****	<0.0001	A-B
Neurons vs. Fibroblast-like	-332.5	Yes	****	<0.0001	A-C
Neurons vs. Mural Cells	-246.6	Yes	****	<0.0001	A-D
Neurons vs. Microglia	-338	Yes	****	<0.0001	A-E
Neurons vs. Oligodendrocytes	-232	Yes	****	<0.0001	A-F
Neurons vs. Polydendrocytes	-174.4	Yes	****	<0.0001	A-G
Neurons vs. Astrocytes	-357.9	Yes	****	<0.0001	A-H
Neurons vs. Macrophages	-347.8	Yes	****	<0.0001	A-I
Neurons vs. Neurogenesis	-219.7	Yes	***	0.0001	A-J
Neurons vs. Ependyma	-342.4	Yes	****	<0.0001	A-K
Endothelial Cells vs. Fibroblast-like	-20.44	No	ns	>0.9999	B-C
Endothelial Cells vs. Mural Cells	65.49	No	ns	>0.9999	B-D
Endothelial Cells vs. Microglia	-25.95	No	ns	>0.9999	B-E
Endothelial Cells vs. Oligodendrocytes	80.1	No	ns	>0.9999	B-F
Endothelial Cells vs. Polydendrocytes	137.7	Yes	*	0.0131	B-G
Endothelial Cells vs. Astrocytes	-45.8	No	ns	>0.9999	B-H
Endothelial Cells vs. Macrophages	-35.75	No	ns	>0.9999	B-I
Endothelial Cells vs. Neurogenesis	92.34	No	ns	>0.9999	B-J
Endothelial Cells vs. Ependyma	-30.32	No	ns	>0.9999	B-K
Fibroblast-like vs. Mural Cells	85.93	No	ns	>0.9999	C-D

Fibroblast-like vs. Microglia	-5.505	No	ns	>0.9999	C-E
Fibroblast-like vs. Oligodendrocytes	100.5	No	ns	0.6204	C-F
Fibroblast-like vs. Polydendrocytes	158.2	Yes	**	0.0059	C-G
Fibroblast-like vs. Astrocytes	-25.36	No	ns	>0.9999	C-H
Fibroblast-like vs. Macrophages	-15.31	No	ns	>0.9999	C-I
Fibroblast-like vs. Neurogenesis	112.8	No	ns	>0.9999	C-J
Fibroblast-like vs. Ependyma	-9.88	No	ns	>0.9999	C-K
Mural Cells vs. Microglia	-91.44	No	ns	>0.9999	D-E
Mural Cells vs. Oligodendrocytes	14.61	No	ns	>0.9999	D-F
Mural Cells vs. Polydendrocytes	72.24	No	ns	>0.9999	D-G
Mural Cells vs. Astrocytes	-111.3	No	ns	0.434	D-H
Mural Cells vs. Macrophages	-101.2	No	ns	>0.9999	D-I
Mural Cells vs. Neurogenesis	26.86	No	ns	>0.9999	D-J
Mural Cells vs. Ependyma	-95.81	No	ns	>0.9999	D-K
Microglia vs. Oligodendrocytes	106	No	ns	>0.9999	E-F
Microglia vs. Polydendrocytes	163.7	No	ns	0.4285	E-G
Microglia vs. Astrocytes	-19.85	No	ns	>0.9999	E-H
Microglia vs. Macrophages	-9.804	No	ns	>0.9999	E-I
Microglia vs. Neurogenesis	118.3	No	ns	>0.9999	E-J
Microglia vs. Ependyma	-4.375	No	ns	>0.9999	E-K
Oligodendrocytes vs. Polydendrocytes	57.64	No	ns	>0.9999	F-G
Oligodendrocytes vs. Astrocytes	-125.9	No	ns	0.1104	F-H
Oligodendrocytes vs. Macrophages	-115.8	No	ns	>0.9999	F-I
Oligodendrocytes vs. Neurogenesis	12.25	No	ns	>0.9999	F-J
Oligodendrocytes vs. Ependyma	-110.4	No	ns	>0.9999	F-K
Polydendrocytes vs. Astrocytes	-183.5	Yes	***	0.0006	G-H
Polydendrocytes vs. Macrophages	-173.5	No	ns	0.4197	G-I
Polydendrocytes vs. Neurogenesis	-45.39	No	ns	>0.9999	G-J
Polydendrocytes vs. Ependyma	-168.1	No	ns	>0.9999	G-K
Astrocytes vs. Macrophages	10.05	No	ns	>0.9999	H-I
Astrocytes vs. Neurogenesis	138.1	No	ns	0.7662	H-J
Astrocytes vs. Ependyma	15.48	No	ns	>0.9999	H-K
Macrophages vs. Neurogenesis	128.1	No	ns	>0.9999	I-J
Macrophages vs. Ependyma	5.429	No	ns	>0.9999	I-K
Neurogenesis vs. Ependyma	-122.7	No	ns	>0.9999	J-K

Test details	Mean rank 1	Mean rank 2	Mean rank diff.	n1	n2	Z
Neurons vs. Endothelial Cells	159.6	471.7	-312.1	315	34	10.96
Neurons vs. Fibroblast-like	159.6	492.1	-332.5	315	25	10.14
Neurons vs. Mural Cells	159.6	406.2	-246.6	315	37	8.995
Neurons vs. Microglia	159.6	497.6	-338	315	8	5.985
Neurons vs. Oligodendrocytes	159.6	391.6	-232	315	43	9.045
Neurons vs. Polydendrocytes	159.6	333.9	-174.4	315	37	6.36
Neurons vs. Astrocytes	159.6	517.5	-357.9	315	23	10.5
Neurons vs. Macrophages	159.6	507.4	-347.8	315	7	5.77
Neurons vs. Neurogenesis	159.6	379.3	-219.7	315	12	4.736
Neurons vs. Ependyma	159.6	502	-342.4	315	5	4.815

Endothelial Cells vs. Fibroblast-like	471.7	492.1	-20.44	34	25	0.4919
Endothelial Cells vs. Mural Cells	471.7	406.2	65.49	34	37	1.747
Endothelial Cells vs. Microglia	471.7	497.6	-25.95	34	8	0.4186
Endothelial Cells vs. Oligodendrocytes	471.7	391.6	80.1	34	43	2.212
Endothelial Cells vs. Polydendrocytes	471.7	333.9	137.7	34	37	3.675
Endothelial Cells vs. Astrocytes	471.7	517.5	-45.8	34	23	1.075
Endothelial Cells vs. Macrophages	471.7	507.4	-35.75	34	7	0.546
Endothelial Cells vs. Neurogenesis	471.7	379.3	92.34	34	12	1.743
Endothelial Cells vs. Ependyma	471.7	502	-30.32	34	5	0.4013
Fibroblast-like vs. Mural Cells	492.1	406.2	85.93	25	37	2.104
Fibroblast-like vs. Microglia	492.1	497.6	-5.505	25	8	0.08591
Fibroblast-like vs. Oligodendrocytes	492.1	391.6	100.5	25	43	2.534
Fibroblast-like vs. Polydendrocytes	492.1	333.9	158.2	25	37	3.873
Fibroblast-like vs. Astrocytes	492.1	517.5	-25.36	25	23	0.5563
Fibroblast-like vs. Macrophages	492.1	507.4	-15.31	25	7	0.2269
Fibroblast-like vs. Neurogenesis	492.1	379.3	112.8	25	12	2.036
Fibroblast-like vs. Ependyma	492.1	502	-9.88	25	5	0.1278
Mural Cells vs. Microglia	406.2	497.6	-91.44	37	8	1.486
Mural Cells vs. Oligodendrocytes	406.2	391.6	14.61	37	43	0.4129
Mural Cells vs. Polydendrocytes	406.2	333.9	72.24	37	37	1.97
Mural Cells vs. Astrocytes	406.2	517.5	-111.3	37	23	2.657
Mural Cells vs. Macrophages	406.2	507.4	-101.2	37	7	1.557
Mural Cells vs. Neurogenesis	406.2	379.3	26.86	37	12	0.5124
Mural Cells vs. Ependyma	406.2	502	-95.81	37	5	1.275
Microglia vs. Oligodendrocytes	497.6	391.6	106	8	43	1.746
Microglia vs. Polydendrocytes	497.6	333.9	163.7	8	37	2.661
Microglia vs. Astrocytes	497.6	517.5	-19.85	8	23	0.3066
Microglia vs. Macrophages	497.6	507.4	-9.804	8	7	0.1201
Microglia vs. Neurogenesis	497.6	379.3	118.3	8	12	1.643
Microglia vs. Ependyma	497.6	502	-4.375	8	5	0.04865
Oligodendrocytes vs. Polydendrocytes	391.6	333.9	57.64	43	37	1.629
Oligodendrocytes vs. Astrocytes	391.6	517.5	-125.9	43	23	3.089
Oligodendrocytes vs. Macrophages	391.6	507.4	-115.8	43	7	1.802
Oligodendrocytes vs. Neurogenesis	391.6	379.3	12.25	43	12	0.2378
Oligodendrocytes vs. Ependyma	391.6	502	-110.4	43	5	1.481
Polydendrocytes vs. Astrocytes	333.9	517.5	-183.5	37	23	4.381
Polydendrocytes vs. Macrophages	333.9	507.4	-173.5	37	7	2.668
Polydendrocytes vs. Neurogenesis	333.9	379.3	-45.39	37	12	0.866
Polydendrocytes vs. Ependyma	333.9	502	-168.1	37	5	2.236
Astrocytes vs. Macrophages	517.5	507.4	10.05	23	7	0.1476
Astrocytes vs. Neurogenesis	517.5	379.3	138.1	23	12	2.459
Astrocytes vs. Ependyma	517.5	502	15.48	23	5	0.1988
Macrophages vs. Neurogenesis	507.4	379.3	128.1	7	12	1.707
Macrophages vs. Ependyma	507.4	502	5.429	7	5	0.05877
Neurogenesis vs. Ependyma	379.3	502	-122.7	12	5	1.461

Sod1-Nfe2l2-cell

Number of families	1
Number of comparisons per family	55
Alpha	0.05

Dunn's multiple comparisons test	Mean rank diff.	Significant?	Summary	Adjusted P Value	
Neurons vs. Endothelial Cells	-297.1	Yes	****	<0.0001	A-B
Neurons vs. Fibroblast-like	-251.1	Yes	****	<0.0001	A-C
Neurons vs. Mural Cells	-139.2	Yes	****	<0.0001	A-D
Neurons vs. Microglia	-276	Yes	****	<0.0001	A-E
Neurons vs. Oligodendrocytes	-34.22	No	ns	>0.9999	A-F
Neurons vs. Polydendrocytes	20.57	No	ns	>0.9999	A-G
Neurons vs. Astrocytes	-245.8	Yes	****	<0.0001	A-H
Neurons vs. Macrophages	-306.9	Yes	****	<0.0001	A-I
Neurons vs. Neurogenesis	-189.3	Yes	**	0.0025	A-J
Neurons vs. Ependyma	-331.2	Yes	***	0.0002	A-K
Endothelial Cells vs. Fibroblast-like	45.99	No	ns	>0.9999	B-C
Endothelial Cells vs. Mural Cells	157.9	Yes	**	0.0014	B-D
Endothelial Cells vs. Microglia	21.1	No	ns	>0.9999	B-E
Endothelial Cells vs. Oligodendrocytes	262.9	Yes	****	<0.0001	B-F
Endothelial Cells vs. Polydendrocytes	317.7	Yes	****	<0.0001	B-G
Endothelial Cells vs. Astrocytes	51.35	No	ns	>0.9999	B-H
Endothelial Cells vs. Macrophages	-9.79	No	ns	>0.9999	B-I
Endothelial Cells vs. Neurogenesis	107.9	No	ns	>0.9999	B-J
Endothelial Cells vs. Ependyma	-34.05	No	ns	>0.9999	B-K
Fibroblast-like vs. Mural Cells	111.9	No	ns	0.3375	C-D
Fibroblast-like vs. Microglia	-24.89	No	ns	>0.9999	C-E
Fibroblast-like vs. Oligodendrocytes	216.9	Yes	****	<0.0001	C-F
Fibroblast-like vs. Polydendrocytes	271.7	Yes	****	<0.0001	C-G
Fibroblast-like vs. Astrocytes	5.36	No	ns	>0.9999	C-H
Fibroblast-like vs. Macrophages	-55.78	No	ns	>0.9999	C-I
Fibroblast-like vs. Neurogenesis	61.86	No	ns	>0.9999	C-J
Fibroblast-like vs. Ependyma	-80.04	No	ns	>0.9999	C-K
Mural Cells vs. Microglia	-136.8	No	ns	>0.9999	D-E
Mural Cells vs. Oligodendrocytes	105	No	ns	0.1653	D-F
Mural Cells vs. Polydendrocytes	159.8	Yes	***	0.0007	D-G
Mural Cells vs. Astrocytes	-106.6	No	ns	0.6028	D-H
Mural Cells vs. Macrophages	-167.7	No	ns	0.5447	D-I
Mural Cells vs. Neurogenesis	-50.07	No	ns	>0.9999	D-J
Mural Cells vs. Ependyma	-192	No	ns	0.586	D-K
Microglia vs. Oligodendrocytes	241.8	Yes	**	0.0038	E-F
Microglia vs. Polydendrocytes	296.6	Yes	****	<0.0001	E-G
Microglia vs. Astrocytes	30.25	No	ns	>0.9999	E-H
Microglia vs. Macrophages	-30.89	No	ns	>0.9999	E-I
Microglia vs. Neurogenesis	86.75	No	ns	>0.9999	E-J
Microglia vs. Ependyma	-55.15	No	ns	>0.9999	E-K
Oligodendrocytes vs. Polydendrocytes	54.79	No	ns	>0.9999	F-G

Oligodendrocytes vs. Astrocytes	-211.5	Yes	****	<0.0001	F-H
Oligodendrocytes vs. Macrophages	-272.7	Yes	**	0.0012	F-I
Oligodendrocytes vs. Neurogenesis	-155	No	ns	0.1437	F-J
Oligodendrocytes vs. Ependyma	-296.9	Yes	**	0.0037	F-K
Polydendrocytes vs. Astrocytes	-266.3	Yes	****	<0.0001	G-H
Polydendrocytes vs. Macrophages	-327.5	Yes	****	<0.0001	G-I
Polydendrocytes vs. Neurogenesis	-209.8	Yes	**	0.0034	G-J
Polydendrocytes vs. Ependyma	-351.7	Yes	***	0.0002	G-K
Astrocytes vs. Macrophages	-61.14	No	ns	>0.9999	H-I
Astrocytes vs. Neurogenesis	56.5	No	ns	>0.9999	H-J
Astrocytes vs. Ependyma	-85.4	No	ns	>0.9999	H-K
Macrophages vs. Neurogenesis	117.6	No	ns	>0.9999	I-J
Macrophages vs. Ependyma	-24.26	No	ns	>0.9999	I-K
Neurogenesis vs. Ependyma	-141.9	No	ns	>0.9999	J-K

Test details	Mean rank 1	Mean rank 2	Mean rank diff.	n1	n2	Z
Neurons vs. Endothelial Cells	207.2	504.4	-297.1	315	34	10.43
Neurons vs. Fibroblast-like	207.2	458.4	-251.1	315	25	7.661
Neurons vs. Mural Cells	207.2	346.4	-139.2	315	37	5.077
Neurons vs. Microglia	207.2	483.3	-276	315	8	4.887
Neurons vs. Oligodendrocytes	207.2	241.5	-34.22	315	43	1.334
Neurons vs. Polydendrocytes	207.2	186.7	20.57	315	37	0.7502
Neurons vs. Astrocytes	207.2	453	-245.8	315	23	7.212
Neurons vs. Macrophages	207.2	514.1	-306.9	315	7	5.091
Neurons vs. Neurogenesis	207.2	396.5	-189.3	315	12	4.079
Neurons vs. Ependyma	207.2	538.4	-331.2	315	5	4.657
Endothelial Cells vs. Fibroblast-like	504.4	458.4	45.99	34	25	1.107
Endothelial Cells vs. Mural Cells	504.4	346.4	157.9	34	37	4.214
Endothelial Cells vs. Microglia	504.4	483.3	21.1	34	8	0.3404
Endothelial Cells vs. Oligodendrocytes	504.4	241.5	262.9	34	43	7.261
Endothelial Cells vs. Polydendrocytes	504.4	186.7	317.7	34	37	8.476
Endothelial Cells vs. Astrocytes	504.4	453	51.35	34	23	1.206
Endothelial Cells vs. Macrophages	504.4	514.1	-9.79	34	7	0.1495
Endothelial Cells vs. Neurogenesis	504.4	396.5	107.9	34	12	2.036
Endothelial Cells vs. Ependyma	504.4	538.4	-34.05	34	5	0.4506
Fibroblast-like vs. Mural Cells	458.4	346.4	111.9	25	37	2.74
Fibroblast-like vs. Microglia	458.4	483.3	-24.89	25	8	0.3884
Fibroblast-like vs. Oligodendrocytes	458.4	241.5	216.9	25	43	5.466
Fibroblast-like vs. Polydendrocytes	458.4	186.7	271.7	25	37	6.652
Fibroblast-like vs. Astrocytes	458.4	453	5.36	25	23	0.1176
Fibroblast-like vs. Macrophages	458.4	514.1	-55.78	25	7	0.8269
Fibroblast-like vs. Neurogenesis	458.4	396.5	61.86	25	12	1.117
Fibroblast-like vs. Ependyma	458.4	538.4	-80.04	25	5	1.036
Mural Cells vs. Microglia	346.4	483.3	-136.8	37	8	2.224
Mural Cells vs. Oligodendrocytes	346.4	241.5	105	37	43	2.967
Mural Cells vs. Polydendrocytes	346.4	186.7	159.8	37	37	4.356
Mural Cells vs. Astrocytes	346.4	453	-106.6	37	23	2.544

Mural Cells vs. Macrophages	346.4	514.1	-167.7	37	7	2.579
Mural Cells vs. Neurogenesis	346.4	396.5	-50.07	37	12	0.9553
Mural Cells vs. Ependyma	346.4	538.4	-192	37	5	2.554
Microglia vs. Oligodendrocytes	483.3	241.5	241.8	8	43	3.98
Microglia vs. Polydendrocytes	483.3	186.7	296.6	8	37	4.821
Microglia vs. Astrocytes	483.3	453	30.25	8	23	0.4671
Microglia vs. Macrophages	483.3	514.1	-30.89	8	7	0.3784
Microglia vs. Neurogenesis	483.3	396.5	86.75	8	12	1.205
Microglia vs. Ependyma	483.3	538.4	-55.15	8	5	0.6132
Oligodendrocytes vs. Polydendrocytes	241.5	186.7	54.79	43	37	1.549
Oligodendrocytes vs. Astrocytes	241.5	453	-211.5	43	23	5.19
Oligodendrocytes vs. Macrophages	241.5	514.1	-272.7	43	7	4.241
Oligodendrocytes vs. Neurogenesis	241.5	396.5	-155	43	12	3.01
Oligodendrocytes vs. Ependyma	241.5	538.4	-296.9	43	5	3.983
Polydendrocytes vs. Astrocytes	186.7	453	-266.3	37	23	6.358
Polydendrocytes vs. Macrophages	186.7	514.1	-327.5	37	7	5.036
Polydendrocytes vs. Neurogenesis	186.7	396.5	-209.8	37	12	4.004
Polydendrocytes vs. Ependyma	186.7	538.4	-351.7	37	5	4.679
Astrocytes vs. Macrophages	453	514.1	-61.14	23	7	0.8978
Astrocytes vs. Neurogenesis	453	396.5	56.5	23	12	1.006
Astrocytes vs. Ependyma	453	538.4	-85.4	23	5	1.097
Macrophages vs. Neurogenesis	514.1	396.5	117.6	7	12	1.568
Macrophages vs. Ependyma	514.1	538.4	-24.26	7	5	0.2626
Neurogenesis vs. Ependyma	396.5	538.4	-141.9	12	5	1.69

Keap1-interact-DA-raw

Number of families	1
Number of comparisons per family	120
Alpha	0.05

Tukey's multiple comparisons test	Mean Diff.	95.00% CI of diff.	Significant?	Summary	Adjusted P Value	
Amer1 vs. *Dpp3*	-1.391	-7.296 to 4.513	No	ns	>0.9999	A-B
Amer1 vs. *Fam117b*	-3.836	-9.740 to 2.069	No	ns	0.6457	A-C
Amer1 vs. *Fam129b*	0.6745	-5.230 to 6.579	No	ns	>0.9999	A-D
Amer1 vs. *Ikbkb*	-0.8466	-6.751 to 5.058	No	ns	>0.9999	A-E
Amer1 vs. *Mad2l1*	0.9083	-4.996 to 6.813	No	ns	>0.9999	A-F
Amer1 vs. *Mcm3*	1.392	-4.513 to 7.297	No	ns	>0.9999	A-G
Amer1 vs. *Nfe2l1*	-46.63	-52.54 to -40.73	Yes	****	<0.0001	A-H
Amer1 vs. *Nfe2l2*	1.324	-4.581 to 7.228	No	ns	>0.9999	A-I
Amer1 vs. *Palb2*	1.38	-4.524 to 7.285	No	ns	>0.9999	A-J
Amer1 vs. *Pgam5*	-1.772	-7.677 to 4.133	No	ns	0.9996	A-K
Amer1 vs. *Ptma*	-3.121	-9.026 to 2.783	No	ns	0.8937	A-L
Amer1 vs. *Slk*	-6.252	-12.16 to -0.3471	Yes	*	0.0267	A-M
Amer1 vs. *Sqstm1*	-21.85	-27.75 to -15.94	Yes	****	<0.0001	A-N
Amer1 vs. *Tsc22d4*	-4.171	-10.08 to 1.733	No	ns	0.5005	A-O
Amer1 vs. *Wdr1*	-10.93	-16.83 to -5.023	Yes	****	<0.0001	A-P

Dpp3 vs. *Fam117b*	-2.444	-8.349 to 3.460	No	ns		0.9862	B-C
Dpp3 vs. *Fam129b*	2.066	-3.839 to 7.970	No	ns		0.9975	B-D
Dpp3 vs. *Ikbkb*	0.5448	-5.360 to 6.449	No	ns	>0.9999		B-E
Dpp3 vs. *Mad2l1*	2.3	-3.605 to 8.204	No	ns		0.9924	B-F
Dpp3 vs. *Mcm3*	2.783	-3.121 to 8.688	No	ns		0.9559	B-G
Dpp3 vs. *Nfe2l1*	-45.24	-51.14 to -39.34	Yes	****	<0.0001		B-H
Dpp3 vs. *Nfe2l2*	2.715	-3.190 to 8.620	No	ns		0.9643	B-I
Dpp3 vs. *Palb2*	2.772	-3.133 to 8.676	No	ns		0.9574	B-J
Dpp3 vs. *Pgam5*	-0.3806	-6.285 to 5.524	No	ns	>0.9999		B-K
Dpp3 vs. *Ptma*	-1.73	-7.634 to 4.175	No	ns		0.9997	B-L
Dpp3 vs. *Slk*	-4.86	-10.76 to 1.044	No	ns		0.2413	B-M
Dpp3 vs. *Sqstm1*	-20.46	-26.36 to -14.55	Yes	****	<0.0001		B-N
Dpp3 vs. *Tsc22d4*	-2.78	-8.685 to 3.125	No	ns		0.9564	B-O
Dpp3 vs. *Wdr1*	-9.536	-15.44 to -3.631	Yes	****	<0.0001		B-P
Fam117b vs. *Fam129b*	4.51	-1.395 to 10.41	No	ns		0.3617	C-D
Fam117b vs. *Ikbkb*	2.989	-2.916 to 8.894	No	ns		0.9225	C-E
Fam117b vs. *Mad2l1*	4.744	-1.161 to 10.65	No	ns		0.2781	C-F
Fam117b vs. *Mcm3*	5.227	-0.6771 to 11.13	No	ns		0.1472	C-G
Fam117b vs. *Nfe2l1*	-42.8	-48.70 to -36.89	Yes	****	<0.0001		C-H
Fam117b vs. *Nfe2l2*	5.159	-0.7454 to 11.06	No	ns		0.1622	C-I
Fam117b vs. *Palb2*	5.216	-0.6887 to 11.12	No	ns		0.1497	C-J
Fam117b vs. *Pgam5*	2.064	-3.841 to 7.968	No	ns		0.9976	C-K
Fam117b vs. *Ptma*	0.7143	-5.190 to 6.619	No	ns	>0.9999		C-L
Fam117b vs. *Slk*	-2.416	-8.321 to 3.488	No	ns		0.9877	C-M
Fam117b vs. *Sqstm1*	-18.01	-23.92 to -12.11	Yes	****	<0.0001		C-N
Fam117b vs. *Tsc22d4*	-0.3358	-6.240 to 5.569	No	ns	>0.9999		C-O
Fam117b vs. *Wdr1*	-7.092	-13.00 to -1.187	Yes	**		0.0049	C-P
Fam129b vs. *Ikbkb*	-1.521	-7.426 to 4.384	No	ns	>0.9999		D-E
Fam129b vs. *Mad2l1*	0.2339	-5.671 to 6.138	No	ns	>0.9999		D-F
Fam129b vs. *Mcm3*	0.7175	-5.187 to 6.622	No	ns	>0.9999		D-G
Fam129b vs. *Nfe2l1*	-47.31	-53.21 to -41.40	Yes	****	<0.0001		D-H
Fam129b vs. *Nfe2l2*	0.6492	-5.255 to 6.554	No	ns	>0.9999		D-I
Fam129b vs. *Palb2*	0.7059	-5.199 to 6.611	No	ns	>0.9999		D-J
Fam129b vs. *Pgam5*	-2.446	-8.351 to 3.458	No	ns		0.9861	D-K
Fam129b vs. *Ptma*	-3.796	-9.700 to 2.109	No	ns		0.6625	D-L
Fam129b vs. *Slk*	-6.926	-12.83 to -1.022	Yes	**		0.0069	D-M
Fam129b vs. *Sqstm1*	-22.52	-28.43 to -16.62	Yes	****	<0.0001		D-N
Fam129b vs. *Tsc22d4*	-4.846	-10.75 to 1.059	No	ns		0.2457	D-O
Fam129b vs. *Wdr1*	-11.6	-17.51 to -5.697	Yes	****	<0.0001		D-P
Ikbkb vs. *Mad2l1*	1.755	-4.150 to 7.659	No	ns		0.9996	E-F
Ikbkb vs. *Mcm3*	2.239	-3.666 to 8.143	No	ns		0.9942	E-G
Ikbkb vs. *Nfe2l1*	-45.78	-51.69 to -39.88	Yes	****	<0.0001		E-H
Ikbkb vs. *Nfe2l2*	2.17	-3.734 to 8.075	No	ns		0.9958	E-I
Ikbkb vs. *Palb2*	2.227	-3.678 to 8.132	No	ns		0.9945	E-J
Ikbkb vs. *Pgam5*	-0.9254	-6.830 to 4.979	No	ns	>0.9999		E-K
Ikbkb vs. *Ptma*	-2.275	-8.179 to 3.630	No	ns		0.9932	E-L
Ikbkb vs. *Slk*	-5.405	-11.31 to 0.4995	No	ns		0.1131	E-M

Ikbkb vs. *Sqstm1*	-21	-26.91 to -15.10	Yes	****	<0.0001	E-N
Ikbkb vs. *Tsc22d4*	-3.325	-9.229 to 2.580	No	ns	0.8377	E-O
Ikbkb vs. *Wdr1*	-10.08	-15.99 to -4.176	Yes	****	<0.0001	E-P
Mad2l1 vs. *Mcm3*	0.4836	-5.421 to 6.388	No	ns	>0.9999	F-G
Mad2l1 vs. *Nfe2l1*	-47.54	-53.44 to -41.64	Yes	****	<0.0001	F-H
Mad2l1 vs. *Nfe2l2*	0.4154	-5.489 to 6.320	No	ns	>0.9999	F-I
Mad2l1 vs. *Palb2*	0.4721	-5.433 to 6.377	No	ns	>0.9999	F-J
Mad2l1 vs. *Pgam5*	-2.68	-8.585 to 3.224	No	ns	0.968	F-K
Mad2l1 vs. *Ptma*	-4.03	-9.934 to 1.875	No	ns	0.5619	F-L
Mad2l1 vs. *Slk*	-7.16	-13.06 to -1.255	Yes	**	0.0042	F-M
Mad2l1 vs. *Sqstm1*	-22.76	-28.66 to -16.85	Yes	****	<0.0001	F-N
Mad2l1 vs. *Tsc22d4*	-5.08	-10.98 to 0.8249	No	ns	0.1811	F-O
Mad2l1 vs. *Wdr1*	-11.84	-17.74 to -5.931	Yes	****	<0.0001	F-P
Mcm3 vs. *Nfe2l1*	-48.02	-53.93 to -42.12	Yes	****	<0.0001	G-H
Mcm3 vs. *Nfe2l2*	-0.06825	-5.973 to 5.836	No	ns	>0.9999	G-I
Mcm3 vs. *Palb2*	-0.01155	-5.916 to 5.893	No	ns	>0.9999	G-J
Mcm3 vs. *Pgam5*	-3.164	-9.068 to 2.741	No	ns	0.8831	G-K
Mcm3 vs. *Ptma*	-4.513	-10.42 to 1.391	No	ns	0.3605	G-L
Mcm3 vs. *Slk*	-7.644	-13.55 to -1.739	Yes	**	0.0014	G-M
Mcm3 vs. *Sqstm1*	-23.24	-29.15 to -17.34	Yes	****	<0.0001	G-N
Mcm3 vs. *Tsc22d4*	-5.563	-11.47 to 0.3413	No	ns	0.0884	G-O
Mcm3 vs. *Wdr1*	-12.32	-18.22 to -6.415	Yes	****	<0.0001	G-P
Nfe2l1 vs. *Nfe2l2*	47.96	42.05 to 53.86	Yes	****	<0.0001	H-I
Nfe2l1 vs. *Palb2*	48.01	42.11 to 53.92	Yes	****	<0.0001	H-J
Nfe2l1 vs. *Pgam5*	44.86	38.95 to 50.76	Yes	****	<0.0001	H-K
Nfe2l1 vs. *Ptma*	43.51	37.61 to 49.41	Yes	****	<0.0001	H-L
Nfe2l1 vs. *Slk*	40.38	34.48 to 46.28	Yes	****	<0.0001	H-M
Nfe2l1 vs. *Sqstm1*	24.78	18.88 to 30.69	Yes	****	<0.0001	H-N
Nfe2l1 vs. *Tsc22d4*	42.46	36.56 to 48.36	Yes	****	<0.0001	H-O
Nfe2l1 vs. *Wdr1*	35.7	29.80 to 41.61	Yes	****	<0.0001	H-P
Nfe2l2 vs. *Palb2*	0.05669	-5.848 to 5.961	No	ns	>0.9999	I-J
Nfe2l2 vs. *Pgam5*	-3.096	-9.000 to 2.809	No	ns	0.8997	I-K
Nfe2l2 vs. *Ptma*	-4.445	-10.35 to 1.460	No	ns	0.387	I-L
Nfe2l2 vs. *Slk*	-7.575	-13.48 to -1.671	Yes	**	0.0017	I-M
Nfe2l2 vs. *Sqstm1*	-23.17	-29.08 to -17.27	Yes	****	<0.0001	I-N
Nfe2l2 vs. *Tsc22d4*	-5.495	-11.40 to 0.4095	No	ns	0.0985	I-O
Nfe2l2 vs. *Wdr1*	-12.25	-18.16 to -6.347	Yes	****	<0.0001	I-P
Palb2 vs. *Pgam5*	-3.152	-9.057 to 2.752	No	ns	0.886	J-K
Palb2 vs. *Ptma*	-4.502	-10.41 to 1.403	No	ns	0.3649	J-L
Palb2 vs. *Slk*	-7.632	-13.54 to -1.728	Yes	**	0.0015	J-M
Palb2 vs. *Sqstm1*	-23.23	-29.13 to -17.32	Yes	****	<0.0001	J-N
Palb2 vs. *Tsc22d4*	-5.552	-11.46 to 0.3528	No	ns	0.09	J-O
Palb2 vs. *Wdr1*	-12.31	-18.21 to -6.403	Yes	****	<0.0001	J-P
Pgam5 vs. *Ptma*	-1.349	-7.254 to 4.555	No	ns	>0.9999	K-L
Pgam5 vs. *Slk*	-4.48	-10.38 to 1.425	No	ns	0.3733	K-M
Pgam5 vs. *Sqstm1*	-20.08	-25.98 to -14.17	Yes	****	<0.0001	K-N
Pgam5 vs. *Tsc22d4*	-2.399	-8.304 to 3.505	No	ns	0.9885	K-O

Pgam5 vs. *Wdr1*	-9.155	-15.06 to -3.251	Yes	****	<0.0001	K-P
Ptma vs. *Slk*	-3.13	-9.035 to 2.774	No	ns	0.8914	L-M
Ptma vs. *Sqstm1*	-18.73	-24.63 to -12.82	Yes	****	<0.0001	L-N
Ptma vs. *Tsc22d4*	-1.05	-6.955 to 4.854	No	ns	>0.9999	L-O
Ptma vs. *Wdr1*	-7.806	-13.71 to -1.902	Yes	***	0.001	L-P
Slk vs. *Sqstm1*	-15.6	-21.50 to -9.692	Yes	****	<0.0001	M-N
Slk vs. *Tsc22d4*	2.08	-3.824 to 7.985	No	ns	0.9973	M-O
Slk vs. *Wdr1*	-4.676	-10.58 to 1.229	No	ns	0.3012	M-P
Sqstm1 vs. *Tsc22d4*	17.68	11.77 to 23.58	Yes	****	<0.0001	N-O
Sqstm1 vs. *Wdr1*	10.92	5.016 to 16.83	Yes	****	<0.0001	N-P
Tsc22d4 vs. *Wdr1*	-6.756	-12.66 to -0.8515	Yes	**	0.0099	O-P

Test details	Mean 1	Mean 2	Mean Diff.	SE of diff.	n1	n2	q	DF
Amer1 vs. *Dpp3*	1.443	2.834	-1.391	1.689	9	9	1.165	128
Amer1 vs. *Fam117b*	1.443	5.278	-3.836	1.689	9	9	3.211	128
Amer1 vs. *Fam129b*	1.443	0.7681	0.6745	1.689	9	9	0.5647	128
Amer1 vs. *Ikbkb*	1.443	2.289	-0.8466	1.689	9	9	0.7087	128
Amer1 vs. *Mad2l1*	1.443	0.5342	0.9083	1.689	9	9	0.7604	128
Amer1 vs. *Mcm3*	1.443	0.0506	1.392	1.689	9	9	1.165	128
Amer1 vs. *Nfe2l1*	1.443	48.07	-46.63	1.689	9	9	39.04	128
Amer1 vs. *Nfe2l2*	1.443	0.1188	1.324	1.689	9	9	1.108	128
Amer1 vs. *Palb2*	1.443	0.06215	1.38	1.689	9	9	1.156	128
Amer1 vs. *Pgam5*	1.443	3.214	-1.772	1.689	9	9	1.483	128
Amer1 vs. *Ptma*	1.443	4.564	-3.121	1.689	9	9	2.613	128
Amer1 vs. *Slk*	1.443	7.694	-6.252	1.689	9	9	5.234	128
Amer1 vs. *Sqstm1*	1.443	23.29	-21.85	1.689	9	9	18.29	128
Amer1 vs. *Tsc22d4*	1.443	5.614	-4.171	1.689	9	9	3.492	128
Amer1 vs. *Wdr1*	1.443	12.37	-10.93	1.689	9	9	9.148	128
Dpp3 vs. *Fam117b*	2.834	5.278	-2.444	1.689	9	9	2.046	128
Dpp3 vs. *Fam129b*	2.834	0.7681	2.066	1.689	9	9	1.729	128
Dpp3 vs. *Ikbkb*	2.834	2.289	0.5448	1.689	9	9	0.4561	128
Dpp3 vs. *Mad2l1*	2.834	0.5342	2.3	1.689	9	9	1.925	128
Dpp3 vs. *Mcm3*	2.834	0.0506	2.783	1.689	9	9	2.33	128
Dpp3 vs. *Nfe2l1*	2.834	48.07	-45.24	1.689	9	9	37.87	128
Dpp3 vs. *Nfe2l2*	2.834	0.1188	2.715	1.689	9	9	2.273	128
Dpp3 vs. *Palb2*	2.834	0.06215	2.772	1.689	9	9	2.32	128
Dpp3 vs. *Pgam5*	2.834	3.214	-0.3806	1.689	9	9	0.3186	128
Dpp3 vs. *Ptma*	2.834	4.564	-1.73	1.689	9	9	1.448	128
Dpp3 vs. *Slk*	2.834	7.694	-4.86	1.689	9	9	4.069	128
Dpp3 vs. *Sqstm1*	2.834	23.29	-20.46	1.689	9	9	17.13	128
Dpp3 vs. *Tsc22d4*	2.834	5.614	-2.78	1.689	9	9	2.327	128
Dpp3 vs. *Wdr1*	2.834	12.37	-9.536	1.689	9	9	7.983	128
Fam117b vs. *Fam129b*	5.278	0.7681	4.51	1.689	9	9	3.776	128
Fam117b vs. *Ikbkb*	5.278	2.289	2.989	1.689	9	9	2.502	128
Fam117b vs. *Mad2l1*	5.278	0.5342	4.744	1.689	9	9	3.971	128
Fam117b vs. *Mcm3*	5.278	0.0506	5.227	1.689	9	9	4.376	128
Fam117b vs. *Nfe2l1*	5.278	48.07	-42.8	1.689	9	9	35.83	128

Fam117b vs. *Nfe2l2*	5.278	0.1188	5.159	1.689	9	9	4.319	128
Fam117b vs. *Palb2*	5.278	0.06215	5.216	1.689	9	9	4.367	128
Fam117b vs. *Pgam5*	5.278	3.214	2.064	1.689	9	9	1.728	128
Fam117b vs. *Ptma*	5.278	4.564	0.7143	1.689	9	9	0.598	128
Fam117b vs. *Slk*	5.278	7.694	-2.416	1.689	9	9	2.023	128
Fam117b vs. *Sqstm1*	5.278	23.29	-18.01	1.689	9	9	15.08	128
Fam117b vs. *Tsc22d4*	5.278	5.614	-0.3358	1.689	9	9	0.2812	128
Fam117b vs. *Wdr1*	5.278	12.37	-7.092	1.689	9	9	5.937	128
Fam129b vs. *Ikbkb*	0.7681	2.289	-1.521	1.689	9	9	1.273	128
Fam129b vs. *Mad2l1*	0.7681	0.5342	0.2339	1.689	9	9	0.1958	128
Fam129b vs. *Mcm3*	0.7681	0.0506	0.7175	1.689	9	9	0.6007	128
Fam129b vs. *Nfe2l1*	0.7681	48.07	-47.31	1.689	9	9	39.6	128
Fam129b vs. *Nfe2l2*	0.7681	0.1188	0.6492	1.689	9	9	0.5435	128
Fam129b vs. *Palb2*	0.7681	0.06215	0.7059	1.689	9	9	0.591	128
Fam129b vs. *Pgam5*	0.7681	3.214	-2.446	1.689	9	9	2.048	128
Fam129b vs. *Ptma*	0.7681	4.564	-3.796	1.689	9	9	3.178	128
Fam129b vs. *Slk*	0.7681	7.694	-6.926	1.689	9	9	5.799	128
Fam129b vs. *Sqstm1*	0.7681	23.29	-22.52	1.689	9	9	18.86	128
Fam129b vs. *Tsc22d4*	0.7681	5.614	-4.846	1.689	9	9	4.057	128
Fam129b vs. *Wdr1*	0.7681	12.37	-11.6	1.689	9	9	9.713	128
Ikbkb vs. *Mad2l1*	2.289	0.5342	1.755	1.689	9	9	1.469	128
Ikbkb vs. *Mcm3*	2.289	0.0506	2.239	1.689	9	9	1.874	128
Ikbkb vs. *Nfe2l1*	2.289	48.07	-45.78	1.689	9	9	38.33	128
Ikbkb vs. *Nfe2l2*	2.289	0.1188	2.17	1.689	9	9	1.817	128
Ikbkb vs. *Palb2*	2.289	0.06215	2.227	1.689	9	9	1.864	128
Ikbkb vs. *Pgam5*	2.289	3.214	-0.9254	1.689	9	9	0.7747	128
Ikbkb vs. *Ptma*	2.289	4.564	-2.275	1.689	9	9	1.904	128
Ikbkb vs. *Slk*	2.289	7.694	-5.405	1.689	9	9	4.525	128
Ikbkb vs. *Sqstm1*	2.289	23.29	-21	1.689	9	9	17.58	128
Ikbkb vs. *Tsc22d4*	2.289	5.614	-3.325	1.689	9	9	2.783	128
Ikbkb vs. *Wdr1*	2.289	12.37	-10.08	1.689	9	9	8.44	128
Mad2l1 vs. *Mcm3*	0.5342	0.0506	0.4836	1.689	9	9	0.4049	128
Mad2l1 vs. *Nfe2l1*	0.5342	48.07	-47.54	1.689	9	9	39.8	128
Mad2l1 vs. *Nfe2l2*	0.5342	0.1188	0.4154	1.689	9	9	0.3478	128
Mad2l1 vs. *Palb2*	0.5342	0.06215	0.4721	1.689	9	9	0.3952	128
Mad2l1 vs. *Pgam5*	0.5342	3.214	-2.68	1.689	9	9	2.244	128
Mad2l1 vs. *Ptma*	0.5342	4.564	-4.03	1.689	9	9	3.373	128
Mad2l1 vs. *Slk*	0.5342	7.694	-7.16	1.689	9	9	5.994	128
Mad2l1 vs. *Sqstm1*	0.5342	23.29	-22.76	1.689	9	9	19.05	128
Mad2l1 vs. *Tsc22d4*	0.5342	5.614	-5.08	1.689	9	9	4.253	128
Mad2l1 vs. *Wdr1*	0.5342	12.37	-11.84	1.689	9	9	9.909	128
Mcm3 vs. *Nfe2l1*	0.0506	48.07	-48.02	1.689	9	9	40.2	128
Mcm3 vs. *Nfe2l2*	0.0506	0.1188	-0.06825	1.689	9	9	0.05713	128
Mcm3 vs. *Palb2*	0.0506	0.06215	-0.01155	1.689	9	9	0.009671	128
Mcm3 vs. *Pgam5*	0.0506	3.214	-3.164	1.689	9	9	2.649	128
Mcm3 vs. *Ptma*	0.0506	4.564	-4.513	1.689	9	9	3.778	128
Mcm3 vs. *Slk*	0.0506	7.694	-7.644	1.689	9	9	6.399	128

Mcm3 vs. *Sqstm1*	0.0506	23.29	-23.24	1.689	9	9	19.46	128
Mcm3 vs. *Tsc22d4*	0.0506	5.614	-5.563	1.689	9	9	4.658	128
Mcm3 vs. *Wdr1*	0.0506	12.37	-12.32	1.689	9	9	10.31	128
Nfe2l1 vs. *Nfe2l2*	48.07	0.1188	47.96	1.689	9	9	40.15	128
Nfe2l1 vs. *Palb2*	48.07	0.06215	48.01	1.689	9	9	40.2	128
Nfe2l1 vs. *Pgam5*	48.07	3.214	44.86	1.689	9	9	37.56	128
Nfe2l1 vs. *Ptma*	48.07	4.564	43.51	1.689	9	9	36.43	128
Nfe2l1 vs. *Slk*	48.07	7.694	40.38	1.689	9	9	33.81	128
Nfe2l1 vs. *Sqstm1*	48.07	23.29	24.78	1.689	9	9	20.75	128
Nfe2l1 vs. *Tsc22d4*	48.07	5.614	42.46	1.689	9	9	35.55	128
Nfe2l1 vs. *Wdr1*	48.07	12.37	35.7	1.689	9	9	29.89	128
Nfe2l2 vs. *Palb2*	0.1188	0.06215	0.05669	1.689	9	9	0.04746	128
Nfe2l2 vs. *Pgam5*	0.1188	3.214	-3.096	1.689	9	9	2.592	128
Nfe2l2 vs. *Ptma*	0.1188	4.564	-4.445	1.689	9	9	3.721	128
Nfe2l2 vs. *Slk*	0.1188	7.694	-7.575	1.689	9	9	6.342	128
Nfe2l2 vs. *Sqstm1*	0.1188	23.29	-23.17	1.689	9	9	19.4	128
Nfe2l2 vs. *Tsc22d4*	0.1188	5.614	-5.495	1.689	9	9	4.6	128
Nfe2l2 vs. *Wdr1*	0.1188	12.37	-12.25	1.689	9	9	10.26	128
Palb2 vs. *Pgam5*	0.06215	3.214	-3.152	1.689	9	9	2.639	128
Palb2 vs. *Ptma*	0.06215	4.564	-4.502	1.689	9	9	3.769	128
Palb2 vs. *Slk*	0.06215	7.694	-7.632	1.689	9	9	6.39	128
Palb2 vs. *Sqstm1*	0.06215	23.29	-23.23	1.689	9	9	19.45	128
Palb2 vs. *Tsc22d4*	0.06215	5.614	-5.552	1.689	9	9	4.648	128
Palb2 vs. *Wdr1*	0.06215	12.37	-12.31	1.689	9	9	10.3	128
Pgam5 vs. *Ptma*	3.214	4.564	-1.349	1.689	9	9	1.13	128
Pgam5 vs. *Slk*	3.214	7.694	-4.48	1.689	9	9	3.75	128
Pgam5 vs. *Sqstm1*	3.214	23.29	-20.08	1.689	9	9	16.81	128
Pgam5 vs. *Tsc22d4*	3.214	5.614	-2.399	1.689	9	9	2.009	128
Pgam5 vs. *Wdr1*	3.214	12.37	-9.155	1.689	9	9	7.665	128
Ptma vs. *Slk*	4.564	7.694	-3.13	1.689	9	9	2.621	128
Ptma vs. *Sqstm1*	4.564	23.29	-18.73	1.689	9	9	15.68	128
Ptma vs. *Tsc22d4*	4.564	5.614	-1.05	1.689	9	9	0.8791	128
Ptma vs. *Wdr1*	4.564	12.37	-7.806	1.689	9	9	6.535	128
Slk vs. *Sqstm1*	7.694	23.29	-15.6	1.689	9	9	13.06	128
Slk vs. *Tsc22d4*	7.694	5.614	2.08	1.689	9	9	1.742	128
Slk vs. *Wdr1*	7.694	12.37	-4.676	1.689	9	9	3.914	128
Sqstm1 vs. *Tsc22d4*	23.29	5.614	17.68	1.689	9	9	14.8	128
Sqstm1 vs. *Wdr1*	23.29	12.37	10.92	1.689	9	9	9.143	128
Tsc22d4 vs. *Wdr1*	5.614	12.37	-6.756	1.689	9	9	5.656	128

Keap1-interact-DA-rel

Number of families	1
Number of comparisons per family	120
Alpha	0.05

Tukey's multiple comparisons test	Mean Diff.	95.00% CI of diff.	Significant?	Summary	Adjusted P Value

Amer1 vs. *Dpp3*	-0.2191	-0.3352 to -0.1030	Yes	****	<0.0001		A-B
Amer1 vs. *Fam117b*	-0.1656	-0.2817 to -0.04946	Yes	***		0.0002	A-C
Amer1 vs. *Fam129b*	0.0627	-0.05343 to 0.1788	No	ns		0.8769	A-D
Amer1 vs. *Ikbkb*	-0.009063	-0.1252 to 0.1071	No	ns	>0.9999		A-E
Amer1 vs. *Mad2l1*	0.07842	-0.03770 to 0.1946	No	ns		0.5802	A-F
Amer1 vs. *Mcm3*	0.09758	-0.01855 to 0.2137	No	ns		0.212	A-G
Amer1 vs. *Nfe2l1*	-0.3987	-0.5148 to -0.2826	Yes	****	<0.0001		A-H
Amer1 vs. *Nfe2l2*	0.09659	-0.01953 to 0.2127	No	ns		0.2262	A-I
Amer1 vs. *Palb2*	0.07985	-0.03628 to 0.1960	No	ns		0.5486	A-J
Amer1 vs. *Pgam5*	-0.2425	-0.3586 to -0.1264	Yes	****	<0.0001		A-K
Amer1 vs. *Ptma*	0.06835	-0.04777 to 0.1845	No	ns		0.7877	A-L
Amer1 vs. *Slk*	0.0105	-0.1056 to 0.1266	No	ns	>0.9999		A-M
Amer1 vs. *Sqstm1*	-0.222	-0.3381 to -0.1058	Yes	****	<0.0001		A-N
Amer1 vs. *Tsc22d4*	0.08374	-0.03239 to 0.1999	No	ns		0.4636	A-O
Amer1 vs. *Wdr1*	-0.122	-0.2381 to -0.005828	Yes	*		0.0293	A-P
Dpp3 vs. *Fam117b*	0.0535	-0.06263 to 0.1696	No	ns		0.9637	B-C
Dpp3 vs. *Fam129b*	0.2818	0.1657 to 0.3979	Yes	****	<0.0001		B-D
Dpp3 vs. *Ikbkb*	0.21	0.09389 to 0.3261	Yes	****	<0.0001		B-E
Dpp3 vs. *Mad2l1*	0.2975	0.1814 to 0.4136	Yes	****	<0.0001		B-F
Dpp3 vs. *Mcm3*	0.3167	0.2005 to 0.4328	Yes	****	<0.0001		B-G
Dpp3 vs. *Nfe2l1*	-0.1796	-0.2957 to -0.06347	Yes	****	<0.0001		B-H
Dpp3 vs. *Nfe2l2*	0.3157	0.1996 to 0.4318	Yes	****	<0.0001		B-I
Dpp3 vs. *Palb2*	0.2989	0.1828 to 0.4151	Yes	****	<0.0001		B-J
Dpp3 vs. *Pgam5*	-0.02342	-0.1395 to 0.09271	No	ns	>0.9999		B-K
Dpp3 vs. *Ptma*	0.2874	0.1713 to 0.4036	Yes	****	<0.0001		B-L
Dpp3 vs. *Slk*	0.2296	0.1135 to 0.3457	Yes	****	<0.0001		B-M
Dpp3 vs. *Sqstm1*	-0.002871	-0.1190 to 0.1133	No	ns	>0.9999		B-N
Dpp3 vs. *Tsc22d4*	0.3028	0.1867 to 0.4190	Yes	****	<0.0001		B-O
Dpp3 vs. *Wdr1*	0.09713	-0.01900 to 0.2133	No	ns		0.2184	B-P
Fam117b vs. *Fam129b*	0.2283	0.1122 to 0.3444	Yes	****	<0.0001		C-D
Fam117b vs. *Ikbkb*	0.1565	0.04039 to 0.2726	Yes	***		0.0007	C-E
Fam117b vs. *Mad2l1*	0.244	0.1279 to 0.3601	Yes	****	<0.0001		C-F
Fam117b vs. *Mcm3*	0.2632	0.1470 to 0.3793	Yes	****	<0.0001		C-G
Fam117b vs. *Nfe2l1*	-0.2331	-0.3492 to -0.1170	Yes	****	<0.0001		C-H
Fam117b vs. *Nfe2l2*	0.2622	0.1461 to 0.3783	Yes	****	<0.0001		C-I
Fam117b vs. *Palb2*	0.2454	0.1293 to 0.3616	Yes	****	<0.0001		C-J
Fam117b vs. *Pgam5*	-0.07692	-0.1930 to 0.03921	No	ns		0.6133	C-K
Fam117b vs. *Ptma*	0.2339	0.1178 to 0.3501	Yes	****	<0.0001		C-L
Fam117b vs. *Slk*	0.1761	0.05996 to 0.2922	Yes	****	<0.0001		C-M
Fam117b vs. *Sqstm1*	-0.05637	-0.1725 to 0.05976	No	ns		0.944	C-N
Fam117b vs. *Tsc22d4*	0.2493	0.1332 to 0.3655	Yes	****	<0.0001		C-O
Fam117b vs. *Wdr1*	0.04363	-0.07250 to 0.1598	No	ns		0.9948	C-P
Fam129b vs. *Ikbkb*	-0.07176	-0.1879 to 0.04437	No	ns		0.7226	D-E
Fam129b vs. *Mad2l1*	0.01573	-0.1004 to 0.1319	No	ns	>0.9999		D-F
Fam129b vs. *Mcm3*	0.03488	-0.08124 to 0.1510	No	ns		0.9996	D-G
Fam129b vs. *Nfe2l1*	-0.4614	-0.5775 to -0.3452	Yes	****	<0.0001		D-H
Fam129b vs. *Nfe2l2*	0.0339	-0.08223 to 0.1500	No	ns		0.9997	D-I

Fam129b vs. *Palb2*	0.01716	-0.09897 to 0.1333	No	ns	>0.9999	D-J
Fam129b vs. *Pgam5*	-0.3052	-0.4213 to -0.1891	Yes	****	<0.0001	D-K
Fam129b vs. *Ptma*	0.005657	-0.1105 to 0.1218	No	ns	>0.9999	D-L
Fam129b vs. *Slk*	-0.0522	-0.1683 to 0.06393	No	ns	0.9707	D-M
Fam129b vs. *Sqstm1*	-0.2847	-0.4008 to -0.1685	Yes	****	<0.0001	D-N
Fam129b vs. *Tsc22d4*	0.02105	-0.09508 to 0.1372	No	ns	>0.9999	D-O
Fam129b vs. *Wdr1*	-0.1847	-0.3008 to -0.06852	Yes	****	<0.0001	D-P
Ikbkb vs. *Mad2l1*	0.08749	-0.02864 to 0.2036	No	ns	0.3856	E-F
Ikbkb vs. *Mcm3*	0.1066	-0.009485 to 0.2228	No	ns	0.1102	E-G
Ikbkb vs. *Nfe2l1*	-0.3896	-0.5057 to -0.2735	Yes	****	<0.0001	E-H
Ikbkb vs. *Nfe2l2*	0.1057	-0.01047 to 0.2218	No	ns	0.1189	E-I
Ikbkb vs. *Palb2*	0.08891	-0.02721 to 0.2050	No	ns	0.3575	E-J
Ikbkb vs. *Pgam5*	-0.2334	-0.3496 to -0.1173	Yes	****	<0.0001	E-K
Ikbkb vs. *Ptma*	0.07742	-0.03871 to 0.1935	No	ns	0.6024	E-L
Ikbkb vs. *Slk*	0.01956	-0.09657 to 0.1357	No	ns	>0.9999	E-M
Ikbkb vs. *Sqstm1*	-0.2129	-0.3290 to -0.09677	Yes	****	<0.0001	E-N
Ikbkb vs. *Tsc22d4*	0.0928	-0.02332 to 0.2089	No	ns	0.2865	E-O
Ikbkb vs. *Wdr1*	-0.1129	-0.2290 to 0.003235	No	ns	0.0662	E-P
Mad2l1 vs. *Mcm3*	0.01915	-0.09697 to 0.1353	No	ns	>0.9999	F-G
Mad2l1 vs. *Nfe2l1*	-0.4771	-0.5932 to -0.3610	Yes	****	<0.0001	F-H
Mad2l1 vs. *Nfe2l2*	0.01817	-0.09796 to 0.1343	No	ns	>0.9999	F-I
Mad2l1 vs. *Palb2*	0.001428	-0.1147 to 0.1176	No	ns	>0.9999	F-J
Mad2l1 vs. *Pgam5*	-0.3209	-0.4371 to -0.2048	Yes	****	<0.0001	F-K
Mad2l1 vs. *Ptma*	-0.01007	-0.1262 to 0.1061	No	ns	>0.9999	F-L
Mad2l1 vs. *Slk*	-0.06793	-0.1841 to 0.04820	No	ns	0.7953	F-M
Mad2l1 vs. *Sqstm1*	-0.3004	-0.4165 to -0.1843	Yes	****	<0.0001	F-N
Mad2l1 vs. *Tsc22d4*	0.005317	-0.1108 to 0.1214	No	ns	>0.9999	F-O
Mad2l1 vs. *Wdr1*	-0.2004	-0.3165 to -0.08425	Yes	****	<0.0001	F-P
Mcm3 vs. *Nfe2l1*	-0.4963	-0.6124 to -0.3801	Yes	****	<0.0001	G-H
Mcm3 vs. *Nfe2l2*	-0.0009836	-0.1171 to 0.1151	No	ns	>0.9999	G-I
Mcm3 vs. *Palb2*	-0.01773	-0.1339 to 0.09840	No	ns	>0.9999	G-J
Mcm3 vs. *Pgam5*	-0.3401	-0.4562 to -0.2240	Yes	****	<0.0001	G-K
Mcm3 vs. *Ptma*	-0.02923	-0.1454 to 0.08690	No	ns	>0.9999	G-L
Mcm3 vs. *Slk*	-0.08708	-0.2032 to 0.02905	No	ns	0.3938	G-M
Mcm3 vs. *Sqstm1*	-0.3195	-0.4357 to -0.2034	Yes	****	<0.0001	G-N
Mcm3 vs. *Tsc22d4*	-0.01384	-0.1300 to 0.1023	No	ns	>0.9999	G-O
Mcm3 vs. *Wdr1*	-0.2195	-0.3357 to -0.1034	Yes	****	<0.0001	G-P
Nfe2l1 vs. *Nfe2l2*	0.4953	0.3791 to 0.6114	Yes	****	<0.0001	H-I
Nfe2l1 vs. *Palb2*	0.4785	0.3624 to 0.5947	Yes	****	<0.0001	H-J
Nfe2l1 vs. *Pgam5*	0.1562	0.04005 to 0.2723	Yes	***	0.0007	H-K
Nfe2l1 vs. *Ptma*	0.467	0.3509 to 0.5832	Yes	****	<0.0001	H-L
Nfe2l1 vs. *Slk*	0.4092	0.2930 to 0.5253	Yes	****	<0.0001	H-M
Nfe2l1 vs. *Sqstm1*	0.1767	0.06060 to 0.2928	Yes	****	<0.0001	H-N
Nfe2l1 vs. *Tsc22d4*	0.4824	0.3663 to 0.5985	Yes	****	<0.0001	H-O
Nfe2l1 vs. *Wdr1*	0.2767	0.1606 to 0.3928	Yes	****	<0.0001	H-P
Nfe2l2 vs. *Palb2*	-0.01674	-0.1329 to 0.09938	No	ns	>0.9999	I-J
Nfe2l2 vs. *Pgam5*	-0.3391	-0.4552 to -0.2230	Yes	****	<0.0001	I-K

Nfe2l2 vs. *Ptma*	-0.02824	-0.1444 to 0.08788	No	ns	>0.9999	I-L
Nfe2l2 vs. *Slk*	-0.0861	-0.2022 to 0.03003	No	ns	0.4139	I-M
Nfe2l2 vs. *Sqstm1*	-0.3185	-0.4347 to -0.2024	Yes	****	<0.0001	I-N
Nfe2l2 vs. *Tsc22d4*	-0.01285	-0.1290 to 0.1033	No	ns	>0.9999	I-O
Nfe2l2 vs. *Wdr1*	-0.2185	-0.3347 to -0.1024	Yes	****	<0.0001	I-P
Palb2 vs. *Pgam5*	-0.3224	-0.4385 to -0.2062	Yes	****	<0.0001	J-K
Palb2 vs. *Ptma*	-0.0115	-0.1276 to 0.1046	No	ns	>0.9999	J-L
Palb2 vs. *Slk*	-0.06935	-0.1855 to 0.04677	No	ns	0.7694	J-M
Palb2 vs. *Sqstm1*	-0.3018	-0.4179 to -0.1857	Yes	****	<0.0001	J-N
Palb2 vs. *Tsc22d4*	0.003889	-0.1122 to 0.1200	No	ns	>0.9999	J-O
Palb2 vs. *Wdr1*	-0.2018	-0.3179 to -0.08568	Yes	****	<0.0001	J-P
Pgam5 vs. *Ptma*	0.3109	0.1947 to 0.4270	Yes	****	<0.0001	K-L
Pgam5 vs. *Slk*	0.253	0.1369 to 0.3691	Yes	****	<0.0001	K-M
Pgam5 vs. *Sqstm1*	0.02055	-0.09558 to 0.1367	No	ns	>0.9999	K-N
Pgam5 vs. *Tsc22d4*	0.3262	0.2101 to 0.4424	Yes	****	<0.0001	K-O
Pgam5 vs. *Wdr1*	0.1205	0.004422 to 0.2367	Yes	*	0.0335	K-P
Ptma vs. *Slk*	-0.05785	-0.1740 to 0.05827	No	ns	0.9314	L-M
Ptma vs. *Sqstm1*	-0.2903	-0.4064 to -0.1742	Yes	****	<0.0001	L-N
Ptma vs. *Tsc22d4*	0.01539	-0.1007 to 0.1315	No	ns	>0.9999	L-O
Ptma vs. *Wdr1*	-0.1903	-0.3064 to -0.07418	Yes	****	<0.0001	L-P
Slk vs. *Sqstm1*	-0.2325	-0.3486 to -0.1163	Yes	****	<0.0001	M-N
Slk vs. *Tsc22d4*	0.07324	-0.04288 to 0.1894	No	ns	0.6922	M-O
Slk vs. *Wdr1*	-0.1325	-0.2486 to -0.01633	Yes	*	0.0103	M-P
Sqstm1 vs. *Tsc22d4*	0.3057	0.1896 to 0.4218	Yes	****	<0.0001	N-O
Sqstm1 vs. *Wdr1*	0.1	-0.01613 to 0.2161	No	ns	0.1799	N-P
Tsc22d4 vs. *Wdr1*	-0.2057	-0.3218 to -0.08957	Yes	****	<0.0001	O-P

Test details	Mean 1	Mean 2	Mean Diff.	SE of diff.	n1	n2	q	DF
Amer1 vs. *Dpp3*	0.09903	0.3181	-0.2191	0.03322	9	9	9.326	128
Amer1 vs. *Fam117b*	0.09903	0.2646	-0.1656	0.03322	9	9	7.049	128
Amer1 vs. *Fam129b*	0.09903	0.03634	0.0627	0.03322	9	9	2.669	128
Amer1 vs. *Ikbkb*	0.09903	0.1081	-0.009063	0.03322	9	9	0.3858	128
Amer1 vs. *Mad2l1*	0.09903	0.02061	0.07842	0.03322	9	9	3.338	128
Amer1 vs. *Mcm3*	0.09903	0.001456	0.09758	0.03322	9	9	4.154	128
Amer1 vs. *Nfe2l1*	0.09903	0.4977	-0.3987	0.03322	9	9	16.97	128
Amer1 vs. *Nfe2l2*	0.09903	0.002439	0.09659	0.03322	9	9	4.112	128
Amer1 vs. *Palb2*	0.09903	0.01918	0.07985	0.03322	9	9	3.399	128
Amer1 vs. *Pgam5*	0.09903	0.3415	-0.2425	0.03322	9	9	10.32	128
Amer1 vs. *Ptma*	0.09903	0.03068	0.06835	0.03322	9	9	2.91	128
Amer1 vs. *Slk*	0.09903	0.08854	0.0105	0.03322	9	9	0.4469	128
Amer1 vs. *Sqstm1*	0.09903	0.321	-0.222	0.03322	9	9	9.448	128
Amer1 vs. *Tsc22d4*	0.09903	0.01529	0.08374	0.03322	9	9	3.565	128
Amer1 vs. *Wdr1*	0.09903	0.221	-0.122	0.03322	9	9	5.191	128
Dpp3 vs. *Fam117b*	0.3181	0.2646	0.0535	0.03322	9	9	2.277	128
Dpp3 vs. *Fam129b*	0.3181	0.03634	0.2818	0.03322	9	9	11.99	128
Dpp3 vs. *Ikbkb*	0.3181	0.1081	0.21	0.03322	9	9	8.94	128
Dpp3 vs. *Mad2l1*	0.3181	0.02061	0.2975	0.03322	9	9	12.66	128

Dpp3 vs. *Mcm3*	0.3181	0.001456	0.3167	0.03322	9	9	13.48	128
Dpp3 vs. *Nfe2l1*	0.3181	0.4977	-0.1796	0.03322	9	9	7.645	128
Dpp3 vs. *Nfe2l2*	0.3181	0.002439	0.3157	0.03322	9	9	13.44	128
Dpp3 vs. *Palb2*	0.3181	0.01918	0.2989	0.03322	9	9	12.73	128
Dpp3 vs. *Pgam5*	0.3181	0.3415	-0.02342	0.03322	9	9	0.9969	128
Dpp3 vs. *Ptma*	0.3181	0.03068	0.2874	0.03322	9	9	12.24	128
Dpp3 vs. *Slk*	0.3181	0.08854	0.2296	0.03322	9	9	9.773	128
Dpp3 vs. *Sqstm1*	0.3181	0.321	-0.002871	0.03322	9	9	0.1222	128
Dpp3 vs. *Tsc22d4*	0.3181	0.01529	0.3028	0.03322	9	9	12.89	128
Dpp3 vs. *Wdr1*	0.3181	0.221	0.09713	0.03322	9	9	4.135	128
Fam117b vs. *Fam129b*	0.2646	0.03634	0.2283	0.03322	9	9	9.717	128
Fam117b vs. *Ikbkb*	0.2646	0.1081	0.1565	0.03322	9	9	6.663	128
Fam117b vs. *Mad2l1*	0.2646	0.02061	0.244	0.03322	9	9	10.39	128
Fam117b vs. *Mcm3*	0.2646	0.001456	0.2632	0.03322	9	9	11.2	128
Fam117b vs. *Nfe2l1*	0.2646	0.4977	-0.2331	0.03322	9	9	9.922	128
Fam117b vs. *Nfe2l2*	0.2646	0.002439	0.2622	0.03322	9	9	11.16	128
Fam117b vs. *Palb2*	0.2646	0.01918	0.2454	0.03322	9	9	10.45	128
Fam117b vs. *Pgam5*	0.2646	0.3415	-0.07692	0.03322	9	9	3.274	128
Fam117b vs. *Ptma*	0.2646	0.03068	0.2339	0.03322	9	9	9.958	128
Fam117b vs. *Slk*	0.2646	0.08854	0.1761	0.03322	9	9	7.495	128
Fam117b vs. *Sqstm1*	0.2646	0.321	-0.05637	0.03322	9	9	2.4	128
Fam117b vs. *Tsc22d4*	0.2646	0.01529	0.2493	0.03322	9	9	10.61	128
Fam117b vs. *Wdr1*	0.2646	0.221	0.04363	0.03322	9	9	1.857	128
Fam129b vs. *Ikbkb*	0.03634	0.1081	-0.07176	0.03322	9	9	3.055	128
Fam129b vs. *Mad2l1*	0.03634	0.02061	0.01573	0.03322	9	9	0.6695	128
Fam129b vs. *Mcm3*	0.03634	0.001456	0.03488	0.03322	9	9	1.485	128
Fam129b vs. *Nfe2l1*	0.03634	0.4977	-0.4614	0.03322	9	9	19.64	128
Fam129b vs. *Nfe2l2*	0.03634	0.002439	0.0339	0.03322	9	9	1.443	128
Fam129b vs. *Palb2*	0.03634	0.01918	0.01716	0.03322	9	9	0.7303	128
Fam129b vs. *Pgam5*	0.03634	0.3415	-0.3052	0.03322	9	9	12.99	128
Fam129b vs. *Ptma*	0.03634	0.03068	0.005657	0.03322	9	9	0.2408	128
Fam129b vs. *Slk*	0.03634	0.08854	-0.0522	0.03322	9	9	2.222	128
Fam129b vs. *Sqstm1*	0.03634	0.321	-0.2847	0.03322	9	9	12.12	128
Fam129b vs. *Tsc22d4*	0.03634	0.01529	0.02105	0.03322	9	9	0.8958	128
Fam129b vs. *Wdr1*	0.03634	0.221	-0.1847	0.03322	9	9	7.86	128
Ikbkb vs. *Mad2l1*	0.1081	0.02061	0.08749	0.03322	9	9	3.724	128
Ikbkb vs. *Mcm3*	0.1081	0.001456	0.1066	0.03322	9	9	4.54	128
Ikbkb vs. *Nfe2l1*	0.1081	0.4977	-0.3896	0.03322	9	9	16.59	128
Ikbkb vs. *Nfe2l2*	0.1081	0.002439	0.1057	0.03322	9	9	4.498	128
Ikbkb vs. *Palb2*	0.1081	0.01918	0.08891	0.03322	9	9	3.785	128
Ikbkb vs. *Pgam5*	0.1081	0.3415	-0.2334	0.03322	9	9	9.937	128
Ikbkb vs. *Ptma*	0.1081	0.03068	0.07742	0.03322	9	9	3.295	128
Ikbkb vs. *Slk*	0.1081	0.08854	0.01956	0.03322	9	9	0.8327	128
Ikbkb vs. *Sqstm1*	0.1081	0.321	-0.2129	0.03322	9	9	9.062	128
Ikbkb vs. *Tsc22d4*	0.1081	0.01529	0.0928	0.03322	9	9	3.95	128
Ikbkb vs. *Wdr1*	0.1081	0.221	-0.1129	0.03322	9	9	4.806	128
Mad2l1 vs. *Mcm3*	0.02061	0.001456	0.01915	0.03322	9	9	0.8154	128

Mad2l1 vs. *Nfe2l1*	0.02061	0.4977	-0.4771	0.03322	9	9	20.31	128
Mad2l1 vs. *Nfe2l2*	0.02061	0.002439	0.01817	0.03322	9	9	0.7735	128
Mad2l1 vs. *Palb2*	0.02061	0.01918	0.001428	0.03322	9	9	0.06078	128
Mad2l1 vs. *Pgam5*	0.02061	0.3415	-0.3209	0.03322	9	9	13.66	128
Mad2l1 vs. *Ptma*	0.02061	0.03068	-0.01007	0.03322	9	9	0.4287	128
Mad2l1 vs. *Slk*	0.02061	0.08854	-0.06793	0.03322	9	9	2.891	128
Mad2l1 vs. *Sqstm1*	0.02061	0.321	-0.3004	0.03322	9	9	12.79	128
Mad2l1 vs. *Tsc22d4*	0.02061	0.01529	0.005317	0.03322	9	9	0.2263	128
Mad2l1 vs. *Wdr1*	0.02061	0.221	-0.2004	0.03322	9	9	8.53	128
Mcm3 vs. *Nfe2l1*	0.001456	0.4977	-0.4963	0.03322	9	9	21.12	128
Mcm3 vs. *Nfe2l2*	0.001456	0.002439	-0.0009836	0.03322	9	9	0.04187	128
Mcm3 vs. *Palb2*	0.001456	0.01918	-0.01773	0.03322	9	9	0.7546	128
Mcm3 vs. *Pgam5*	0.001456	0.3415	-0.3401	0.03322	9	9	14.48	128
Mcm3 vs. *Ptma*	0.001456	0.03068	-0.02923	0.03322	9	9	1.244	128
Mcm3 vs. *Slk*	0.001456	0.08854	-0.08708	0.03322	9	9	3.707	128
Mcm3 vs. *Sqstm1*	0.001456	0.321	-0.3195	0.03322	9	9	13.6	128
Mcm3 vs. *Tsc22d4*	0.001456	0.01529	-0.01384	0.03322	9	9	0.589	128
Mcm3 vs. *Wdr1*	0.001456	0.221	-0.2195	0.03322	9	9	9.345	128
Nfe2l1 vs. *Nfe2l2*	0.4977	0.002439	0.4953	0.03322	9	9	21.08	128
Nfe2l1 vs. *Palb2*	0.4977	0.01918	0.4785	0.03322	9	9	20.37	128
Nfe2l1 vs. *Pgam5*	0.4977	0.3415	0.1562	0.03322	9	9	6.648	128
Nfe2l1 vs. *Ptma*	0.4977	0.03068	0.467	0.03322	9	9	19.88	128
Nfe2l1 vs. *Slk*	0.4977	0.08854	0.4092	0.03322	9	9	17.42	128
Nfe2l1 vs. *Sqstm1*	0.4977	0.321	0.1767	0.03322	9	9	7.523	128
Nfe2l1 vs. *Tsc22d4*	0.4977	0.01529	0.4824	0.03322	9	9	20.54	128
Nfe2l1 vs. *Wdr1*	0.4977	0.221	0.2767	0.03322	9	9	11.78	128
Nfe2l2 vs. *Palb2*	0.002439	0.01918	-0.01674	0.03322	9	9	0.7127	128
Nfe2l2 vs. *Pgam5*	0.002439	0.3415	-0.3391	0.03322	9	9	14.43	128
Nfe2l2 vs. *Ptma*	0.002439	0.03068	-0.02824	0.03322	9	9	1.202	128
Nfe2l2 vs. *Slk*	0.002439	0.08854	-0.0861	0.03322	9	9	3.665	128
Nfe2l2 vs. *Sqstm1*	0.002439	0.321	-0.3185	0.03322	9	9	13.56	128
Nfe2l2 vs. *Tsc22d4*	0.002439	0.01529	-0.01285	0.03322	9	9	0.5472	128
Nfe2l2 vs. *Wdr1*	0.002439	0.221	-0.2185	0.03322	9	9	9.303	128
Palb2 vs. *Pgam5*	0.01918	0.3415	-0.3224	0.03322	9	9	13.72	128
Palb2 vs. *Ptma*	0.01918	0.03068	-0.0115	0.03322	9	9	0.4895	128
Palb2 vs. *Slk*	0.01918	0.08854	-0.06935	0.03322	9	9	2.952	128
Palb2 vs. *Sqstm1*	0.01918	0.321	-0.3018	0.03322	9	9	12.85	128
Palb2 vs. *Tsc22d4*	0.01918	0.01529	0.003889	0.03322	9	9	0.1656	128
Palb2 vs. *Wdr1*	0.01918	0.221	-0.2018	0.03322	9	9	8.59	128
Pgam5 vs. *Ptma*	0.3415	0.03068	0.3109	0.03322	9	9	13.23	128
Pgam5 vs. *Slk*	0.3415	0.08854	0.253	0.03322	9	9	10.77	128
Pgam5 vs. *Sqstm1*	0.3415	0.321	0.02055	0.03322	9	9	0.8747	128
Pgam5 vs. *Tsc22d4*	0.3415	0.01529	0.3262	0.03322	9	9	13.89	128
Pgam5 vs. *Wdr1*	0.3415	0.221	0.1205	0.03322	9	9	5.131	128
Ptma vs. *Slk*	0.03068	0.08854	-0.05785	0.03322	9	9	2.463	128
Ptma vs. *Sqstm1*	0.03068	0.321	-0.2903	0.03322	9	9	12.36	128
Ptma vs. *Tsc22d4*	0.03068	0.01529	0.01539	0.03322	9	9	0.655	128

Ptma vs. *Wdr1*	0.03068	0.221	-0.1903	0.03322	9	9	8.101	128
Slk vs. *Sqstm1*	0.08854	0.321	-0.2325	0.03322	9	9	9.895	128
Slk vs. *Tsc22d4*	0.08854	0.01529	0.07324	0.03322	9	9	3.118	128
Slk vs. *Wdr1*	0.08854	0.221	-0.1325	0.03322	9	9	5.638	128
Sqstm1 vs. *Tsc22d4*	0.321	0.01529	0.3057	0.03322	9	9	13.01	128
Sqstm1 vs. *Wdr1*	0.321	0.221	0.1	0.03322	9	9	4.257	128
Tsc22d4 vs. *Wdr1*	0.01529	0.221	-0.2057	0.03322	9	9	8.756	128

Keap1-interact-glut-raw

Number of families	1
Number of comparisons per family	120
Alpha	0.05

Tukey's multiple comparisons test	Mean Diff.	95.00% CI of diff.	Significant?	Summary	Adjusted P Value	
Amer1 vs. *Dpp3*	-1.088	-5.108 to 2.931	No	ns	>0.9999	A-B
Amer1 vs. *Fam117b*	-5.209	-9.228 to -1.190	Yes	**	0.0011	A-C
Amer1 vs. *Fam129b*	0.8036	-3.216 to 4.823	No	ns	>0.9999	A-D
Amer1 vs. *Ikbkb*	-1.018	-5.037 to 3.002	No	ns	>0.9999	A-E
Amer1 vs. *Mad2l1*	1.064	-2.955 to 5.083	No	ns	>0.9999	A-F
Amer1 vs. *Mcm3*	1.543	-2.476 to 5.562	No	ns	0.9949	A-G
Amer1 vs. *Nfe2l1*	-40.28	-44.30 to -36.27	Yes	****	<0.0001	A-H
Amer1 vs. *Nfe2l2*	1.493	-2.526 to 5.512	No	ns	0.9964	A-I
Amer1 vs. *Palb2*	1.447	-2.572 to 5.466	No	ns	0.9974	A-J
Amer1 vs. *Pgam5*	-2.949	-6.968 to 1.070	No	ns	0.4518	A-K
Amer1 vs. *Ptma*	-5.328	-9.347 to -1.309	Yes	***	0.0007	A-L
Amer1 vs. *Slk*	-20.69	-24.71 to -16.68	Yes	****	<0.0001	A-M
Amer1 vs. *Sqstm1*	-29.68	-33.70 to -25.66	Yes	****	<0.0001	A-N
Amer1 vs. *Tsc22d4*	-0.9286	-4.948 to 3.091	No	ns	>0.9999	A-O
Amer1 vs. *Wdr1*	-8.436	-12.45 to -4.416	Yes	****	<0.0001	A-P
Dpp3 vs. *Fam117b*	-4.12	-8.140 to -0.1012	Yes	*	0.038	B-C
Dpp3 vs. *Fam129b*	1.892	-2.127 to 5.911	No	ns	0.9634	B-D
Dpp3 vs. *Ikbkb*	0.07084	-3.948 to 4.090	No	ns	>0.9999	B-E
Dpp3 vs. *Mad2l1*	2.152	-1.867 to 6.172	No	ns	0.8977	B-F
Dpp3 vs. *Mcm3*	2.632	-1.388 to 6.651	No	ns	0.6553	B-G
Dpp3 vs. *Nfe2l1*	-39.2	-43.22 to -35.18	Yes	****	<0.0001	B-H
Dpp3 vs. *Nfe2l2*	2.582	-1.438 to 6.601	No	ns	0.6863	B-I
Dpp3 vs. *Palb2*	2.535	-1.484 to 6.555	No	ns	0.7141	B-J
Dpp3 vs. *Pgam5*	-1.86	-5.880 to 2.159	No	ns	0.9684	B-K
Dpp3 vs. *Ptma*	-4.24	-8.259 to -0.2206	Yes	*	0.0271	B-L
Dpp3 vs. *Slk*	-19.61	-23.63 to -15.59	Yes	****	<0.0001	B-M
Dpp3 vs. *Sqstm1*	-28.59	-32.61 to -24.57	Yes	****	<0.0001	B-N
Dpp3 vs. *Tsc22d4*	0.1598	-3.859 to 4.179	No	ns	>0.9999	B-O
Dpp3 vs. *Wdr1*	-7.347	-11.37 to -3.328	Yes	****	<0.0001	B-P
Fam117b vs. *Fam129b*	6.012	1.993 to 10.03	Yes	****	<0.0001	C-D
Fam117b vs. *Ikbkb*	4.191	0.1720 to 8.211	Yes	*	0.0312	C-E
Fam117b vs. *Mad2l1*	6.273	2.253 to 10.29	Yes	****	<0.0001	C-F

Fam117b vs. *Mcm3*	6.752	2.733 to 10.77	Yes	****	<0.0001	C-G
Fam117b vs. *Nfe2l1*	-35.08	-39.09 to -31.06	Yes	****	<0.0001	C-H
Fam117b vs. *Nfe2l2*	6.702	2.683 to 10.72	Yes	****	<0.0001	C-I
Fam117b vs. *Palb2*	6.656	2.637 to 10.68	Yes	****	<0.0001	C-J
Fam117b vs. *Pgam5*	2.26	-1.759 to 6.279	No	ns	0.8562	C-K
Fam117b vs. *Ptma*	-0.1194	-4.139 to 3.900	No	ns	>0.9999	C-L
Fam117b vs. *Slk*	-15.49	-19.51 to -11.47	Yes	****	<0.0001	C-M
Fam117b vs. *Sqstm1*	-24.47	-28.49 to -20.45	Yes	****	<0.0001	C-N
Fam117b vs. *Tsc22d4*	4.28	0.2610 to 8.300	Yes	*	0.0241	C-O
Fam117b vs. *Wdr1*	-3.227	-7.246 to 0.7925	No	ns	0.2919	C-P
Fam129b vs. *Ikbkb*	-1.821	-5.840 to 2.198	No	ns	0.9739	D-E
Fam129b vs. *Mad2l1*	0.2603	-3.759 to 4.280	No	ns	>0.9999	D-F
Fam129b vs. *Mcm3*	0.7396	-3.280 to 4.759	No	ns	>0.9999	D-G
Fam129b vs. *Nfe2l1*	-41.09	-45.11 to -37.07	Yes	****	<0.0001	D-H
Fam129b vs. *Nfe2l2*	0.6895	-3.330 to 4.709	No	ns	>0.9999	D-I
Fam129b vs. *Palb2*	0.6434	-3.376 to 4.663	No	ns	>0.9999	D-J
Fam129b vs. *Pgam5*	-3.752	-7.772 to 0.2668	No	ns	0.0981	D-K
Fam129b vs. *Ptma*	-6.132	-10.15 to -2.113	Yes	****	<0.0001	D-L
Fam129b vs. *Slk*	-21.5	-25.52 to -17.48	Yes	****	<0.0001	D-M
Fam129b vs. *Sqstm1*	-30.49	-34.50 to -26.47	Yes	****	<0.0001	D-N
Fam129b vs. *Tsc22d4*	-1.732	-5.751 to 2.287	No	ns	0.9836	D-O
Fam129b vs. *Wdr1*	-9.239	-13.26 to -5.220	Yes	****	<0.0001	D-P
Ikbkb vs. *Mad2l1*	2.081	-1.938 to 6.101	No	ns	0.9203	E-F
Ikbkb vs. *Mcm3*	2.561	-1.459 to 6.580	No	ns	0.6989	E-G
Ikbkb vs. *Nfe2l1*	-39.27	-43.29 to -35.25	Yes	****	<0.0001	E-H
Ikbkb vs. *Nfe2l2*	2.511	-1.509 to 6.530	No	ns	0.7286	E-I
Ikbkb vs. *Palb2*	2.465	-1.555 to 6.484	No	ns	0.7549	E-J
Ikbkb vs. *Pgam5*	-1.931	-5.951 to 2.088	No	ns	0.9564	E-K
Ikbkb vs. *Ptma*	-4.311	-8.330 to -0.2914	Yes	*	0.0221	E-L
Ikbkb vs. *Slk*	-19.68	-23.70 to -15.66	Yes	****	<0.0001	E-M
Ikbkb vs. *Sqstm1*	-28.66	-32.68 to -24.64	Yes	****	<0.0001	E-N
Ikbkb vs. *Tsc22d4*	0.08896	-3.930 to 4.108	No	ns	>0.9999	E-O
Ikbkb vs. *Wdr1*	-7.418	-11.44 to -3.399	Yes	****	<0.0001	E-P
Mad2l1 vs. *Mcm3*	0.4793	-3.540 to 4.499	No	ns	>0.9999	F-G
Mad2l1 vs. *Nfe2l1*	-41.35	-45.37 to -37.33	Yes	****	<0.0001	F-H
Mad2l1 vs. *Nfe2l2*	0.4292	-3.590 to 4.449	No	ns	>0.9999	F-I
Mad2l1 vs. *Palb2*	0.3831	-3.636 to 4.402	No	ns	>0.9999	F-J
Mad2l1 vs. *Pgam5*	-4.013	-8.032 to 0.006524	No	ns	0.0509	F-K
Mad2l1 vs. *Ptma*	-6.392	-10.41 to -2.373	Yes	****	<0.0001	F-L
Mad2l1 vs. *Slk*	-21.76	-25.78 to -17.74	Yes	****	<0.0001	F-M
Mad2l1 vs. *Sqstm1*	-30.75	-34.76 to -26.73	Yes	****	<0.0001	F-N
Mad2l1 vs. *Tsc22d4*	-1.992	-6.012 to 2.027	No	ns	0.9435	F-O
Mad2l1 vs. *Wdr1*	-9.499	-13.52 to -5.480	Yes	****	<0.0001	F-P
Mcm3 vs. *Nfe2l1*	-41.83	-45.85 to -37.81	Yes	****	<0.0001	G-H
Mcm3 vs. *Nfe2l2*	-0.05009	-4.069 to 3.969	No	ns	>0.9999	G-I
Mcm3 vs. *Palb2*	-0.09621	-4.115 to 3.923	No	ns	>0.9999	G-J
Mcm3 vs. *Pgam5*	-4.492	-8.511 to -0.4728	Yes	*	0.0127	G-K

Mcm3 vs. *Ptma*	-6.871	-10.89 to -2.852	Yes	****	<0.0001	G-L
Mcm3 vs. *Slk*	-22.24	-26.26 to -18.22	Yes	****	<0.0001	G-M
Mcm3 vs. *Sqstm1*	-31.22	-35.24 to -27.21	Yes	****	<0.0001	G-N
Mcm3 vs. *Tsc22d4*	-2.472	-6.491 to 1.547	No	ns	0.7509	G-O
Mcm3 vs. *Wdr1*	-9.979	-14.00 to -5.960	Yes	****	<0.0001	G-P
Nfe2l1 vs. *Nfe2l2*	41.78	37.76 to 45.80	Yes	****	<0.0001	H-I
Nfe2l1 vs. *Palb2*	41.73	37.71 to 45.75	Yes	****	<0.0001	H-J
Nfe2l1 vs. *Pgam5*	37.34	33.32 to 41.35	Yes	****	<0.0001	H-K
Nfe2l1 vs. *Ptma*	34.96	30.94 to 38.98	Yes	****	<0.0001	H-L
Nfe2l1 vs. *Slk*	19.59	15.57 to 23.61	Yes	****	<0.0001	H-M
Nfe2l1 vs. *Sqstm1*	10.6	6.584 to 14.62	Yes	****	<0.0001	H-N
Nfe2l1 vs. *Tsc22d4*	39.36	35.34 to 43.38	Yes	****	<0.0001	H-O
Nfe2l1 vs. *Wdr1*	31.85	27.83 to 35.87	Yes	****	<0.0001	H-P
Nfe2l2 vs. *Palb2*	-0.04613	-4.065 to 3.973	No	ns	>0.9999	I-J
Nfe2l2 vs. *Pgam5*	-4.442	-8.461 to -0.4227	Yes	*	0.0149	I-K
Nfe2l2 vs. *Ptma*	-6.821	-10.84 to -2.802	Yes	****	<0.0001	I-L
Nfe2l2 vs. *Slk*	-22.19	-26.21 to -18.17	Yes	****	<0.0001	I-M
Nfe2l2 vs. *Sqstm1*	-31.17	-35.19 to -27.16	Yes	****	<0.0001	I-N
Nfe2l2 vs. *Tsc22d4*	-2.422	-6.441 to 1.598	No	ns	0.7784	I-O
Nfe2l2 vs. *Wdr1*	-9.929	-13.95 to -5.909	Yes	****	<0.0001	I-P
Palb2 vs. *Pgam5*	-4.396	-8.415 to -0.3766	Yes	*	0.0171	J-K
Palb2 vs. *Ptma*	-6.775	-10.79 to -2.756	Yes	****	<0.0001	J-L
Palb2 vs. *Slk*	-22.14	-26.16 to -18.12	Yes	****	<0.0001	J-M
Palb2 vs. *Sqstm1*	-31.13	-35.15 to -27.11	Yes	****	<0.0001	J-N
Palb2 vs. *Tsc22d4*	-2.376	-6.395 to 1.644	No	ns	0.8023	J-O
Palb2 vs. *Wdr1*	-9.883	-13.90 to -5.863	Yes	****	<0.0001	J-P
Pgam5 vs. *Ptma*	-2.379	-6.399 to 1.640	No	ns	0.8004	K-L
Pgam5 vs. *Slk*	-17.75	-21.77 to -13.73	Yes	****	<0.0001	K-M
Pgam5 vs. *Sqstm1*	-26.73	-30.75 to -22.71	Yes	****	<0.0001	K-N
Pgam5 vs. *Tsc22d4*	2.02	-1.999 to 6.040	No	ns	0.9368	K-O
Pgam5 vs. *Wdr1*	-5.487	-9.506 to -1.467	Yes	***	0.0004	K-P
Ptma vs. *Slk*	-15.37	-19.39 to -11.35	Yes	****	<0.0001	L-M
Ptma vs. *Sqstm1*	-24.35	-28.37 to -20.33	Yes	****	<0.0001	L-N
Ptma vs. *Tsc22d4*	4.4	0.3804 to 8.419	Yes	*	0.0169	L-O
Ptma vs. *Wdr1*	-3.107	-7.127 to 0.9119	No	ns	0.3566	L-P
Slk vs. *Sqstm1*	-8.987	-13.01 to -4.967	Yes	****	<0.0001	M-N
Slk vs. *Tsc22d4*	19.77	15.75 to 23.79	Yes	****	<0.0001	M-O
Slk vs. *Wdr1*	12.26	8.240 to 16.28	Yes	****	<0.0001	M-P
Sqstm1 vs. *Tsc22d4*	28.75	24.73 to 32.77	Yes	****	<0.0001	N-O
Sqstm1 vs. *Wdr1*	21.25	17.23 to 25.27	Yes	****	<0.0001	N-P
Tsc22d4 vs. *Wdr1*	-7.507	-11.53 to -3.488	Yes	****	<0.0001	O-P

Test details	Mean 1	Mean 2	Mean Diff.	SE of diff.	n1	n2	q	DF
Amer1 vs. *Dpp3*	1.561	2.65	-1.088	1.166	26	26	1.32	400
Amer1 vs. *Fam117b*	1.561	6.77	-5.209	1.166	26	26	6.32	400
Amer1 vs. *Fam129b*	1.561	0.7579	0.8036	1.166	26	26	0.975	400
Amer1 vs. *Ikbkb*	1.561	2.579	-1.018	1.166	26	26	1.235	400

Amer1 vs. *Mad2l1*	1.561	0.4976	1.064	1.166	26	26	1.291	400
Amer1 vs. *Mcm3*	1.561	0.01825	1.543	1.166	26	26	1.872	400
Amer1 vs. *Nfe2l1*	1.561	41.85	-40.28	1.166	26	26	48.88	400
Amer1 vs. *Nfe2l2*	1.561	0.06834	1.493	1.166	26	26	1.812	400
Amer1 vs. *Palb2*	1.561	0.1145	1.447	1.166	26	26	1.756	400
Amer1 vs. *Pgam5*	1.561	4.51	-2.949	1.166	26	26	3.578	400
Amer1 vs. *Ptma*	1.561	6.89	-5.328	1.166	26	26	6.465	400
Amer1 vs. *Slk*	1.561	22.26	-20.69	1.166	26	26	25.11	400
Amer1 vs. *Sqstm1*	1.561	31.24	-29.68	1.166	26	26	36.01	400
Amer1 vs. *Tsc22d4*	1.561	2.49	-0.9286	1.166	26	26	1.127	400
Amer1 vs. *Wdr1*	1.561	9.997	-8.436	1.166	26	26	10.23	400
Dpp3 vs. *Fam117b*	2.65	6.77	-4.12	1.166	26	26	4.999	400
Dpp3 vs. *Fam129b*	2.65	0.7579	1.892	1.166	26	26	2.295	400
Dpp3 vs. *Ikbkb*	2.65	2.579	0.07084	1.166	26	26	0.08595	400
Dpp3 vs. *Mad2l1*	2.65	0.4976	2.152	1.166	26	26	2.611	400
Dpp3 vs. *Mcm3*	2.65	0.01825	2.632	1.166	26	26	3.193	400
Dpp3 vs. *Nfe2l1*	2.65	41.85	-39.2	1.166	26	26	47.56	400
Dpp3 vs. *Nfe2l2*	2.65	0.06834	2.582	1.166	26	26	3.132	400
Dpp3 vs. *Palb2*	2.65	0.1145	2.535	1.166	26	26	3.076	400
Dpp3 vs. *Pgam5*	2.65	4.51	-1.86	1.166	26	26	2.257	400
Dpp3 vs. *Ptma*	2.65	6.89	-4.24	1.166	26	26	5.144	400
Dpp3 vs. *Slk*	2.65	22.26	-19.61	1.166	26	26	23.79	400
Dpp3 vs. *Sqstm1*	2.65	31.24	-28.59	1.166	26	26	34.69	400
Dpp3 vs. *Tsc22d4*	2.65	2.49	0.1598	1.166	26	26	0.1939	400
Dpp3 vs. *Wdr1*	2.65	9.997	-7.347	1.166	26	26	8.914	400
Fam117b vs. *Fam129b*	6.77	0.7579	6.012	1.166	26	26	7.295	400
Fam117b vs. *Ikbkb*	6.77	2.579	4.191	1.166	26	26	5.085	400
Fam117b vs. *Mad2l1*	6.77	0.4976	6.273	1.166	26	26	7.61	400
Fam117b vs. *Mcm3*	6.77	0.01825	6.752	1.166	26	26	8.192	400
Fam117b vs. *Nfe2l1*	6.77	41.85	-35.08	1.166	26	26	42.56	400
Fam117b vs. *Nfe2l2*	6.77	0.06834	6.702	1.166	26	26	8.131	400
Fam117b vs. *Palb2*	6.77	0.1145	6.656	1.166	26	26	8.075	400
Fam117b vs. *Pgam5*	6.77	4.51	2.26	1.166	26	26	2.742	400
Fam117b vs. *Ptma*	6.77	6.89	-0.1194	1.166	26	26	0.1448	400
Fam117b vs. *Slk*	6.77	22.26	-15.49	1.166	26	26	18.79	400
Fam117b vs. *Sqstm1*	6.77	31.24	-24.47	1.166	26	26	29.69	400
Fam117b vs. *Tsc22d4*	6.77	2.49	4.28	1.166	26	26	5.193	400
Fam117b vs. *Wdr1*	6.77	9.997	-3.227	1.166	26	26	3.915	400
Fam129b vs. *Ikbkb*	0.7579	2.579	-1.821	1.166	26	26	2.21	400
Fam129b vs. *Mad2l1*	0.7579	0.4976	0.2603	1.166	26	26	0.3158	400
Fam129b vs. *Mcm3*	0.7579	0.01825	0.7396	1.166	26	26	0.8974	400
Fam129b vs. *Nfe2l1*	0.7579	41.85	-41.09	1.166	26	26	49.85	400
Fam129b vs. *Nfe2l2*	0.7579	0.06834	0.6895	1.166	26	26	0.8366	400
Fam129b vs. *Palb2*	0.7579	0.1145	0.6434	1.166	26	26	0.7806	400
Fam129b vs. *Pgam5*	0.7579	4.51	-3.752	1.166	26	26	4.553	400
Fam129b vs. *Ptma*	0.7579	6.89	-6.132	1.166	26	26	7.44	400
Fam129b vs. *Slk*	0.7579	22.26	-21.5	1.166	26	26	26.08	400

Fam129b vs. *Sqstm1*	0.7579	31.24	-30.49	1.166	26	26	36.99	400
Fam129b vs. *Tsc22d4*	0.7579	2.49	-1.732	1.166	26	26	2.102	400
Fam129b vs. *Wdr1*	0.7579	9.997	-9.239	1.166	26	26	11.21	400
Ikbkb vs. *Mad2l1*	2.579	0.4976	2.081	1.166	26	26	2.525	400
Ikbkb vs. *Mcm3*	2.579	0.01825	2.561	1.166	26	26	3.107	400
Ikbkb vs. *Nfe2l1*	2.579	41.85	-39.27	1.166	26	26	47.64	400
Ikbkb vs. *Nfe2l2*	2.579	0.06834	2.511	1.166	26	26	3.046	400
Ikbkb vs. *Palb2*	2.579	0.1145	2.465	1.166	26	26	2.99	400
Ikbkb vs. *Pgam5*	2.579	4.51	-1.931	1.166	26	26	2.343	400
Ikbkb vs. *Ptma*	2.579	6.89	-4.311	1.166	26	26	5.23	400
Ikbkb vs. *Slk*	2.579	22.26	-19.68	1.166	26	26	23.87	400
Ikbkb vs. *Sqstm1*	2.579	31.24	-28.66	1.166	26	26	34.78	400
Ikbkb vs. *Tsc22d4*	2.579	2.49	0.08896	1.166	26	26	0.1079	400
Ikbkb vs. *Wdr1*	2.579	9.997	-7.418	1.166	26	26	9	400
Mad2l1 vs. *Mcm3*	0.4976	0.01825	0.4793	1.166	26	26	0.5816	400
Mad2l1 vs. *Nfe2l1*	0.4976	41.85	-41.35	1.166	26	26	50.17	400
Mad2l1 vs. *Nfe2l2*	0.4976	0.06834	0.4292	1.166	26	26	0.5208	400
Mad2l1 vs. *Palb2*	0.4976	0.1145	0.3831	1.166	26	26	0.4648	400
Mad2l1 vs. *Pgam5*	0.4976	4.51	-4.013	1.166	26	26	4.869	400
Mad2l1 vs. *Ptma*	0.4976	6.89	-6.392	1.166	26	26	7.755	400
Mad2l1 vs. *Slk*	0.4976	22.26	-21.76	1.166	26	26	26.4	400
Mad2l1 vs. *Sqstm1*	0.4976	31.24	-30.75	1.166	26	26	37.3	400
Mad2l1 vs. *Tsc22d4*	0.4976	2.49	-1.992	1.166	26	26	2.417	400
Mad2l1 vs. *Wdr1*	0.4976	9.997	-9.499	1.166	26	26	11.53	400
Mcm3 vs. *Nfe2l1*	0.01825	41.85	-41.83	1.166	26	26	50.75	400
Mcm3 vs. *Nfe2l2*	0.01825	0.06834	-0.05009	1.166	26	26	0.06077	400
Mcm3 vs. *Palb2*	0.01825	0.1145	-0.09621	1.166	26	26	0.1167	400
Mcm3 vs. *Pgam5*	0.01825	4.51	-4.492	1.166	26	26	5.45	400
Mcm3 vs. *Ptma*	0.01825	6.89	-6.871	1.166	26	26	8.337	400
Mcm3 vs. *Slk*	0.01825	22.26	-22.24	1.166	26	26	26.98	400
Mcm3 vs. *Sqstm1*	0.01825	31.24	-31.22	1.166	26	26	37.88	400
Mcm3 vs. *Tsc22d4*	0.01825	2.49	-2.472	1.166	26	26	2.999	400
Mcm3 vs. *Wdr1*	0.01825	9.997	-9.979	1.166	26	26	12.11	400
Nfe2l1 vs. *Nfe2l2*	41.85	0.06834	41.78	1.166	26	26	50.69	400
Nfe2l1 vs. *Palb2*	41.85	0.1145	41.73	1.166	26	26	50.63	400
Nfe2l1 vs. *Pgam5*	41.85	4.51	37.34	1.166	26	26	45.3	400
Nfe2l1 vs. *Ptma*	41.85	6.89	34.96	1.166	26	26	42.41	400
Nfe2l1 vs. *Slk*	41.85	22.26	19.59	1.166	26	26	23.77	400
Nfe2l1 vs. *Sqstm1*	41.85	31.24	10.6	1.166	26	26	12.86	400
Nfe2l1 vs. *Tsc22d4*	41.85	2.49	39.36	1.166	26	26	47.75	400
Nfe2l1 vs. *Wdr1*	41.85	9.997	31.85	1.166	26	26	38.64	400
Nfe2l2 vs. *Palb2*	0.06834	0.1145	-0.04613	1.166	26	26	0.05596	400
Nfe2l2 vs. *Pgam5*	0.06834	4.51	-4.442	1.166	26	26	5.389	400
Nfe2l2 vs. *Ptma*	0.06834	6.89	-6.821	1.166	26	26	8.276	400
Nfe2l2 vs. *Slk*	0.06834	22.26	-22.19	1.166	26	26	26.92	400
Nfe2l2 vs. *Sqstm1*	0.06834	31.24	-31.17	1.166	26	26	37.82	400
Nfe2l2 vs. *Tsc22d4*	0.06834	2.49	-2.422	1.166	26	26	2.938	400

Nfe2l2 vs. *Wdr1*	0.06834	9.997	-9.929	1.166	26	26	12.05	400
Palb2 vs. *Pgam5*	0.1145	4.51	-4.396	1.166	26	26	5.333	400
Palb2 vs. *Ptma*	0.1145	6.89	-6.775	1.166	26	26	8.22	400
Palb2 vs. *Slk*	0.1145	22.26	-22.14	1.166	26	26	26.86	400
Palb2 vs. *Sqstm1*	0.1145	31.24	-31.13	1.166	26	26	37.77	400
Palb2 vs. *Tsc22d4*	0.1145	2.49	-2.376	1.166	26	26	2.882	400
Palb2 vs. *Wdr1*	0.1145	9.997	-9.883	1.166	26	26	11.99	400
Pgam5 vs. *Ptma*	4.51	6.89	-2.379	1.166	26	26	2.887	400
Pgam5 vs. *Slk*	4.51	22.26	-17.75	1.166	26	26	21.53	400
Pgam5 vs. *Sqstm1*	4.51	31.24	-26.73	1.166	26	26	32.43	400
Pgam5 vs. *Tsc22d4*	4.51	2.49	2.02	1.166	26	26	2.451	400
Pgam5 vs. *Wdr1*	4.51	9.997	-5.487	1.166	26	26	6.657	400
Ptma vs. *Slk*	6.89	22.26	-15.37	1.166	26	26	18.64	400
Ptma vs. *Sqstm1*	6.89	31.24	-24.35	1.166	26	26	29.55	400
Ptma vs. *Tsc22d4*	6.89	2.49	4.4	1.166	26	26	5.338	400
Ptma vs. *Wdr1*	6.89	9.997	-3.107	1.166	26	26	3.77	400
Slk vs. *Sqstm1*	22.26	31.24	-8.987	1.166	26	26	10.9	400
Slk vs. *Tsc22d4*	22.26	2.49	19.77	1.166	26	26	23.98	400
Slk vs. *Wdr1*	22.26	9.997	12.26	1.166	26	26	14.87	400
Sqstm1 vs. *Tsc22d4*	31.24	2.49	28.75	1.166	26	26	34.88	400
Sqstm1 vs. *Wdr1*	31.24	9.997	21.25	1.166	26	26	25.78	400
Tsc22d4 vs. *Wdr1*	2.49	9.997	-7.507	1.166	26	26	9.108	400

Keap1-interact-glut-rel

Number of families	1
Number of comparisons per family	120
Alpha	0.05

Tukey's multiple comparisons test	Mean Diff.	95.00% CI of diff.	Significant?	Summary	Adjusted P Value		
Amer1 vs. *Dpp3*	-0.1903	-0.2568 to -0.1237	Yes	****	<0.0001		A-B
Amer1 vs. *Fam117b*	-0.2322	-0.2988 to -0.1656	Yes	****	<0.0001		A-C
Amer1 vs. *Fam129b*	0.07134	0.004753 to 0.1379	Yes	*		0.0224	A-D
Amer1 vs. *Ikbkb*	-0.01459	-0.08118 to 0.05200	No	ns	>0.9999		A-E
Amer1 vs. *Mad2l1*	0.088	0.02141 to 0.1546	Yes	***		0.0008	A-F
Amer1 vs. *Mcm3*	0.1067	0.04008 to 0.1733	Yes	****	<0.0001		A-G
Amer1 vs. *Nfe2l1*	-0.326	-0.3926 to -0.2594	Yes	****	<0.0001		A-H
Amer1 vs. *Nfe2l2*	0.1058	0.03921 to 0.1724	Yes	****	<0.0001		A-I
Amer1 vs. *Palb2*	0.07187	0.005280 to 0.1385	Yes	*		0.0204	A-J
Amer1 vs. *Pgam5*	-0.372	-0.4386 to -0.3054	Yes	****	<0.0001		A-K
Amer1 vs. *Ptma*	0.06088	-0.005709 to 0.1275	No	ns		0.1176	A-L
Amer1 vs. *Slk*	-0.1489	-0.2155 to -0.08231	Yes	****	<0.0001		A-M
Amer1 vs. *Sqstm1*	-0.3234	-0.3900 to -0.2568	Yes	****	<0.0001		A-N
Amer1 vs. *Tsc22d4*	0.1004	0.03383 to 0.1670	Yes	****	<0.0001		A-O
Amer1 vs. *Wdr1*	-0.0714	-0.1380 to -0.004810	Yes	*		0.0221	A-P
Dpp3 vs. *Fam117b*	-0.04197	-0.1086 to 0.02462	No	ns		0.7152	B-C
Dpp3 vs. *Fam129b*	0.2616	0.1950 to 0.3282	Yes	****	<0.0001		B-D

Dpp3 vs. *Ikbkb*	0.1757	0.1091 to 0.2423	Yes	****	<0.0001		B-E
Dpp3 vs. *Mad2l1*	0.2783	0.2117 to 0.3449	Yes	****	<0.0001		B-F
Dpp3 vs. *Mcm3*	0.2969	0.2303 to 0.3635	Yes	****	<0.0001		B-G
Dpp3 vs. *Nfe2l1*	-0.1358	-0.2024 to -0.06918	Yes	****	<0.0001		B-H
Dpp3 vs. *Nfe2l2*	0.2961	0.2295 to 0.3626	Yes	****	<0.0001		B-I
Dpp3 vs. *Palb2*	0.2621	0.1955 to 0.3287	Yes	****	<0.0001		B-J
Dpp3 vs. *Pgam5*	-0.1818	-0.2484 to -0.1152	Yes	****	<0.0001		B-K
Dpp3 vs. *Ptma*	0.2511	0.1846 to 0.3177	Yes	****	<0.0001		B-L
Dpp3 vs. *Slk*	0.04136	-0.02523 to 0.1079	No	ns		0.7369	B-M
Dpp3 vs. *Sqstm1*	-0.1331	-0.1997 to -0.06653	Yes	****	<0.0001		B-N
Dpp3 vs. *Tsc22d4*	0.2907	0.2241 to 0.3573	Yes	****	<0.0001		B-O
Dpp3 vs. *Wdr1*	0.1189	0.05227 to 0.1855	Yes	****	<0.0001		B-P
Fam117b vs. *Fam129b*	0.3036	0.2370 to 0.3702	Yes	****	<0.0001		C-D
Fam117b vs. *Ikbkb*	0.2176	0.1511 to 0.2842	Yes	****	<0.0001		C-E
Fam117b vs. *Mad2l1*	0.3202	0.2536 to 0.3868	Yes	****	<0.0001		C-F
Fam117b vs. *Mcm3*	0.3389	0.2723 to 0.4055	Yes	****	<0.0001		C-G
Fam117b vs. *Nfe2l1*	-0.0938	-0.1604 to -0.02721	Yes	***		0.0002	C-H
Fam117b vs. *Nfe2l2*	0.338	0.2714 to 0.4046	Yes	****	<0.0001		C-I
Fam117b vs. *Palb2*	0.3041	0.2375 to 0.3707	Yes	****	<0.0001		C-J
Fam117b vs. *Pgam5*	-0.1398	-0.2064 to -0.07320	Yes	****	<0.0001		C-K
Fam117b vs. *Ptma*	0.2931	0.2265 to 0.3597	Yes	****	<0.0001		C-L
Fam117b vs. *Slk*	0.08333	0.01674 to 0.1499	Yes	**		0.0021	C-M
Fam117b vs. *Sqstm1*	-0.09115	-0.1577 to -0.02456	Yes	***		0.0004	C-N
Fam117b vs. *Tsc22d4*	0.3326	0.2661 to 0.3992	Yes	****	<0.0001		C-O
Fam117b vs. *Wdr1*	0.1608	0.09425 to 0.2274	Yes	****	<0.0001		C-P
Fam129b vs. *Ikbkb*	-0.08593	-0.1525 to -0.01934	Yes	**		0.0012	D-E
Fam129b vs. *Mad2l1*	0.01666	-0.04993 to 0.08325	No	ns	>0.9999		D-F
Fam129b vs. *Mcm3*	0.03533	-0.03126 to 0.1019	No	ns		0.9044	D-G
Fam129b vs. *Nfe2l1*	-0.3974	-0.4640 to -0.3308	Yes	****	<0.0001		D-H
Fam129b vs. *Nfe2l2*	0.03445	-0.03214 to 0.1010	No	ns		0.9208	D-I
Fam129b vs. *Palb2*	0.0005269	-0.06606 to 0.06712	No	ns	>0.9999		D-J
Fam129b vs. *Pgam5*	-0.4434	-0.5100 to -0.3768	Yes	****	<0.0001		D-K
Fam129b vs. *Ptma*	-0.01046	-0.07705 to 0.05613	No	ns	>0.9999		D-L
Fam129b vs. *Slk*	-0.2202	-0.2868 to -0.1537	Yes	****	<0.0001		D-M
Fam129b vs. *Sqstm1*	-0.3947	-0.4613 to -0.3281	Yes	****	<0.0001		D-N
Fam129b vs. *Tsc22d4*	0.02907	-0.03752 to 0.09566	No	ns		0.9815	D-O
Fam129b vs. *Wdr1*	-0.1427	-0.2093 to -0.07615	Yes	****	<0.0001		D-P
Ikbkb vs. *Mad2l1*	0.1026	0.03600 to 0.1692	Yes	****	<0.0001		E-F
Ikbkb vs. *Mcm3*	0.1213	0.05467 to 0.1879	Yes	****	<0.0001		E-G
Ikbkb vs. *Nfe2l1*	-0.3114	-0.3780 to -0.2449	Yes	****	<0.0001		E-H
Ikbkb vs. *Nfe2l2*	0.1204	0.05379 to 0.1870	Yes	****	<0.0001		E-I
Ikbkb vs. *Palb2*	0.08646	0.01987 to 0.1530	Yes	**		0.0011	E-J
Ikbkb vs. *Pgam5*	-0.3574	-0.4240 to -0.2908	Yes	****	<0.0001		E-K
Ikbkb vs. *Ptma*	0.07547	0.008879 to 0.1421	Yes	*		0.0104	E-L
Ikbkb vs. *Slk*	-0.1343	-0.2009 to -0.06772	Yes	****	<0.0001		E-M
Ikbkb vs. *Sqstm1*	-0.3088	-0.3754 to -0.2422	Yes	****	<0.0001		E-N
Ikbkb vs. *Tsc22d4*	0.115	0.04841 to 0.1816	Yes	****	<0.0001		E-O

Ikbkb vs. *Wdr1*	-0.05681	-0.1234 to 0.009778	No	ns	0.1995	E-P
Mad2l1 vs. *Mcm3*	0.01867	-0.04792 to 0.08526	No	ns	0.9999	F-G
Mad2l1 vs. *Nfe2l1*	-0.414	-0.4806 to -0.3474	Yes	****	<0.0001	F-H
Mad2l1 vs. *Nfe2l2*	0.01779	-0.04880 to 0.08438	No	ns	>0.9999	F-I
Mad2l1 vs. *Palb2*	-0.01613	-0.08272 to 0.05046	No	ns	>0.9999	F-J
Mad2l1 vs. *Pgam5*	-0.46	-0.5266 to -0.3934	Yes	****	<0.0001	F-K
Mad2l1 vs. *Ptma*	-0.02712	-0.09371 to 0.03947	No	ns	0.9906	F-L
Mad2l1 vs. *Slk*	-0.2369	-0.3035 to -0.1703	Yes	****	<0.0001	F-M
Mad2l1 vs. *Sqstm1*	-0.4114	-0.4780 to -0.3448	Yes	****	<0.0001	F-N
Mad2l1 vs. *Tsc22d4*	0.01241	-0.05418 to 0.07900	No	ns	>0.9999	F-O
Mad2l1 vs. *Wdr1*	-0.1594	-0.2260 to -0.09281	Yes	****	<0.0001	F-P
Mcm3 vs. *Nfe2l1*	-0.4327	-0.4993 to -0.3661	Yes	****	<0.0001	G-H
Mcm3 vs. *Nfe2l2*	-0.0008777	-0.06747 to 0.06571	No	ns	>0.9999	G-I
Mcm3 vs. *Palb2*	-0.0348	-0.1014 to 0.03179	No	ns	0.9145	G-J
Mcm3 vs. *Pgam5*	-0.4787	-0.5453 to -0.4121	Yes	****	<0.0001	G-K
Mcm3 vs. *Ptma*	-0.04579	-0.1124 to 0.02080	No	ns	0.5705	G-L
Mcm3 vs. *Slk*	-0.2556	-0.3222 to -0.1890	Yes	****	<0.0001	G-M
Mcm3 vs. *Sqstm1*	-0.4301	-0.4966 to -0.3635	Yes	****	<0.0001	G-N
Mcm3 vs. *Tsc22d4*	-0.006258	-0.07285 to 0.06033	No	ns	>0.9999	G-O
Mcm3 vs. *Wdr1*	-0.1781	-0.2447 to -0.1115	Yes	****	<0.0001	G-P
Nfe2l1 vs. *Nfe2l2*	0.4318	0.3652 to 0.4984	Yes	****	<0.0001	H-I
Nfe2l1 vs. *Palb2*	0.3979	0.3313 to 0.4645	Yes	****	<0.0001	H-J
Nfe2l1 vs. *Pgam5*	-0.04599	-0.1126 to 0.02060	No	ns	0.5627	H-K
Nfe2l1 vs. *Ptma*	0.3869	0.3203 to 0.4535	Yes	****	<0.0001	H-L
Nfe2l1 vs. *Slk*	0.1771	0.1105 to 0.2437	Yes	****	<0.0001	H-M
Nfe2l1 vs. *Sqstm1*	0.002651	-0.06394 to 0.06924	No	ns	>0.9999	H-N
Nfe2l1 vs. *Tsc22d4*	0.4264	0.3599 to 0.4930	Yes	****	<0.0001	H-O
Nfe2l1 vs. *Wdr1*	0.2546	0.1880 to 0.3212	Yes	****	<0.0001	H-P
Nfe2l2 vs. *Palb2*	-0.03393	-0.1005 to 0.03266	No	ns	0.9297	I-J
Nfe2l2 vs. *Pgam5*	-0.4778	-0.5444 to -0.4112	Yes	****	<0.0001	I-K
Nfe2l2 vs. *Ptma*	-0.04491	-0.1115 to 0.02167	No	ns	0.6047	I-L
Nfe2l2 vs. *Slk*	-0.2547	-0.3213 to -0.1881	Yes	****	<0.0001	I-M
Nfe2l2 vs. *Sqstm1*	-0.4292	-0.4958 to -0.3626	Yes	****	<0.0001	I-N
Nfe2l2 vs. *Tsc22d4*	-0.005381	-0.07197 to 0.06121	No	ns	>0.9999	I-O
Nfe2l2 vs. *Wdr1*	-0.1772	-0.2438 to -0.1106	Yes	****	<0.0001	I-P
Palb2 vs. *Pgam5*	-0.4439	-0.5105 to -0.3773	Yes	****	<0.0001	J-K
Palb2 vs. *Ptma*	-0.01099	-0.07758 to 0.05560	No	ns	>0.9999	J-L
Palb2 vs. *Slk*	-0.2208	-0.2874 to -0.1542	Yes	****	<0.0001	J-M
Palb2 vs. *Sqstm1*	-0.3953	-0.4618 to -0.3287	Yes	****	<0.0001	J-N
Palb2 vs. *Tsc22d4*	0.02854	-0.03804 to 0.09513	No	ns	0.9844	J-O
Palb2 vs. *Wdr1*	-0.1433	-0.2099 to -0.07668	Yes	****	<0.0001	J-P
Pgam5 vs. *Ptma*	0.4329	0.3663 to 0.4995	Yes	****	<0.0001	K-L
Pgam5 vs. *Slk*	0.2231	0.1565 to 0.2897	Yes	****	<0.0001	K-M
Pgam5 vs. *Sqstm1*	0.04864	-0.01795 to 0.1152	No	ns	0.4599	K-N
Pgam5 vs. *Tsc22d4*	0.4724	0.4058 to 0.5390	Yes	****	<0.0001	K-O
Pgam5 vs. *Wdr1*	0.3006	0.2340 to 0.3672	Yes	****	<0.0001	K-P
Ptma vs. *Slk*	-0.2098	-0.2764 to -0.1432	Yes	****	<0.0001	L-M

Ptma vs. *Sqstm1*	-0.3843	-0.4509 to -0.3177	Yes	****	<0.0001	L-N
Ptma vs. *Tsc22d4*	0.03953	-0.02706 to 0.1061	No	ns	0.7969	L-O
Ptma vs. *Wdr1*	-0.1323	-0.1989 to -0.06569	Yes	****	<0.0001	L-P
Slk vs. *Sqstm1*	-0.1745	-0.2411 to -0.1079	Yes	****	<0.0001	M-N
Slk vs. *Tsc22d4*	0.2493	0.1827 to 0.3159	Yes	****	<0.0001	M-O
Slk vs. *Wdr1*	0.0775	0.01091 to 0.1441	Yes	**	0.007	M-P
Sqstm1 vs. *Tsc22d4*	0.4238	0.3572 to 0.4904	Yes	****	<0.0001	N-O
Sqstm1 vs. *Wdr1*	0.252	0.1854 to 0.3186	Yes	****	<0.0001	N-P
Tsc22d4 vs. *Wdr1*	-0.1718	-0.2384 to -0.1052	Yes	****	<0.0001	O-P

Test details	Mean 1	Mean 2	Mean Diff.	SE of diff.	n1	n2	q	DF
Amer1 vs. *Dpp3*	0.1072	0.2975	-0.1903	0.01931	26	26	13.93	400
Amer1 vs. *Fam117b*	0.1072	0.3394	-0.2322	0.01931	26	26	17.01	400
Amer1 vs. *Fam129b*	0.1072	0.03586	0.07134	0.01931	26	26	5.225	400
Amer1 vs. *Ikbkb*	0.1072	0.1218	-0.01459	0.01931	26	26	1.068	400
Amer1 vs. *Mad2l1*	0.1072	0.0192	0.088	0.01931	26	26	6.444	400
Amer1 vs. *Mcm3*	0.1072	0.0005252	0.1067	0.01931	26	26	7.812	400
Amer1 vs. *Nfe2l1*	0.1072	0.4332	-0.326	0.01931	26	26	23.88	400
Amer1 vs. *Nfe2l2*	0.1072	0.001403	0.1058	0.01931	26	26	7.748	400
Amer1 vs. *Palb2*	0.1072	0.03533	0.07187	0.01931	26	26	5.263	400
Amer1 vs. *Pgam5*	0.1072	0.4792	-0.372	0.01931	26	26	27.24	400
Amer1 vs. *Ptma*	0.1072	0.04632	0.06088	0.01931	26	26	4.458	400
Amer1 vs. *Slk*	0.1072	0.2561	-0.1489	0.01931	26	26	10.9	400
Amer1 vs. *Sqstm1*	0.1072	0.4306	-0.3234	0.01931	26	26	23.68	400
Amer1 vs. *Tsc22d4*	0.1072	0.006784	0.1004	0.01931	26	26	7.353	400
Amer1 vs. *Wdr1*	0.1072	0.1786	-0.0714	0.01931	26	26	5.229	400
Dpp3 vs. *Fam117b*	0.2975	0.3394	-0.04197	0.01931	26	26	3.074	400
Dpp3 vs. *Fam129b*	0.2975	0.03586	0.2616	0.01931	26	26	19.16	400
Dpp3 vs. *Ikbkb*	0.2975	0.1218	0.1757	0.01931	26	26	12.86	400
Dpp3 vs. *Mad2l1*	0.2975	0.0192	0.2783	0.01931	26	26	20.38	400
Dpp3 vs. *Mcm3*	0.2975	0.0005252	0.2969	0.01931	26	26	21.74	400
Dpp3 vs. *Nfe2l1*	0.2975	0.4332	-0.1358	0.01931	26	26	9.943	400
Dpp3 vs. *Nfe2l2*	0.2975	0.001403	0.2961	0.01931	26	26	21.68	400
Dpp3 vs. *Palb2*	0.2975	0.03533	0.2621	0.01931	26	26	19.2	400
Dpp3 vs. *Pgam5*	0.2975	0.4792	-0.1818	0.01931	26	26	13.31	400
Dpp3 vs. *Ptma*	0.2975	0.04632	0.2511	0.01931	26	26	18.39	400
Dpp3 vs. *Slk*	0.2975	0.2561	0.04136	0.01931	26	26	3.029	400
Dpp3 vs. *Sqstm1*	0.2975	0.4306	-0.1331	0.01931	26	26	9.749	400
Dpp3 vs. *Tsc22d4*	0.2975	0.006784	0.2907	0.01931	26	26	21.29	400
Dpp3 vs. *Wdr1*	0.2975	0.1786	0.1189	0.01931	26	26	8.704	400
Fam117b vs. *Fam129b*	0.3394	0.03586	0.3036	0.01931	26	26	22.23	400
Fam117b vs. *Ikbkb*	0.3394	0.1218	0.2176	0.01931	26	26	15.94	400
Fam117b vs. *Mad2l1*	0.3394	0.0192	0.3202	0.01931	26	26	23.45	400
Fam117b vs. *Mcm3*	0.3394	0.0005252	0.3389	0.01931	26	26	24.82	400
Fam117b vs. *Nfe2l1*	0.3394	0.4332	-0.0938	0.01931	26	26	6.869	400
Fam117b vs. *Nfe2l2*	0.3394	0.001403	0.338	0.01931	26	26	24.75	400
Fam117b vs. *Palb2*	0.3394	0.03533	0.3041	0.01931	26	26	22.27	400

Fam117b vs. *Pgam5*	0.3394	0.4792	-0.1398	0.01931	26	26	10.24	400
Fam117b vs. *Ptma*	0.3394	0.04632	0.2931	0.01931	26	26	21.47	400
Fam117b vs. *Slk*	0.3394	0.2561	0.08333	0.01931	26	26	6.103	400
Fam117b vs. *Sqstm1*	0.3394	0.4306	-0.09115	0.01931	26	26	6.675	400
Fam117b vs. *Tsc22d4*	0.3394	0.006784	0.3326	0.01931	26	26	24.36	400
Fam117b vs. *Wdr1*	0.3394	0.1786	0.1608	0.01931	26	26	11.78	400
Fam129b vs. *Ikbkb*	0.03586	0.1218	-0.08593	0.01931	26	26	6.293	400
Fam129b vs. *Mad2l1*	0.03586	0.0192	0.01666	0.01931	26	26	1.22	400
Fam129b vs. *Mcm3*	0.03586	0.0005252	0.03533	0.01931	26	26	2.587	400
Fam129b vs. *Nfe2l1*	0.03586	0.4332	-0.3974	0.01931	26	26	29.1	400
Fam129b vs. *Nfe2l2*	0.03586	0.001403	0.03445	0.01931	26	26	2.523	400
Fam129b vs. *Palb2*	0.03586	0.03533	0.0005269	0.01931	26	26	0.03859	400
Fam129b vs. *Pgam5*	0.03586	0.4792	-0.4434	0.01931	26	26	32.47	400
Fam129b vs. *Ptma*	0.03586	0.04632	-0.01046	0.01931	26	26	0.7662	400
Fam129b vs. *Slk*	0.03586	0.2561	-0.2202	0.01931	26	26	16.13	400
Fam129b vs. *Sqstm1*	0.03586	0.4306	-0.3947	0.01931	26	26	28.91	400
Fam129b vs. *Tsc22d4*	0.03586	0.006784	0.02907	0.01931	26	26	2.129	400
Fam129b vs. *Wdr1*	0.03586	0.1786	-0.1427	0.01931	26	26	10.45	400
Ikbkb vs. *Mad2l1*	0.1218	0.0192	0.1026	0.01931	26	26	7.513	400
Ikbkb vs. *Mcm3*	0.1218	0.0005252	0.1213	0.01931	26	26	8.88	400
Ikbkb vs. *Nfe2l1*	0.1218	0.4332	-0.3114	0.01931	26	26	22.81	400
Ikbkb vs. *Nfe2l2*	0.1218	0.001403	0.1204	0.01931	26	26	8.816	400
Ikbkb vs. *Palb2*	0.1218	0.03533	0.08646	0.01931	26	26	6.331	400
Ikbkb vs. *Pgam5*	0.1218	0.4792	-0.3574	0.01931	26	26	26.18	400
Ikbkb vs. *Ptma*	0.1218	0.04632	0.07547	0.01931	26	26	5.527	400
Ikbkb vs. *Slk*	0.1218	0.2561	-0.1343	0.01931	26	26	9.836	400
Ikbkb vs. *Sqstm1*	0.1218	0.4306	-0.3088	0.01931	26	26	22.61	400
Ikbkb vs. *Tsc22d4*	0.1218	0.006784	0.115	0.01931	26	26	8.422	400
Ikbkb vs. *Wdr1*	0.1218	0.1786	-0.05681	0.01931	26	26	4.16	400
Mad2l1 vs. *Mcm3*	0.0192	0.0005252	0.01867	0.01931	26	26	1.367	400
Mad2l1 vs. *Nfe2l1*	0.0192	0.4332	-0.414	0.01931	26	26	30.32	400
Mad2l1 vs. *Nfe2l2*	0.0192	0.001403	0.01779	0.01931	26	26	1.303	400
Mad2l1 vs. *Palb2*	0.0192	0.03533	-0.01613	0.01931	26	26	1.181	400
Mad2l1 vs. *Pgam5*	0.0192	0.4792	-0.46	0.01931	26	26	33.69	400
Mad2l1 vs. *Ptma*	0.0192	0.04632	-0.02712	0.01931	26	26	1.986	400
Mad2l1 vs. *Slk*	0.0192	0.2561	-0.2369	0.01931	26	26	17.35	400
Mad2l1 vs. *Sqstm1*	0.0192	0.4306	-0.4114	0.01931	26	26	30.13	400
Mad2l1 vs. *Tsc22d4*	0.0192	0.006784	0.01241	0.01931	26	26	0.909	400
Mad2l1 vs. *Wdr1*	0.0192	0.1786	-0.1594	0.01931	26	26	11.67	400
Mcm3 vs. *Nfe2l1*	0.0005252	0.4332	-0.4327	0.01931	26	26	31.69	400
Mcm3 vs. *Nfe2l2*	0.0005252	0.001403	-0.0008777	0.01931	26	26	0.06427	400
Mcm3 vs. *Palb2*	0.0005252	0.03533	-0.0348	0.01931	26	26	2.549	400
Mcm3 vs. *Pgam5*	0.0005252	0.4792	-0.4787	0.01931	26	26	35.06	400
Mcm3 vs. *Ptma*	0.0005252	0.04632	-0.04579	0.01931	26	26	3.353	400
Mcm3 vs. *Slk*	0.0005252	0.2561	-0.2556	0.01931	26	26	18.72	400
Mcm3 vs. *Sqstm1*	0.0005252	0.4306	-0.4301	0.01931	26	26	31.49	400
Mcm3 vs. *Tsc22d4*	0.0005252	0.006784	-0.006258	0.01931	26	26	0.4583	400

Mcm3 vs. *Wdr1*	0.0005252	0.1786	-0.1781	0.01931	26	26	13.04	400
Nfe2l1 vs. *Nfe2l2*	0.4332	0.001403	0.4318	0.01931	26	26	31.62	400
Nfe2l1 vs. *Palb2*	0.4332	0.03533	0.3979	0.01931	26	26	29.14	400
Nfe2l1 vs. *Pgam5*	0.4332	0.4792	-0.04599	0.01931	26	26	3.368	400
Nfe2l1 vs. *Ptma*	0.4332	0.04632	0.3869	0.01931	26	26	28.33	400
Nfe2l1 vs. *Slk*	0.4332	0.2561	0.1771	0.01931	26	26	12.97	400
Nfe2l1 vs. *Sqstm1*	0.4332	0.4306	0.002651	0.01931	26	26	0.1942	400
Nfe2l1 vs. *Tsc22d4*	0.4332	0.006784	0.4264	0.01931	26	26	31.23	400
Nfe2l1 vs. *Wdr1*	0.4332	0.1786	0.2546	0.01931	26	26	18.65	400
Nfe2l2 vs. *Palb2*	0.001403	0.03533	-0.03393	0.01931	26	26	2.484	400
Nfe2l2 vs. *Pgam5*	0.001403	0.4792	-0.4778	0.01931	26	26	34.99	400
Nfe2l2 vs. *Ptma*	0.001403	0.04632	-0.04491	0.01931	26	26	3.289	400
Nfe2l2 vs. *Slk*	0.001403	0.2561	-0.2547	0.01931	26	26	18.65	400
Nfe2l2 vs. *Sqstm1*	0.001403	0.4306	-0.4292	0.01931	26	26	31.43	400
Nfe2l2 vs. *Tsc22d4*	0.001403	0.006784	-0.005381	0.01931	26	26	0.394	400
Nfe2l2 vs. *Wdr1*	0.001403	0.1786	-0.1772	0.01931	26	26	12.98	400
Palb2 vs. *Pgam5*	0.03533	0.4792	-0.4439	0.01931	26	26	32.51	400
Palb2 vs. *Ptma*	0.03533	0.04632	-0.01099	0.01931	26	26	0.8048	400
Palb2 vs. *Slk*	0.03533	0.2561	-0.2208	0.01931	26	26	16.17	400
Palb2 vs. *Sqstm1*	0.03533	0.4306	-0.3953	0.01931	26	26	28.94	400
Palb2 vs. *Tsc22d4*	0.03533	0.006784	0.02854	0.01931	26	26	2.09	400
Palb2 vs. *Wdr1*	0.03533	0.1786	-0.1433	0.01931	26	26	10.49	400
Pgam5 vs. *Ptma*	0.4792	0.04632	0.4329	0.01931	26	26	31.7	400
Pgam5 vs. *Slk*	0.4792	0.2561	0.2231	0.01931	26	26	16.34	400
Pgam5 vs. *Sqstm1*	0.4792	0.4306	0.04864	0.01931	26	26	3.562	400
Pgam5 vs. *Tsc22d4*	0.4792	0.006784	0.4724	0.01931	26	26	34.6	400
Pgam5 vs. *Wdr1*	0.4792	0.1786	0.3006	0.01931	26	26	22.02	400
Ptma vs. *Slk*	0.04632	0.2561	-0.2098	0.01931	26	26	15.36	400
Ptma vs. *Sqstm1*	0.04632	0.4306	-0.3843	0.01931	26	26	28.14	400
Ptma vs. *Tsc22d4*	0.04632	0.006784	0.03953	0.01931	26	26	2.895	400
Ptma vs. *Wdr1*	0.04632	0.1786	-0.1323	0.01931	26	26	9.687	400
Slk vs. *Sqstm1*	0.2561	0.4306	-0.1745	0.01931	26	26	12.78	400
Slk vs. *Tsc22d4*	0.2561	0.006784	0.2493	0.01931	26	26	18.26	400
Slk vs. *Wdr1*	0.2561	0.1786	0.0775	0.01931	26	26	5.676	400
Sqstm1 vs. *Tsc22d4*	0.4306	0.006784	0.4238	0.01931	26	26	31.04	400
Sqstm1 vs. *Wdr1*	0.4306	0.1786	0.252	0.01931	26	26	18.45	400
Tsc22d4 vs. *Wdr1*	0.006784	0.1786	-0.1718	0.01931	26	26	12.58	400

Nfe2l2-Keap1-neuron

Number of families	1
Number of comparisons per family	28
Alpha	0.05

Dunn's multiple comparisons test	Mean rank diff.	Significant?	Summary	Adjusted P Value	
Non-neuron vs. Glutamatergic	104.6	Yes	****	<0.0001	A-B
Non-neuron vs. GABAergic (not PV)	144.4	Yes	****	<0.0001	A-C

Non-neuron vs. GABAergic (PV)	185	Yes	****	<0.0001	A-D
Non-neuron vs. SPNs	-72.93	No	ns	>0.9999	A-E
Non-neuron vs. Cholinergic	8.662	No	ns	>0.9999	A-F
Non-neuron vs. DAergic (not MB)	15.34	No	ns	>0.9999	A-G
Non-neuron vs. DAergic (MB)	40.87	No	ns	>0.9999	A-H
Glutamatergic vs. GABAergic (not PV)	39.74	No	ns	>0.9999	B-C
Glutamatergic vs. GABAergic (PV)	80.39	No	ns	0.2015	B-D
Glutamatergic vs. SPNs	-177.5	Yes	***	0.0003	B-E
Glutamatergic vs. Cholinergic	-95.95	No	ns	>0.9999	B-F
Glutamatergic vs. DAergic (not MB)	-89.28	No	ns	>0.9999	B-G
Glutamatergic vs. DAergic (MB)	-63.75	No	ns	>0.9999	B-H
GABAergic (not PV) vs. GABAergic (PV)	40.65	No	ns	>0.9999	C-D
GABAergic (not PV) vs. SPNs	-217.3	Yes	****	<0.0001	C-E
GABAergic (not PV) vs. Cholinergic	-135.7	No	ns	0.5541	C-F
GABAergic (not PV) vs. DAergic (not MB)	-129	No	ns	>0.9999	C-G
GABAergic (not PV) vs. DAergic (MB)	-103.5	No	ns	>0.9999	C-H
GABAergic (PV) vs. SPNs	-257.9	Yes	****	<0.0001	D-E
GABAergic (PV) vs. Cholinergic	-176.3	No	ns	0.1245	D-F
GABAergic (PV) vs. DAergic (not MB)	-169.7	No	ns	0.6927	D-G
GABAergic (PV) vs. DAergic (MB)	-144.1	No	ns	0.4144	D-H
SPNs vs. Cholinergic	81.6	No	ns	>0.9999	E-F
SPNs vs. DAergic (not MB)	88.27	No	ns	>0.9999	E-G
SPNs vs. DAergic (MB)	113.8	No	ns	>0.9999	E-H
Cholinergic vs. DAergic (not MB)	6.675	No	ns	>0.9999	F-G
Cholinergic vs. DAergic (MB)	32.21	No	ns	>0.9999	F-H
DAergic (not MB) vs. DAergic (MB)	25.53	No	ns	>0.9999	G-H

Test details	Mean rank 1	Mean rank 2	Mean rank diff.	n1	n2	Z
Non-neuron vs. Glutamatergic	336.5	231.9	104.6	231	153	6.362
Non-neuron vs. GABAergic (not PV)	336.5	192.2	144.4	231	89	7.334
Non-neuron vs. GABAergic (PV)	336.5	151.5	185	231	34	6.384
Non-neuron vs. SPNs	336.5	409.5	-72.93	231	17	1.84
Non-neuron vs. Cholinergic	336.5	327.9	8.662	231	8	0.1527
Non-neuron vs. DAergic (not MB)	336.5	321.2	15.34	231	5	0.2151
Non-neuron vs. DAergic (MB)	336.5	295.7	40.87	231	9	0.7625
Glutamatergic vs. GABAergic (not PV)	231.9	192.2	39.74	153	89	1.89
Glutamatergic vs. GABAergic (PV)	231.9	151.5	80.39	153	34	2.688
Glutamatergic vs. SPNs	231.9	409.5	-177.5	153	17	4.402
Glutamatergic vs. Cholinergic	231.9	327.9	-95.95	153	8	1.677
Glutamatergic vs. DAergic (not MB)	231.9	321.2	-89.28	153	5	1.245
Glutamatergic vs. DAergic (MB)	231.9	295.7	-63.75	153	9	1.178
GABAergic (not PV) vs. GABAergic (PV)	192.2	151.5	40.65	89	34	1.278
GABAergic (not PV) vs. SPNs	192.2	409.5	-217.3	89	17	5.204
GABAergic (not PV) vs. Cholinergic	192.2	327.9	-135.7	89	8	2.33
GABAergic (not PV) vs. DAergic (not MB)	192.2	321.2	-129	89	5	1.779
GABAergic (not PV) vs. DAergic (MB)	192.2	295.7	-103.5	89	9	1.875
GABAergic (PV) vs. SPNs	151.5	409.5	-257.9	34	17	5.504

GABAergic (PV) vs. Cholinergic	151.5	327.9	-176.3	34	8	2.845
GABAergic (PV) vs. DAergic (not MB)	151.5	321.2	-169.7	34	5	2.245
GABAergic (PV) vs. DAergic (MB)	151.5	295.7	-144.1	34	9	2.437
SPNs vs. Cholinergic	409.5	327.9	81.6	17	8	1.206
SPNs vs. DAergic (not MB)	409.5	321.2	88.27	17	5	1.1
SPNs vs. DAergic (MB)	409.5	295.7	113.8	17	9	1.75
Cholinergic vs. DAergic (not MB)	327.9	321.2	6.675	8	5	0.07422
Cholinergic vs. DAergic (MB)	327.9	295.7	32.21	8	9	0.4202
DAergic (not MB) vs. DAergic (MB)	321.2	295.7	25.53	5	9	0.2902

Park7-Keap1-neuron

Number of families	1
Number of comparisons per family	28
Alpha	0.05

Dunn's multiple comparisons test	Mean rank diff.	Significant?	Summary	Adjusted P Value	
Non-neuron vs. Glutamatergic	113.2	Yes	****	<0.0001	A-B
Non-neuron vs. GABAergic (not PV)	127.1	Yes	****	<0.0001	A-C
Non-neuron vs. GABAergic (PV)	146.5	Yes	****	<0.0001	A-D
Non-neuron vs. SPNs	-60.32	No	ns	>0.9999	A-E
Non-neuron vs. Cholinergic	-25.52	No	ns	>0.9999	A-F
Non-neuron vs. DAergic (not MB)	-13.95	No	ns	>0.9999	A-G
Non-neuron vs. DAergic (MB)	72.3	No	ns	>0.9999	A-H
Glutamatergic vs. GABAergic (not PV)	13.9	No	ns	>0.9999	B-C
Glutamatergic vs. GABAergic (PV)	33.35	No	ns	>0.9999	B-D
Glutamatergic vs. SPNs	-173.5	Yes	***	0.0005	B-E
Glutamatergic vs. Cholinergic	-138.7	No	ns	0.4302	B-F
Glutamatergic vs. DAergic (not MB)	-127.1	No	ns	>0.9999	B-G
Glutamatergic vs. DAergic (MB)	-40.86	No	ns	>0.9999	B-H
GABAergic (not PV) vs. GABAergic (PV)	19.44	No	ns	>0.9999	C-D
GABAergic (not PV) vs. SPNs	-187.4	Yes	***	0.0002	C-E
GABAergic (not PV) vs. Cholinergic	-152.6	No	ns	0.246	C-F
GABAergic (not PV) vs. DAergic (not MB)	-141	No	ns	>0.9999	C-G
GABAergic (not PV) vs. DAergic (MB)	-54.76	No	ns	>0.9999	C-H
GABAergic (PV) vs. SPNs	-206.8	Yes	***	0.0003	D-E
GABAergic (PV) vs. Cholinergic	-172	No	ns	0.1546	D-F
GABAergic (PV) vs. DAergic (not MB)	-160.4	No	ns	0.9443	D-G
GABAergic (PV) vs. DAergic (MB)	-74.2	No	ns	>0.9999	D-H
SPNs vs. Cholinergic	34.8	No	ns	>0.9999	E-F
SPNs vs. DAergic (not MB)	46.38	No	ns	>0.9999	E-G
SPNs vs. DAergic (MB)	132.6	No	ns	>0.9999	E-H
Cholinergic vs. DAergic (not MB)	11.58	No	ns	>0.9999	F-G
Cholinergic vs. DAergic (MB)	97.82	No	ns	>0.9999	F-H
DAergic (not MB) vs. DAergic (MB)	86.24	No	ns	>0.9999	G-H

Test details	Mean rank 1	Mean rank 2	Mean rank diff.	n1	n2	Z

Non-neuron vs. Glutamatergic	333.9	220.7	113.2	231	153	6.881
Non-neuron vs. GABAergic (not PV)	333.9	206.8	127.1	231	89	6.455
Non-neuron vs. GABAergic (PV)	333.9	187.4	146.5	231	34	5.055
Non-neuron vs. SPNs	333.9	394.2	-60.32	231	17	1.522
Non-neuron vs. Cholinergic	333.9	359.4	-25.52	231	8	0.4499
Non-neuron vs. DAergic (not MB)	333.9	347.8	-13.95	231	5	0.1956
Non-neuron vs. DAergic (MB)	333.9	261.6	72.3	231	9	1.349
Glutamatergic vs. GABAergic (not PV)	220.7	206.8	13.9	153	89	0.661
Glutamatergic vs. GABAergic (PV)	220.7	187.4	33.35	153	34	1.115
Glutamatergic vs. SPNs	220.7	394.2	-173.5	153	17	4.301
Glutamatergic vs. Cholinergic	220.7	359.4	-138.7	153	8	2.424
Glutamatergic vs. DAergic (not MB)	220.7	347.8	-127.1	153	5	1.773
Glutamatergic vs. DAergic (MB)	220.7	261.6	-40.86	153	9	0.755
GABAergic (not PV) vs. GABAergic (PV)	206.8	187.4	19.44	89	34	0.6113
GABAergic (not PV) vs. SPNs	206.8	394.2	-187.4	89	17	4.487
GABAergic (not PV) vs. Cholinergic	206.8	359.4	-152.6	89	8	2.62
GABAergic (not PV) vs. DAergic (not MB)	206.8	347.8	-141	89	5	1.945
GABAergic (not PV) vs. DAergic (MB)	206.8	261.6	-54.76	89	9	0.9923
GABAergic (PV) vs. SPNs	187.4	394.2	-206.8	34	17	4.413
GABAergic (PV) vs. Cholinergic	187.4	359.4	-172	34	8	2.775
GABAergic (PV) vs. DAergic (not MB)	187.4	347.8	-160.4	34	5	2.123
GABAergic (PV) vs. DAergic (MB)	187.4	261.6	-74.2	34	9	1.255
SPNs vs. Cholinergic	394.2	359.4	34.8	17	8	0.5145
SPNs vs. DAergic (not MB)	394.2	347.8	46.38	17	5	0.5778
SPNs vs. DAergic (MB)	394.2	261.6	132.6	17	9	2.039
Cholinergic vs. DAergic (not MB)	359.4	347.8	11.58	8	5	0.1287
Cholinergic vs. DAergic (MB)	359.4	261.6	97.82	8	9	1.276
DAergic (not MB) vs. DAergic (MB)	347.8	261.6	86.24	5	9	0.9801

Sqstm1-Keap1-neuron

Number of families	1
Number of comparisons per family	28
Alpha	0.05

Dunn's multiple comparisons test	Mean rank diff.	Significant?	Summary	Adjusted P Value	
Non-neuron vs. Glutamatergic	-19.6	No	ns	>0.9999	A-B
Non-neuron vs. GABAergic (not PV)	-26.09	No	ns	>0.9999	A-C
Non-neuron vs. GABAergic (PV)	143	Yes	****	<0.0001	A-D
Non-neuron vs. SPNs	-118.1	No	ns	0.081	A-E
Non-neuron vs. Cholinergic	-150.7	No	ns	0.2215	A-F
Non-neuron vs. DAergic (not MB)	-40.61	No	ns	>0.9999	A-G
Non-neuron vs. DAergic (MB)	48.08	No	ns	>0.9999	A-H
Glutamatergic vs. GABAergic (not PV)	-6.484	No	ns	>0.9999	B-C
Glutamatergic vs. GABAergic (PV)	162.6	Yes	****	<0.0001	B-D
Glutamatergic vs. SPNs	-98.5	No	ns	0.4088	B-E
Glutamatergic vs. Cholinergic	-131.1	No	ns	0.6151	B-F

Glutamatergic vs. DAergic (not MB)	-21	No	ns	>0.9999	B-G
Glutamatergic vs. DAergic (MB)	67.69	No	ns	>0.9999	B-H
GABAergic (not PV) vs. GABAergic (PV)	169.1	Yes	****	<0.0001	C-D
GABAergic (not PV) vs. SPNs	-92.01	No	ns	0.7716	C-E
GABAergic (not PV) vs. Cholinergic	-124.6	No	ns	0.9066	C-F
GABAergic (not PV) vs. DAergic (not MB)	-14.52	No	ns	>0.9999	C-G
GABAergic (not PV) vs. DAergic (MB)	74.17	No	ns	>0.9999	C-H
GABAergic (PV) vs. SPNs	-261.1	Yes	****	<0.0001	D-E
GABAergic (PV) vs. Cholinergic	-293.7	Yes	****	<0.0001	D-F
GABAergic (PV) vs. DAergic (not MB)	-183.6	No	ns	0.4231	D-G
GABAergic (PV) vs. DAergic (MB)	-94.91	No	ns	>0.9999	D-H
SPNs vs. Cholinergic	-32.58	No	ns	>0.9999	E-F
SPNs vs. DAergic (not MB)	77.49	No	ns	>0.9999	E-G
SPNs vs. DAergic (MB)	166.2	No	ns	0.297	E-H
Cholinergic vs. DAergic (not MB)	110.1	No	ns	>0.9999	F-G
Cholinergic vs. DAergic (MB)	198.8	No	ns	0.2665	F-H
DAergic (not MB) vs. DAergic (MB)	88.69	No	ns	>0.9999	G-H

Test details	Mean rank 1	Mean rank 2	Mean rank diff.	n1	n2	Z
Non-neuron vs. Glutamatergic	267.2	286.8	-19.6	231	153	1.192
Non-neuron vs. GABAergic (not PV)	267.2	293.3	-26.09	231	89	1.325
Non-neuron vs. GABAergic (PV)	267.2	124.2	143	231	34	4.934
Non-neuron vs. SPNs	267.2	385.3	-118.1	231	17	2.979
Non-neuron vs. Cholinergic	267.2	417.9	-150.7	231	8	2.656
Non-neuron vs. DAergic (not MB)	267.2	307.8	-40.61	231	5	0.5694
Non-neuron vs. DAergic (MB)	267.2	219.1	48.08	231	9	0.8971
Glutamatergic vs. GABAergic (not PV)	286.8	293.3	-6.484	153	89	0.3083
Glutamatergic vs. GABAergic (PV)	286.8	124.2	162.6	153	34	5.436
Glutamatergic vs. SPNs	286.8	385.3	-98.5	153	17	2.442
Glutamatergic vs. Cholinergic	286.8	417.9	-131.1	153	8	2.291
Glutamatergic vs. DAergic (not MB)	286.8	307.8	-21	153	5	0.2929
Glutamatergic vs. DAergic (MB)	286.8	219.1	67.69	153	9	1.251
GABAergic (not PV) vs. GABAergic (PV)	293.3	124.2	169.1	89	34	5.316
GABAergic (not PV) vs. SPNs	293.3	385.3	-92.01	89	17	2.204

GABAergic (not PV) vs. Cholinergic	293.3	417.9	-124.6	89	8	2.14
GABAergic (not PV) vs. DAergic (not MB)	293.3	307.8	-14.52	89	5	0.2002
GABAergic (not PV) vs. DAergic (MB)	293.3	219.1	74.17	89	9	1.344
GABAergic (PV) vs. SPNs	124.2	385.3	-261.1	34	17	5.571
GABAergic (PV) vs. Cholinergic	124.2	417.9	-293.7	34	8	4.737
GABAergic (PV) vs. DAergic (not MB)	124.2	307.8	-183.6	34	5	2.43
GABAergic (PV) vs. DAergic (MB)	124.2	219.1	-94.91	34	9	1.605
SPNs vs. Cholinergic	385.3	417.9	-32.58	17	8	0.4817
SPNs vs. DAergic (not MB)	385.3	307.8	77.49	17	5	0.9655
SPNs vs. DAergic (MB)	385.3	219.1	166.2	17	9	2.555
Cholinergic vs. DAergic (not MB)	417.9	307.8	110.1	8	5	1.224
Cholinergic vs. DAergic (MB)	417.9	219.1	198.8	8	9	2.593
DAergic (not MB) vs. DAergic (MB)	307.8	219.1	88.69	5	9	1.008

0
